# Australian Marine and Terrestrial *Streptomyces*-Derived Surugamides, and Synthetic Analogs, and Their Ability to Inhibit *Dirofilaria immitis* (Heartworm) Motility

**DOI:** 10.3390/md22070312

**Published:** 2024-07-09

**Authors:** Taizong Wu, Waleed M. Hussein, Kaumadi Samarasekera, Yuxuan Zhu, Zeinab G. Khalil, Shengbin Jin, David F. Bruhn, Yovany Moreno, Angela A. Salim, Robert J. Capon

**Affiliations:** 1 Institute for Molecular Bioscience, The University of Queensland, Brisbane, QLD 4072, Australia; wutaizong@tio.org.cn (T.W.); w.hussein@uq.edu.au (W.M.H.); kaumadi@sci.sjp.ac.lk (K.S.); yuxuan.zhu1@uq.net.au (Y.Z.); z.khalil@uq.edu.au (Z.G.K.); shengbin.jin@uq.edu.au (S.J.); 2 Boehringer Ingelheim Animal Health, USA Inc., 1730 Olympic Drive, Athens, GA 30601, USA; david.bruhn@boehringer-ingelheim.com (D.F.B.); yovany.moreno@boehringer-ingelheim.com (Y.M.)

**Keywords:** antimycins, surugamides, acyl-surugamides, anthelmintic, heartworm, *Dirofilaria immitis*, *Haemonchus contortus*, *Streptomyces* sp. CMB-M0112, *Streptomyces* sp. CMB-MRB032

## Abstract

A bioassay-guided chemical investigation of a bacterium, *Streptomyces* sp. CMB-MRB032, isolated from sheep feces collected near Bathurst, Victoria, Australia, yielded the known polyketide antimycins A4a (**1**) and A2a (**2**) as potent inhibitors of *Dirofilaria immitis* (heartworm) microfilaria (mf) motility (EC_50_ 0.0013–0.0021 µg/mL), along with the octapeptide surugamide A (**3**) and the new *N*-methylated analog surugamide K (**4**). With biological data suggesting surugamides may also exhibit activity against *D. immitis*, a GNPS molecular network analysis of a library of microbes sourced from geographically diverse Australian ecosystems identified a further five taxonomically and chemically distinct surugamide producers. Scaled-up cultivation of one such producer, *Streptomyces* sp. CMB-M0112 isolated from a marine sediment collected at Shorncliff, Qld, Australia, yielded **3** along with the new acyl-surugamides A1–A4 (**5**–**8**). Solid-phase peptide synthesis provided additional synthetic analogs, surugamides S1–S3 (**9**–**11**), while derivatization of **3** returned the semi-synthetic surugamide S4 (**12**) and acyl-surugamides AS1–AS3 (**13**–**15**). The natural acyl-surugamide A3 (**7**) and semi-synthetic acyl-surugamide AS3 (**15**) were shown to selectively inhibit *D. immitis* mf motility (EC_50_ 3.3–3.4 µg/mL), however, unlike antimycins **1** and **2**, were inactive against the gastrointestinal nematode *Haemonchus contortus* L1–L3 larvae (EC_50_ > 25 µg/mL) and were not cytotoxic to mammalian cells (human colorectal carcinoma SW620, IC_50_ > 30 µg/mL). A structure–activity relationship (SAR) study on the surugamides **3**–**15** revealed that selective acylation of the Lys^3^-ε-NH_2_ correlates with anthelmintic activity.

## 1. Introduction

Helminth infections in commercial livestock are of major socio-economic importance worldwide [1]. First discovered from soil-dwelling *Streptomyces* in the 1980s, the macrolactone ivermectin and related analogs have emerged as the gold standard for the treatment of helminth infections [2]. In addition to safeguarding livestock, anthelmintics such as macrolactones are important for the health and welfare of companion animals (e.g., dogs and cats). With respect to companion animals, *Dirofilaria immitis* (also known as heartworm) is a major parasitic threat that causes chronic damage to the heart, lungs, and arteries that can result in complications leading to fatal outcomes in dogs, or severe respiratory distress and even sudden death in cats [3]. *D. immitis* is a filarial nematode with a complex lifecycle spanning mosquito vectors (microfilariae—L3 larvae) and mammalian hosts (L3 and L4 larvae, and adult). Adult *D. immitis* reside in the pulmonary arteries and in the right ventricle of the host, releasing microfilariae (mf) into the bloodstream. If ingested by mosquito vectors of the genera *Culex*, *Aedes,* or *Anopheles*, the mf develop from L1 larvae to infective L3 larvae [3]. While anthelmintic macrolactones are the mainstay for preventive control of heartworm infections, the incidence and severity of drug-resistant helminth infections have compromised the efficacy of most marketed classes of commercial anthelmintics (whether used to treat commercial livestock or companion animals). Hence, there is an urgent need to identify new chemical classes that anticipate the spread of resistance and facilitate enhanced user compliance in order to discover, develop, and bring new and improved anthelmintics to the market.

As noted in a recent 2023 review [4], fungi and bacteria can be a prolific source of natural products exhibiting anthelmintic properties, several classes of which have been developed into commercial products. Inspired by these successes, we screened extracts of microbes isolated from diverse substrates sampled across Australia, against both the heartworm *D. immitis* and livestock gastrointestinal parasite *Haemonchus contortus*. This screen identified numerous extracts that exhibited potent in vitro inhibition of *D. immitis* mf motility, including *Streptomyces* sp. CMB-MRB032 recovered from nematode-infected sheep feces from a farm near Bathurst, VIC, Australia.

Although a preliminary bioassay-guided fractionation supported by a Global Natural Products Social (GNPS) analysis [5] attributed the anthelmintic activity in an ISP2 extract of CMB-MRB032 to the well–known antimycin class of cytotoxin [6], it was also noted that several active fractions incorporated surugamides, a relatively rare class of cyclic octapeptide [7,8]. With the cytotoxic properties of antimycins making them ill suited for development as anthelmintics [9], our attention was drawn to the possible anthelmintic potential of the surugamides. In an effort to supplement the low level of surugamide production exhibited by CMB-MRB032, we expanded our GNPS molecular network search to include a more expansive and diverse inhouse microbe library, leading to the detection of multiple additional isolates capable of producing surugamides. Of note, *Streptomyces* sp. CMB-M0112 isolated from a marine sediment collected near Shorncliffe, Qld., Australia, was a particularly promising surugamide producer. Herein, we report on the isolation, structure characterization, and anthelmintic potential of known and new natural surugamides from *Streptomyces* spp. CMB-MRB032 and CMB-M0112, as well as an array of semi-synthetic and synthetic surugamides.

## 2. Results and Discussion

**Figure 1:**
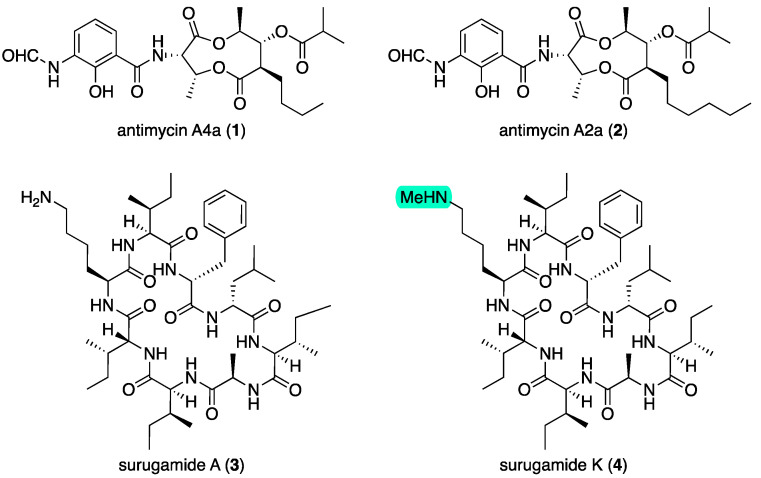
Metabolites **1**–**4** from CMB-MRB032. Green highlight—variation in the Lys^3^ moiety relative to surugamide A (**3**).

**Figure 2:**
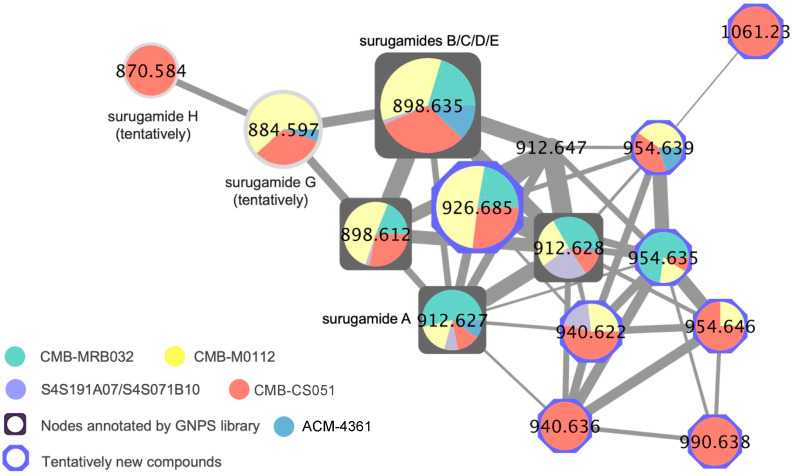
The surugamide molecular family from a GNPS molecular network of surugamide producers cultivated on ISP2 agar, highlighting surugamides A–E, and G–H, and new analogs.

The EtOAc extract derived from an ISP2 agar culture of *Streptomyces* CMB-MRB032 exhibited potent activity against *D. immitis* mf motility (EC_50_ 0.06 μg/mL) with notable but lower activity against *H. contortus* in L1–L3 development (EC_50_ 3.0 μg/mL). Subsequent medium-scale cultivation (80 × ISP2 agar plates) followed by bioassay-guided isolation and GNPS analysis revealed surugamides and antimycins in active fractions (Figures S3 and S4). Consistent with the activity profile of the unfractionated extract, samples of antimycins A4a (**1**) and A2a (**2**) (Figure 1) obtained from our pure compound library (Figures S79–S82) exhibited potent activity against *D. immitis* (EC_50_ 0.0013 and 0.0021 μg/mL) and lesser activity against *H. contortus* (EC_50_ 3.5 and 1.8 μg/mL), with significant cytotoxicity towards SW620 human colon carcinoma cells (IC_50_ 0.061 and 0.080 μg/mL). Subsequent large-scale cultivation of CMB-MRB032 (400 × ISP2 agar plates) permitted isolation of the known surugamide A (**3**) [7,8] and new analog surugamide K (**4**) (Figure 1), with structures identified on the basis of detailed spectroscopic and Marfey’s analysis (as outlined below). GNPS analysis of the crude extract also allowed the detection of the known surugamides B–E [7] and putative new analogs as minor co-metabolites (Figure 2). As neither **3** nor **4** exhibited activity against either *D. immitis* or *H. contortus* (EC_50_ > 25 μg/mL), we turned our attention to investigating minor surugamide co-metabolites (including from alternate producers).

As noted above, a GNPS molecular network analysis of a library of 1712 microbial extracts identified 15 additional surugamide producers (Figure S5), all of which had been screened and shown to inhibit *D. immitis* mf motility (Table S1). Comparative UPLC-DAD analysis permitted the exclusion of replicates, prioritizing the available unique surugamide producers such as *Streptomyces* spp. CMB-MRB032, CMB-M0112, S4S-00191A07, S4S-00007B10, CMB-CS051, and ACM-4361 (Table 1), with a comparison of 16S rRNA sequences confirming taxonomic diversity (Table S2 and Figure S6). While a comparative analysis of GNPS molecular networks confirmed that all produced surugamides A–E (and some surugamides G–H), as well as new analogs (Table 1 and Figure 2), a single-ion extraction of the UPLC-QTOF data proved more effective at detecting antimycins, which were shown to be present in selected extracts (Table 1 and Figure S78).

**Table 1:** Microbial surugamide producers, and chemical and biological analysis of ISP2 extracts.

Microbial Isolate	Source	Chemical Analysis		Biological Analysis	
		Known Surugamides Detected (GNPS) ^f^	Known Antimycins Detected (SIE-MS)	*D. immitis* mf Motility EC_50_ (μg/mL)	*H. contortus* L1–L3 Larval Development EC_50_ (μg/mL)
*Streptomyces* sp. CMB-MRB032	terrestrial ^a^	A–E, G	A1, A2a, A4a	0.06	3.0
*Streptomyces* sp. CMB-M0112	marine ^b^	A–E, G	A2a ^g^, A4a ^g^	1.2	>20
*Streptomyces* sp. S4S-00191A07	terrestrial ^c^	A–E ^g^	A2a ^g^, A4a ^g^	<25 ^h^	<25 ^h^
*Streptomyces* sp. S4S-00007B10	terrestrial ^c^	A–E ^g^	n.a.	<25 ^h^	<25 ^h^
*Streptomyces* sp. CMB-CS051	marine ^d^	A–E, G–H	A1, A2a, A4a ^g^	<25 ^h^	>25
*Streptomyces sampsonii* ACM-4361	terrestrial ^e^	A–E ^g^, G	n.a.	5.2	<25 ^h^

^a^ sheep feces; ^b^ sediment; ^c^ soil; ^d^ cone snail; ^e^ potato scab; ^f^ as B–E share a common node, at least one and possibly all are present; ^g^ very minor; ^h^ EC_100_ based on single-dose test; n.a.: not analyzed.

**Figure 3:**
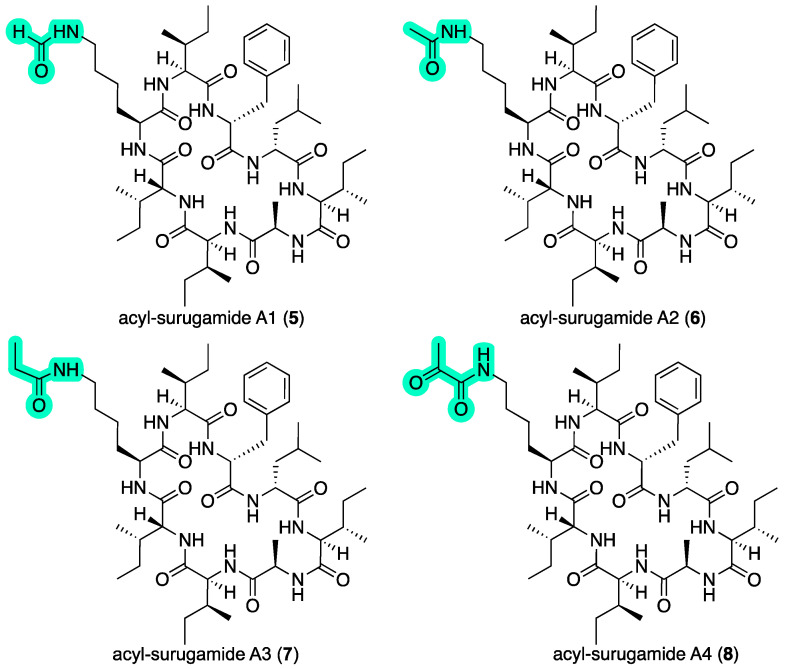
Acyl-surugamides from CMB-M0112. Green highlights—variation in the Lys^3^ moiety relative to surugamide A (**3**).

To improve our access to new surugamides, we elected to investigate CMB-M0112, which produced putative new surugamides with *m*/*z* 926 (C_49_H_83_N_9_O_8_), *m*/*z* 940 (C_49_H_81_N_9_O_9_), and *m*/*z* 954 (C_50_H_83_N_9_O_9_). Miniaturized microbial cultivation profiling (MATRIX) [10] of CMB-M0112 in ×11 media under ×3 conditions inclusive of a time study, revealed optimal yields of new surugamides on ISP2 agar up to 28 days (Figure S8). Based on the above, fractionation of a large-scale 21-day cultivation (380 × ISP2 agar plates) yielded the new acyl-surugamides A1–A4 (**5**–**8**) (Figure 3).

### 2.1. Structure Elucidation of Surugamides from Streptomyces sp. CMB-MRB032

HRESI(+)MS analysis of **3** returned a molecular formula (C_48_H_81_N_9_O_8_, Δmmu +0.6), which together with the UV–vis and NMR (DMSO-*d*_6_) data (Table 2 and Table 3 and Table S4, Figures S10 and S11) were consistent with the known octapeptide surugamide A. First isolated in 2015 from the Japanese deep-sea sediment-derived *Streptomyces* sp. JAMM992, surugamide A was originally assigned as *cyclo*[-l-Ile^1^-d-Ile^2^-l-Lys^3^-l-Ile^4^-d-Phe^5^-d-Leu^6^-l-Ile^7^-d-Ala^8^-] (**9**) [7]. A subsequent 2019 report on its total synthesis led to the d-Ile^2^ residue being revised to d-*allo*-Ile^2^ [8]. Alert to the potential for misassignment when differentiating between Ile and *allo*-Ile residues [11], we elected to synthesize both the d-*allo*-Ile^2^ (**3**) and a d-Ile^2^ (**9**) isomers of surugamide A. Comparison of the 1D and 2D NMR (DMSO-*d*_6_) data (Figures S48–S51) and an HPLC-DAD co-injection of natural and synthetic isomers (Figure S52) confirmed that surugamide A (**3**) did indeed incorporate a d-*allo*-Ile^2^ residue. Having acquired a comprehensive NMR data set for **3** and **9**, we take this opportunity to revise NMR assignments for **3** (Table S4) and assign a trivial name to the synthetic analog d-Ile^2^ surugamide A as surugamide S1 (**9**).

**Figure 4:**
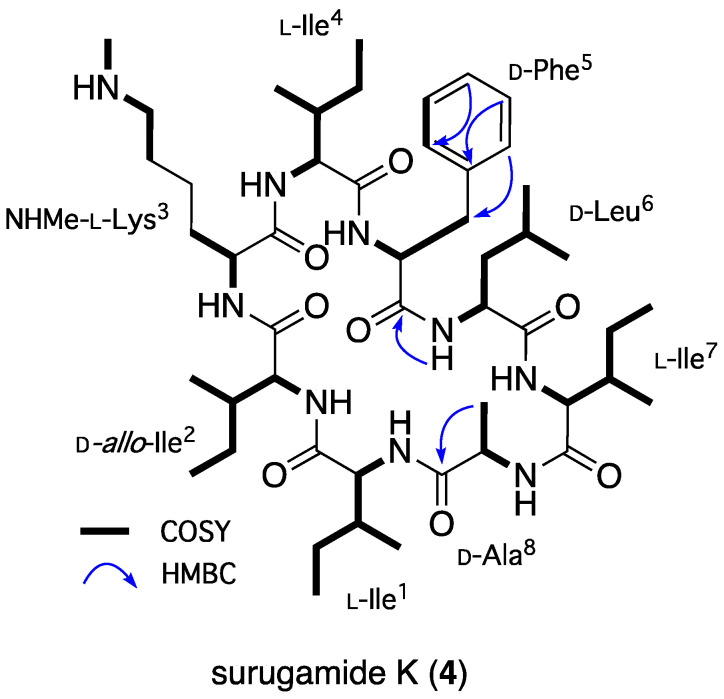
Selected HMBC and COSY NMR (DMSO-*d*_6_) correlations for **4**.

HRESI(+)MS analysis of **4** returned a molecular formula (C_49_H_83_N_9_O_8_, Δmmu +3.0) consistent with a homolog (+CH_2_) of **3**. Comparison of the NMR (DMSO-*d*_6_) data for **4** (Table 2 and Table 3 and Table S5, Figures S12–S17) with **3** attributed the principal differences to *N*-methylation of the Lys^3^-ε-NH_2_ (NHCH_3_ *δ*_H_ 2.54; *δ*_C_ 32.5; NHCH_3_, *δ*_H_ 8.41, s), with 2D NMR correlations (Figure 4) and an MS/MS analysis (Figure S47) establishing the planar structure, and a Marfey’s analysis (Figure S9) confirming the absolute configuration of surugamide K (**4**).

### 2.2. Structure Elucidation of Surugamides from Streptomyces sp. CMB-M0112

**Figure 5:**
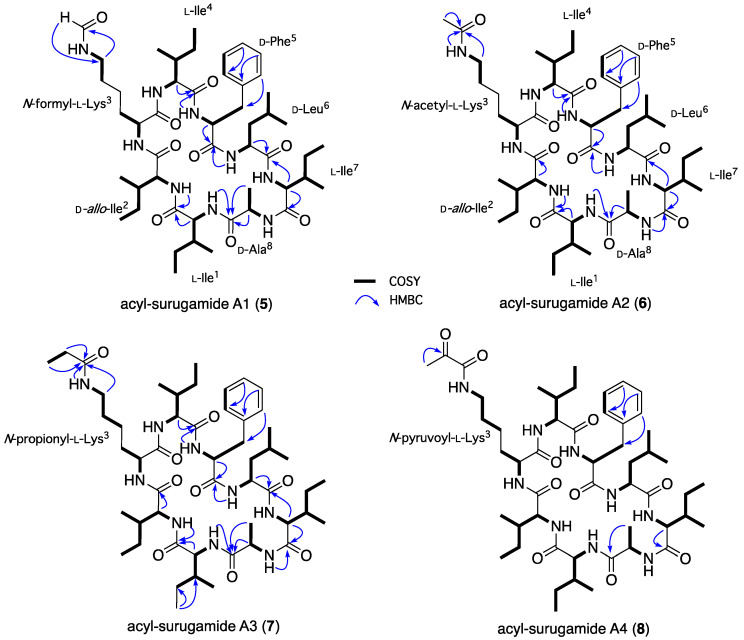
Selected HMBC and COSY NMR (DMSO-*d*_6_) correlations for **5**–**8**.

HRESI(+)MS analysis of **5** revealed a molecular formula (C_49_H_81_N_9_O_9_, Δmmu –7.4) consistent with a formylated (+CO) analog of **3**. Comparison of the NMR (DMSO-*d*_6_) data for **5** (Table 2 and Table 3 and Table S6, Figure 5 and Figure S19–S24) with **3** supported this hypothesis, with the principal differences attributed to an *N*-formyl-Lys^3^ moiety (*NH*-CHO *δ*_H_ 7.97; *δ*_C_ 161.0), and diagnostic HMBC correlations (Figure 5) and an MS/MS analysis (Figure S47) establishing the planar structure for acyl-surugamide A1 (**5**).

HRESI(+)MS analysis of **6** revealed a molecular formula (C_49_H_81_N_9_O_9_, Δmmu +2.6) consistent with an acetylated (+COCH_2_) analog of **3**. Comparison of the NMR (DMSO-*d*_6_) data for **6** (Table 3 and Table 4 and Table S7, Figure 5 and Figure S26–S31) with **3** supported this hypothesis, with the principal differences attributed to an *N*-acetyl-Lys^3^ moiety (*NH*-COCH_3_ *δ*_H_ 1.77; *δ*_C_ 22.6, 169.0), and diagnostic HMBC correlations (Figure 5) and an MS/MS analysis (Figure S47) establishing the planar structure for acyl-surugamide A2 (**6**). During the course of preparing this manuscript, **6** was reported from the marine *Streptomyces albidoflavus* RKJM-0023, and coincidentally assigned the same trivial nomenclature [12]. 

HRESI(+)MS analysis of **7** revealed a molecular formula (C_51_H_85_N_9_O_9_, Δmmu +2.4), consistent with a homolog (+CH_2_) of **6**. As only very small quantities of **7** could be isolated from CMB-M0112, and speculating on the likely structure, a sample of surugamide A (**3**) was treated with propionyl chloride and triethylamine to yield an *N*-propionyl-l-Lys^6^ derivative that exhibited the same UPLC retention time and ^1^H NMR (DMSO-*d*_6_) spectrum as **7**. Comparison of the full NMR (DMSO-*d*_6_) data for **7** (Table 3 and Table 4 and Table S8, Figure 5 and Figure S33–S38) with **6** revealed a high level of similarity, with the principal difference being replacement of the *N*-acetyl in **6** with an *N-*propionyl moiety in **7** (*δ*_H_ 2.04, q (*J* = 7.6 Hz), 0.97, t (*J* = 7.6 Hz); *δ*_C_ 172.8, 28.5, 9.96). Diagnostic HMBC correlations (Figure 5) and an MS/MS analysis (Figure S47) established the planar structure for acyl-surugamide A3 (**7**)**.**

HRESI(+)MS analysis of **8** returned a molecular formula (C_51_H_83_N_9_O_10_, Δmmu +2.4) consistent with an oxidized (+O, –H_2_) analog of **7**. Although only isolated as a minor co-metabolite, comparison of the NMR (DMSO-*d*_6_) data for **8** (Table 3 and Table 4 and Table S9, Figure 5 and Figure S40–S45) with **7** attributed the principal differences to replacement of the *N*-propionyl-l-Lys^3^ in **7** with an *N*-pyruvonyl-l-Lys^3^ moiety in **8**. While a lack of material precluded detection of the amide carbonyl NMR resonances, an HMBC correlation from the pyruvonoyl H_3_-3′ to a deshielded C-2′ ketone carbon (*δ*_C_ 197.3) and the presence of a deshielded l-Lys^3^-ε-*NH* (*δ*_H_ 8.51) provided supportive evidence for the *N*-pyruvonyl-l-Lys^3^ moiety. Furthermore, diagnostic HMBC correlations (Figure 5) and an MS/MS analysis (Figure S47) supported the planar structure for acyl-surugamide A4 (**8**).

The absolute configurations of **5**–**8** were confirmed by Marfey’s analyses (Figure S9). 

**Table 2:** ^1^H NMR (DMSO-*d*_6_) data for surugamides A (**3**), K (**4**), and acyl-surugamide A1 (**5**).

Amino Acid	Position	(3) *δ*_H_, Mult (*J* in Hz)	(4) *δ*_H_, Mult (*J* in Hz)	(5) *δ*_H_, Mult (*J* in Hz)
l-Ile^1^	2	4.18, m	4.16, m	4.16, dd (8.0, 8.0)
	3	1.73, m	1.74, m	1.75, m
	4_a_	1.46, m	1.46, m	1.46, m
	4_b_	1.13, m	1.13, m	1.11, m
	5	0.82 ^A^	0.81 ^A^	0.81 ^A^
	6	0.80 ^B^	0.80 ^A^	0.80 ^A^
	NH	8.27, d (7.4)	8.03, d (7.5)	8.29, d (8.0)
d-*allo*-Ile^2^	2	4.16, m	4.16, m	4.18, m
	3	1.83, m	1.83, m	1.81, m
	4_a_	1.34, m	1.34, m	1.33, m
	4_b_	1.21, m	1.21, m	1.20, m
	5	0.82 ^A^	0.82 ^B^	0.82 ^A^
	6	0.82 ^A^	0.82 ^B^	0.81 ^A^
	NH	8.04, d (7.2)	8.34, d (7.5)	7.95 ^B^
l-Lys^3^/*N-*acyl-l-Lys^3^	2	4.31, m	4.31, m	4.28, m
	3_a_	1.54 m	1.53, m	1.54, m
	3_b_	1.41, m	1.23, m	1.42, m
	4_a_	1.25, m	1.17, m	1.21, m
	4_b_	1.16, m	1.13, m	1.14, m
	5_a_	1.42, m	1.43, m	1.30, m
	5_b_	1.35, m	1.38, m	1.17, m
	6_a_	2.71, m, 2H	2.78, m, 2H	3.03, ddd (13.3, 6.6, 6.6)
	6_b_	-	-	2.97, ddd (13.0, 6.6, 6.6)
	6-NH/NH_2_	7.62, br s, 2H	8.41, br s	7.93 ^B^
	6-NHMe	-	2.54, s	-
	NH	7.52, br s	7.51, br s	7.61, br s
	1′	-	-	7.97, s
l-Ile^4^	2	3.87, dd (6.4, 6.4)	3.85, dd (6.6, 6.6)	3.85, dd (6.4, 6.4)
	3	1.45, m	1.45, m	1.43, m
	4_a_	1.18, m	1.19, m	1.18, m
	4_b_	0.81 ^B^	0.82 ^B^	0.81 ^A^
	5	0.68, dd (7.2, 7.2)	0.68, t (7.2)	0.68, dd (7.2, 7.2)
	6	0.44, d (6.9)	0.43, d (6.8)	0.44, d (6.6)
	NH	7.85, br s	7.86, br s	7.81 ^C^
d-Phe^5^	2	4.37, ddd (11.5, 8.2, 3.2)	4.37, ddd (11.5, 8.2, 3.2)	4.38, ddd (11.7, 8.2, 3.4)
	3_a_	3.25, m	3.24, dd (13.8, 3.2)	3.25, dd (14.3, 3.4)
	3_b_	2.69, dd (13.7, 11.5)	2.69, dd (13.8, 11.5)	2.68, dd (14.3, 11.7)
	5	7.23 ^C^	7.23, m	7.22, m
	6	7.23 ^C^	7.24, m	7.24, m
	7	7.17, t (6.7)	7.17, m	7.17, m
	NH	8.46, d (8.2)	8.49, d (8.3)	8.43, d (8.3)
d-Leu^6^	2	4.23, m	4.22, m	4.25, m
	3_a_	1.86, m	1.87, m	1.86, m
	3_b_	1.48, m	1.47, m	1.46, m
	4	1.68, m	1.67, m	1.67, m
	5	0.86, d (6.7)	0.86, d (6.6)	0.86, d (6.6)
	6	0.94, d (6.9)	0.93, d (6.6)	0.93, d (6.6)
	NH	7.70, d (7.4)	7.71, d (7.5)	7.73, d (8.3)
l-Ile^7^	2	4.07, dd (7.2, 7.2)	4.06, dd (8.5, 8.5)	4.08, dd (7.2, 7.2)
	3	1.79, m	1.79, m	1.77, m
	4_a_	1.26, m	1.25, m	1.26, m
	4_b_	1.13, m	1.13, m	1.11, m
	5	0.80 ^B^	0.79, dd (7.6, 7.6)	0.79
	6	0.80 ^B^	0.80 ^A^	0.80 ^A^
	NH	7.14, br s	7.11, m	7.12, d (7.2)
d-Ala^8^	2	4.27, m	4.27, m	4.22, dd (6.6, 6.6)
	3	1.22, d (6.6)	1.21, d (7.0)	1.21, d (6.6)
	NH	7.78, d (6.0)	7.77, d (6.6)	7.81 ^C^

^A–C^ Resonances with the same superscript within a column are overlapping, and assignments can be interchanged.

**Table 3:** ^13^C NMR (DMSO-*d*_6_) data for surugamides A (**3**) and K (**4**), and acyl-surugamides A1–A4 (**5**–**8**).

Amino Acid	Position	3	4	5	6	7	8
		*δ*_C_, Type	*δ*_C_, Type	*δ*_C_, Type	*δ*_C_, Type	*δ*_C_, Type	*δ*_C_, Type
l-Ile^1^	1	172.4 ^A^, C	172.4 ^A^, C	172.1, C	172.1, C	172.1, C	nd
	2	57.5, CH	57.7, CH	57.6, CH	57.6, CH	57.6, CH	57.4, CH
	3	35.3, CH	35.2, CH	35.2, CH	35.2, CH	35.2, CH	35.6, CH
	4	24.4, CH_2_	24.5 ^B^, CH_2_	24.4, CH_2_	24.4, CH_2_	24.4, CH_2_	24.4, CH_2_
	5	10.6, CH_3_	10.6, CH_3_	10.6, CH_3_	10.6, CH_3_	10.6, CH_3_	10.6, CH_3_
	6	15.0, CH_3_	15.1, CH_3_	15.1, CH_3_	15.07, CH_3_	15.1 ^A^, CH_3_	15.1 ^A^, CH_3_
d-*allo-*Ile^2^	1	170.9 ^B^, C	171.0 ^B^, C	170.9, C	170.9 ^A^, C	171.0 ^B^, C	nd
	2	56.7, CH	56.7, CH	56.6, CH	56.5, CH	56.6, CH	56.4, CH
	3	36.2, CH	36.2, CH	36.3, CH	36.3, CH	36.3, CH	36.5, CH
	4	25.7, CH_2_	25.7, CH_2_	25.7, CH_2_	25.7, CH_2_	25.7, CH_2_	25.7, CH_2_
	5	11.5, CH_3_	11.5, CH_3_	11.4, CH_3_	11.4, CH_3_	11.4, CH_3_	11.4, CH_3_
	6	14.4, CH_3_	14.5, CH_3_	14.4, CH_3_	14.4, CH_3_	14.4, CH_3_	14.4, CH_3_
l-Lys^3^/*N-*acyl-l-Lys^3^	1	172.4 ^A^, C	172.4 ^A^, C	nd	172.5, C	172.6, C	nd
	2	51.7, CH	51.7, CH	52.1, CH	52.0, CH	52.1, CH	52.0, CH
	3	31.3, CH_2_	31.3, CH_2_	31.6, CH_2_	31.6, CH_2_	31.6, CH_2_	31.5, CH_2_
	4	21.8, CH_2_	22.0, CH_2_	22.5, CH_2_	22.5, CH_2_	22.6, CH_2_	22.5, CH_2_
	5	26.1, CH_2_	24.5 ^B^, CH_2_	28.2, CH_2_	28.3, CH_2_	28.4, CH_2_	28.3, CH_2_
	6	38.9, CH_2_	48.2, CH_2_	37.2, CH_2_	38.6, CH_2_	38.5, CH_2_	38.6, CH_2_
	NHMe	-	32.5, CH_3_	-	-	-	-
	1′	-	-	161.0, CH	169.0, C	172.8, C	nd
	2′	-	-	-	22.6, CH_3_	28.5, CH_2_	197.3, C
	3′	-	-	-	-	9.96, CH_3_	24.9, CH_3_
l-Ile^4^	1	171.2, C	171.1 ^C^, C	171.2, C	171.1, C	171.1, C	nd
	2	57.8, CH	57.9, CH	57.9, CH	57.9, CH	57.9, CH	57.7, CH
	3	35.7 ^C^, CH	35.7 ^D^, CH	35.7, CH	35.7, CH	35.7, CH	35.8, CH
	4	24.7, CH_2_	24.8, CH_2_	24.7, CH_2_	24.7, CH_2_	24.7, CH_2_	24.6, CH_2_
	5	11.0, CH_3_	11.1, CH_3_	11.1, CH_3_	11.1, CH_3_	11.0, CH_3_	11.0, CH_3_
	6	14.7, CH_3_	14.8, CH_3_	14.8, CH_3_	14.7, CH_3_	14.8, CH_3_	14.8, CH_3_
d-Phe^5^	1	171.0 ^B^, C	171.1 ^C^, C	171.0, C	170.9 ^A^, C	171.0 ^B^, C	nd
	2	54.7, CH	54.8, CH	54.6, CH	54.6, CH	54.5, CH	54.5, CH
	3	36.5, CH_2_	36.5, CH_2_	36.5, CH_2_	36.5, CH_2_	36.5, CH_2_	36.5, CH_2_
	4	138.0, C	138.1, C	138.1, C	138.0, C	138.0, C	138.0, C
	5	129.0, CH	129.1, CH	129.0, CH	129.0, CH	129.0, CH	129.0, CH
	6	128.1, CH	128.2, CH	128.1, CH	128.1, CH	128.1, CH	128.1, CH
	7	126.3, CH	126.3, CH	126.3, CH	126.3, CH	126.3, CH	126.2, CH
d-Leu^6^	1	172.6, C	172.7, C	172.6, C	172.6, C	172.7, C	nd
	2	52.3, CH	52.3, CH	52.3, CH	52.2, CH	52.3, CH	52.2, CH
	3	40.2, CH_2_	40.2, CH_2_	40.2, CH_2_	40.2, CH_2_	40.2, CH_2_	40.3, CH_2_
	4	24.3, CH	24.3, CH	24.3, CH	24.3, CH	24.3, CH	24.3, CH
	5	21.5, CH_3_	21.5, CH_3_	21.5, CH_3_	21.4, CH_3_	21.4, CH_3_	21.5, CH_3_
	6	23.2, CH_3_	23.2, CH_3_	23.1, CH_3_	23.1, CH_3_	23.1, CH_3_	23.1, CH_3_
l-Ile^7^	1	169.8, C	169.8, C	169.9, C	169.9, C	170.0, C	169.9, C
	2	57.4, CH	57.5, CH	57.4, CH	57.3, CH	57.3, CH	57.2, CH
	3	35.7 ^C^, CH	35.7 ^D^, CH	35.8, CH	35.8, CH	35.9, CH	36.0, CH
	4	23.9, CH_2_	24.0, CH_2_	24.0, CH_2_	24.0, CH_2_	24.0, CH_2_	24.1, CH_2_
	5	11.3, CH_3_	11.3, CH_3_	11.2, CH_3_	11.2, CH_3_	11.3, CH_3_	11.2, CH_3_
	6	15.2, CH_3_	15.2, CH_3_	15.1, CH_3_	15.12, CH_3_	15.1 ^A^, CH_3_	15.1 ^A^, CH_3_
d-Ala^8^	1	172.4 ^A^, C	172.4 ^A^, C	172.4, C	172.4, C	172.5, C	172.4, C
	2	47.9, CH	48.1, CH	48.0, CH	48.1, CH	48.1, CH	48.0, CH
	3	19.0, CH_3_	19.1, CH_3_	18.8, CH_3_	18.8, CH_3_	18.8, CH_3_	18.8, CH_3_

^A–D^ Assignments with the same superscript within a column are interchangeable. nd = not detected.

**Table 4:** ^1^H NMR (DMSO-*d*_6_) data for acyl-surugamides A2–A4 (**6–8**).

Amino Acid	Position	(6) *δ*_H_, Mult (*J* in Hz)	(7) *δ*_H_, Mult (*J* in Hz)	(8) *δ*_H_, Mult (*J* in Hz)
l-Ile^1^	2	4.16, dd (8.0, 8.0)	4.16, dd (8.2, 7.8)	4.21, dd (8.3, 8.3)
	3	1.75, m	1.75, m	1.75, m
	4_a_	1.46, m	1.46, m	1.45, m
	4_b_	1.13, m	1.13, m	1.10, m
	5	0.81 ^A^	0.81 ^A^	0.81 ^A^
	6	0.80 ^A^	0.81 ^A^	0.79 ^B^
	NH	8.30, d (7.4)	8.30, d (7.3)	8.24, d (8.3)
d-*allo-*Ile^2^	2	4.19, dd (7.6, 4.5)	4.19, dd (7.6, 4.5)	4.21, dd (7.6, 4.5)
	3	1.82, m	1.81, m	1.80, m
	4_a_	1.34, m	1.34, m	1.33, m
	4_b_	1.20, m	1.20, m	1.19, m
	5	0.82 ^A^	0.83 ^A^	0.82 ^A^
	6	0.81 ^A^	0.80 ^B^	0.80 ^B^
	NH	7.95, d (7.6)	7.94, d (7.6)	7.95, d (7.6)
*N-*acyl-l-Lys^3^	2	4.28, m	4.27, m	4.29, m
	3_a_	1.54, m	1.55, m	1.55, m
	3_b_	1.41, m	1.41, m	1.42, m
	4_a_	1.19, m	1.13, m, 2H	1.17, m
	4_b_	1.12, m		1.12, m
	5_a_	1.28, m	1.28, m	1.37, m
	5_b_	1.21, m	1.23, m	1.30, m
	6_a_	3.01, ddd (13.3, 6.6, 6.6)	3.03, m	3.06, m
	6_b_	2.87, ddd (13.0, 6.6, 6.6)	2.87, m	3.00, m
	6-NH	7.75, dd (6.0, 6.0)	7.69, dd (5.5, 5.5)	8.51, dd (6.0, 6.0)
	NH	7.60, br s	7.60, br s	7.69, br s
	2′	1.77, s	2.04, q (7.6)	-
	3′	-	0.97, t (7.6)	2.32, s
l-Ile^4^	2	3.85, dd (6.4, 6.4)	3.84, dd (6.5, 6.3)	3.88, dd (6.7, 6.7)
	3	1.43, m	1.44, m	1.44, m
	4_a_	1.18, m	1.20, m	1.10, m
	4_b_	0.81 ^A^	0.81 ^A^	0.80 ^B^
	5	0.68, dd (7.3, 7.3)	0.68, dd (7.3, 7.3)	0.79, dd (7.4, 7.4)
	6	0.44, d (6.7)	0.43, d (6.5)	0.44, d (6.8)
	NH	7.81 ^B^	7.82 ^C^	7.71 ^C^
d-Phe^5^	2	4.38, ddd (11.7, 8.2, 3.4)	4.38, ddd (11.7, 8.3, 3.4)	4.39, ddd (11.6, 8.3, 3.3)
	3_a_	3.24, dd (14.3, 3.4)	3.25, dd (14.0, 3.4)	3.24, m
	3_b_	2.68, dd (14.3, 11.7)	2.67, dd (14.0, 11.7)	2.68, dd (13.6, 11.6)
	5	7.23, m	7.22, m	7.22, m
	6	7.24, m	7.24, m	7.24, m
	7	7.18, m	7.17, m	7.17, t (7.2)
	NH	8.43, d (8.3)	8.43, d (8.3)	8.41, d (8.4)
d-Leu^6^	2	4.25, m	4.38, ddd (11.7, 8.3, 3.4)	4.28, m
	3_a_	1.86, m	3.25, dd (14.0, 3.4)	a. 1.81, m
	3_b_	1.47, m	2.67, dd (14.0, 11.7)	b. 1.47, m
	4	1.68, m	7.22, m	1.66, m
	5	0.86, d (6.5)	7.24, m	0.86, d (6.5)
	6	0.93, d (6.5)	7.17, m	0.92, d (6.5)
	NH	7.73, d (7.4)	8.43, d (8.3)	7.72 ^c^
l-Ile^7^	2	4.08, dd (7.2, 7.2)	4.08, dd (7.2, 7.2)	4.09, dd (7.6, 7.6)
	3	1.77, m	1.77, m	1.76, m
	4_a_	1.27, m	1.26, m	1.28, m
	4_b_	1.11, m	1.11, m	1.11, m
	5	0.79, t (7.5)	0.78	0.79 ^B^
	6	0.81 ^A^	0.80 ^B^	0.80 ^B^
	NH	7.12, d (7.2)	7.22, br s	7.26
d-Ala^8^	2	4.23, dd (6.6, 6.6)	4.22, dd (7.0, 6.7)	4.26, dd (6.7, 6.7)
	3	1.21, d (6.6)	1.20, d (6.8)	1.20, d (6.7)
	NH	7.81 ^B^	7.83 ^C^	7.86, d (6.7)

^A–C^ Resonances with the same superscript within a column are overlapping, and assignments can be interchanged.

### 2.3. Synthetic Surugamide Analogs

**Figure 6:**
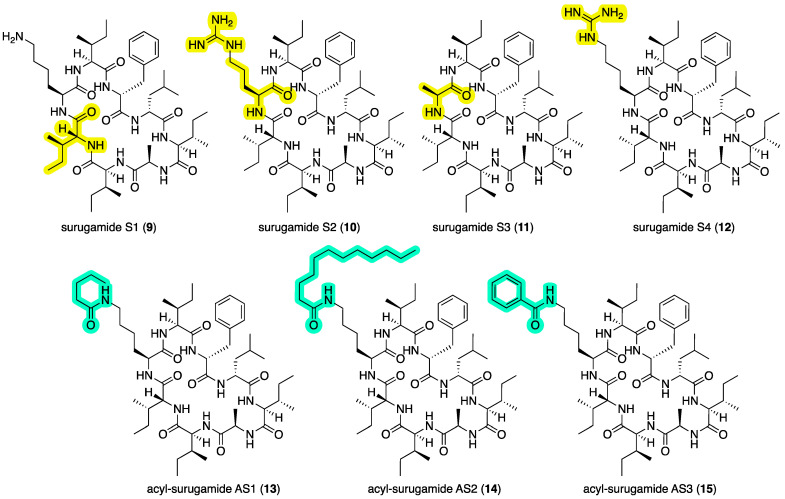
Synthetic surugamides S1–S4 (**9**–**12**) and acyl-surugamides AS1–AS3 (**13**–**15**). Highlights: variation in the amino acids (yellow) and acylation of Lys^3^ (green), relative to surugamide A (**3**).

In addition to the natural surugamides **3**–**4** and acyl-surugamides **5**–**8** recovered from CMB-MRB032 and CMB-M0112, to support structure elucidation studies we synthesized **3** and its isomer **9**, and to further support structure–activity relationship investigations, synthetic efforts were extended to surugamides S1–S4 (**9**–**12**) and acyl-surugamides AS1–AS3 (**13**–**15**) (Figure 6), as outlined below.

#### 2.3.1. Synthesis of Surugamides A (**3**) and S1–S3 (**9**–**11**) 

Solid-phase peptide synthesis was used to prepare surugamide A (**3**) as well as the new synthetic surugamides S1–S3 (**9**–**11**), where the latter are d-*allo*-Ile^2^ to d-Ile^2^, l-Lys^3^ to l-Arg^3^, and l-Lys^3^ to l-Ala^3^ substitution analogs of **3**, respectively. Structures for **3** and **9**–**11** were confirmed by spectroscopic analysis (Figures S53–S65).

#### 2.3.2. Synthesis of Surugamide S4 (**12**) and Acyl-Surugamides AS1–AS3 (**13**–**15**) 

Using standard derivatization protocols, authentic samples of **3** were transformed to the guanidino analog surugamide S4 (**12**), as well as the *N*-pentanoyl-l-Lys^3^, *N*-dodecanoyl-l-Lys^3^, and *N*-benzoyl-l-Lys^3^ amides, and acyl-surugamides AS1 (**13**), AS2 (**14**), and AS3 (**15**), respectively. Structures for all **12**–**15** were confirmed by spectroscopic analysis (Figures S66–S77).

### 2.4. Surugamide Structure–Activity Relationships

The antimycins A4a (**1**) and A2a (**2**) exhibited potent inhibition of motility of *D. immitis* mf (EC_50_ 0.0013 and 0.0021 μg/mL) and to a lesser degree *H. contortus* L1–L3 larvae development (EC_50_ 3.5 and 1.8 μg/mL), but were highly cytotoxic to human colon carcinoma cells (IC_50_ 0.061 and 0.080 μg/mL) (Table 5). Based on this, **1** and **2** are almost certainly largely responsible for the anthelmintic activity detected in the extract of *Streptomyces* sp. CMB-MRB032. Biological screening of the natural, semi-synthetic, and synthetic surugamides **3**–**15** recovered in this study supported the hypothesis that surugamides can also exhibit anthelmintic activity. Indeed, the natural product acyl-surugamide A3 (**7**) proved to be selectively active against *D. immitis* mf (EC_50_ 3.3 μg/mL) as did the acyl-surugamide AS3 (**15**) (EC_50_ 3.4 μg/mL), with neither exhibiting activity against either *H. contortus* L1–L3 larvae (EC_50_ > 25 μg/mL) or SW620 human colon carcinoma cells (IC_50_ > 30 μg/mL). This surugamide structure–activity relationship (SAR) indicates that not only do changes to the cyclic octapeptide sequence relative to **3** ablate activity against *D. immitis*, but that there is a size requirement for acylation of l-Lys^3^, where acetyl (e.g., **6**) is too small, pentanoyl (e.g., **13**) too large, with both propanoyl (e.g., **7**) and benzoyl (e.g., **15**) being acceptable. 

All natural and synthetic surugamides were also tested and found to be inactive (IC_50_ > 30 μM) against Gram-positive bacterium *Staphylococcus aureus* ATCC 25923 and fungus *Candida albicans* ATCC 10231 (Figure S83). The selective nature of the surugamide SAR against *D. immitis* supports the proposition that this class of natural products is not mere cell wall disrupters or cytotoxins, but likely elicit a motility inhibitory effect on *D. immitis* mf by binding with and modifying the function of a specific molecular target(s)/pathway.

**Table 5:** Effect of **1–15** on inhibition of motility of *D. immitis* mf and *H. contortus* L3 larvae.

Compound	*D. immitis* mf Motility EC_50_ (μg/mL)	*H. contortus* L1–L3 Larvae Development EC_50_ (μg/mL)	Human Colorectal Carcinoma Cells (SW620) IC_50_ (μg/mL)
antimycin A4a (**1**)	0.0013	3.5	0.061
antimycin A2a (**2**)	0.0021	1.8	0.080
surugamide A (**3**)	>25	>25	>30
surugamide K (**4**)	>25	>25	>30
acyl-surugamide A1 (**5**)	>25	>25	>30
acyl-surugamide A2 (**6**)	>25	>25	>30
acyl-surugamide A3 (**7**)	3.3	>25	nd
acyl-surugamide A4 (**8**)	16.0	>25	>30
surugamide S1 (**9**)	>25	>25	nd
surugamide S2 (**10**)	> 25	>25	nd
surugamide S3 (**11**)	> 25	>25	nd
surugamide S4 (**12**)	>25	>25	nd
acyl-surugamide AS1 (**13**)	21	>25	>30
acyl-surugamide AS2 (**14**)	>25	>25	>30
acyl-surugamide AS3 (**15**)	3.4	>25	>30

nd = not determined. Highlights: beige: EC_50_ < 5 μg/mL in all three assays; green: EC_50_ < 5 μg/mL against *D. immitis*.

### 2.5. Other Reported Surugamides and Their Activities

**Figure 7:**
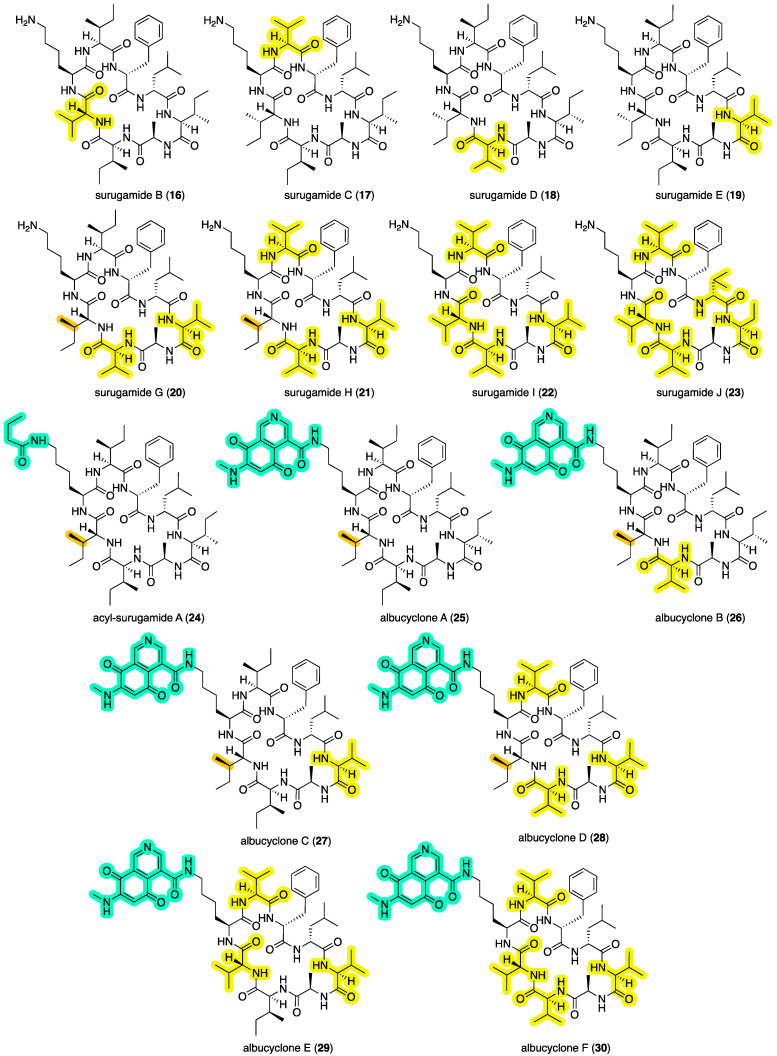
Known surugamides **16**–**23**, acyl-surugamide A (**24**) and albucyclones **25**–**30**. Highlights: variation in the amino acids (yellow) and acylation of Lys^3^ (green), relative to surugamide A (**3**), and likely stereochemical misassignments (brown).

Putting our contribution in context, surugamides A (**3**) and B–E (**16**–**19**) (Figure 7) were first reported in 2013 [7] from a deep-sea sediment-derived *Streptomyces* sp. JAMM992 collected by a remote unmanned vehicle at –106 m in Kinko Bay, Japan, at which time they were reported to be inhibitors of bovine cathepsin B, a cysteine protease implicated in invasion of metastatic tumor cells—although potency values were modest to negligible (IC_50_ 16–36 μM). In 2019 [8], the structure of surugamide A (and by inference surugamide C–E) was revised by total synthesis, replacing the d-Ile^2^ with a d-*allo*-Ile^2^ residue. Surugamide A (**3**) was subsequently reisolated in 2014 [13] from *Streptomyces* sp. RM-27-46 recovered from the soil of a coal mine fire site and was reported to exhibit moderate antibacterial activity against *Staphylococcus aureus* (MIC 10 μM). A 2016 [14] account attributed the trivial name surugamide F to a structurally unrelated acyclic decapeptide, while a 2017 [15] report on elicitor screens on *Streptomyces albus* J1074 described the isolation and structure elucidation of surugamides G–J (**20**–**23**) and acyl-surugamide A (**24**), as well as the biosynthetically related albucyclones A–F (**25**–**30**) (Figure 7). As a note of caution, this latter study asserted amino acid stereochemical assignments were identical to those (incorrectly) assigned to surugamides A–E in 2013 [7]. As such, it seems likely that **20**–**22** and **24**–**28** are incorrectly attributed to a d-Ile^2^ rather than d-*allo*-Ile^2^ residue (see 2019 [8] reassignment of **3**). Most recently, a 2024 [12] report described the new analog acyl-surugamide A2 (**6**) from a Mediterranean Sea marine tunicate-derived *Streptomyces albidoflavus* RKJM-0023. 

Interestingly, a 2013 [16] report on a cyclic octapeptide champacyclin isolated from a marine sediment-derived *Streptomyces champavatti* described it as having the same amino acid composition as surugamide A, but with a different sequence (*cyclo*[-d-*allo*Ile^1^-d-Ala^2^-l-Ile^3^-d-Leu^4^-d-Phe^5^-l-Ile^6^-l-Ile^7^-l-Lys^8^-]. Comparison of ^1^H NMR data (600 MHz, DMSO-*d*_6_) and MS/MS fragmentation patterns reported for champacyclin [16], with those obtained for surugamide A isolated from CMB-MRB032 (Figures S84–S86), revealed that champacyclin is identical to surugamide A, and hence its assignment should be revised accordingly. Champacyclin (surugamide A) was reported to inhibit the fire blight pathogen *Erwinia amylovora* at 25 μM (40% inhibition), although this low level of potency might be better characterized as inactive [16].

## 3. Materials and Methods

### 3.1. General Experimental Procedures

Chiroptical measurements ([α]_D_) were obtained on a JASCO P-1010 polarimeter in a 100 × 2 mm cell at specified temperatures. Nuclear magnetic resonance (NMR) spectra were acquired on a Bruker Avance 600 MHz spectrometer with a 5 mm PASEL ^1^H/D-^13^C Z-Gradient probe. In all cases, spectra were acquired at 25 °C in DMSO-*d*_6_ or CDCl_3_ with referencing to residual ^1^H or ^13^C signals (DMSO-*d*_6_, δ_H_ 2.50 and δ_C_ 39.5; CDCl_3_, δ_H_ 7.16 and δ_C_ 77.14). High-resolution ESIMS spectra were obtained on a Bruker micrOTOF mass spectrometer by direct injection in MeOH at 3 μL/min using sodium formate clusters as an internal calibrant. Liquid chromatography–diode array–mass spectrometry (HPLC-DAD-MS) data were acquired either on an Agilent 1260 series separation module equipped with an Agilent G6125B series LC/MSD mass detector and diode array detector or on Shimadzu LCMS-2020. Semi-preparative HPLCs were performed using Agilent 1100 series HPLC instruments with corresponding detectors, fraction collectors, and software inclusively. UPLC chromatograms were obtained on Agilent 1290 infinity UPLC system equipped with a diode array multiple wavelength detector (Zorbax C_8_ RRHD 1.8 μm, 50 × 2.1 mm column, eluting at 0.417 mL/min, 2.50 min gradient elution from 90% H_2_O/MeCN to 100% MeCN with a constant 0.01% TFA modifier). UPLC-QTOF analysis was performed on UPLC-QTOF instrument comprising an Agilent 1290 Infinity II UPLC (Zorbax C_8_ RRHD 1.8 μm, 50 × 2.1 mm column, eluting with 0.417 mL/min, 2.50 min gradient elution from 90% H_2_O/MeCN to 100% MeCN with a constant 0.1% formic acid modifier) coupled to an Agilent 6545 QTOF. MS/MS analysis was performed on the same instrument for ions detected in the full scan at an intensity above 1000 counts at 10 scans/s, with an isolation width of 4 ~m/z using a fixed collision energy and a maximum of 3 selected precursors per cycle. Chemicals were purchased from Sigma-Aldrich or Merck unless otherwise specified. Analytical-grade solvents were used for solvent extractions. Chromatography solvents were of HPLC grade supplied by Labscan or Sigma-Aldrich and filtered/degassed through 0.45 μm polytetrafluoroethylene (PTFE) membrane prior to use. Deuterated solvents were purchased from Cambridge Isotopes. Microorganisms were manipulated under sterile conditions using a Laftech class II biological safety cabinet and incubated in either MMM Friocell incubators (Lomb Scientific) or an Innova 42R incubator shaker (John Morris).

### 3.2. Cultivation and Fractionation of CMB-MRB032

Medium-scale cultivation of *Streptomyces* sp. CMB-MRB032 was carried out on ISP2 agar plates (×80), incubated at 30 °C for 14 d. Following incubation, the agar was harvested, diced (~1.5 cm^2^) and extracted with EtOAc (3 × 600 mL), and the combined organic phase concentrated in vacuo at 40 °C to yield an extract (590.8 mg), which was further partitioned between hexane (2 × 50 mL), and 90% aqueous MeOH (50 mL) and concentrated in vacuo at 40 °C to yield hexane (62.7 mg), and MeOH (173.1 mg) solubles. The MeOH solubles were fractionated by preparative HPLC chromatography (Phenomenex C_8,_ 10 µm, 21.2 × 250 mm column, gradient elution at 20 mL/min over 30 min from 90% H_2_O/MeCN to 100% MeCN with constant 0.01% TFA/MeCN modifier) to yield 14 fractions, which were tested for anthelmintic activities. UPLC-QTOF and GNPS analysis of the active fractions 9–13 suggested the presence of surugamides and antimycins. The presence of antimycins in these fractions was confirmed by co-injections with authentic standards of antimycins A4a (**1**) and A2a (**2**). A subsequent large-scale cultivation was carried out to isolate and identify the surugamides present in the active fractions.

The scaled-up cultivation of *Streptomyces* sp. CMB-MRB032 was carried out on ISP2 agar plates (×400), incubated at 30 °C for 14 d. Following incubation the agar was harvested, diced (~1.5 cm^2^) and extracted with EtOAc (10 × 600 mL), and the combined organic phase concentrated in vacuo at 40 °C to yield an extract (890.2 mg), which was further partitioned between hexane (3 × 50 mL), DCM (3 × 50 mL) and 90% aqueous MeOH (50 mL) and concentrated under N_2_ at 40 ^o^ C to yield hexane (188.4 mg), DCM (504.6 mg) and MeOH (197.2 mg) solubles. The DCM and MeOH solubles were combined and fractionated by Sephadex LH-20 (MeOs) resulting in nine fractions. Sephadex fraction F-3 (30.7 mg) was further subjected to preparative HPLC chromatography (Phenomenex C_8_, 10 µm, 21.2 × 250 mm column, gradient elution at 20 mL/min over 30 min from 90% H_2_O/MeCN to 100% MeCN with a constant 0.01% TFA/MeCN modifier) to give 12 fractions (F3-1 to F3-12). Fraction F3-5 was further purified through semi-preparative HPLC chromatography (Agilent Zorbax, SB-C_8_, 5 µm, 250 × 9.4 mm column, isocratic elution at 3 mL/min over 35 min with 53% H_2_O/MeCN) to yield surugamide A (**3**) (1.8 mg, 0.20%) and surugamide K (**4**) (0.5 mg, 0.05%). (Note: % yields estimated on a mass-to-mass basis against the weight of the crude EtOAc extract partition).

*surugamide A* (**3**): White powder, [α]_D_ –0.6 (*c* 0.1, MeOH); 1D and 2D NMR (DMSO-*d*_6_), Table 2 and Table 3 and Table S4, Figures S10 and S11; HRESIMS *m*/*z* 912.1616 [M + H]^+^ (calcd for C_48_H_81_N_9_O_8_, 912.1610).

*surugamide K* (**4**): Colorless oil; [α]_D_ +5.0 (*c* 0.1, MeOH); 1D and 2D NMR (DMSO-*d*_6_), Table 2 and Table 3 and Table S5, Figures S12–S17; HRESIMS *m*/*z* 926.6467 [M + H]^+^ (calcd for C_49_H_83_N_9_O_8_, 926.6397).

### 3.3. Cultivation and Fractionation of CMB-M0112

A seed culture of *Streptomyces* sp. CMB-M0112 was prepared by inoculating a flask (250 mL) containing ISP2 broth medium (80 mL) with bacterial colonies followed by incubation at 30 °C for 5 days with shaking (190 rpm). Aliquots of the seed culture (100 μL) were streaked onto individual ISP2 agar plates (×380), which were incubated at 30 °C for 21 days, after which the agar was harvested, diced (~1.5 cm^2^), and extracted with EtOAc (500 mL), with the decanted organic layer concentrated in vacuo to yield an extract (610 mg), which was subjected to sequential trituration to afford (after drying under nitrogen at 40 °C) *n*-hexane (78.2 mg) and DCM/MeOH (499.2 mg) solubles. The DCM/MeOH solubles were subjected to Sephadex LH20 gel chromatography with gradient elution from 50% MeOH/DCM to 100% MeOH, yielding 50 fractions (F01–F50). F17–1F9 were combined (21.3 mg) and subjected to semi-preparative HPLC-DAD (Agilent Zorbax C_3,_ 5 μm, 250 × 9.4 mm column, 3 mL/min gradient elution over 15 min from 50% H_2_O/MeCN to 45% H_2_O /MeCN, with a constant 0.01% TFA/MeCN modifier) to yield acyl-surugamide A1 (**5**) (1.2 mg, 0.2%), acyl-surugamide A2 (**6**) (2.0 mg, 0.2%), acyl-surugamide A3 (**7**) (0.6 mg, 0.1%), and acyl-surugamide A4 (**8**) (0.8 mg, 0.1%). (Note: % yields estimated on a mass-to-mass basis against the weight of the crude EtOAc extract partition). 

*acyl-surugamide A1* (**5**): Colorless oil; [α]_D_ +5.0 (*c* 0.1, MeOH); 1D and 2D NMR (600 MHz, DMSO-*d*_6_), Table 2 and Table 3 and Table S6, Figures S19–S24; HRESIMS *m*/*z* 962.6123 [M + Na]^+^ (calcd for C_49_H_81_N_9_NaO_9_, 962.6049).

*acyl-surugamide A2* (**6**): Colorless oil; [α]_D_ −5.0 (*c* 0.2, MeOH); 1D and 2D NMR (600 MHz, DMSO-*d*_6_), Table 3 and Table 4 and Table S7, Figures S26–S31; HRESIMS *m*/*z* 976.6232 [M + Na]^+^ (calcd for C_50_H_83_N_9_NaO_9_, 976.6206).

*acyl-surugamide A3* (**7**): Colorless oil; 1D and 2D NMR (DMSO-*d*_6_), Table 3 and Table 4 and Table S8, Figures S33–S38; HRESIMS *m*/*z* 990.6355 [M + Na]^+^ (calcd for C_51_H_85_N_9_NaO_9_, 990.6362).

*acyl-surugamide A4* (**8**): Colorless oil; [α]_D_ −4.3 (*c* 0.05, MeOH); 1D and 2D NMR (DMSO-*d*_6_), Table 3 and Table 4 and Table S9, Figures S40–S45; HRESIMS *m*/*z* 1004.6174 [M + Na]^+^ (calcd for C_51_H_83_N_9_NaO_10_, 1004.6155).

### 3.4. Synthesis of Surugamides A (***3***) and S1–S3 (***9***–***11***)

Peptides **3** and **9**–**11** were synthesized by manual stepwise solid phase peptide synthesis (SPPS) on 2-chlorotrityl chloride (2-CTC) resin (substitution ratio: 1.55 mmol/g, 0.5 mmol scale, 32 mg) using HATU/DIPEA Fmoc-chemistry. Steps included: 

*(i) Coupling the first amino acid to the resin*: After swelling the 2-CTC resin for 20 min in dry DCM (2 mL) a solution of the first amino acid (1.2 eq.) and DIPEA (22 µL, 0.13 mmol, 2.5 eq.) in dry DCM (2 mL) was mixed with the resin for 2 h, after which the resin was filtered and MeOH (200 µL) added and mixed for 15 min to cap the resin. The capped resin was washed with dry DCM (5 × 1 min), 1:1 DCM: MeOH (5 × 1 min), and MeOH (2 × 1 min).

*(ii) Elongation of peptide sequence on resin*: Amino acid activation was achieved by dissolving the target Fmoc-protected amino acid (0.16 mmol, 3.2 eq.) in 0.4 M HATU/DMF solution (0.38 mL, 0.15 mmol, 3.0 eq.) followed by the addition of DIPEA (53 µL, 0.3 mmol, 6.0 eq.). The coupling cycle consisted of Fmoc deprotection with 20% of piperidine in DMF (twice, 5 and 10 min), a 5 min DMF flow-wash, followed by coupling with preactivated Fmoc-amino acids (3.2 eq., 2 × 30 min). Upon completion, the Fmoc was removed with 20% piperidine in DMF (twice, 5 and 10 min), and the resin was washed sequentially with DMF (5 × 1 min), DCM (5 × 1 min), and MeOH (1 min), before drying in a vacuum desiccator.

*(iii) Cleavage of linear protected peptide from resin*: After swelling for 20 min in dry DCM (2 mL), the resin was mixed with 20% HFIP/DCM (2 mL × 3 × 20 min), and the combined filtrate concentrated in vacuo to give the crude fully protected linear peptide.

*(iv) Cyclization of protected peptide:* Protected linear peptide 0.5 mg/mL in DMF was stirred vigorously and a mixture of 0.4 M HATU (3 eq.), HOBT (3 eq.) and collidine (3 eq.) in DMF (2 mL) added very slowly over 30 min. After 14 h, and/or after LC-MS analysis confirmed completion, the reaction mixture was concentrated in vacuo, and the residue was subjected to preparative HPLC (Phenomenex C_8_, 10 µm, 9.4 × 250 mm column, gradient elution at 20 mL/min over 20 min from 90% H_2_O/MeCN to 100% MeCN with a constant 0.01% TFA/MeCN modifier). After lyophilization, the protected cyclic peptide was obtained as an amorphous powder.

*(v) Deprotection of 2,2,4,6,7-pentamethyldihydrobenzofuran-5-sulfonyl (Pbf) from Arg:* The peptide was stirred for 3 h in a cleavage cocktail (5 mL) comprising trifluoroacetic acid (TFA), triisopropylsilane (TIS), and H_2_O (95:2.5:2.5) after which the reaction mixture was concentrated under a stream of nitrogen, and the residue washed with ice-cold ether (5 mL) prior to purification by HPLC. 

*(vi) Deprotection of tert-butyloxycarbonyl (Boc) from Lys*: The peptide was stirred for 3 h in a cleavage solution (1 mL) consisting of dry TFA and DCM (50:50) after which the reaction mixture was concentrated under a stream of nitrogen, and the residue taken up in MeCN/water (5 mL) and freeze-dried to give pure cyclic peptide. 

*surugamide A* (**3**): Colorless oil; NMR (DMSO-*d*_6_) Table 2 and Table 3 and Table S4, and Figures S53 and S54; HRESIMS *m*/*z* 912.6296 [M + H]^+^ (calcd for C_48_H_82_N_9_O_8_, 912.6281).

*surugamide S1* (**9**): Colorless oil; NMR (DMSO-*d*_6_) Figures S57 and S58; HRESIMS *m*/*z* 912.6282 [M + H]^+^ (calcd for C_48_H_82_N_9_O_8_, 912.6281).

*surugamide S2* (**10**): amorphous powder; NMR (DMSO-*d*_6_) Figures S60 and S61; HRESIMS *m*/*z* 940.6395 [M + H]^+^ (calcd for C_48_H_82_N_11_O_8_, 940.6342).

*surugamide S3* (**11**): amorphous powder; NMR (DMSO-*d*_6_) Figures S63 and S64; HRESIMS *m*/*z* 877.5572 [M + Na]^+^ (calcd for C_45_H_74_N_8_NaO_8_, 877.5522).

### 3.5. Synthesis of Surugamide S4 (***12***) and Acyl-Surugamides A3 (***7***) and AS1–AS3 (***13***–***15***) 

Each of the following derivatizations was carried out on a chromatography fraction obtained from the extract of CMB-MRB032, enriched in surugamide A (**3**).

*acyl-surugamide A3* (**7**): A solution of **3** (8 mg) in anhydrous THF (300 μL) with propionyl chloride (20 μL) and triethylamine (20 μL) was stirred at r.t overnight, after which the solvent was removed in vacuo and the residue subjected to semi-preparative HPLC (Zorbax SB-C_3_, 5 μm, 9.4 × 250 mm column, isocratic elution at # mL/min over 15 min with 45% H_2_O/MeCN with a constant 0.01% TFA/MeCN modifier) to yield acyl-surugamide A3 (**7**) (2.3 mg, # %) identical with **7** recovered from CMB-M0112. Colorless oil; [α]_D_ -113 (*c* 0.08, MeOH); NMR (DMSO-*d*_6_), Table 2 and Table 3 and Table S8, Figures S33–S38, HRESIMS *m*/*z* 968.6578 [M + H]^+^ (calcd for C_51_H_86_N_9_O_9_, 968.6543).

*surugamide S4* (**12**): A solution of **3** (5 mg) in anhydrous THF (300 μL) with 1H-pyrazole-1-carboximidamide (1 mg, 13 μM) and DIPEA (50 μL) was stirred at 40 °C for 6 h, after which the solvent was removed in vacuo and the residue subjected to semi-preparative HPLC (Zorbax-XDB C_8_ 5 μm, 9.4 × 250 mm column, gradient elution at 3 mL/min over 10 min from 50% H_2_O /MeCN to 45% H_2_O /MeCN with a constant 0.01% TFA/MeCN modifier) to yield surugamide S4 (**12**, 0.6 mg). Colorless oil; NMR (DMSO-*d*_6_) Figures S66 and S67; HRESIMS *m*/*z* 954.6532 [M + H]^+^ (calcd for C_49_H_84_N_11_O_8_, 954.6499).

*acyl-surugamide AS1* (**13**): A solution of **3** (5 mg) in anhydrous THF (300 μL) with valeric acid (5 μL), dicyclohexylcarbodiimide (DCC) (2 mg) and dimethylaminopyridine (DMAP) (2 mg) was stirred at r.t for 1 h, after which the solvent was removed in vacuo and the residue subjected to semi-preparative HPLC (Zorbax SB-C_3_, 5 μm, 9.4 × 250 mm column, gradient elution at 3 mL/min over 15 min from 45% H_2_O/MeCN to 40% H_2_O/MeCN with a constant 0.01% TFA/MeCN modifier) to acyl-surugamide AS1 (**13**) (1.3 mg). Colorless oil; NMR (DMSO-*d*_6_) Figures S69 and S70; HRESIMS *m*/*z* 996.6912 [M + H]^+^ (calcd for C_53_H_90_N_9_O_9_, 996.6856).

*acyl-surugamide AS2* (**14**): A solution of **3** (5 mg) in anhydrous THF (300 μL) with lauric acid (2 mg), DCC (2 mg), and DMAP (2 mg) was stirred at r.t overnight, after which the solvent was removed in vacuo and the residue subjected to semi-preparative HPLC (Zorbax SB-C_3_, 5 μm, 9.4 × 250 mm column, isocratic elution at 3 mL/mim over 15 min with 25% H_2_O/MeCN with a 0.01% TFA/MeCN modifier) to yield acyl-surugamide AS2 (**14**, 1.3 mg). Colorless oil; NMR (DMSO-*d*_6_) Figures S72 and S73; HRESIMS *m*/*z* 1094.8011 [M + H]^+^ (calcd for C_60_H_104_N_9_O_9_, 1094.7952).

*acyl-surugamide AS3* (**15**): A solution of **3** (5 mg) in anhydrous THF (300 μL) with benzoic acid (2 mg), DCC (2 mg) and DMAP (2 mg) was stirred at r.t overnight, after which the solvent was removed in vacuo and the residue subjected to semi-preparative HPLC (Zorbax SB-C_3_ 5 μm, 9.4 × 250 mm column, isocratic elution at 3 mL/min over 15 min with 35% H_2_O/MeCN with a 0.01% TFA/MeCN modifier) to yield acyl-surugamide AS43 (**15**, 0.5 mg). Colorless oil; NMR (DMSO-*d*_6_) Figures S75 and S76; HRESIMS *m*/*z* 1016.6600 [M + H]^+^ (calcd for C_55_H_86_N_9_O_9_, 1016.6543).

### 3.6. Antibacterial Assay

LB agar plates inoculated with *Staphylococcus aureus* ATCC 25923 were incubated at 37 °C for 24 h, after which several colonies were transferred to fresh sterile LB broth, which was incubated at 37 °C for 24 h, and following measurement of optical density, the cell density adjusted to 5 × 10^5^ CFU/mL. Analytes (**3**–**8**, **13**–**15**, and controls) were dissolved in DMSO and diluted with H_2_O to afford stock solutions (600 µM, 20% DMSO), which were serially diluted with 20% DMSO to yield analyte concentrations ranging from 600 to 0.2 µM. An aliquot (10 µL) of each analyte dilution was transferred to a 96-well microtiter plate along with freshly prepared bacterium broth (190 µL) to final concentrations of 30–0.01 µM in 1% DMSO. The resulting assay plates were incubated at 37 °C for 18 h and the optical density of each well was measured spectrophotometrically at 600 nm using POLARstar Omega plate reader (BMG LABTECH, Offenburg, Germany). The positive control was rifampicin and ampicillin (10 µM in 1% DMSO), and negative control was 1% DMSO in culture broth, together with extracts prepared from LB broth medium without bacterial inoculation. Each analysis was repeated two times and the data represented graphically, and IC_50_ and MIC values were calculated using GraphPad Prism version 10.0.1 (Figure S83).

### 3.7. Antifungal Assay

SD agar plates inoculated with *Candida albicans ATCC 10231* were incubated at 27 °C for 48 h, after which several colonies were transferred to fresh sterile SD broth (4 mL), which was incubated at 27 °C for 48 and, following measurement of optical density, the cell density adjusted to 5 × 10^5^ CFU/mL. An aliquot (10 µL) of analytes (**3**–**8**, **13**–**15**) prepared as above for antibacterial assays was transferred to a 96-well microtiter plate, and freshly prepared fungal broth (190 µL) was added to each well to give final concentrations of 30–0.01 µM in 1% DMSO. The resulting assay plates were incubated at 27 °C for 48 h and the optical density of each well was measured spectrophotometrically at 600 nm using POLARstar Omega plate reader (BMG LABTECH, Offenburg, Germany). The positive control was amphotericin (10 µM in 1% DMSO) and negative control was 1% DMSO, together with extracts prepared from SD broth without fungal inoculation. Each analysis was repeated two times and the data represented graphically, and IC_50_ and MIC values calculated using GraphPad Prism version 10.0.1 (Figure S83).

### 3.8. Cytotoxic Assay

Aliquots (3000 cells/well in 190 µL of Roswell Park Memorial Institute medium supplemented with 10% fetal bovine serum) of human colorectal (SW620) carcinoma cells were transferred to 96-well microtiter plates and incubated at 37 °C in 5% CO_2_ for 3 days. An aliquot (10 µL) of analytes (**3**–**8**, **13–15**), as prepared above for antibacterial assays, was transferred to a 96-well microtiter plate and incubated again for 24 h, after which an aliquot (10 µL) of a solution of 3-(4,5-dimethylthiazol-2-yl)-2,5-diphenyltetrazolium bromide (MTT) in phosphate-buffered saline (5 mg/mL) was added to each well, which were again incubated for 4 h. The media were then carefully removed (pipette) and the residue dissolved in DMSO (100 µL) by shaking at 50 rpm for 2 min. Finally, the absorbance of each well was measured spectrophotometrically at 600 nm using POLARstar Omega plate reader (BMG LABTECH, Offenburg, Germany). The positive control was sodium dodecyl sulfate (SDS) and negative control was 1% DMSO. Each analysis was repeated two times and the data represented graphically, and IC_50_ and MIC values were calculated using GraphPad Prism version 10.0.1 (Figure S83).

### 3.9. Anthelmintic Assays

*Inhibition of motility of D. immitis microfilariae.* Approximately two hundred and fifty *Dirofilaria immitis* microfilariae suspended in a total volume of 100 µL RPMI 1640 media (Hyclone) was added to wells of a microtiter plate containing various concentrations of test compounds formulated in 100% DMSO. Plates were incubated for ~72 h at 37 °C and 5% CO_2_. Parasite motility quantitative descriptors were calculated for each well after imaging in a camera-based system. The properties of each test compound at a given dose were expressed as percentage motility inhibition after normalization with the average motility of positive (1.0 µM Gramicidin) and negative (DMSO) controls on each plate. Dose–response assays were conducted to determine EC_50_ values.

*H. contortus L1-L3 larvae development assay (LDA).* Approximately 20 L1 stage *Haemonchus contortus* were delivered to wells of a microtiter plate containing nutrient medium and various concentrations of test compounds dissolved in 100% DMSO. Plates were incubated for four days at 27 °C and 85% relative humidity. The resulting worms (L3s) were imaged in a camera-based system and quantitative motility descriptors were calculated. The properties of each test compound at a given dose were expressed as percentage inhibition after normalization of the motility descriptor values with the average motility of positive (1.0 µM Ivermectin) and negative controls (DMSO only), respectively. Dose–response assays were conducted to determine an EC_50_ value. 

## 4. Conclusions

This study demonstrates the value of screening microbial extracts to detect natural products exhibiting anthelmintic properties, and the use of GNPS molecular networking as a means to rapidly dereplicate new from known, and rare from common, and detect new producers of prioritized microbial natural products. The study also discloses for the first time that members of the surugamide family of *Streptomyces* cyclic octapeptides are non-cytotoxic, selective inhibitors of the motility of *D. immitis* microfilaria. 

More specifically, a bioassay and chemistry-guided investigation of Australian marine and terrestrial *Streptomyces* spp. led to the isolation and identification of surugamide A (**3**), and the new natural analogs surugamide K (**4**) and acyl-surugamides A1–A4 (**5**–**8**). To address structure assignment/re-assignment issues, and explore the structure–activity relationships, we undertook the synthesis of **3** and the new synthetic surugamides S1–S4 (**9**–**12**) and semi-synthetic acyl-surugamides AS1–AS3 (**13**–**14**). A detailed spectroscopic analysis of **3**–**14** facilitated an independent confirmation of the structure for **3** and prompted a reexamination of the structures assigned to all other known members of the surugamide structure class (**16**–**30**). The latter highlighted inconsistencies in the reported structures for **20**–**21** and **24**–**28** (d-Ile should be replaced by *allo*-d-Ile) and confirmed that champacyclin is not isomeric with but is in fact identical to **3**. 

Furthermore, the detection of six taxonomically and chemically unique surugamide-producing Australian *Streptomyces* spp, from both marine and terrestrial sources, demonstrated that despite limited reports on the structural diversity of this compound class in the scientific literature, the biosynthetic genes needed to produce surugamides are relatively widespread. Of particular note was the co-occurrence with the surugamides of the highly cytotoxic and anthelmintic antimycins. This latter observation warrants comment as it highlights a limitation of GNPS molecular networking as a “first-line” tool to prioritize/deprioritize extracts—where the detection of a known nuisance class (e.g., antimycins) in an anthelmintic extract may be seen as sufficient to deprioritize and abandon further investigation. While such an approach is not without merit, on this occasion, it would have excluded investigation of the anthelmintic properties of the non-cytotoxic surugamides. As noted in this study, such exclusions can be avoided by reacquiring bioassay and GNPS analyses on fractionated extracts and/or extracts obtained from cultivation profiling on multiple media (MATRIX). 

Finally, a *D. immitis* mf SAR assessment of the natural, synthetic, and semi-synthetic surugamides assembled during this study highlights the importance of the core octapeptide sequence and selective acylation of the Lys^3^-ε-NH_2_ residue. That the acyl-surugamides A3 (**7**) and AS3 (**15**) were the sole surugamide anthelmintic actives, but were neither cytotoxic, antibacterial, or antifungal, suggests a molecular target-oriented mode-of-action (i.e., discrete binding interaction). Given the rise in anthelmintic resistance, future investigations into the mode of action of the acyl-surugamides would appear warranted, as such knowledge may reveal a promising new target that could inform the development of future anthelmintics.

## Data Availability

Raw NMR data for **3**–**8** have been deposited in the Natural Product Magnetic Resonance Database Project (NP-MRD).

## Supplementary Materials

The following supporting information can be downloaded at: https://www.mdpi.com/article/10.3390/md22070312/s1, taxonomic analysis of CMB-MRB032 and CMB-M0112, GNPS analyses; cultivation profiling (MATRIX); NMR spectroscopic data (**3**–**15**), Marfey’s analyses, and MS/MS spectra.
