# Supplementary material for: Australian Marine and Terrestrial Streptomyces-Derived Surugamides, and Synthetic Analogs, and Their Ability to Inhibit Dirofilaria immitis (Heartworm) Motility
Source: Mar Drugs. 2024 Jul 9;22(7):312. doi: 10.3390/md22070312 (PMC11277932; doi:10.3390/md22070312)
Supplement: Supplementary file 1 [file marinedrugs-22-00312-s001.zip › marinedrugs-3089348-supplementary.pdf]

# Supplementary Materials

# Australian marine and terrestrial *Streptomyces* derived surugamides, and synthetic analogues, and their ability to inhibit *Dirofilaria immitis* (heartworm) motility.

Taizong Wu[1,#], Waleed M. Hussein[1,#], Kaumadi Samarasekera[1], Yuxuan Zhu[1], Zeinab G. Khalil[1], Shengbin Jin[1],David Bruhn[2], Yovany Moreno[2], Angela A. Salim[1,*] and Robert J. Capon[1,*]

[1]Institute for Molecular Bioscience, The University of Queensland, Brisbane, QLD 4072, Australia
[2]Boehringer Ingelheim Animal Health, USA Inc. 1730 Olympic Drive, Athens GA, 30601, USA

* corresponding author: a.salim@uq.edu.au; r.capon@uq.edu.au
[#] both authors contributed equally

## Table of Contents

*1. Collection and Taxonomy of CMB-MRB032 and CMB-M0112* ................................................ *3*

*2. GNPS Molecular Networking of active fractions* ................................................ *5*

*3. GNPS Molecular Networking search for other strains producing surugamides* ................................................ *7*

*4. Media MATRIX and Time study* ................................................ *9*

*5. Marfey's analysis* ................................................ *11*

*6. Spectroscopic data for natural products 3-8* ................................................ *12*

*7. Spectroscopic data of synthetic products* ................................................ *52*

*8. Detection of antimycins in crude extracts* ................................................ *69*

*9. Bioassay results* ................................................ *73*

*10. Comparison of champacyclin and surugamide A* ................................................ *74*

## 1. Collection and Taxonomy of CMB-MRB032 and CMB-M0112

*Streptomyces* sp. CMB-MRB032 was isolated from a sheep faecal sample collected from a farm in Bathurst, VIC, Australia, in 2017. *Streptomyces* sp. CMB-M0112 was isolated from marine sediment collected in Shorncliff, Qld, Australia, in 2007.

Genomic DNA was extracted from the mycelia using the DNeasy Plant Mini Kit (Qiagen) as per the manufacturer's protocol. The 16S rRNA genes were amplified by PCR using the universal primers 27F (5'-AGAGTTTGATCCTGGCTCAG-3') and 1492R (5'- TACGGCTACCTTCTTACGACTT-3') purchased from Sigma-Aldrich. The PCR mixture (50 µL) contained genomic DNA (2 µL, 20–40 ng), EmeraldAmpn GT PCR Master Mix (2XPremix) (25 µL), primer (0.2 µM, each), and $H_2O$ (up to 50 µL). PCR was performed using the following conditions: initial denaturation at 95 °C for 2 min, 40 cycles in series of 95 °C for 20 s (denaturation), 56 °C for 20 s (annealing) and 72 °C for 30 s (extension), followed by one cycle at 72 °C for 5 min. The PCR products were purified with PCR purification kit (Qiagen) and sequenced. BLAST analysis (NCBI database) showed that the amplified 16S rRNA sequence of CMB-MRB032 (Accession number OR346138) and CMB-M0112 (Accession number OR346138) have 99.5% and 98.8% identity with *Streptomyces koyangensis* strain VK-A60T and *Streptomyces lividans* strain XQ46, respectively (Figures S1 and S2).

| Description | Scientific Name | Max Score | Total Score | Query Cover | E value | Per. Ident | Acc. Len | Accession |
|---|---|---|---|---|---|---|---|---|
| Streptomyces koyangensis strain VK-A60T chromosome, complete genome | Streptomyces koyangensis | 1367 | 9540 | 88% | 0.0 | 99.47% | 7220839 | CP031742.1 |
| Streptomyces koyangensis strain CAP12 16S ribosomal RNA gene, partial sequence | Streptomyces koyangensis | 1367 | 1367 | 88% | 0.0 | 99.47% | 1463 | OQ557958.1 |
| Streptomyces koyangensis strain CAP08 16S ribosomal RNA gene, partial sequence | Streptomyces koyangensis | 1367 | 1367 | 88% | 0.0 | 99.47% | 1460 | OQ557959.1 |
| Streptomyces koyangensis strain ST378 16S ribosomal RNA gene, partial sequence | Streptomyces koyangensis | 1367 | 1367 | 88% | 0.0 | 99.47% | 1349 | KP096287.1 |
| Streptomyces koyangensis strain ST370 16S ribosomal RNA gene, partial sequence | Streptomyces koyangensis | 1367 | 1367 | 88% | 0.0 | 99.47% | 1359 | KP096286.1 |
| Streptomyces koyangensis strain ST201 16S ribosomal RNA gene, partial sequence | Streptomyces koyangensis | 1367 | 1367 | 88% | 0.0 | 99.47% | 1353 | KP096278.1 |
| Streptomyces sp. strain EAG2 16S ribosomal RNA gene, partial sequence | Streptomyces sp. | 1367 | 1367 | 88% | 0.0 | 99.47% | 1517 | KY593924.1 |
| Streptomyces koyangensis strain SCSIO 5802 chromosome, complete genome | Streptomyces koyangensis | 1367 | 9529 | 88% | 0.0 | 99.47% | 6861301 | CP049945.1 |
| Actinomycetales bacterium AO1518 16S ribosomal RNA gene, partial sequence | Actinomycetales bacterium A... | 1367 | 1367 | 88% | 0.0 | 99.47% | 1406 | JQ924114.1 |
| Actinomycetales bacterium AB1084 16S ribosomal RNA gene, partial sequence | Actinomycetales bacterium A... | 1367 | 1367 | 88% | 0.0 | 99.47% | 1406 | JQ924097.1 |
| Streptomyces sp. strain MGB 2739 16S ribosomal RNA gene, partial sequence | Streptomyces sp. | 1367 | 1367 | 88% | 0.0 | 99.47% | 1439 | MN339811.1 |
| Streptomyces hydrogenans strain Kris3 16S ribosomal RNA gene, partial sequence | Streptomyces hydrogenans | 1365 | 1365 | 88% | 0.0 | 99.47% | 1239 | MT588802.1 |
| Streptomyces sp. strain HBUM206419 16S ribosomal RNA gene, partial sequence | Streptomyces sp. | 1365 | 1365 | 88% | 0.0 | 99.47% | 1437 | MT540570.1 |
| Streptomyces sp. strain HBUM206352 16S ribosomal RNA gene, partial sequence | Streptomyces sp. | 1365 | 1365 | 88% | 0.0 | 99.47% | 1437 | MT540264.1 |
| Streptomyces sp. strain HBUM206360 16S ribosomal RNA gene, partial sequence | Streptomyces sp. | 1365 | 1365 | 88% | 0.0 | 99.47% | 1417 | MT540253.1 |
| Streptomyces sp. strain P12-37 16S ribosomal RNA gene, partial sequence | Streptomyces sp. | 1365 | 1365 | 88% | 0.0 | 99.47% | 1354 | MT255053.1 |
| Streptomyces violascens strain NL57 16S ribosomal RNA gene, partial sequence | Streptomyces violascens | 1365 | 1365 | 88% | 0.0 | 99.47% | 1406 | MT214322.1 |
| Streptomyces sp. strain VSP3 16S ribosomal RNA gene, partial sequence | Streptomyces sp. | 1365 | 1365 | 88% | 0.0 | 99.47% | 1436 | MT176507.1 |
| Streptomyces sp. strain BSP1 16S ribosomal RNA gene, partial sequence | Streptomyces sp. | 1365 | 1365 | 88% | 0.0 | 99.47% | 1494 | MT176505.1 |
| Streptomyces daghestanicus strain AN1078 16S ribosomal RNA gene, partial sequence | Streptomyces daghestanicus | 1365 | 1365 | 88% | 0.0 | 99.47% | 1448 | OR527156.1 |
| Streptomyces sp. strain AJ-1 16S ribosomal RNA gene, partial sequence | Streptomyces sp. | 1365 | 1365 | 88% | 0.0 | 99.47% | 1524 | OR518414.1 |
| Streptomyces sp. strain TG1A-3 16S ribosomal RNA gene, partial sequence | Streptomyces sp. | 1365 | 1365 | 88% | 0.0 | 99.47% | 1338 | OR485890.1 |
| Streptomyces sp. strain TG1A-25 16S ribosomal RNA gene, partial sequence | Streptomyces sp. | 1365 | 1365 | 88% | 0.0 | 99.47% | 1338 | OR485888.1 |
| Streptomyces sp. strain S_04425 16S ribosomal RNA gene, partial sequence | Streptomyces sp. | 1365 | 1365 | 88% | 0.0 | 99.47% | 1388 | OR211586.1 |

**Figure S1**. Blast search for CMB-MRB032.

| | Description | Scientific Name | Max Score | Total Score | Query Cover | E value | Per. Ident | Acc. Len | Accession |
|---|---|---|---|---|---|---|---|---|---|
| ☑ | Streptomyces lividans strain XQ46 16S ribosomal RNA gene, partial sequence | Streptomyces lividans | 2106 | 2106 | 99% | 0.0 | 98.82% | 1439 | KU291362.1 |
| ☑ | Streptomyces sp. F-6 16S ribosomal RNA gene, partial sequence | Streptomyces sp. F-6 | 2106 | 2106 | 99% | 0.0 | 98.90% | 1367 | FJ405358.1 |
| ☑ | Streptomyces sp. 620F 16S ribosomal RNA gene, partial sequence | Streptomyces sp. 620F | 2104 | 2104 | 99% | 0.0 | 98.82% | 1406 | KP998448.1 |
| ☑ | Streptomyces sp. strain NX-R-69 16S ribosomal RNA gene, partial sequence | Streptomyces sp. | 2104 | 2104 | 99% | 0.0 | 98.82% | 1469 | OQ438230.1 |
| ☑ | Streptomyces sp. OUCMDZ3434 16S ribosomal RNA gene, partial sequence | Streptomyces sp. OUCMDZ-3434 | 2104 | 2104 | 99% | 0.0 | 98.82% | 1453 | KJ818249.1 |
| ☑ | Streptomyces sp. OA30 16S ribosomal RNA gene, partial sequence | Streptomyces sp. OA30 | 2104 | 2104 | 99% | 0.0 | 98.82% | 1419 | JN942117.1 |
| ☑ | Streptomyces sp. OA6 16S ribosomal RNA gene, partial sequence | Streptomyces sp. OA6 | 2104 | 2104 | 99% | 0.0 | 98.82% | 1421 | JN942112.1 |
| ☑ | Streptomyces sp. CP-3 16S ribosomal RNA gene, partial sequence | Streptomyces sp. CP-3 | 2104 | 2104 | 99% | 0.0 | 98.82% | 1400 | JN628972.1 |
| ☑ | Streptomyces sp. L131D 16S ribosomal RNA gene, partial sequence | Streptomyces sp. L131D | 2104 | 2104 | 99% | 0.0 | 98.82% | 1421 | HM797397.1 |
| ☑ | Streptomyces sp. A333 Ydz-QZ 16S ribosomal RNA gene, partial sequence | Streptomyces sp. A333 Ydz-QZ | 2104 | 2104 | 99% | 0.0 | 98.82% | 1441 | EU368815.1 |
| ☑ | Actinomycetales bacterium B3(2010) strain B3 16S ribosomal RNA gene, partial sequence | Actinomycetales bacterium B3(2… | 2104 | 2104 | 99% | 0.0 | 98.82% | 1431 | HM007152.1 |
| ☑ | Streptomyces sp. HT17 Ydz-XM 16S ribosomal RNA gene, partial sequence | Streptomyces sp. HT17 Ydz-XM | 2104 | 2104 | 99% | 0.0 | 98.82% | 1434 | EU384280.1 |
| ☑ | Streptomyces champavatii strain XQ45 16S ribosomal RNA gene, partial sequence | Streptomyces champavatii | 2102 | 2102 | 99% | 0.0 | 98.74% | 1415 | KU291361.1 |
| ☑ | Actinomycetia bacterium strain Qhu-D73 16S ribosomal RNA gene, partial sequence | Actinomycetes bacterium | 2102 | 2102 | 99% | 0.0 | 98.82% | 1406 | OP881652.1 |
| ☑ | Actinobacterium NDZKDS16 16S ribosomal RNA gene, partial sequence | actinobacterium NDZKDS16 | 2102 | 2102 | 99% | 0.0 | 98.82% | 1386 | KJ506725.1 |
| ☑ | Streptomyces sp. HT22 Ydz-XM 16S ribosomal RNA gene, partial sequence | Streptomyces sp. HT22 Ydz-XM | 2102 | 2102 | 99% | 0.0 | 98.82% | 1438 | EU384282.1 |
| ☑ | Streptomyces sp. strain BSP1 16S ribosomal RNA gene, partial sequence | Streptomyces sp. | 2100 | 2100 | 99% | 0.0 | 98.82% | 1494 | MT176505.1 |
| ☑ | Streptomyces sp. 604F chromosome, complete genome | Streptomyces sp. 604F | 2100 | 14699 | 99% | 0.0 | 98.82% | 6900059 | CP026490.1 |
| ☑ | Streptomyces sp. strain Bm21 16S ribosomal RNA gene, partial sequence | Streptomyces sp. | 2100 | 2100 | 99% | 0.0 | 98.82% | 1459 | MN187447.1 |
| ☑ | Streptomyces albidoflavus strain UYFA156 chromosome, complete genome | Streptomyces albidoflavus | 2100 | 14705 | 99% | 0.0 | 98.82% | 6823997 | CP040466.1 |
| ☑ | Streptomyces sp. strain 7A2 16S ribosomal RNA gene, partial sequence | Streptomyces sp. | 2100 | 2100 | 99% | 0.0 | 98.82% | 1432 | MK456470.1 |

**Figure S2**. Blast search for CMB-M0112.

## 2. GNPS Molecular Networking of active fractions

A

| CMB-MRB032 | ***Dirofilaria immitis* - Mf Motility % Inhibition Motility** | | | | ***H. contortus* - LDA** | | | |
|---|---|---|---|---|---|---|---|---|
| | **2.5 ppm** | | **0.25 ppm** | | **2.5 ppm** | | **0.25 ppm** | |
| | **Rep 1** | **Rep 2** | **Rep 1** | **Rep 2** | **Rep 1** | **Rep 2** | **Rep 1** | **Rep 2** |
| Hexane fraction | 100 | 99 | 100 | 99 | -2 | 0 | -1 | 0 |
| MeOH fraction | 100 | 100 | 66 | 86 | -1 | -6 | -3 | 1 |
| Fraction F08 | 0 | 3 | 0 | 11 | -8 | 3 | -2 | 5 |
| Fraction F09 | 100 | 100 | 99 | 99 | 97 | 97 | 2 | 8 |
| Fraction F10 | 100 | 100 | 100 | 99 | 98 | 98 | 2 | 4 |
| Fraction F11 | 100 | 100 | 100 | 97 | 35 | 41 | 2 | 2 |
| Fraction F12 | 100 | 100 | 100 | 99 | 56 | 65 | 11 | 20 |
| Fraction F13 | 100 | 99 | 100 | 100 | -8 | 2 | -3 | 0 |
| Fraction F14 | 100 | 100 | 4 | 6 | -1 | 6 | 0 | 1 |

B

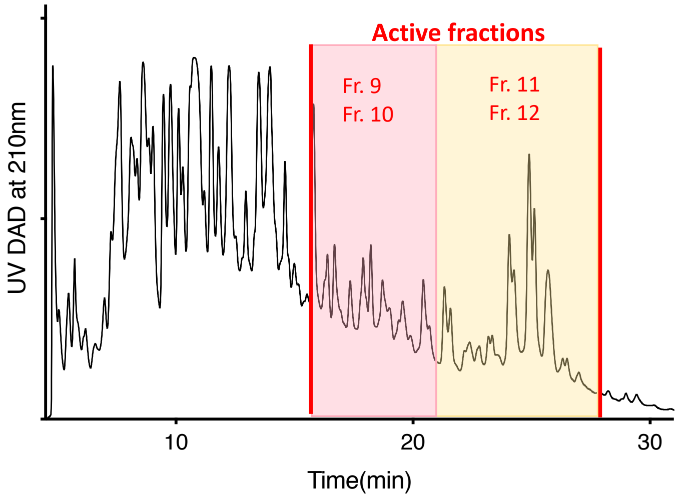


C

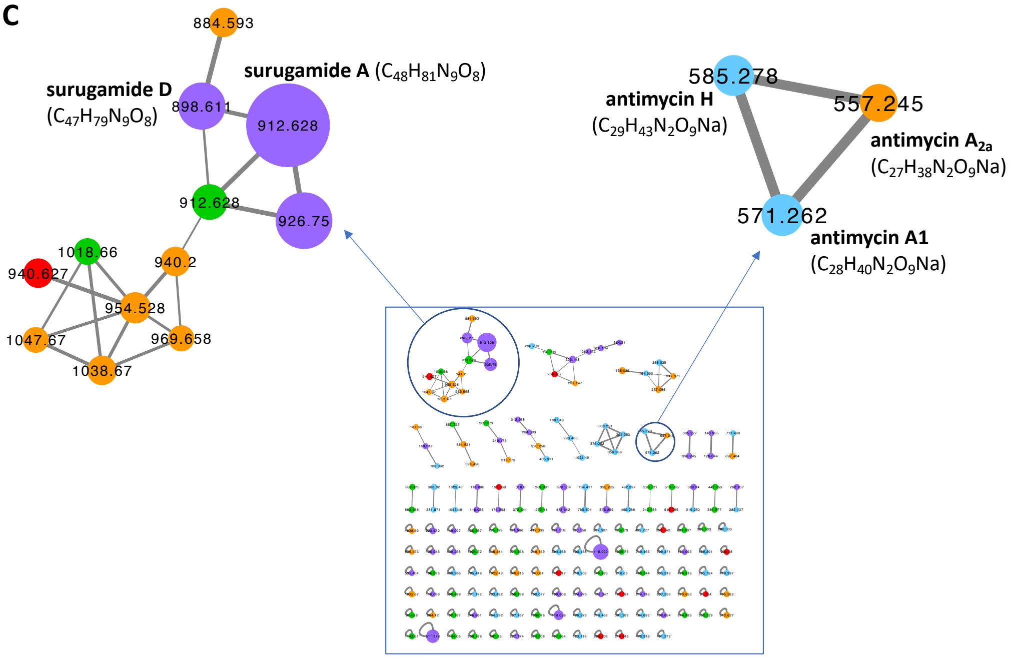


**Figure S3.** (A) Anthelmintics data for CMB-MRB032 HPLC fractions. (B) HPLC chromatograms for CMB-MRB032 MeOH soluble fractions, highlighting the active fractions. (C) GNPS molecular networking for CMB-MRB032 active fractions. Purple: present in all active fractions; Green: present in fraction 9; Red: present in fractions 9 and 10; Orange: present in fraction 10; Blue: present in fractions 11 and 12.

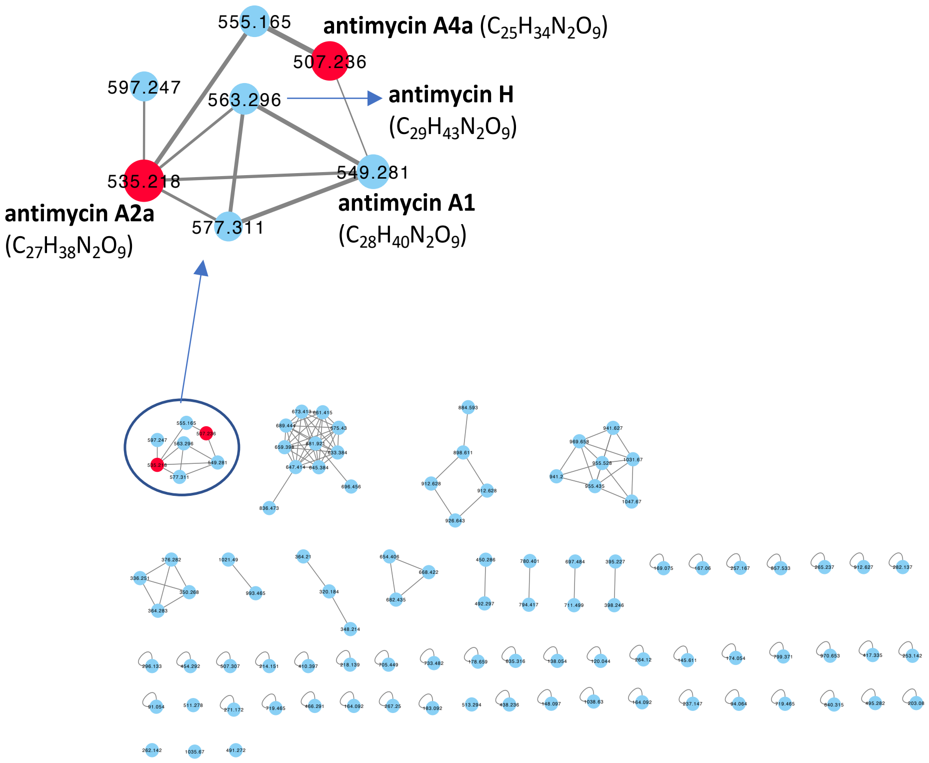


**Figure S4.** GNPS molecular networking of pure compounds antimycins A4a and A2a and CMB-MRB032 active fractions 11 and 12. Red: present in both standards and fractions; Blue: present in fractions 11 and 12.

### 3. GNPS Molecular Networking search for other strains producing surugamides

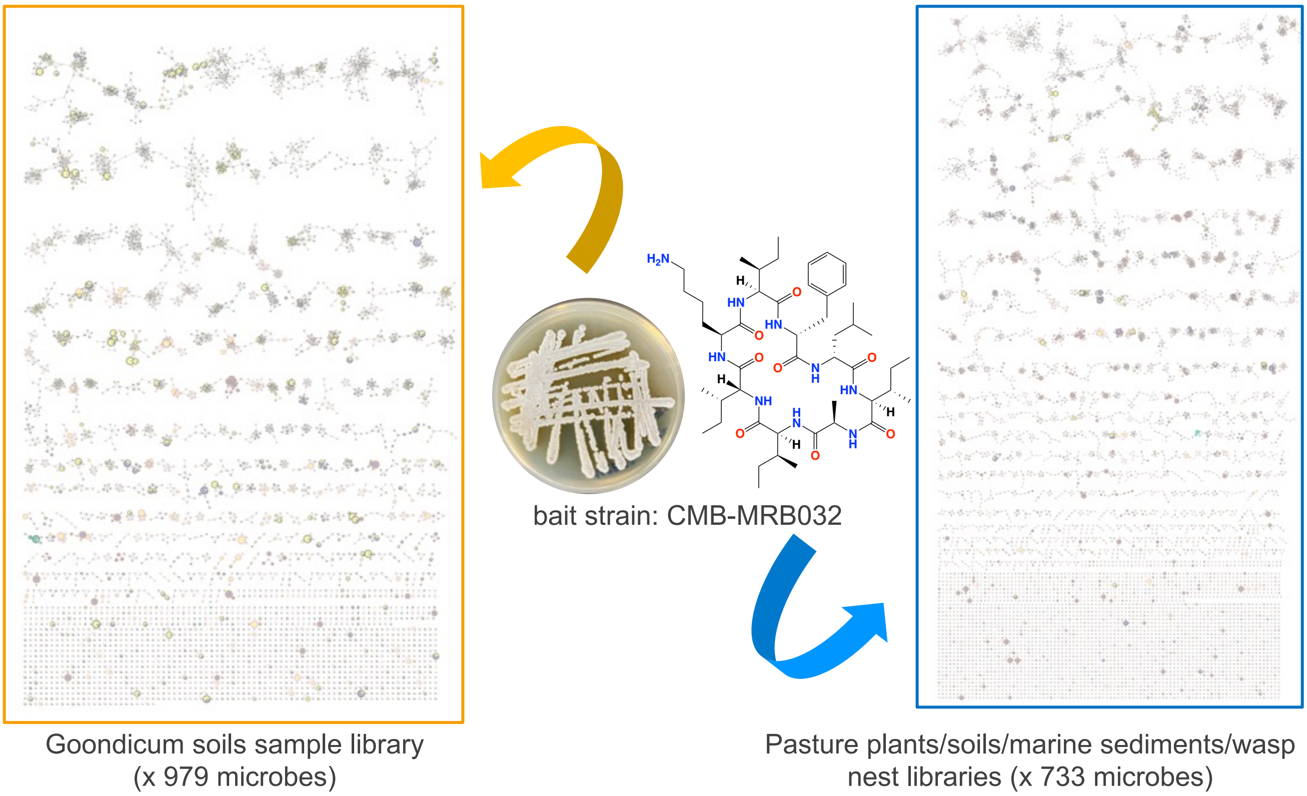


**Figure S5.** GNPS molecular network of surugamides producers in Capon Lab in-house microbial library.

**Table S1**. Surugamides producers identified by the GNPS molecular networking, their sources and anthelmintic activity.

| Microbe isolate codes | Microbe source | *D. immitis* MF motility $EC_{50}$ (μg/mL) | *H. contortus* L3 motility $EC_{50}$ (μg/mL) |
|---|---|---|---|
| CMB-MRB010[a] | sheep faeces | 0.04 | > 20 |
| CMB-MRB012[a] | sheep faeces | 0.05 | > 20 |
| CMB-MRB029[a] | sheep faeces | 0.05 | > 20 |
| CMB-MRB030[a] | sheep faeces | 0.04 | > 20 |
| CMB-MRB032[a] | sheep faeces | 0.06 | 3.0 |
| CMB-M0112[b] | marine sediment | 1.2 | > 20 |
| CMB-M0116[b] | marine sediment | 1.7 | > 20 |
| CMB-M0118[b] | marine sediment | 1.4 | > 20 |
| ACM4361 | ATCC-25495[d] | 5.2 | > 25 |
| S4S00071B10 | Goondicum soil | < 25[e] | < 25[e] |
| S4S00191A07 | Goondicum soil | < 25[e] | < 25[e] |
| CMB-CS049[c] | cone snail | < 25[e] | > 25 |
| CMB-CS050[c] | cone snail | > 25 | > 25 |
| CMB-CS051[c] | cone snail | < 25[e] | > 25 |
| CMB-CS075 | cone snail | < 25[e] | > 25 |
| CMB-CS146 | cone snail | < 25[e] | > 25 |

[a-c] Strains with same subscript are replicates based on UPLC-DAD profiles

[d] ATCC type strain *Streptomyces sampsonii* (Millard and Burr) Waksman isolated from potato scab, synonym *Streptomyces albidoflavus*.

[e] Tested at a single concentration, and exhibiting $EC_{75} – EC_{100}$ at 25 μg/mL.

**Table S2**. Taxonomic identification[a] of selected surugamide producers

| Strain | Accession number | Strain identification |
|---|---|---|
| CMB-CS051 | OR346109 | *Streptomyces lividans* (99.1% identity) |
| S4S-00071B10 | OR887069 | *Streptomyces showdoensis* (98.2% identity) |
| S4S-00191A07 | OR879801 | *Streptomyces hydrogenans* (98.6% identity) |
| CMB-M0112 | OR879802 | *Streptomyces lividans* (98.8% identity) |
| CMB-MRB032 | OR346138 | *Streptomyces koyangensis* (99.5% identity) |

[a] 16S rRNA sequences were obtained following the protocol describes in Section 1.

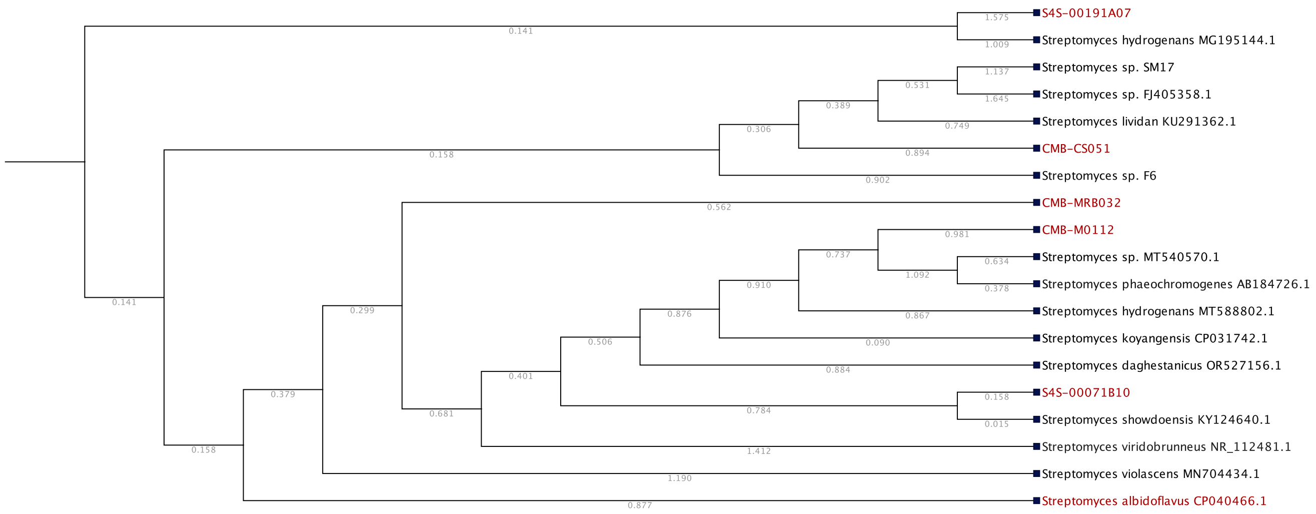


**Figure S6**. Phylogenetic tree of surugamide-producing strains (red) was constructed using Geneious Prime Software. The phylogenetic tree was generated based on the 16S rRNA gene sequences of selected *Streptomyces* strains. Bootstrap values are indicated at the nodes and represent the reliability of the clades; higher values denote greater statistical support. The tree illustrates the phylogenetic relationships among the strains, with smaller numbers indicating greater phylogenetic distances.

## 4. Media MATRIX and Time study

CMB-MRB032 and CMB-M0112 were selected for media MATRIX study, in which the two bacteria were cultivated on/in 11 different media (Table S3) under three conditions (agar base, shaken broth and static broth) in microbioreactors (Figure S7) to give 33 different extracts, which were analysed by UPLC. The results showed that surugamides were produced in low quantity in all media for both strains after 7-days cultivation, therefore the original ISP2 agar were chosen for time study (Figure S8).

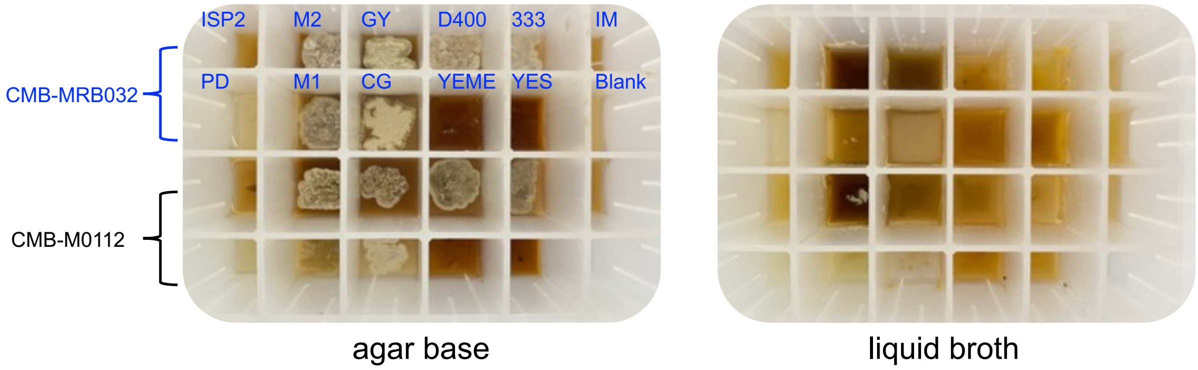


**Figure S7**. CMB-MRB032 and CMB-M0112 in microbioreactors in media MATRIX.

**Table S3.** Components of media used in CMB-MRB032 and CMB-M0112 cultivation in microbioreactors

| Medium | Composition (per Litre) |
| --- | --- |
| International Streptomyces Project-2 (ISP2) | yeast extract (4.0 g), malt extract (10.0 g), glucose (4.0 g), pH 7.2 |
| M2 | mannitol (40.0 g), maltose (40.0 g), yeast extract (10.0 g), $K_2HPO_4$ (2.0 g), $MgSO_4.7H_2O$ (0.5 g), $FeSO_4.7H2O$ (0.01 g) |
| Glucose Yeast Extract Starch (GY) | yeast extract (4.0 g), malt extract (10.0 g), dextrose (4.0 g), $CaCO_3$ (2.0 g), soluble starch (20.0 g) |
| D400 | glycose (10.0 g), malt extract (20 g), peptone (3.0 g), soluble starch (20 g), yeast extract (5.0 g), $CaCO_3$ (3.0 g) |
| 333 | glucose (5.0 g), peptone (3.0 g), soluble starch (10.0 g), yeast extract (3.0 g), $CaCO_3$ (3.0 g) |
| Inhibitory Mould (IM) | yeast extract (4.0 g), malt extract (10.0 g), glucose (4 g), mannitol (4.0 g) |
| Potato Dextrose (PD) | potato extract (4.0 g), dextrose (20.0 g), pH 5.6 |
| M1 | peptone (2.0 g), yeast extract (4.0 g), starch (10.0 g), pH 7.0 |
| Chloramphenicol Glucose (CG) | glycerol (30.0 g), casein peptone (2.0 g), $K_2HPO_4$ (1.0 g), NaCl (1.0 g), $MgSO_4 \cdot 7H_2O$ (0.5 g), trace element solution (5.0 mL) |
| Yeast Extract-Malt Extract (YEME) | yeast extract (3.0 g), malt extract (3.0 g), peptone (5.0 g), glucose (10.0 g), sucrose (250.0 g) |
| Yeast Extract Supplements (YES) | sucrose (150 g), yeast extract (20 g), $MgSO_4.7H_2O$ (0.5 g), $ZnSO_4.7H_2O$ (0.01 g), $CuSO_4.5H_2O$ (0.005 g) |

A time study on CMB-M0112 on ISP2 agar revealed the 28-day culture contained isolable amount of surugamides.

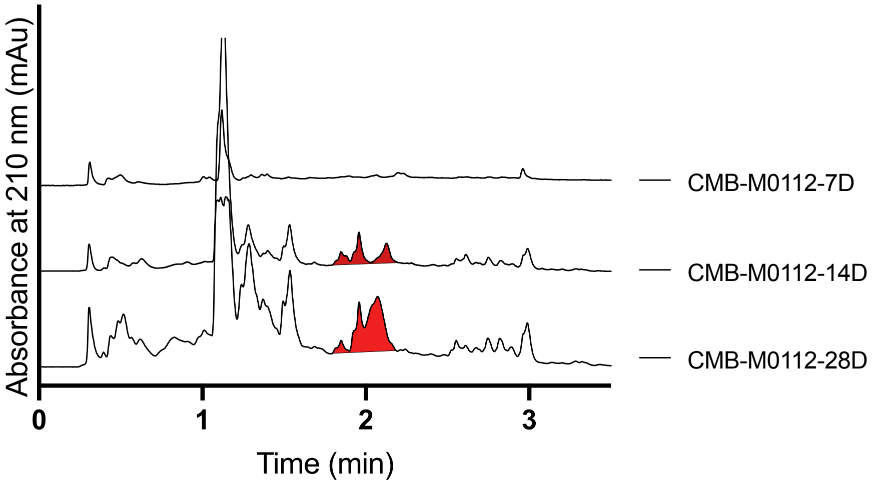


**Figure S8**. Production of surugamides in CMB-M0112 ISP2 agar at different times

## 5. Marfey's analysis

Samples of surugamides **3**–**8** (50 μg each) were subjected the standard Marfey's hydrolysis and derivatisation, after which individual aliquots of Marfey's derivatised analytes (3 μL) were subjected to LCMS analysis.

*Hydrolysis and derivatisation.* A sample analyte (50 μg) in 6 M HCl (100 μL) were heated to 100 °C in a sealed vial for 12 h, after which the hydrolysate was concentrated to dryness at 40 °C under a stream of dry $N_2$. The hydrolysate was then treated with 1 M NaHCO3 (20 μL) and L-FDAA (1-fluoro-2,4-dinitrophenyl-5-L-alanine amide) as a 1% (w/v) solution in acetone (40 μL) at 40 °C for 1 h, after which the reaction was neutralised with 1 M HCl (20 μL), diluted with MeCN (200 μL) and filtered (0.45 μm PTFE) prior to analysis.

*Standard Marfey's HPLC method.* An aliquot of Marfey's derivatised analyte (3 μL) was subjected to HPLC-DAD-MS analysis using a binary solvent system (Phase A: 95% $H_2O$ : 5% MeCN : 0.1% formic acid; Phase B: 95% MeOH : 5% MeCN : 0.1% formic acid) on an Agilent Poroshell 120 SB-C8 2.7 μm, 3.0×150 mm column, at 50 °C with a 0.8 mL/min linear gradient over 29 min from 16% to 63% Phase B in A, and with DAD (340 nm) and ESI(±)MS monitoring, supported by single ion extraction (SIE) methodology, and with comparison to authentic standards of Marfey's derivatized amino acids.

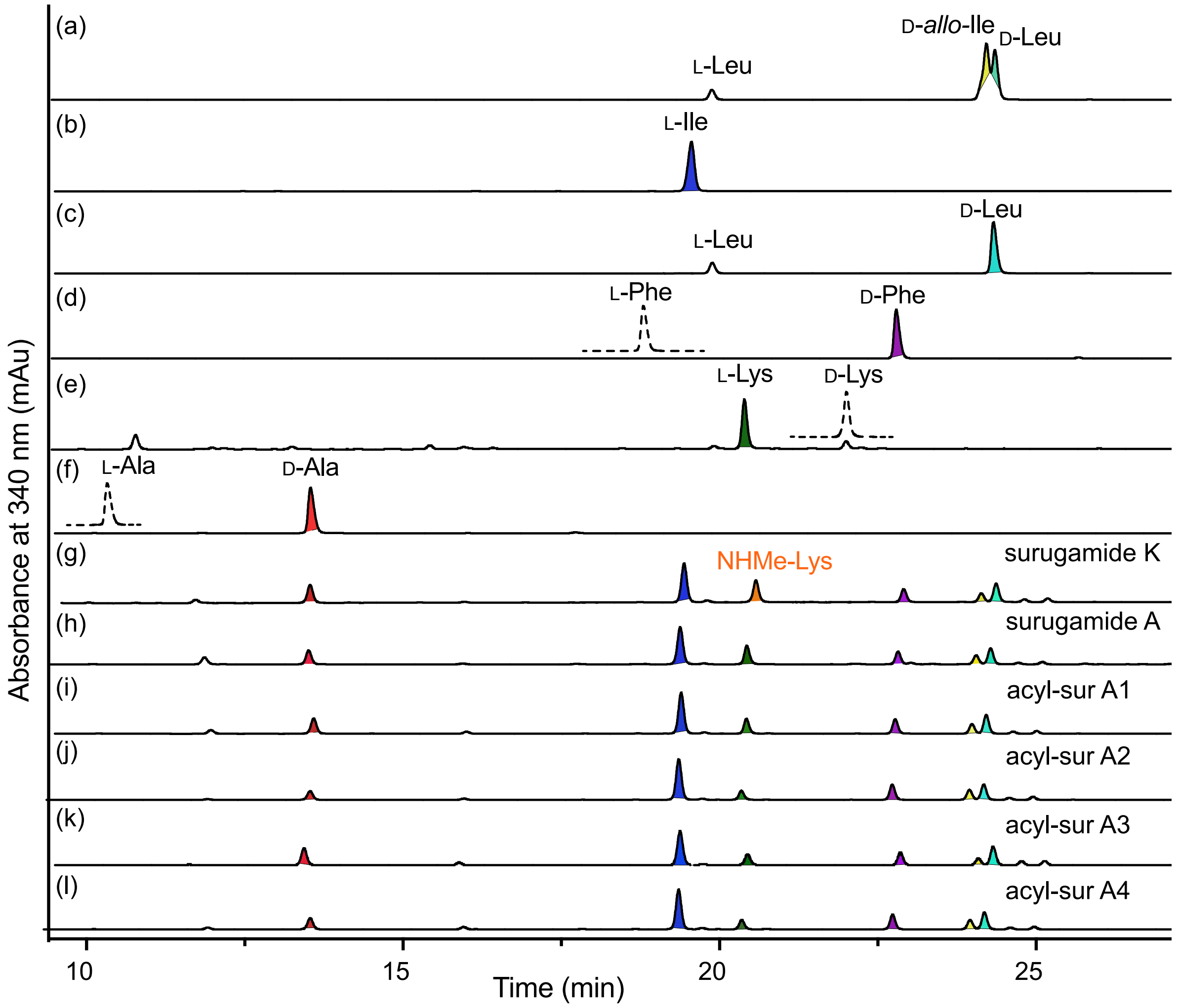


**Figure S9.** $C_{18}$ Marfey's analysis of surugamides. HPLC-DAD (340 nm) chromatograms of L-FDAA derivatives of authentic standards of (a) D-alloIle and D-Leu, (b) L-Ile, (c) D-Leu, (d) D-Phe, (e) L-Lys, (f) D-Ala. HPLC-DAD (340 nm) chromatograms of L-FDAA amino acid derivatives of acid hydrolysates of (g) surugamide K (4), (h) surugamide A (3), (i) acyl-surugamide A1 (5), (j) acyl-surugamide A2 (6), (k) acyl-surugamide A3 (7), (l) acyl-surugamide A4 (8).

## 6. Spectroscopic data for natural products 3-8

**Table S4**. 1D and 2D NMR (600 MHz, DMSO-$d_6$) data for synthetic surugamide A (**3**)

| | surugamide A | | | | Reported data[1] | |
|---|---|---|---|---|---|---|
| | $\delta_C$ | $\delta_H$, mult ($J$ in Hz) | COSY | HMBC | $\delta_C$ | $\delta_H$, mult ($J$ in Hz) |
| **L-Ile[1]** | | | | | | |
| 1 | 172.4 | - | | | 172.1 | - |
| 2 | 57.5 | 4.18, m | 3, NH | 1, 3, 4 | 57.8 | 4.14, m |
| 3 | 35.3 | 1.73, m | 2 | 5, 6 | 35.3 | 1.72, m |
| 4 | 24.4 | a. 1.46, m | 3 | 3, 5, 6 | 24.7 | a. 1.46, m |
| | | b. 1.13, m | | 5, 6 | | b. 1.13, m |
| 5 | 10.6 | 0.82[a] | | | 11.9 | 0.79[a] |
| 6 | 15.0 | 0.80[b] | | | 14.8 | 0.79[a] |
| NH | | 8.27, d (7.4) | 2 | 1, Ala[8]-1 | | 8.50, d (8.3) |
| **D-allo-Ile[2]** | | | | | | |
| 1 | 170.9 | - | | | N.D. | |
| 2 | 56.7 | 4.16, m | 3, NH | 1, 3, 6 | 57.0 | 4.13, m |
| 3 | 36.2 | 1.83, m | 2, 4 | 5, 6 | 36.3 | 1.81, m |
| 4 | 25.7 | a. 1.34, m | 3 | 2, 3, 5, 6 | 26.0 | a. 1.33, m |
| | | b. 1.21, m | | 5, 6 | | b. 1.21, m |
| 5 | 11.5 | 0.82[a] | | | 11.9 | 0.79[a] |
| 6 | 14.4 | 0.82[a] | | | 14.8 | 0.79[a] |
| NH | | 8.04, d (7.2) | 2 | 1, 2, Ile[1]-1 | | 7.98, br s |
| **L-Lys[3]** | | | | | | |
| 1 | 172.4 | - | | | N.D. | |
| 2 | 51.7 | 4.31, m | 3, NH | 1, allo-Ile[2]-1 | 52.1 | 4.31, m |
| 3 | 31.3 | a. 1.54 m | 2, 4 | | 31.7 | a. 1.51, m |
| | | b. 1.41, m | | | | b. 1.35, m |
| 4 | 21.8 | a. 1.25, m | 5 | | 22.3 | a. 1.17, m |
| | | b. 1.16, m | | | | b. 1.08, m |
| 5 | 26.1 | a. 1.42, m | 4, 6 | | 28.2 | a. 1.35 m |
| | | b. 1.35, m | 6 | | | b. 1.25, m |
| 6 | 38.9 | 2.71, m | 5a, 5b, | | 39.7 | 2.58 |
| 6-$NH_2$ | | 7.62, br s | 6 | | | |
| NH | | 7.52, br s | 2 | | | 7.45, br s |
| **L-Ile[4]** | | | | | | |
| 1 | 171.2 | - | | | N.D. | - |
| 2 | 57.8 | 3.87, dd (6.4, 6.4) | 3, NH | 1, 3, 6 | 58.2 | 3.82 t, (6.2) |
| 3 | 35.7[c] | 1.45, m | 2, 6 | 4 | 35.9 | 1.41, m |
| 4 | 24.7 | a. 1.18, m | 5 | | 25.1 | a. 1.18, m |
| | | b. 0.81[b] | | | | b. 0.79, m |
| 5 | 11.0 | 0.68, dd (7.2, 7.2) | 4 | 3, 4 | 11.4 | 0.66, t (7.2) |
| 6 | 14.7 | 0.44, d (6.9) | 3 | 2, 3, 4 | 15.0 | 0.41, d (6.9) |
| NH | | 7.85, br s | 2 | | | 7.91, d (7.6) |
| **D-Phe[5]** | | | | | | |
| 1 | 171.0 | | | | 171.5 | - |
| 2 | 54.7 | 4.37, ddd (11.5, 8.2, 3.2) | 3, NH | 1, Ile[4]-1 | 55.0 | 4.35, m |
| 3 | 36.5 | a. 3.25, m | 2 | 4, 5, 6 | 36.8 | a. 3.25, dd (13.6, |
| | | b. 2.69, dd (13.7, 11.5) | 2 | | | b. 2.67, t (13.6) |
| 4 | 138.0 | - | | | 138.0 | - |
| 5/9 | 129.0 | 7.23[c] | 6/8 | 3, 7 | 129.3 | 7.23[b] |
| 6/8 | 128.1 | 7.23[c] | 5/9, 7 | 4 | 128.4 | 7.22[b] |
| 7 | 126.3 | 7.17, t (6.7) | 6/8 | 5/9 | 126.6 | 7.16, br d |
| NH | | 8.46, d (8.2) | 2 | 3, Ile[4]-1 | | 8.45 |

| | | | | | | |
|---|---|---|---|---|---|---|
| **D-Leu$^{6}$** | | | | | | |
| 1 | 172.6 | - | | | 172.9 | - |
| 2 | 52.3 | 4.23, m | 3a, 3b, NH | 1, 3, 4 | 52.7 | 4.20, m |
| 3 | 40.2 | a. 1.86, m | 2 | | 40.4 | a. 1.88, m |
| | | b. 1.48, m | 2 | | | b. 1.46, m |
| 4 | 24.3 | 1.68, m | 5 | | 24.6 | 1.68, m |
| 5 | 21.5 | 0.86, d (6.7) | 4 | 3, 4, 6 | 21.7 | 0.84, d (6.9) |
| 6 | 23.2 | 0.94, d (6.9) | 4 | 3, 4, 6 | 23.5 | 0.92, d (6.9) |
| NH | | 7.70, d (7.4) | 2 | 2, 3, Phe$^{5}$-1 | | 7.72, d (7.6) |
| **L-Ile$^{7}$** | | | | | | |
| 1 | 169.8 | - | | | 169.8 | - |
| 2 | 57.4 | 4.07, dd (7.2, 7.2) | 3, NH | 1, 3, 4, 6, Leu$^{6}$-1 | 57.7 | 4.06, t (7.2) |
| 3 | 35.7[c] | 1.79, m | 2 | 4, 5, 6 | 36.1 | 1.77, m |
| 4 | 23.9 | a. 1.26, m | | | 24.2 | a. 1.20, m |
| | | b. 1.13, m | | | | b. 1.11, m |
| 5 | 11.3 | 0.80[b] | | | 11.9 | 0.79[a] |
| 6 | 15.2 | 0.80[b] | | | 15.4 | 0.79[a] |
| NH | | 7.14, br s | 2 | | | 7.00, br s |
| **D-Ala$^{8}$** | | | | | | |
| 1 | 172.4 | - | | | 172.8 | - |
| 2 | 47.9 | 4.27, m | 3, NH | 1, Ile$^{7}$-1 | 48.2 | 4.27, m |
| 3 | 19.0 | 1.22, d (6.6) | 2 | 1 | 19.2 | 1.19, d (6.6) |
| NH | | 7.78, d (6.0) | 2 | 1, Ile$^{7}$-1 | | 7.73, br s |

[a-c] Resonances with the same superscripts within a column are overlapped.

[1] Takada *et al. J. Org. Chem.* **2013**, *78*, 6746-6750.

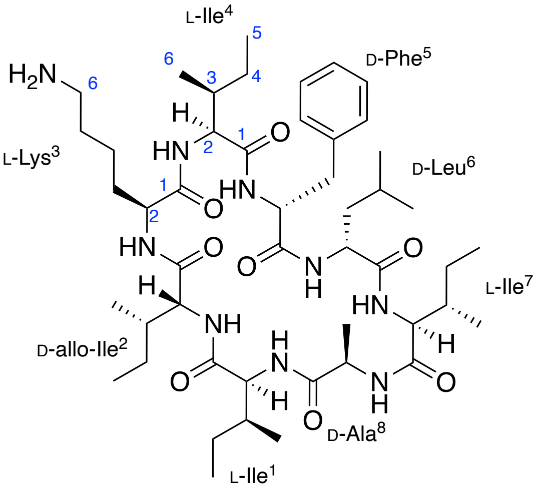


surugamide A (**3**)

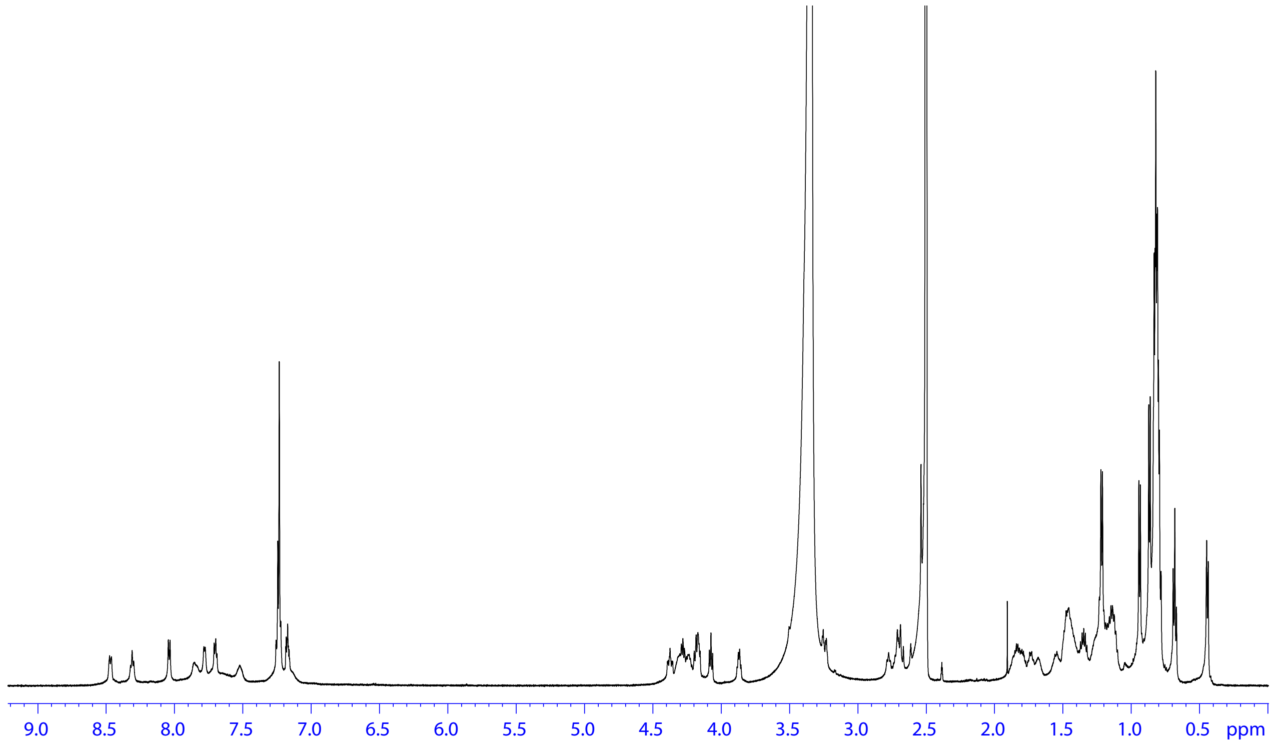


**Figure S10**. $^{1}$H NMR (600 MHz, DMSO-$d_6$) spectrum of surugamide A (**3**).

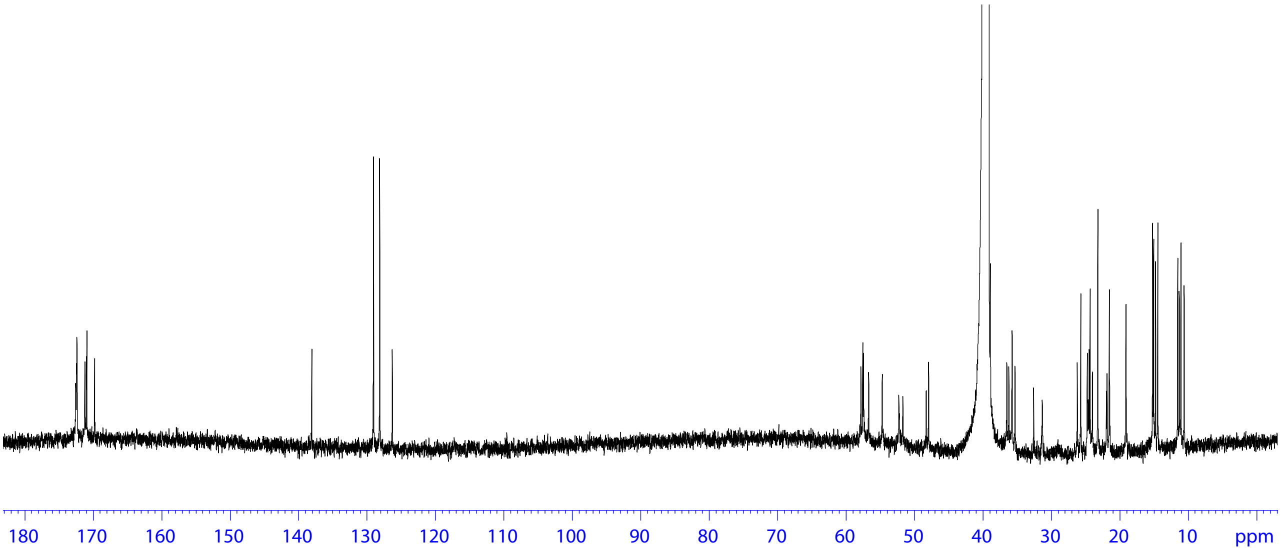


**Figure S11**. $^{13}$C NMR (150 MHz, DMSO-$d_6$) spectrum of surugamide A (**3**)

**Table S5**. 1D and 2D NMR (600 MHz, DMSO-$d_6$) data for surugamide K (**4**)

| | $\delta_C$ | $\delta_H$, mult (*J* in Hz) | COSY | HMBC |
|---|---|---|---|---|
| **L-Ile[1]** | | | | |
| 1 | 172.4[a] | - | | |
| 2 | 57.7 | 4.16, m | 3, NH | |
| 3 | 35.2 | 1.74, m | 2, 4b, 6 | |
| 4 | 24.5[b] | a. 1.46, m | 4b, 5 | |
| | | b. 1.13, m | 3, 4a | |
| 5 | 10.6 | 0.81[a] | 4a | |
| 6 | 15.1 | 0.80[a] | 3 | |
| NH | | 8.03, d (7.5) | 2 | |
| **D-allo-Ile[2]** | | | | |
| 1 | 171.0[c] | - | | |
| 2 | 56.7 | 4.16, m | 3, NH | |
| 3 | 36.2 | 1.83, m | 2, 4b, 6 | |
| 4 | 25.7 | a. 1.34, m | 4b, 5 | |
| | | b. 1.21, m | 3, 4a | |
| 5 | 11.5 | 0.82[b] | 4a | |
| 6 | 14.5 | 0.82[b] | 3 | |
| NH | | 8.34, d (7.5) | 2 | |
| **NHMe-L-Lys[3]** | | | | |
| 1 | 172.4[a] | - | | |
| 2 | 51.7 | 4.31, m | 3a, 3b, NH | |
| 3 | 31.3 | a. 1.53, m | 2 | |
| | | b. 1.23, m | 2 | |
| 4 | 22.0 | a. 1.17, m | | |
| | | b. 1.13, m | | |
| 5 | 24.5[b] | a. 1.43, m | 6 | |
| | | b. 1.38, m | 6 | |
| 6 | 48.2 | 2.78, m | 5a, 5b, NHMe | |
| NHMe | 32.5 | 2.54, s | NHMe | |
| NHMe | | 8.41, br s | 6, NHMe | |
| NH | | 7.51, br s | 2 | |
| **L-Ile[4]** | | | | |
| 1 | 171.1[c] | - | | |
| 2 | 57.9 | 3.85, dd (6.6, 6.6) | 3, NH | 6 |
| 3 | 35.7[d] | 1.45, m | 2, 4b, 6 | 5, 6 |
| 4 | 24.8 | a. 1.19, m | 5 | 5, 6 |
| | | b. 0.82[b] | 3 | |
| 5 | 11.1 | 0.68, t (7.2) | 4 | |
| 6 | 14.8 | 0.43, d (6.8) | 3 | |
| NH | | 7.86, br s | 2 | |
| **D-Phe[5]** | | | | |
| 1 | 171.1[c] | | | |
| 2 | 54.8 | 4.37, ddd (11.5, 8.2, 3.2) | 3a, 3b, NH | |
| 3 | 36.5 | a. 3.24, dd (13.8, 3.2) | 2 | |
| | | b. 2.69, dd (13.8, 11.5) | 2 | |
| 4 | 138.1 | - | | |
| 5/9 | 129.1 | 7.23, m | | 3, 7 |
| 6/8 | 128.2 | 7.24, m | 7 | 4 |
| 7 | 126.3 | 7.17, m | 6/8 | 5/9 |
| NH | | 8.49, d (8.3) | 2 | |
| **D-Leu[6]** | | | | |
| 1 | 172.7 | - | | |

| | | | | |
|---|---|---|---|---|
| 2 | 52.3 | 4.22, m | 3a, 3b, NH | 1, 3, 4 |
| 3 | 40.2 | a. 1.87, m | 2, 3b | 5, 6 |
| | | b. 1.47, m | 2, 3a, 4 | |
| 4 | 24.3 | 1.67, m | 3b, 5, 6 | 5, 6 |
| 5 | 21.5 | 0.86, d (6.6) | 4 | 6 |
| 6 | 23.2 | 0.93, d (6.6) | 4 | 5 |
| NH | | 7.71, d (7.5) | 2 | Phe[5]-1 |
| **L-Ile[7]** | | | | |
| 1 | 169.8 | - | | |
| 2 | 57.5 | 4.06, dd (8.5, 8.5) | 3, NH | |
| 3 | 35.7[d] | 1.79, m | 2, 4b, 6 | |
| 4 | 24.0 | a. 1.25, m | 4b, 5 | |
| | | b. 1.13, m | 3, 4a, 5 | |
| 5 | 11.3 | 0.79, dd (7.6, 7.6) | 4a, 4b | |
| 6 | 15.2 | 0.80[a] | 3 | |
| NH | | 7.11, m | 2 | |
| **D-Ala[8]** | | | | |
| 1 | 172.4[a] | - | | |
| 2 | 48.1 | 4.27, m | 3, NH | 3 |
| 3 | 19.1 | 1.21, d (7.0) | 2 | 1 |
| NH | | 7.77, d (6.6) | 2 | |

[a-d] Resonances with the same superscripts within a column are overlapped.

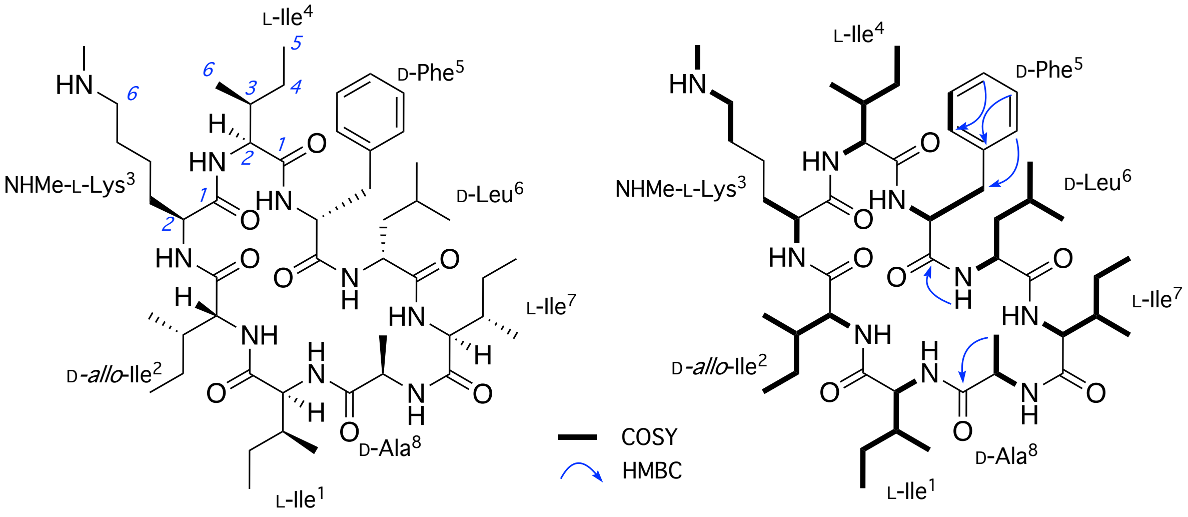


surugamide K (**4**)

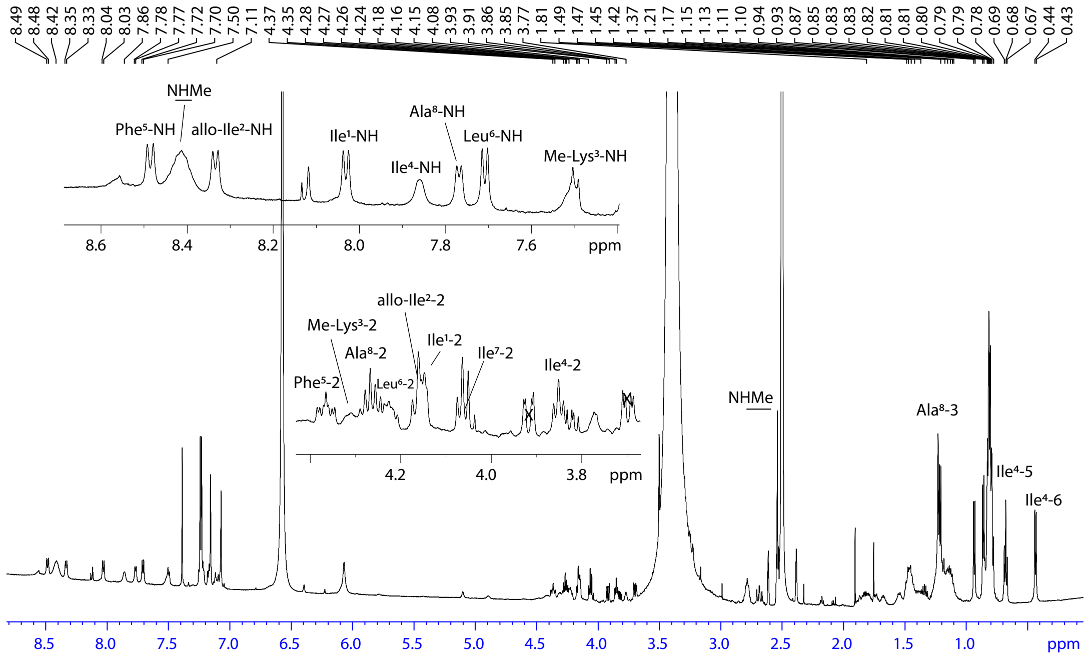


**Figure S12**. $^{1}H$ NMR (600 MHz, DMSO-$d_6$) spectrum of surugamide K (**4**)

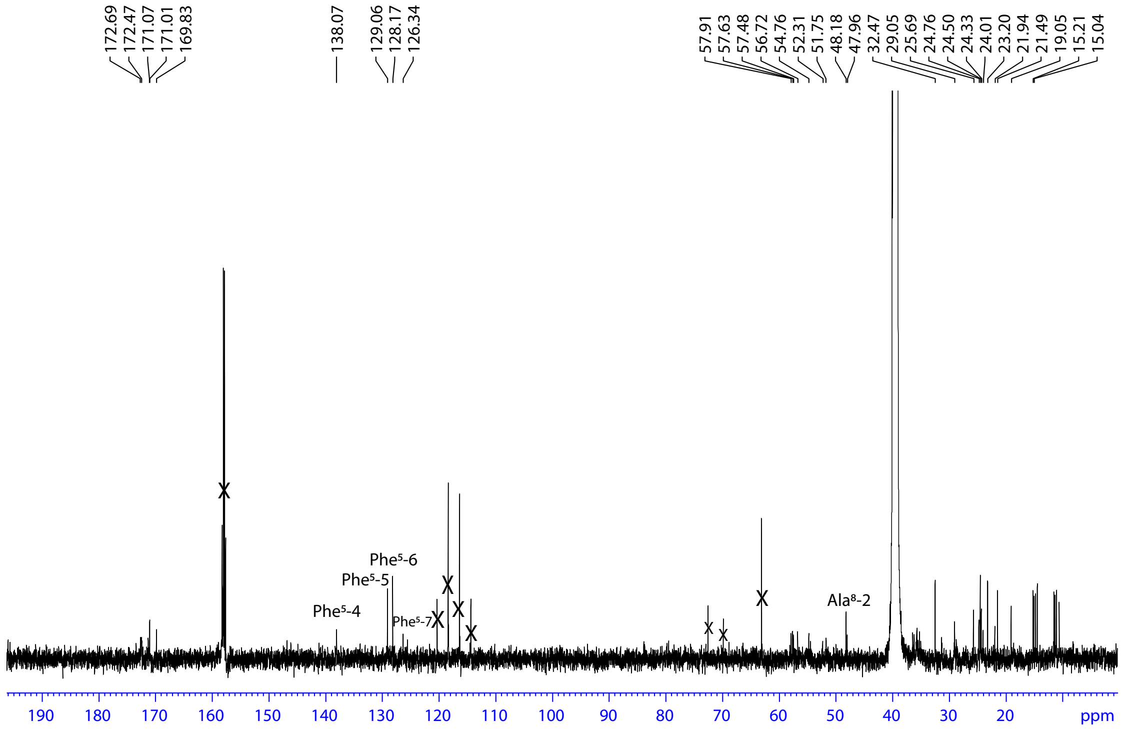


**Figure S13**. $^{13}C$ NMR (150 MHz, DMSO-$d_6$) spectrum of surugamide K (**4**)

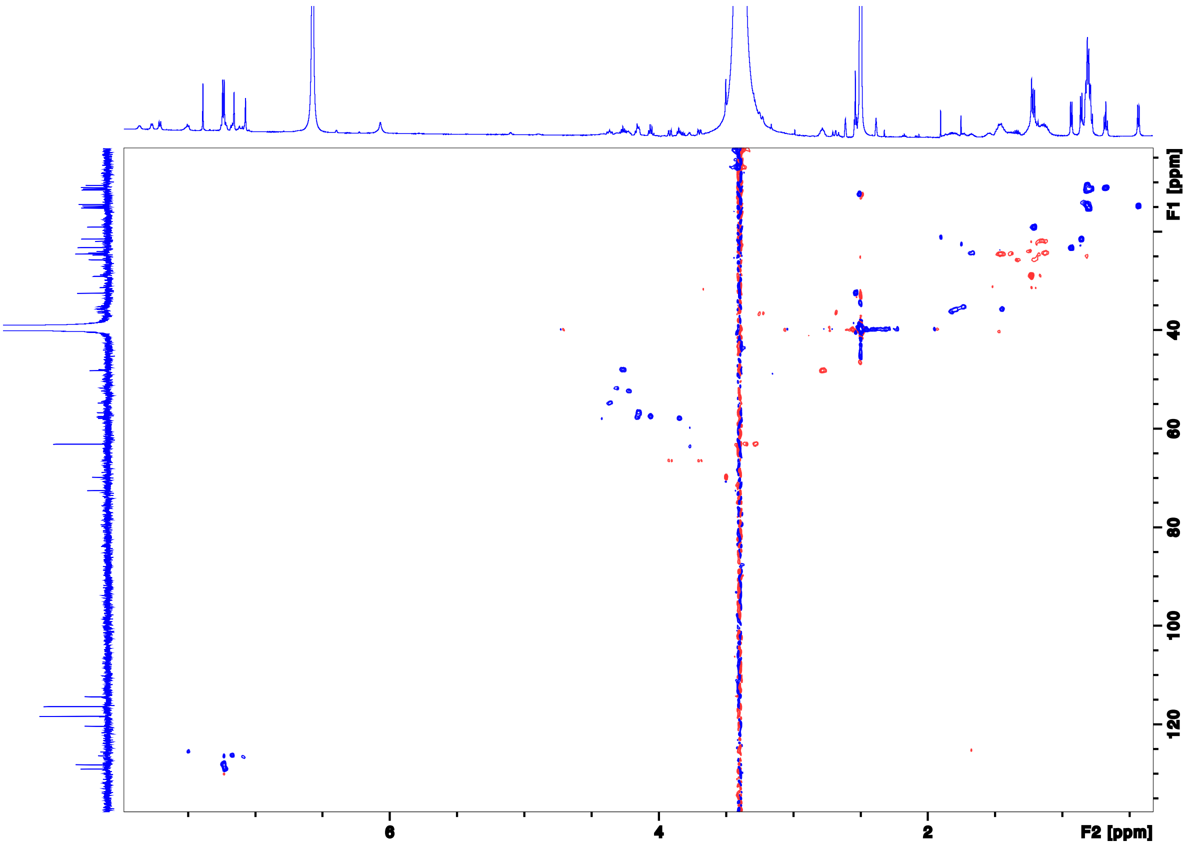


**Figure S14**. HSQC NMR (600 MHz, DMSO-$d_6$) spectrum of surugamide K (**4**)

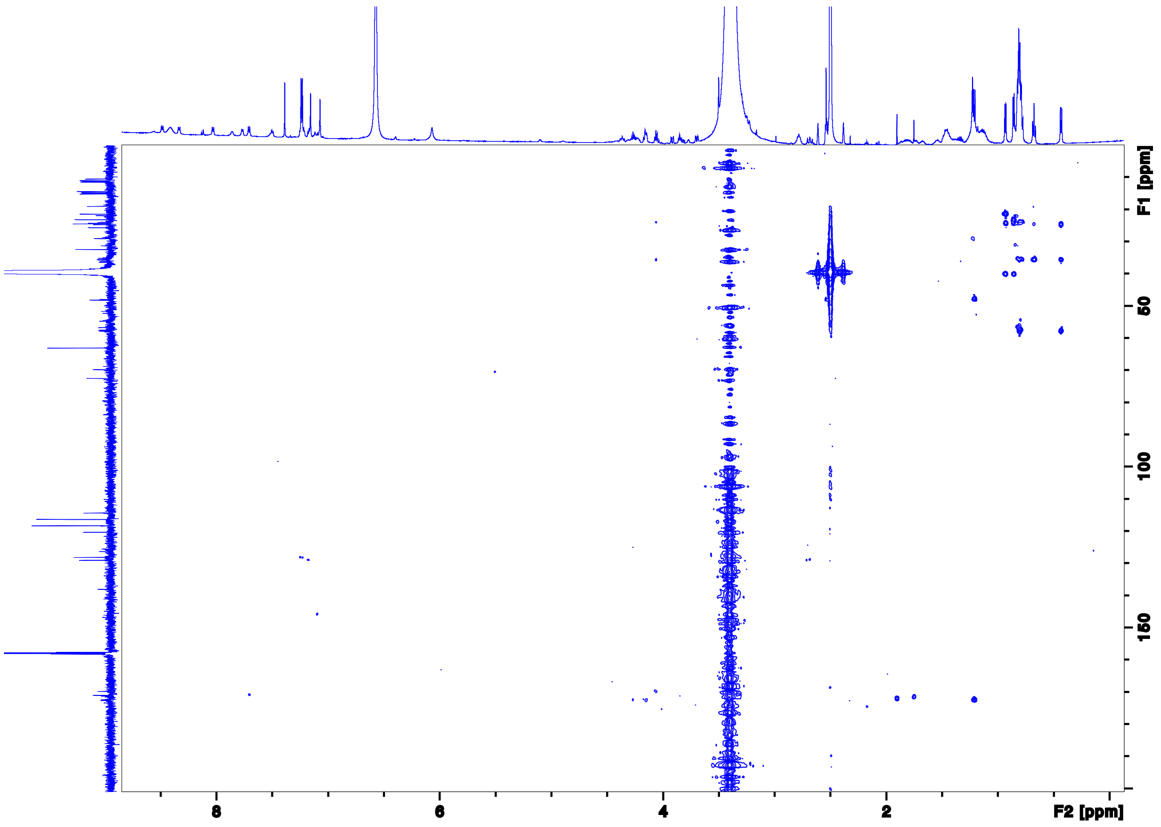


**Figure S15**. HMBC NMR (600 MHz, DMSO-$d_6$) spectrum of surugamide K (**4**)

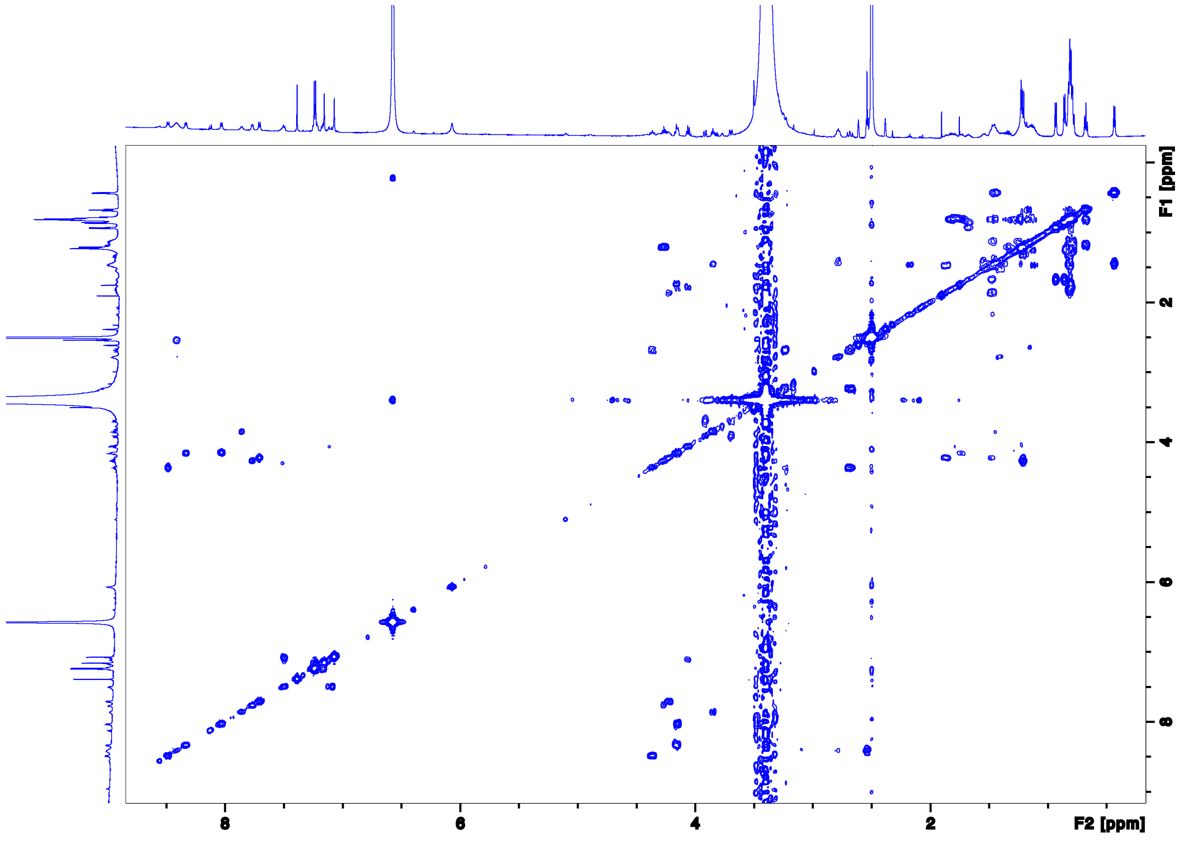


**Figure S16**. COSY NMR (600 MHz, DMSO-$d_6$) spectrum of surugamide K (**4**)

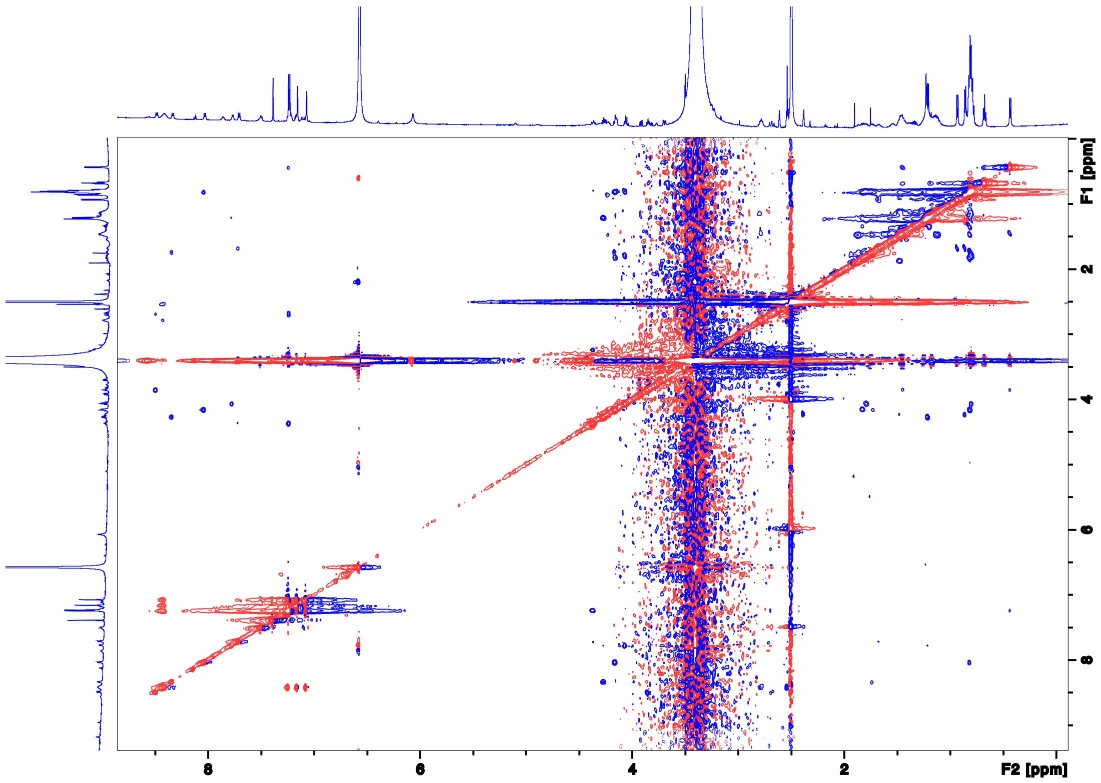


**Figure S17**. ROESY NMR (600 MHz, DMSO-$d_6$) spectrum of surugamide K (**4**)

# Mass Spectrum Molecular Formula Report

**Analysis Info**

| | | | |
|---|---|---|---|
| Analysis Name | D:\Data\Taizong\K_surugamide 926.d | Acquisition Date | 3/15/2023 3:18:38 PM |
| Method | tune-medhigh_AS.m | Operator | a.salim |
| Sample Name | K_surugamide 926 | Instrument / Ser# | micrOTOF 213750.00232 |
| Comment | | | |

**Acquisition Parameter**

| | | | | | |
|---|---|---|---|---|---|
| Source Type | ESI | Ion Polarity | Positive | Set Nebulizer | 0.5 Bar |
| Focus | Not active | | | Set Dry Heater | 180 °C |
| Scan Begin | 200 m/z | Set Capillary | 4500 V | Set Dry Gas | 5.0 l/min |
| Scan End | 1500 m/z | Set End Plate Offset | -500 V | Set Divert Valve | Source |

**Generate Molecular Formula Parameter**

| | | |
|---|---|---|
| Formula, min. | | |
| Formula, max. | | |
| Measured m/z | Tolerance | Charge |
| Check Valence | Minimum | Maximum |
| Nirogen Rule | Electron Configuration | |
| Filter H/C Ratio | Minimum | Maximum |
| Estimate Carbon | | |

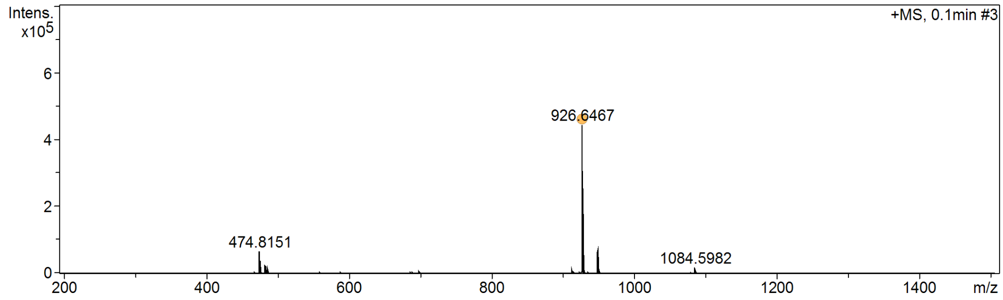


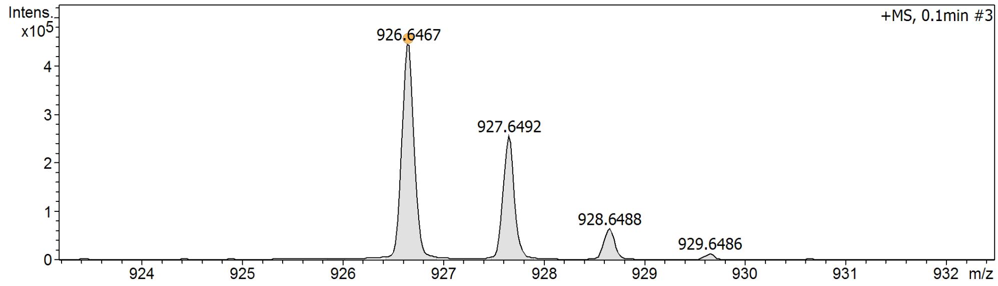


| Meas. m/z | # | Ion Formula | m/z | err [ppm] | mSigma | # Sigma | Score | rdb | e⁻ Conf | N-Rule |
|---|---|---|---|---|---|---|---|---|---|---|
| 926.6467 | 1 | C49H84N9O8 | 926.6437 | 3.2 | 17.0 | 1 | 100.00 | 12.5 | even | ok |
| | 2 | C44H84N11O10 | 926.6397 | -7.5 | 20.8 | 2 | 0.32 | 8.5 | even | ok |
| | 3 | C43H84N13O9 | 926.6509 | 4.6 | 21.9 | 3 | 21.29 | 8.5 | even | ok |

Bruker Compass DataAnalysis 4.1 printed: 3/15/2023 3:29:57 PM Page 1 of 1

**Figure S18**. HRMS measurement for surugamide K (**4**)

**Table S6**. 1D and 2D NMR (600 MHz, DMSO-$d_6$) data for acyl-surugamide A1 (**5**)

| | $\delta_C$ | $\delta_H$, mult (*J* in Hz) | COSY | HMBC |
|---|---|---|---|---|
| **L-Ile[1]** | | | | |
| 1 | 172.1 | - | | |
| 2 | 57.6 | 4.16, dd (8.0, 8.0) | 3, NH | 1 |
| 3 | 35.2 | 1.75, m | 2, 4b, 6 | |
| 4 | 24.4 | a. 1.46, m | 4b, 5 | |
| | | b. 1.11, m | 3, 4a | |
| 5 | 10.6 | 0.81[a] | 4a | |
| 6 | 15.1 | 0.80[a] | 3 | |
| NH | | 8.29, d (8.0) | 2 | Ala[8]-1 |
| **D-allo-Ile[2]** | | | | |
| 1 | 170.9 | - | | |
| 2 | 56.6 | 4.18, m | 3, NH | 1 |
| 3 | 36.3 | 1.81, m | 2, 4b, 6 | |
| 4 | 25.7 | a. 1.33, m | 4b, 5 | 5, 6 |
| | | b. 1.20, m | 3, 4a | 5, 6 |
| 5 | 11.4 | 0.82[a] | 4a | |
| 6 | 14.4 | 0.81[a] | 3 | |
| NH | | 7.95[b] | 2 | Ile[1]-1 |
| **N-acetyl-L-Lys[3]** | | | | |
| 1 | n.d. | - | | |
| 2 | 52.1 | 4.28, m | 3a, 3b, NH | |
| 3 | 31.6 | a. 1.54, m | 2 | |
| | | b. 1.42, m | 2 | |
| 4 | 22.5 | a. 1.21, m | | |
| | | b. 1.14, m | | |
| 5 | 28.2 | a. 1.30, m | 6 | |
| | | b. 1.17, m | 6 | |
| 6 | 37.2 | a. 3.03, ddd (13.3, 6.6, 6.6) | 5a, 5b, 6-NH | 1’ |
| | | b. 2.97, ddd (13.0, 6.6, 6.6) | | 1’ |
| 6-NH | | 7.93[b] | 6a, 6b | |
| NH | | 7.61, br s | 2 | |
| 1’ | 161.0 | 7.97, s | | 6 |
| **L-Ile[4]** | | | | |
| 1 | 171.2 | - | | |
| 2 | 57.9 | 3.85, dd (6.4, 6.4) | 3, NH | 1, 6 |
| 3 | 35.7 | 1.43, m | 2, 6 | 5, 6 |
| 4 | 24.7 | a. 1.18, m | 5 | 5, 6 |
| | | b. 0.81 | | |
| 5 | 11.1 | 0.68, dd (7.2, 7.2) | 4 | |
| 6 | 14.8 | 0.44, d (6.6) | 3 | |
| NH | | 7.81[c] | 2 | |
| **D-Phe[5]** | | | | |
| 1 | 171.0 | | | |
| 2 | 54.6 | 4.38, ddd (11.7, 8.2, 3.4) | 3a, 3b, NH | 1 |
| 3 | 36.5 | a. 3.25, dd (14.3, 3.4) | 2 | |
| | | b. 2.68, dd (14.3, 11.7) | 2 | |
| 4 | 138.1 | - | | |
| 5/9 | 129.0 | 7.22, m | | 3, 7 |
| 6/8 | 128.1 | 7.24, m | 7 | 4 |
| 7 | 126.3 | 7.17, m | 6/8 | 5/9 |
| NH | | 8.43, d (8.3) | 2 | Ile[4]-1 |

| | | | | |
|---|---|---|---|---|
| **D-Leu$^6$** | | | | |
| 1 | 172.6 | - | | |
| 2 | 52.3 | 4.25, m | 3a, 3b, NH | 1, 3, 4 |
| 3 | 40.2 | a. 1.86, m | 2 | 5, 6 |
| | | b. 1.46, m | 2 | |
| 4 | 24.3 | 1.67, m | 5, 6 | 3, 5, 6 |
| 5 | 21.5 | 0.86, d (6.6) | 4 | 3, 4, 6 |
| 6 | 23.1 | 0.93, d (6.6) | 4 | 3, 4, 5 |
| NH | | 7.73, d (8.3) | 2 | Phe$^5$-1 |
| **L-Ile$^7$** | | | | |
| 1 | 169.9 | - | | |
| 2 | 57.4 | 4.08, dd (7.2, 7.2) | 3, NH | 1, Leu$^6$-1 |
| 3 | 35.8 | 1.77, m | 2, 4b, 6 | |
| 4 | 24.0 | a. 1.26, m | 4b, 5 | |
| | | b. 1.11, m | 3, 4a, 5 | |
| 5 | 11.2 | 0.79 | 4a, 4b | |
| 6 | 15.1 | 0.80[a] | 3 | |
| NH | | 7.12, d (7.2) | 2 | |
| **D-Ala$^8$** | | | | |
| 1 | 172.4 | - | | |
| 2 | 48.0 | 4.22, dd (6.6, 6.6) | 3, NH | 1 |
| 3 | 18.8 | 1.21, d (6.6) | 2 | 1, 2 |
| NH | | 7.81[c] | 2 | |

[a-c] Resonances with the same superscripts within a column are overlapped.

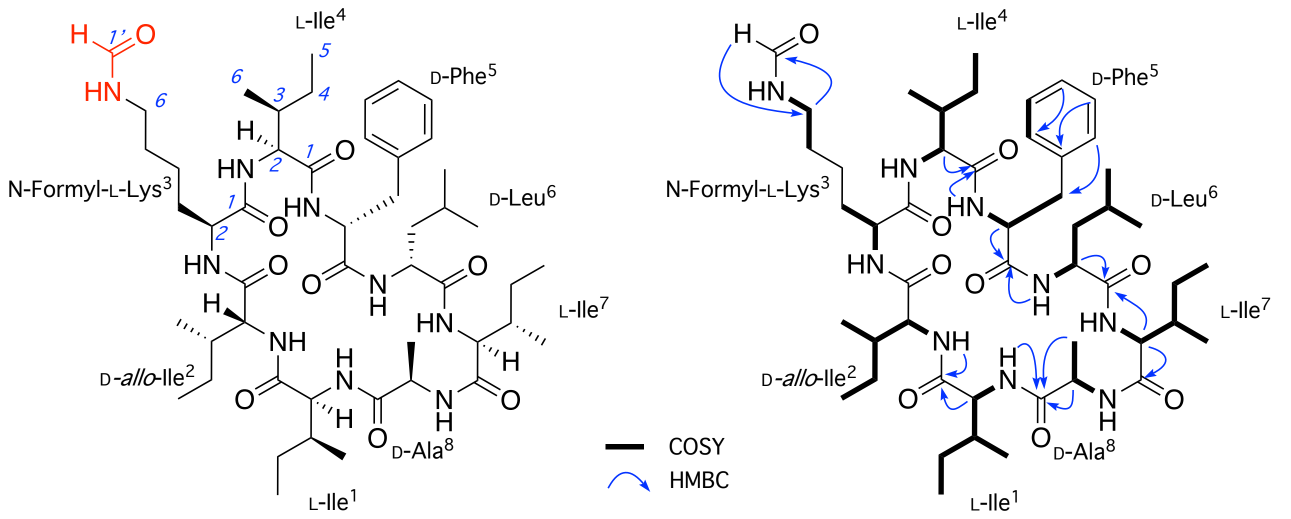


acyl-surugamide A1 (**5**)

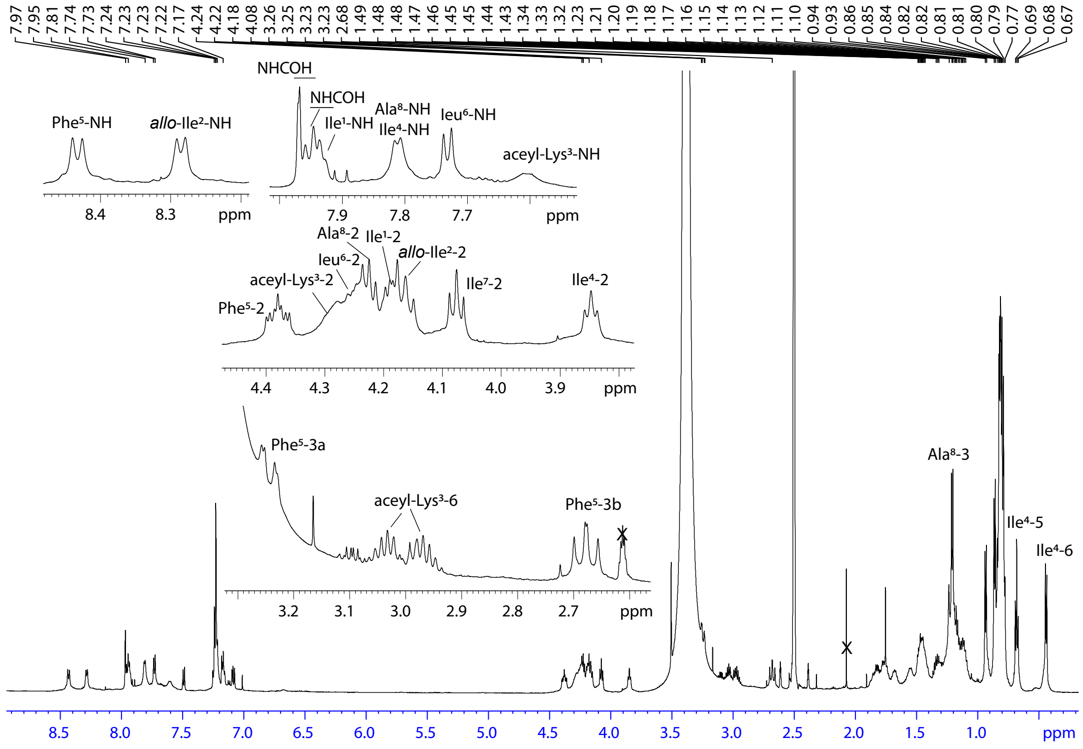


**Figure S19**. $^{1}$H NMR (600 MHz, DMSO-$d_6$) spectrum of acyl-surugamide A1 (**5**)

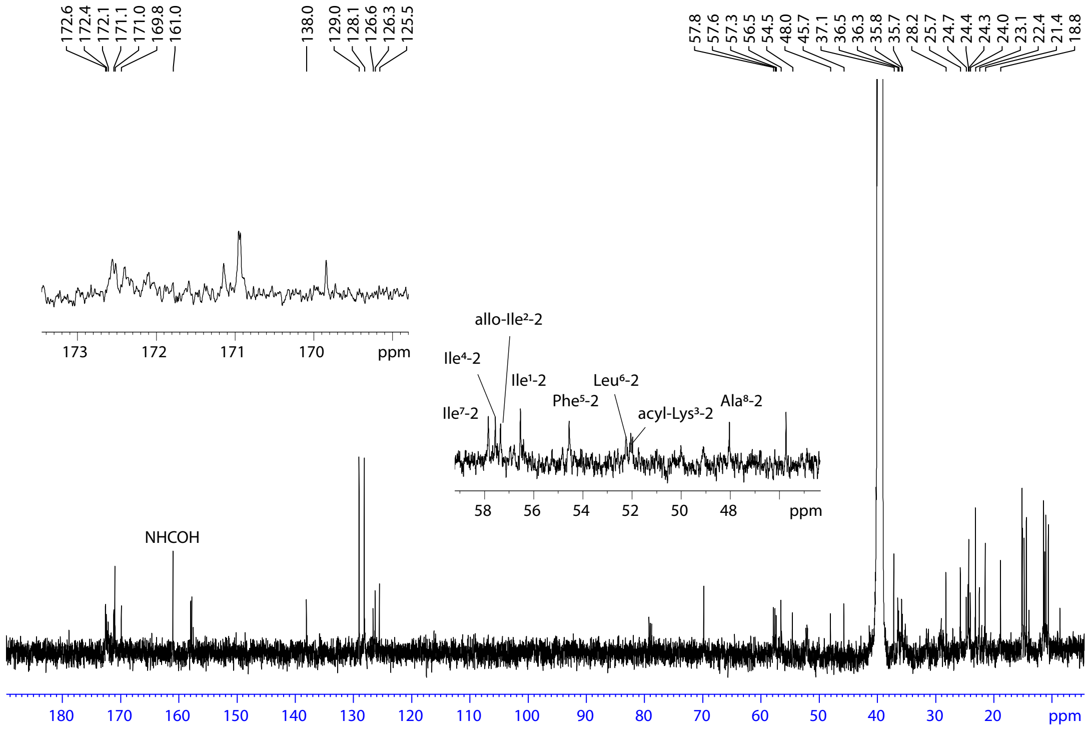


**Figure S20**. $^{13}$C NMR (150 MHz, DMSO-$d_6$) spectrum of acyl-surugamide A1 (**5**)

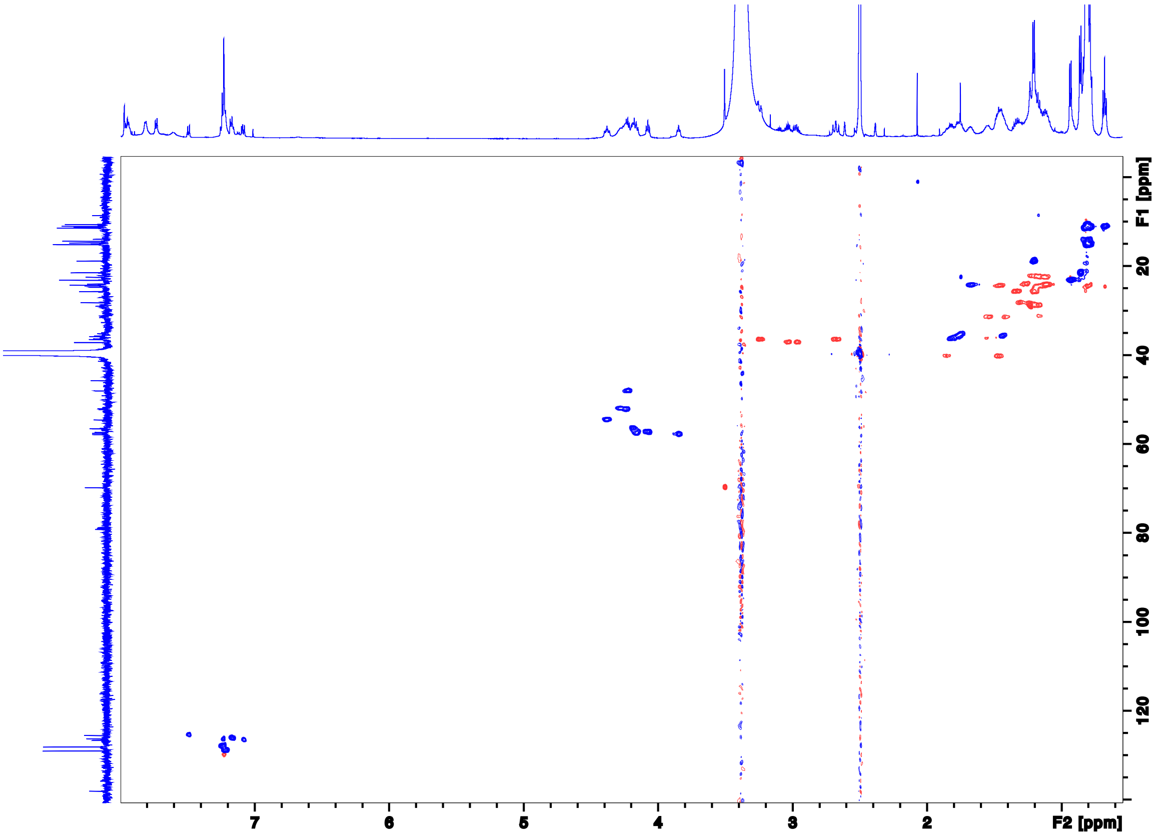


**Figure S21**. HSQC NMR (600 MHz, DMSO-$d_6$) spectrum of acyl-surugamide A1 (**5**)

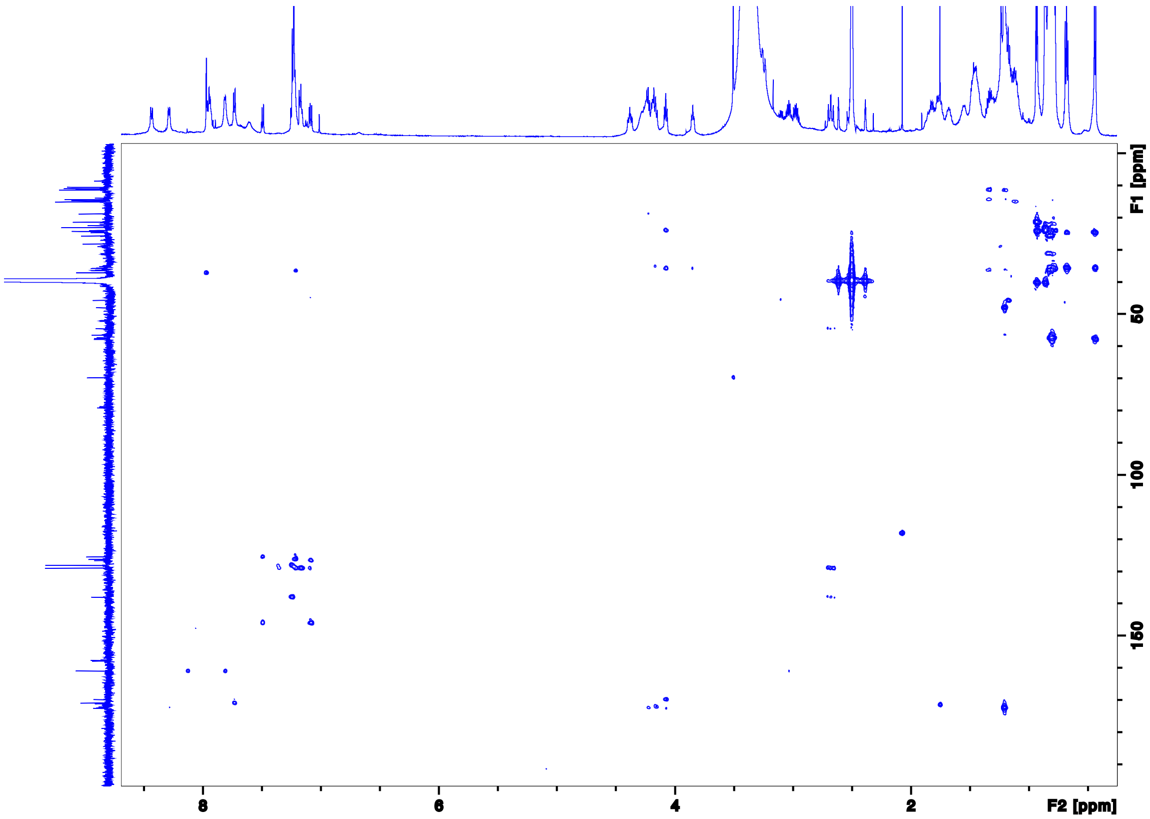


**Figure S22**. HMBC NMR (600 MHz, DMSO-$d_6$) spectrum of acyl-surugamide A1 (**5**)

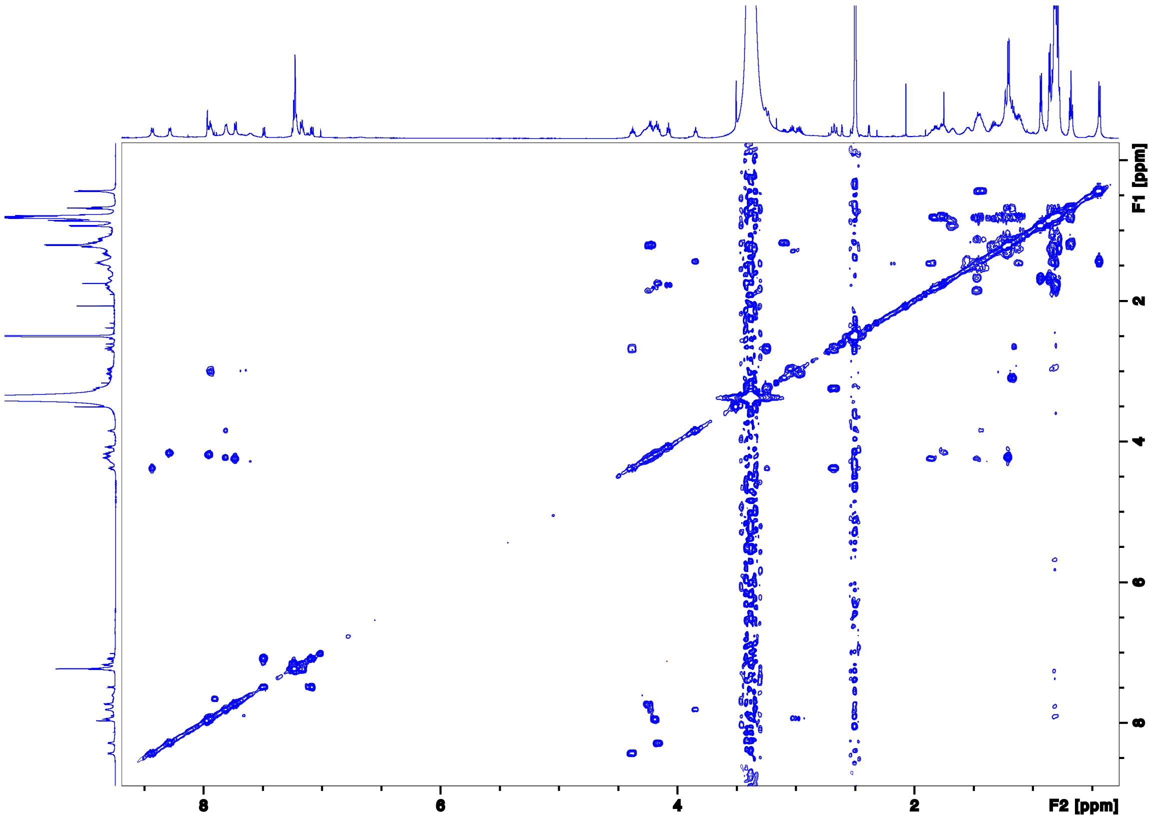


**Figure S23**. COSY NMR (600 MHz, DMSO-$d_6$) spectrum of acyl-surugamide A1 (**5**)

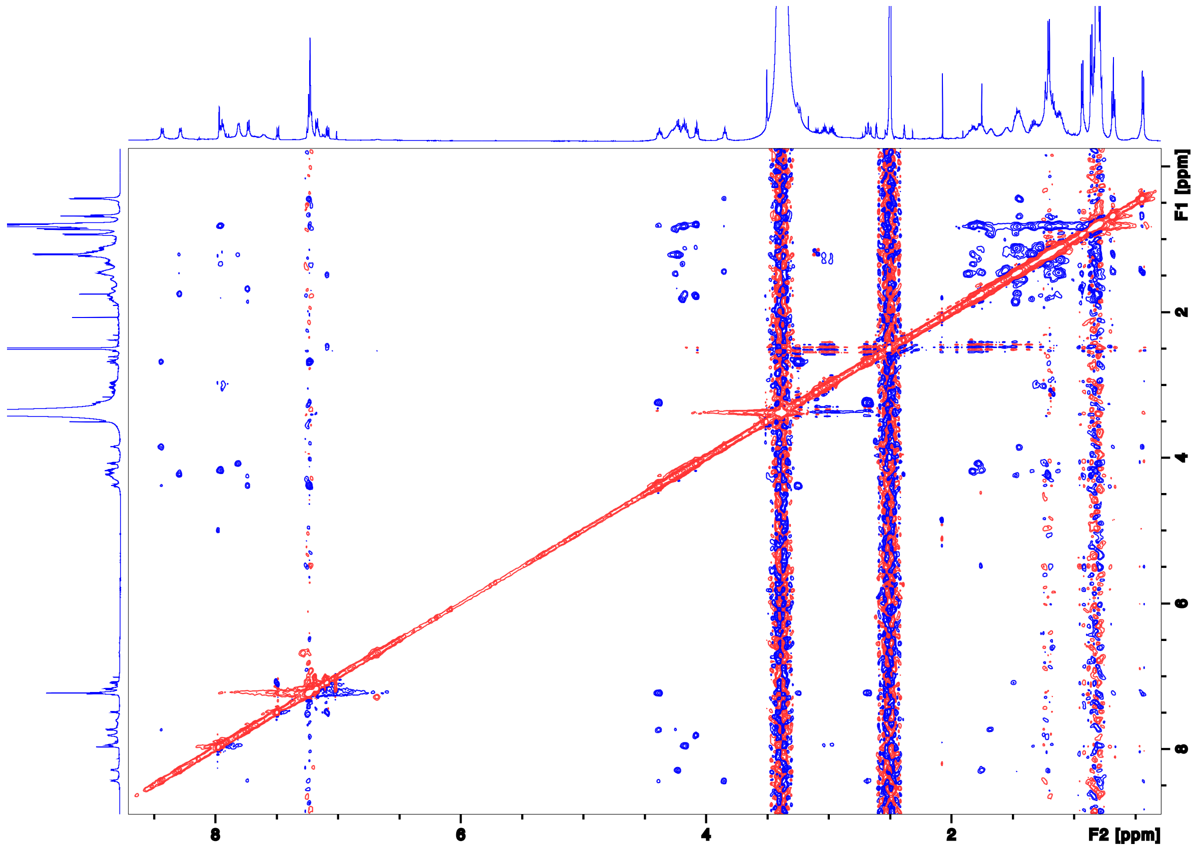


**Figure S24**. ROESY NMR (600 MHz, DMSO-$d_6$) spectrum of acyl-surugamide A1 (**5**)

# Mass Spectrum Molecular Formula Report

**Analysis Info**

| | | | |
|---|---|---|---|
| Analysis Name | D:\Data\Taizong\M0112_DCM_F17_19_V20-22.d | Acquisition Date | 10/25/2022 5:23:59 PM |
| Method | tune-medhigh_AP.m | Operator | a.salim |
| Sample Name | M0112_DCM_F17_19_V20-22 | Instrument / Ser# | micrOTOF 213750.00232 |
| Comment | | | |

**Acquisition Parameter**

| | | | | | |
|---|---|---|---|---|---|
| Source Type | ESI | Ion Polarity | Positive | Set Nebulizer | 0.5 Bar |
| Focus | Not active | | | Set Dry Heater | 180 °C |
| Scan Begin | 100 m/z | Set Capillary | 4500 V | Set Dry Gas | 5.0 l/min |
| Scan End | 1500 m/z | Set End Plate Offset | -500 V | Set Divert Valve | Source |

**Generate Molecular Formula Parameter**

| | | |
|---|---|---|
| Formula, min. | | |
| Formula, max. | | |
| Measured m/z | Tolerance | Charge |
| Check Valence | Minimum | Maximum |
| Nirogen Rule | Electron Configuration | |
| Filter H/C Ratio | Minimum | Maximum |
| Estimate Carbon | | |

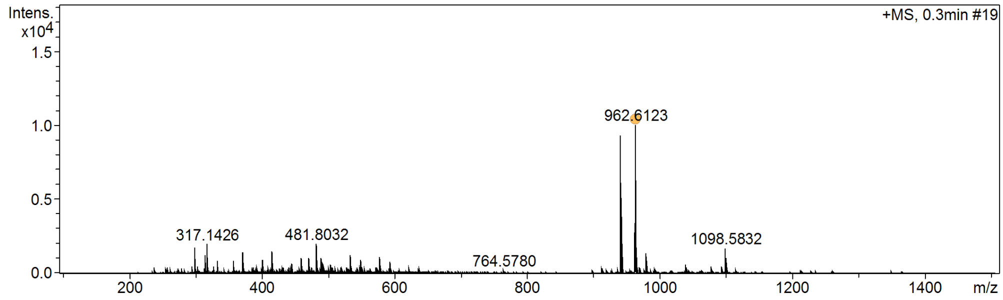


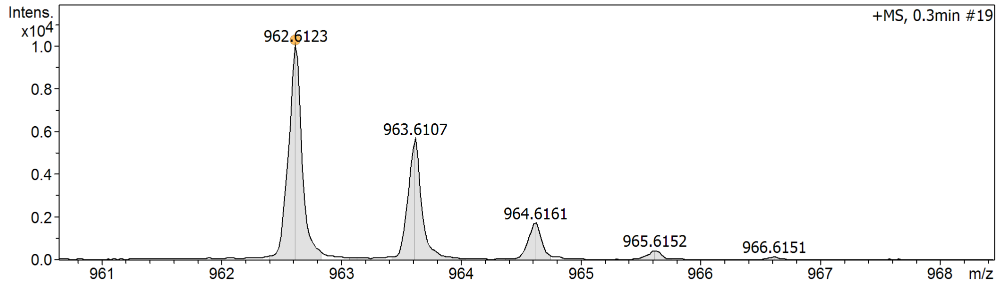


| Meas. m/z | # | Ion Formula | m/z | err [ppm] | mSigma | # Sigma | Score | rdb | e¯ Conf | N-Rule |
|---|---|---|---|---|---|---|---|---|---|---|
| 962.6123 | 1 | C49H81N9NaO9 | 962.6049 | 7.7 | 7.2 | 2 | 0.03 | 13.5 | even | ok |

Bruker Compass DataAnalysis 4.1 printed: 10/25/2022 5:35:59 PM Page 1 of 1

**Figure S25**. HRMS measurement for acyl-surugamide A1 (**5**)

**Table S7**. 1D and 2D NMR (600 MHz, DMSO-$d_6$) data for acyl-surugamide A2 (**6**)

| | $\delta_C$ | $\delta_H$, mult ($J$ in Hz) | COSY | HMBC |
|---|---|---|---|---|
| **L-Ile**[1] | | | | |
| 1 | 172.1 | - | | |
| 2 | 57.6 | 4.16, dd (8.0, 8.0) | 3, NH | 1, 3, 4 |
| 3 | 35.2 | 1.75, m | 2, 4b, 6 | |
| 4 | 24.4 | a. 1.46, m | 4b, 5 | |
| | | b. 1.13, m | 3, 4a | |
| 5 | 10.6 | 0.81[a] | 4a | |
| 6 | 15.07 | 0.80[a] | 3 | |
| NH | | 8.30, d (7.4) | 2 | Ala[8]-1 |
| **D-allo-Ile**[2] | | | | |
| 1 | 170.9[a] | - | | |
| 2 | 56.5 | 4.19, dd (7.6, 4.5) | 3, NH | 1, 3, 6 |
| 3 | 36.3 | 1.82, m | 2, 4a, 6 | |
| 4 | 25.7 | a. 1.34, m | 3, 4b, 5 | 5, 6 |
| | | b. 1.20, m | 4a | 5, 6 |
| 5 | 11.4 | 0.82[a] | 4a | |
| 6 | 14.4 | 0.81[a] | 3 | |
| NH | | 7.95, d (7.6) | 2 | Ile[1]-1 |
| **N-acetyl-L-Lys**[3] | | | | |
| 1 | 172.5 | - | | |
| 2 | 52.0 | 4.28, m | 3b, NH | |
| 3 | 31.6 | a. 1.54, m | 3b | |
| | | b. 1.41, m | 2 | |
| 4 | 22.5 | a. 1.19, m | 5 | |
| | | b. 1.12, m | | |
| 5 | 28.3 | a. 1.28, m | 6a, 6b | |
| | | b. 1.21, m | | |
| 6 | 38.6 | a. 3.01, ddd (13.3, 6.6, 6.6) | 5a, 6b, 6-NH | 1’ |
| | | b. 2.87, ddd (13.0, 6.6, 6.6) | 5a, 6a, 6-NH | 1’ |
| 6-NH | | 7.75, dd (6.0, 6.0) | 6a, 6b | 1’ |
| NH | | 7.60, br s | 2 | |
| 1’ | 169.0 | - | | |
| 2’ | 22.6 | 1.77, s | | 1’ |
| **L-Ile**[4] | | | | |
| 1 | 171.1 | - | | |
| 2 | 57.9 | 3.85, dd (6.4, 6.4) | 3, NH | 1, 3, 4, 6 |
| 3 | 35.7 | 1.43, m | 2, 4b, 6 | 5, 6 |
| 4 | 24.7 | a. 1.18, m | 5 | 5, 6 |
| | | b. 0.81[a] | 3, 5 | |
| 5 | 11.1 | 0.68, dd (7.3, 7.3) | 4a, 4b | |
| 6 | 14.7 | 0.44, d (6.7) | 3 | |
| NH | | 7.81[b] | 2 | |
| **D-Phe**[5] | | | | |
| 1 | 170.9[a] | | | |
| 2 | 54.6 | 4.38, ddd (11.7, 8.2, 3.4) | 3a, 3b, NH | 1 |
| 3 | 36.5 | a. 3.24, dd (14.3, 3.4) | 2 | |
| | | b. 2.68, dd (14.3, 11.7) | 2 | |
| 4 | 138.0 | - | | |
| 5/9 | 129.0 | 7.23, m | | 3, 7 |
| 6/8 | 128.1 | 7.24, m | 7 | 4 |
| 7 | 126.3 | 7.18, m | 6/8 | 5/9 |
| NH | | 8.43, d (8.3) | 2 | Ile[4]-1 |

| | | | | |
|---|---|---|---|---|
| **D-Leu$^{6}$** | | | | |
| 1 | 172.6 | - | | |
| 2 | 52.2 | 4.25, m | 3a, 3b, NH | |
| 3 | 40.2 | a. 1.86, m | 2, 3b | 5, 6 |
| | | b. 1.47, m | 2, 3a, 4 | |
| 4 | 24.3 | 1.68, m | 3b, 5, 6 | 3, 5, 6 |
| 5 | 21.4 | 0.86, d (6.5) | 4 | 3, 4, 6 |
| 6 | 23.1 | 0.93, d (6.5) | 4 | 3, 4, 5 |
| NH | | 7.73, d (7.4) | 2 | Phe$^{5}$-1 |
| **L-Ile$^{7}$** | | | | |
| 1 | 169.9 | - | | |
| 2 | 57.3 | 4.08, dd (7.2, 7.2) | 3, NH | 1, 3, 4, 6, Leu$^{6}$-1 |
| 3 | 35.8 | 1.77, m | 2, 4b, 6 | |
| 4 | 24.0 | a. 1.27, m | 5 | |
| | | b. 1.11, m | 3, 5 | |
| 5 | 11.2 | 0.79, t (7.5) | 4a, 4b | |
| 6 | 15.12 | 0.81[a] | 3 | |
| NH | | 7.12, d (7.2) | 2 | |
| **D-Ala$^{8}$** | | | | |
| 1 | 172.4 | - | | |
| 2 | 48.1 | 4.23, dd (6.6, 6.6) | 3, NH | 1 |
| 3 | 18.8 | 1.21, d (6.6) | 2 | 1, 2 |
| NH | | 7.81[b] | 2 | Ile$^{7}$-1 |

[a-b] Resonances with the same superscripts within a column are overlapped.

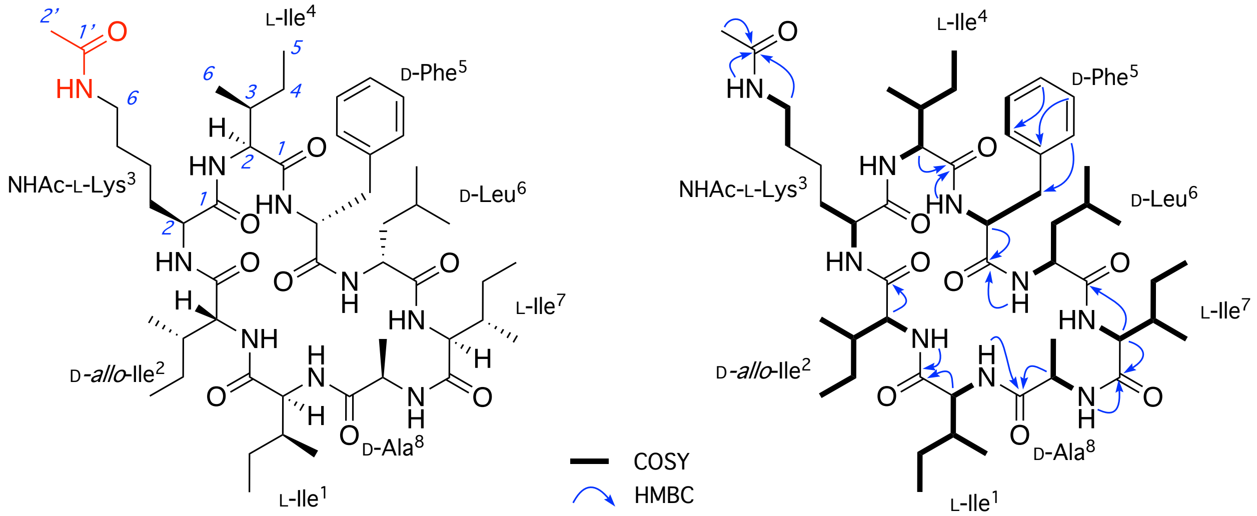


acyl-surugamide A2 (**6**)

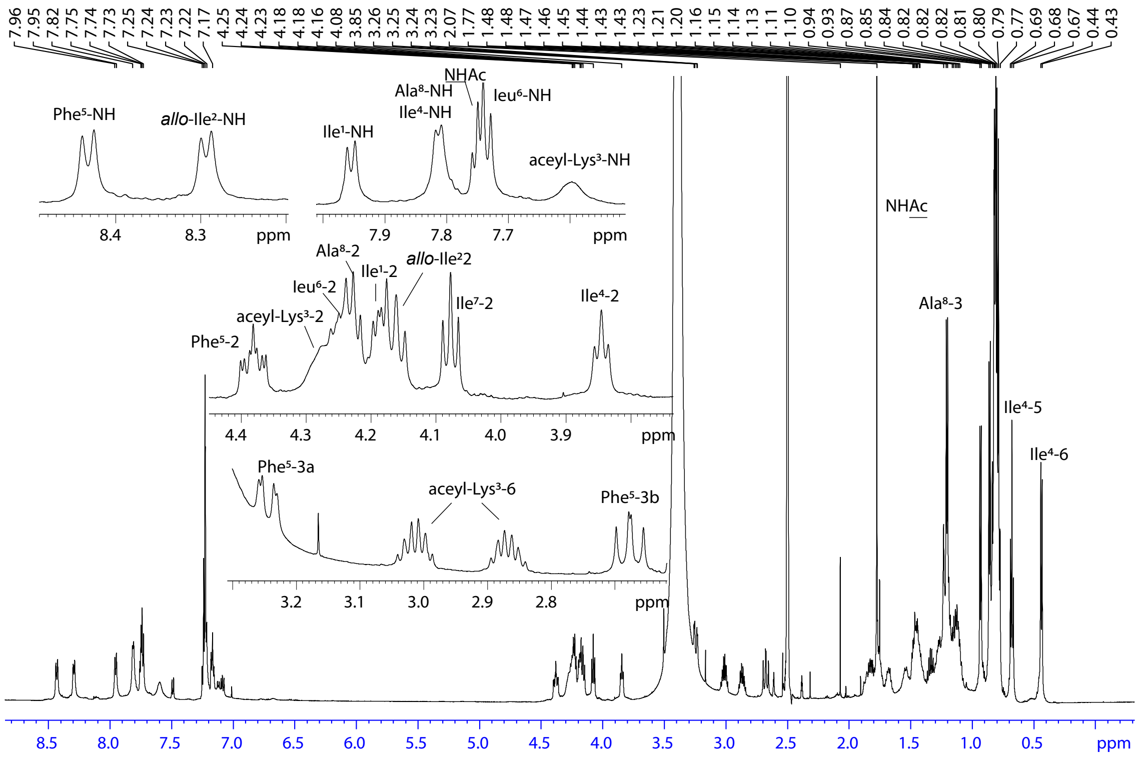


**Figure S26**. $^{1}$H NMR (600 MHz, DMSO-$d_6$) spectrum of acyl-surugamide A2 (**6**)

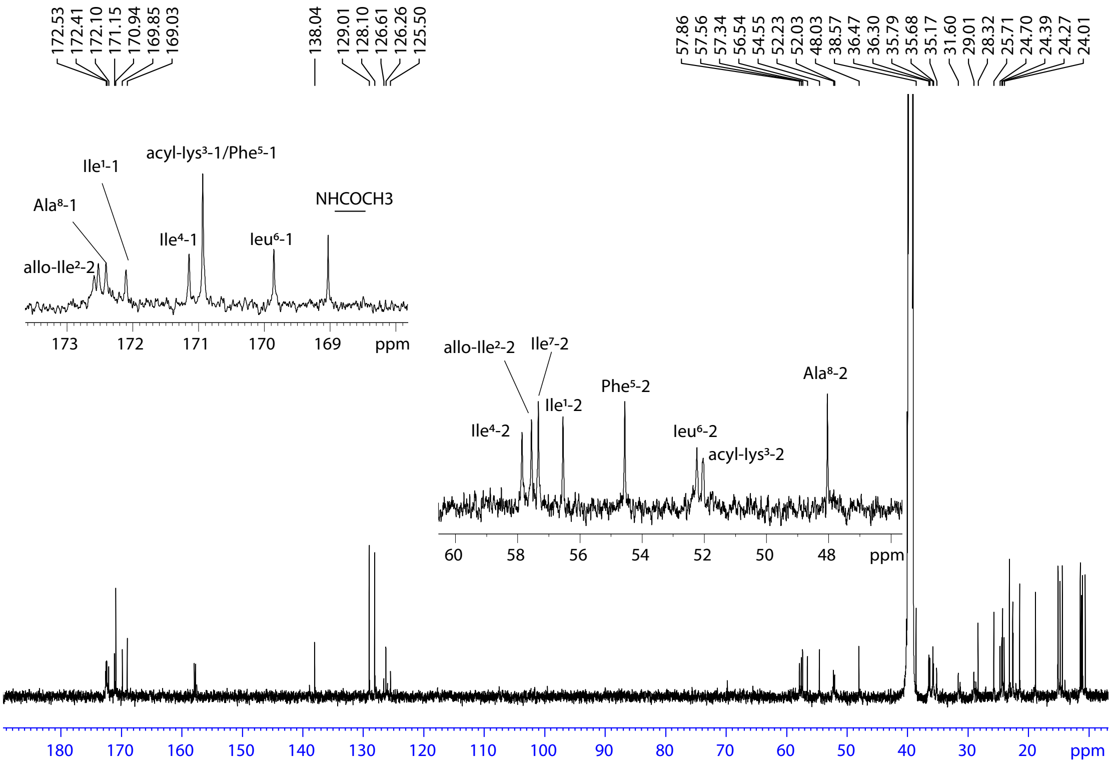


**Figure S27**. $^{13}$C NMR (150 MHz, DMSO-$d_6$) spectrum of acyl-surugamide A2 (**6**)

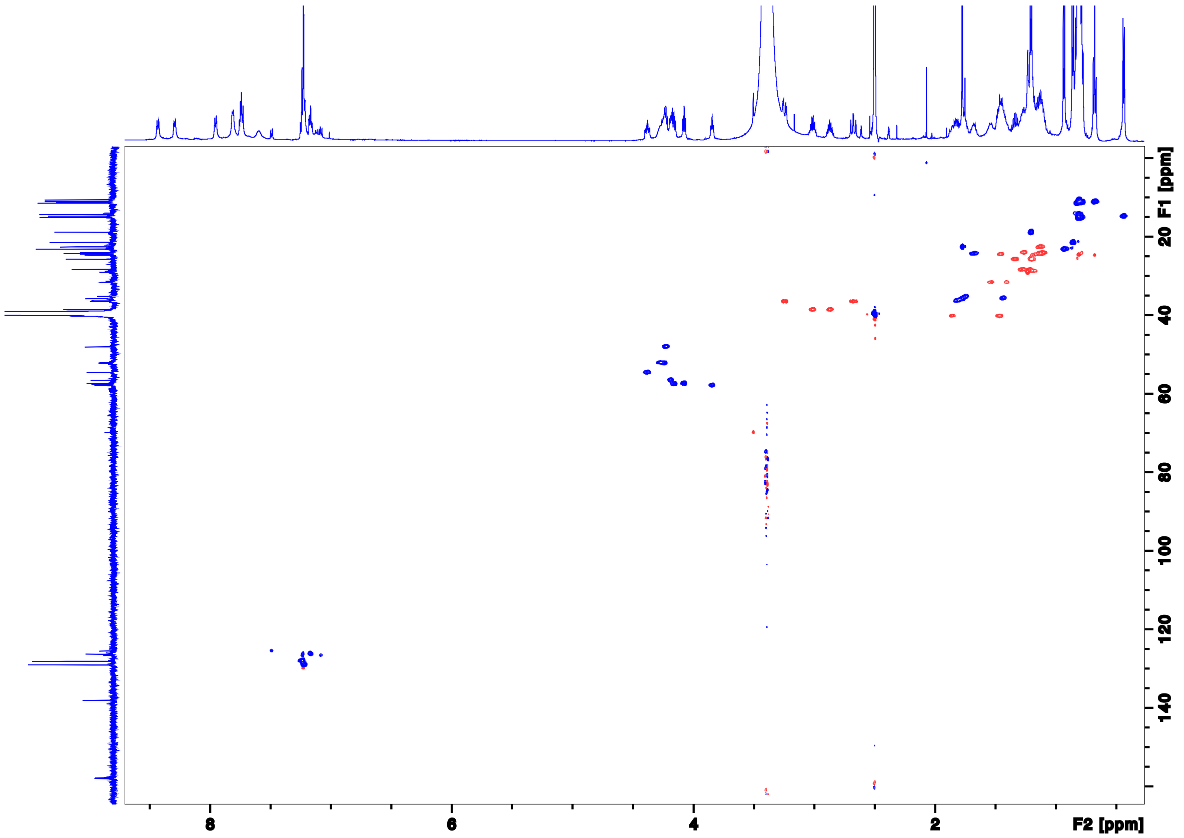


**Figure S28**. HSQC NMR (DMSO-$d_6$) spectrum of acyl-surugamide A2 (**6**)

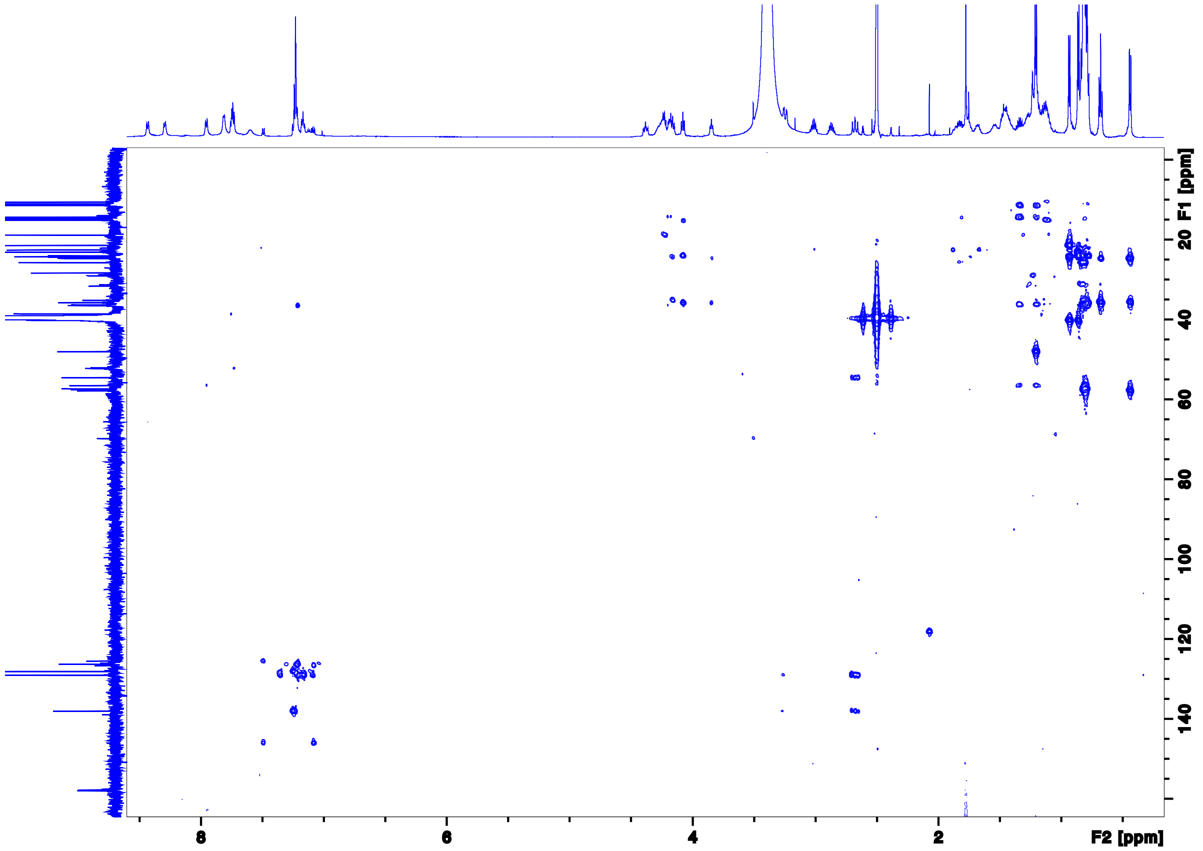


**Figure S29**. HMBC NMR (DMSO-$d_6$) spectrum of acyl-surugamide A2 (**6**)

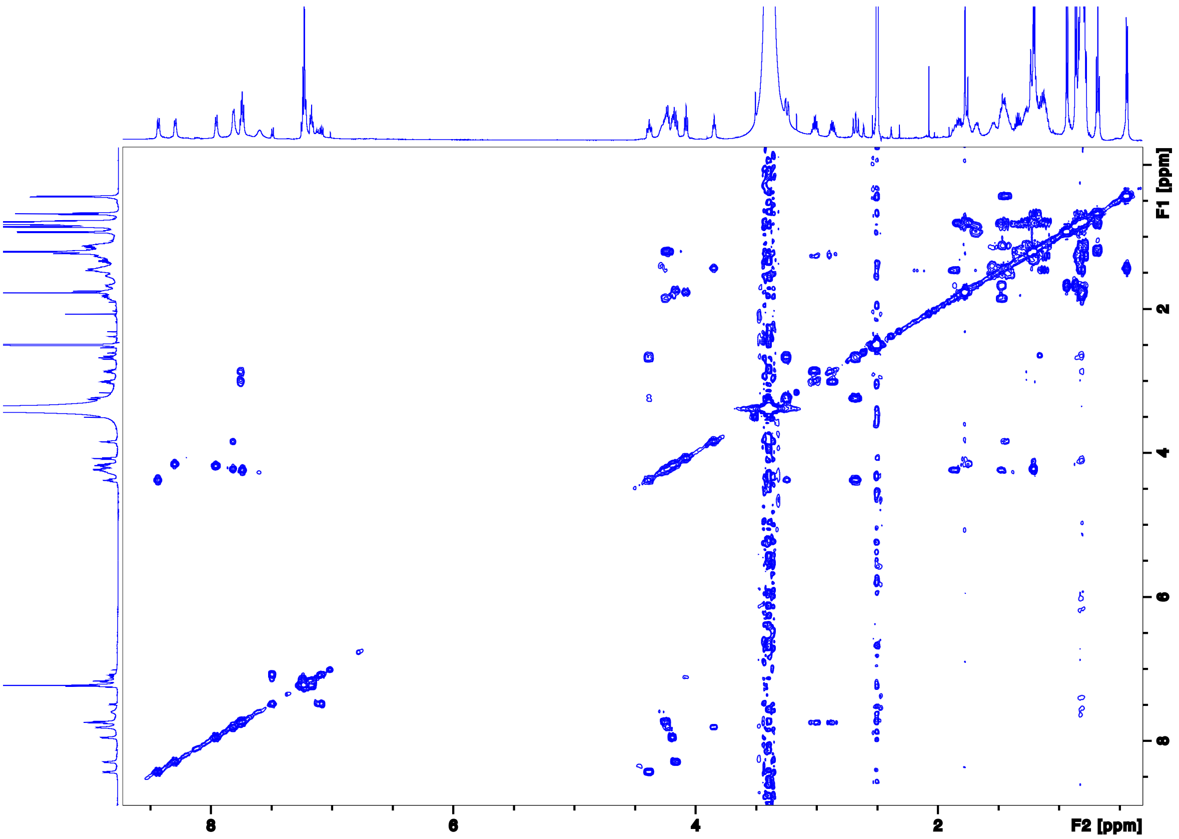


**Figure S30**. COSY NMR (DMSO-$d_6$) spectrum of acyl-surugamide A2 (**6**)

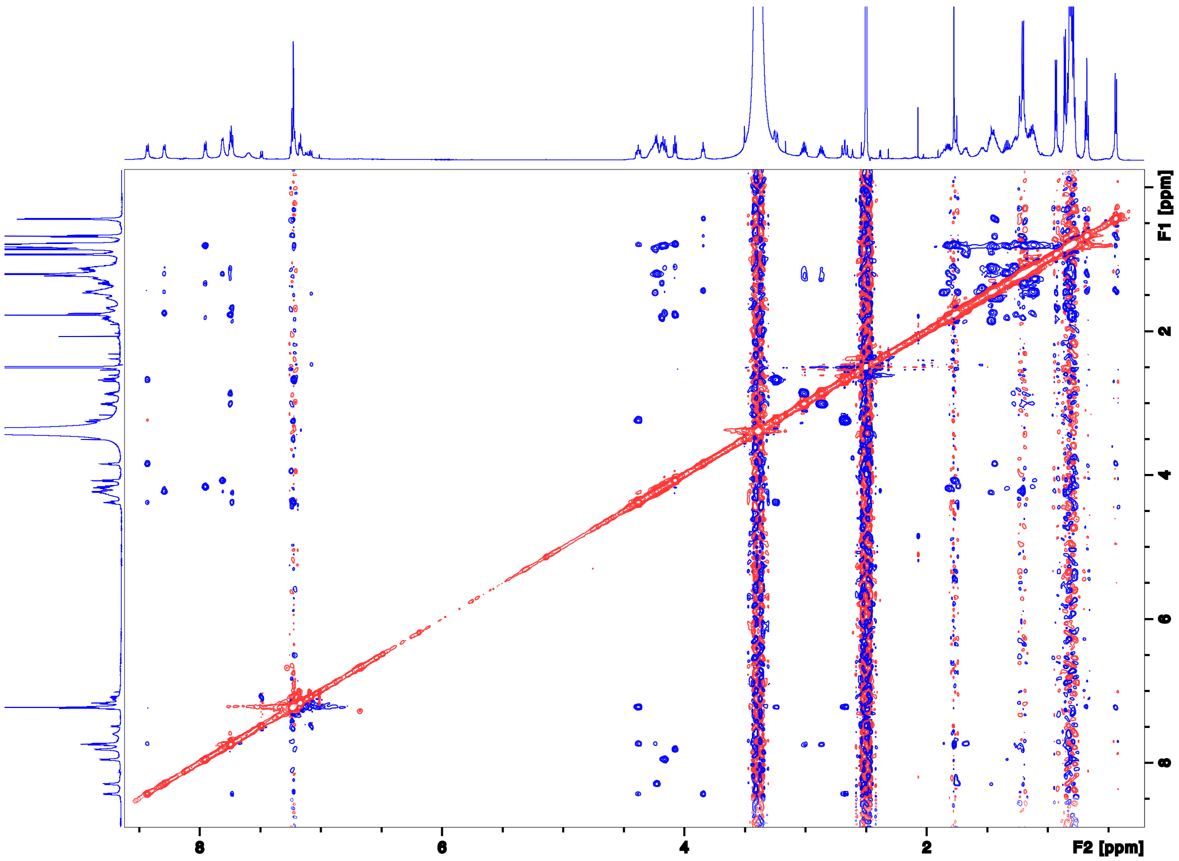


**Figure S31**. ROESY NMR (DMSO-$d_6$) spectrum of acyl-surugamide A2 (**6**)

## Mass Spectrum Molecular Formula Report

**Analysis Info**

| | | | |
|---|---|---|---|
| Analysis Name | D:\Data\Taizong\M0112_DCM_F17_19_V23-25.d | Acquisition Date | 10/25/2022 5:26:39 PM |
| Method | tune-medhigh_AP.m | Operator | a.salim |
| Sample Name | M0112_DCM_F17_19_V23-25 | Instrument / Ser# | micrOTOF 213750.00232 |
| Comment | | | |

**Acquisition Parameter**

| | | | | | |
|---|---|---|---|---|---|
| Source Type | ESI | Ion Polarity | Positive | Set Nebulizer | 0.5 Bar |
| Focus | Not active | | | Set Dry Heater | 180 °C |
| Scan Begin | 100 m/z | Set Capillary | 4500 V | Set Dry Gas | 5.0 l/min |
| Scan End | 1500 m/z | Set End Plate Offset | -500 V | Set Divert Valve | Source |

**Generate Molecular Formula Parameter**

| | | |
|---|---|---|
| Formula, min. | | |
| Formula, max. | | |
| Measured m/z | Tolerance | Charge |
| Check Valence | Minimum | Maximum |
| Nirogen Rule | Electron Configuration | |
| Filter H/C Ratio | Minimum | Maximum |
| Estimate Carbon | | |

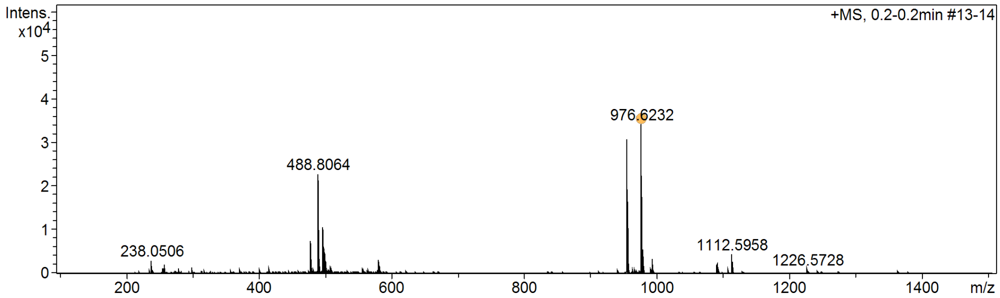


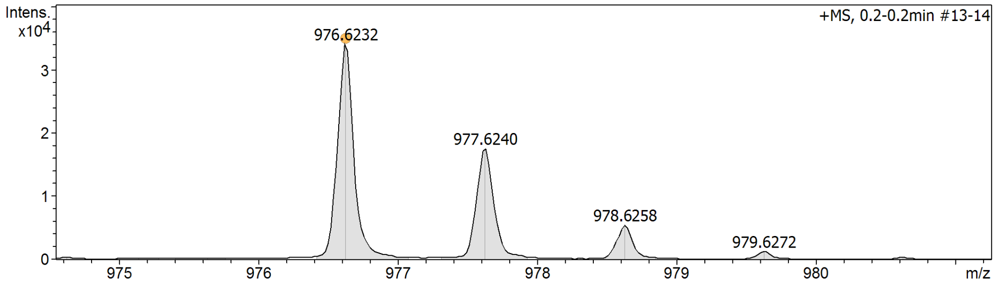


| Meas. m/z | # | Ion Formula | m/z | err [ppm] | mSigma | # Sigma | Score | rdb | e⁻ Conf | N-Rule |
|---|---|---|---|---|---|---|---|---|---|---|
| 976.6232 | 1 | C50H83N9NaO9 | 976.6206 | 2.6 | 34.7 | 5 | 15.85 | 13.5 | even | ok |

Bruker Compass DataAnalysis 4.1 printed: 10/25/2022 5:32:27 PM Page 1 of 1

**Figure S32**. HRMS measurement for acyl-surugamide A2 (**6**)

**Table S8**. 1D and 2D NMR (600 MHz, DMSO-$d_6$) data for acyl-surugamide A3 (**7**)*

| | $\delta_C$ | $\delta_H$, mult ($J$ in Hz) | COSY | HMBC |
|---|---|---|---|---|
| **L-Ile[1]** | | | | |
| 1 | 172.1 | - | | |
| 2 | 57.6 | 4.16, dd (8.2, 7.8) | 3, NH | 1 |
| 3 | 35.2 | 1.75, m | 2, 4b, 6 | |
| 4 | 24.4 | a. 1.46, m | 4b | |
| | | b. 1.13, m | 3, 4a | |
| 5 | 10.6 | 0.81[a] | | 3, 4 |
| 6 | 15.1[a] | 0.81[a] | 3 | 2, 3, 4 |
| NH | | 8.30, d (7.3) | 2 | Ala[8]-1 |
| **D-allo-Ile[2]** | | | | |
| 1 | 171.0[b] | - | | |
| 2 | 56.6 | 4.19, dd (7.6, 4.5) | 3, NH | 1 |
| 3 | 36.3 | 1.81, m | 2, 4a, 6 | |
| 4 | 25.7 | a. 1.34, m | 3, 4b, 5 | |
| | | b. 1.20, m | 4a | |
| 5 | 11.4 | 0.83[a] | 4a | 3, 4 |
| 6 | 14.4 | 0.80[b] | 3 | 2, 3, 4 |
| NH | | 7.94, d (7.6) | 2 | Ile[1]-1 |
| **N-propionyl-L-Lys[3]** | | | | |
| 1 | 172.6 | - | | |
| 2 | 52.1 | 4.27, m | 3b, NH | |
| 3 | 31.6 | a. 1.55, m | 3b | |
| | | b. 1.41, m | 2, 3a | |
| 4 | 22.6 | 1.13, m, 2H | 5 | |
| 5 | 28.4 | a. 1.28, m | 6a, 6b | |
| | | b. 1.23, m | | |
| 6 | 38.5 | a. 3.03, m | 5a, 6b, 6-NH | 1’ |
| | | b. 2.87, m | 5a, 6a, 6-NH | 1’ |
| 6-NH | | 7.69, dd (5.5, 5.5) | 6a, 6b | 1’ |
| NH | | 7.60, br s | 2 | |
| 1’ | 172.8 | - | | |
| 2’ | 28.5 | 2.04, q (7.6) | 3’ | 1’ |
| 3’ | 9.96 | 0.97, t (7.6) | 2’ | 1’, 2’ |
| **L-Ile[4]** | | | | |
| 1 | 171.1 | - | | |
| 2 | 57.9 | 3.84, dd (6.5, 6.3) | 3, NH | 1 |
| 3 | 35.7 | 1.44, m | 2, 4b, 6 | |
| 4 | 24.7 | a. 1.20, m | 5 | |
| | | b. 0.81[a] | 3, 5 | |
| 5 | 11.0 | 0.68, dd (7.3, 7.3) | 4a, 4b | 3, 4 |
| 6 | 14.8 | 0.43, d (6.5) | 3 | 2, 3, 4 |
| NH | | 7.82[c] | 2 | |
| **D-Phe[5]** | | | | |
| 1 | 171.0[b] | | | |
| 2 | 54.5 | 4.38, ddd (11.7, 8.3, 3.4) | 3a, 3b, NH | 1 |
| 3 | 36.5 | a. 3.25, dd (14.0, 3.4) | 2 | |
| | | b. 2.67, dd (14.0, 11.7) | 2 | |
| 4 | 138.0 | - | | |
| 5/9 | 129.0 | 7.22, m | | 3, 7 |
| 6/8 | 128.1 | 7.24, m | 7 | 4 |
| 7 | 126.3 | 7.17, m | 6/8 | 5/9 |

| | | | | |
|---|---|---|---|---|
| NH | | 8.43, d (8.3) | 2 | 1, Ile$^{4}$-1 |
| **D-Leu$^{6}$** | | | | |
| 1 | 172.7 | - | | |
| 2 | 52.3 | 4.25, m | 3a, 3b, NH | 1 |
| 3 | 40.2 | a. 1.85, m | 2, 3b | 5, 6 |
| | | b. 1.47, m | 2, 3a, 4 | |
| 4 | 24.3 | 0.86, m | 3b, 5, 6 | 3, 5, 6 |
| 5 | 21.4 | 0.86, d (6.5) | 4 | 3, 4, 6 |
| 6 | 23.1 | 0.93, d (6.5) | 4 | 3, 4, 5 |
| NH | | 7.74, d (7.5) | 2 | Phe$^{5}$-1 |
| **L-Ile$^{7}$** | | | | |
| 1 | 170.0 | - | | |
| 2 | 57.3 | 4.08, dd (7.2, 7.2) | 3, NH | 1, Leu$^{6}$-1 |
| 3 | 35.9 | 1.77, m | 2, 4b, 6 | |
| 4 | 24.0 | a. 1.26, m | 5 | |
| | | b. 1.11, m | 3, 5 | |
| 5 | 11.3 | 0.78 | 4a, 4b | |
| 6 | 15.1[a] | 0.80[b] | 3 | |
| NH | | 7.22, br s | 2 | |
| **D-Ala$^{8}$** | | | | |
| 1 | 172.5 | - | | |
| 2 | 48.1 | 4.22, dd (7.0, 6.7) | 3, NH | 1 |
| 3 | 18.8 | 1.20, d (6.8) | 2 | 1 |
| NH | | 7.83[c] | 2 | Ile$^{7}$-1 |

[a-c] Resonances with the same superscripts within a column are overlapped. * NMR data was obtained from the semi-synthetic sample of **7**.

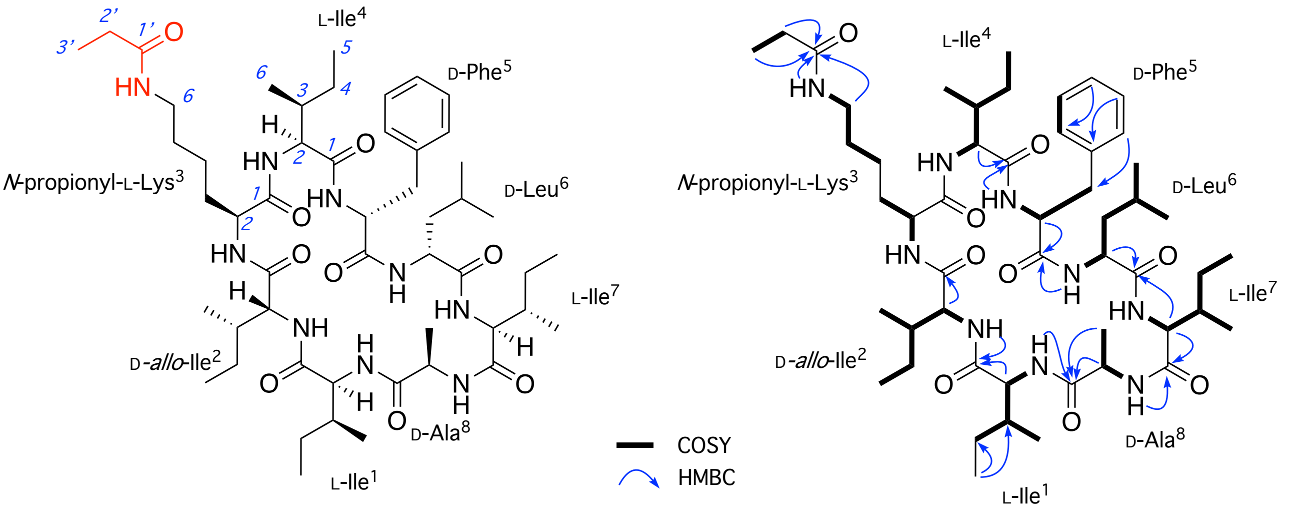


acyl-surugamide A3 (**7**)

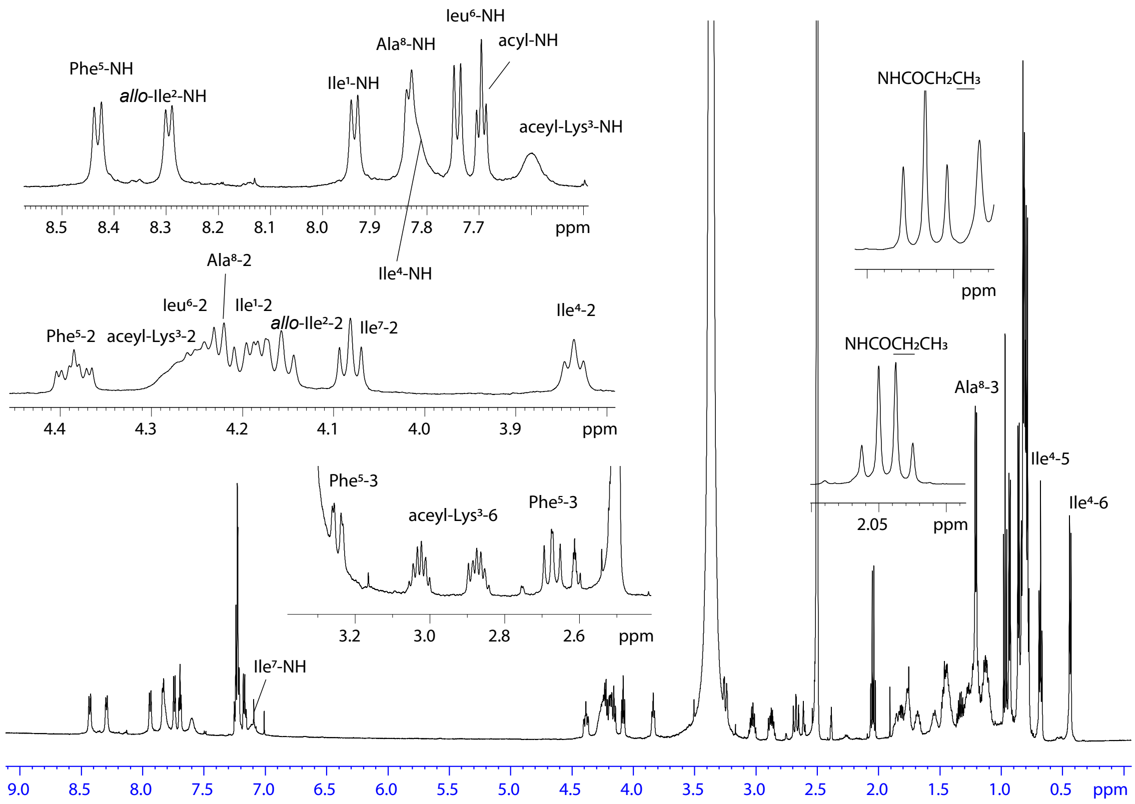


**Figure S33**. $^{1}$H NMR (600 MHz, DMSO-$d_6$) spectrum of acyl-surugamide A3 (**7**)

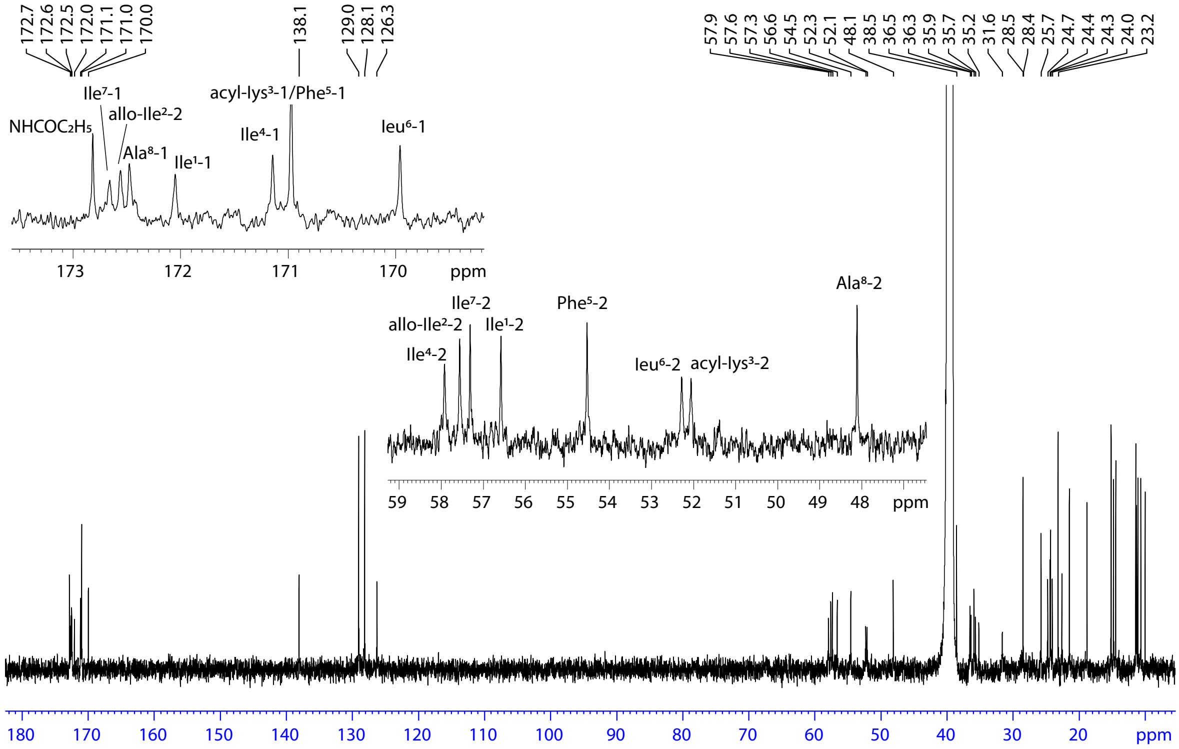


**Figure S34**. $^{13}$C NMR (150 MHz, DMSO-$d_6$) spectrum of acyl-surugamide A3 (**7**)

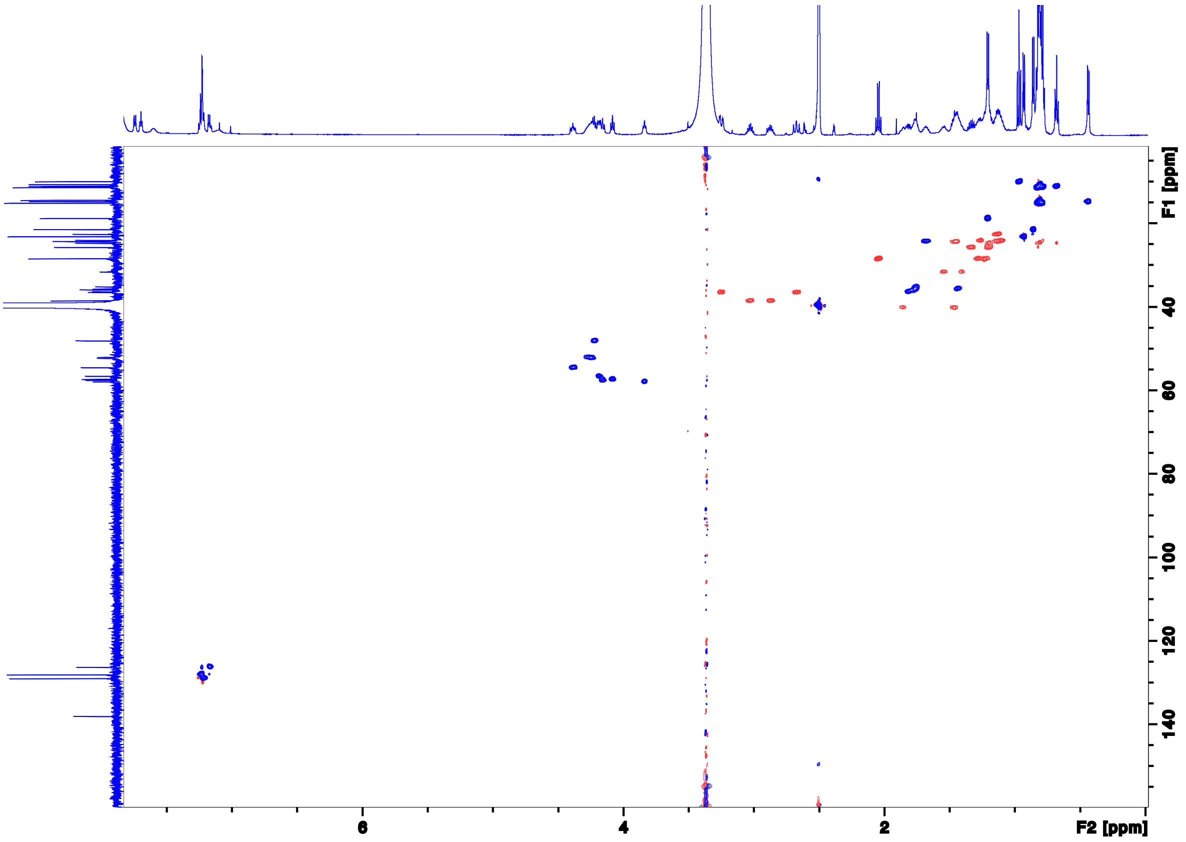


**Figure S35**. HSQC NMR (600 MHz, DMSO-$d_6$) spectrum of acyl-surugamide A3 (**7**)

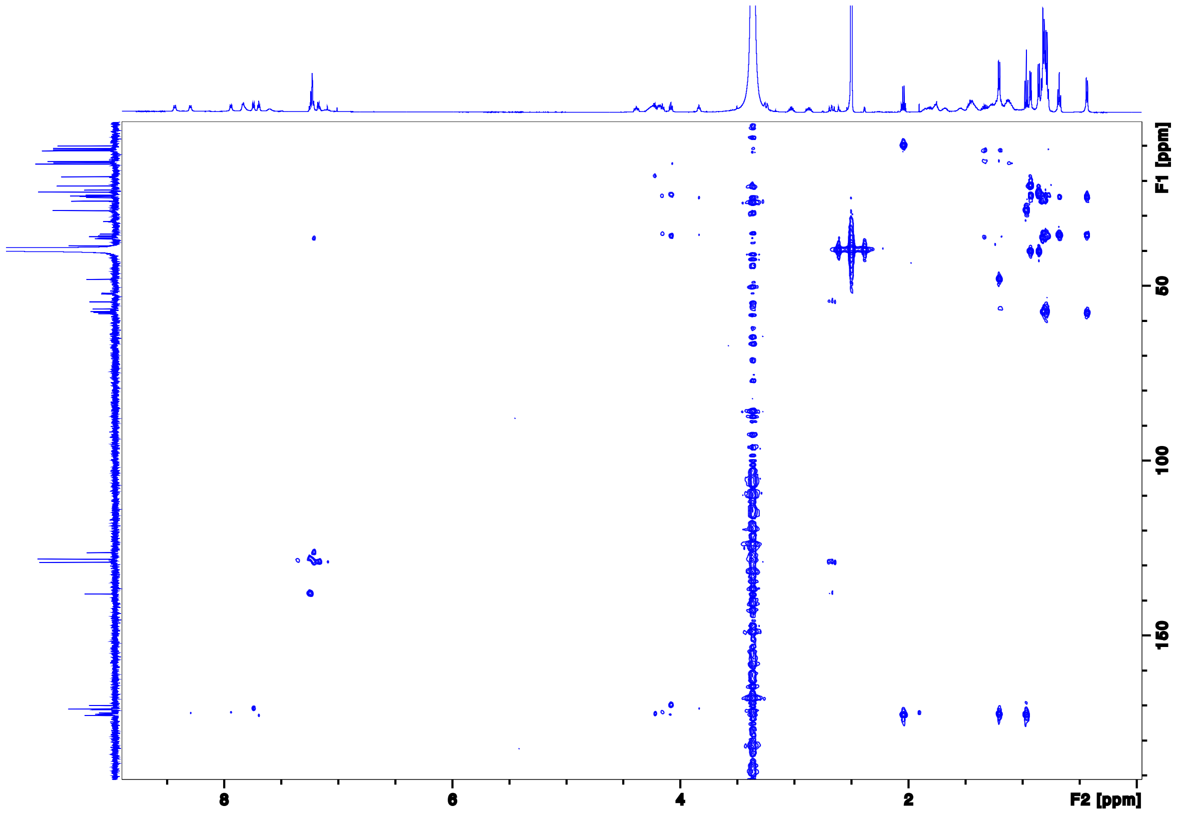


**Figure S36**. HMBC NMR (600 MHz, DMSO-$d_6$) spectrum of acyl-surugamide A3 (**7**)

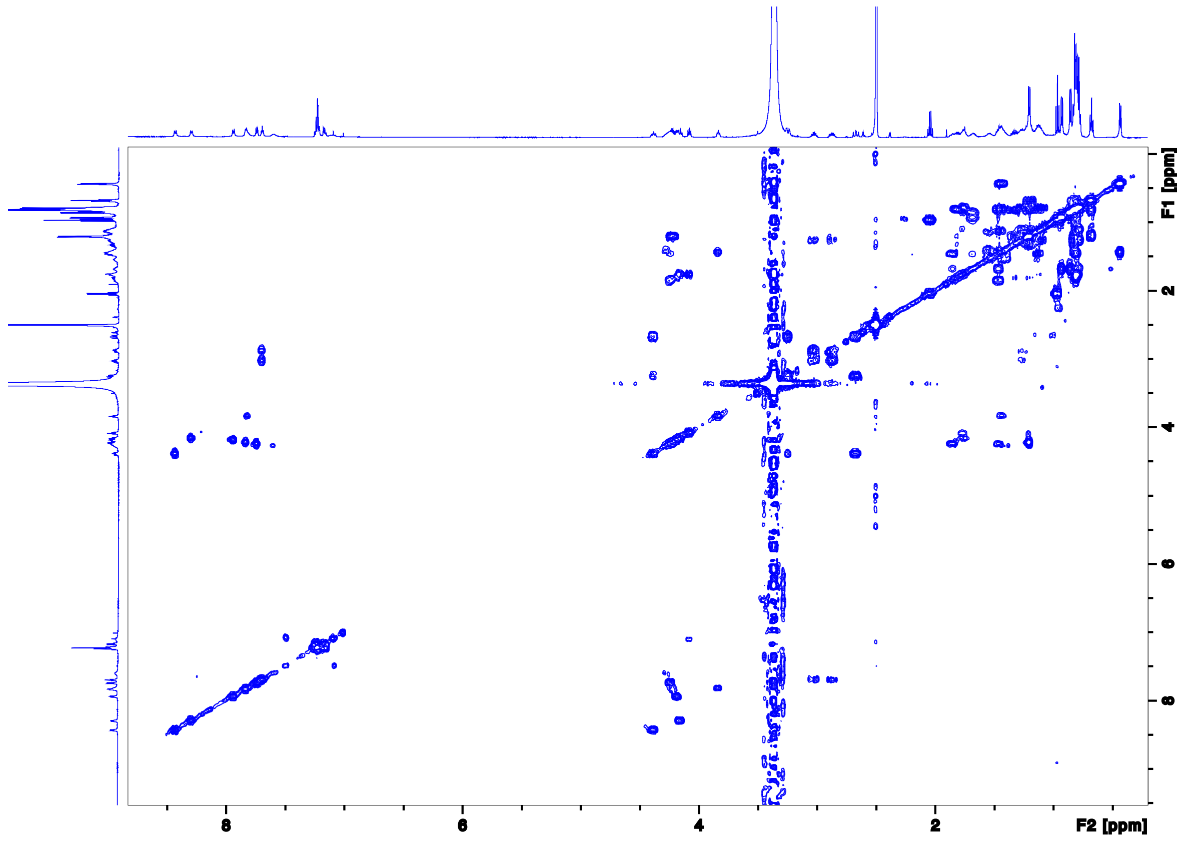


**Figure S37**. COSY NMR (600 MHz, DMSO-$d_6$) spectrum of acyl-surugamide A3 (**7**)

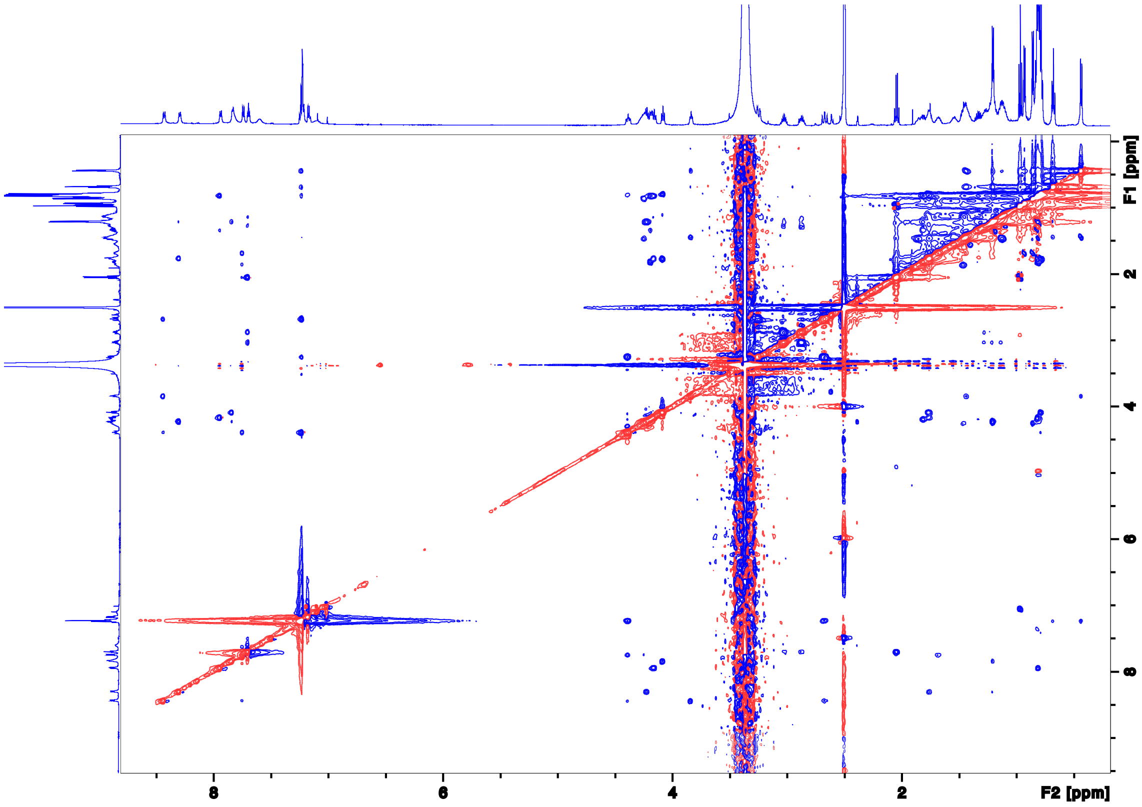


**Figure S38**. ROESY NMR (600 MHz, DMSO-$d_6$) spectrum of acyl-surugamide A3 (**7**)

# Mass Spectrum Molecular Formula Report

**Analysis Info**

| | | | | |
|---|---|---|---|---|
| Analysis Name | D:\Data\Taizong\CMB_M0112_F17_19_V27_28_2000001.d | Acquisition Date | 11/3/2022 1:21:38 PM | |
| Method | tune-medhigh_AP.m | Operator | a.salim | |
| Sample Name | CMB_M0112_F17_19_V27_28_2 | Instrument / Ser# | micrOTOF | 213750.00232 |
| Comment | | | | |

**Acquisition Parameter**

| | | | | | |
|---|---|---|---|---|---|
| Source Type | ESI | Ion Polarity | Positive | Set Nebulizer | 0.5 Bar |
| Focus | Not active | | | Set Dry Heater | 180 °C |
| Scan Begin | 100 m/z | Set Capillary | 4500 V | Set Dry Gas | 5.0 l/min |
| Scan End | 1500 m/z | Set End Plate Offset | -500 V | Set Divert Valve | Source |

**Generate Molecular Formula Parameter**

Formula, min.
Formula, max.
Measured m/z — Tolerance — Charge
Check Valence — Minimum — Maximum
Nirogen Rule — Electron Configuration
Filter H/C Ratio — Minimum — Maximum
Estimate Carbon

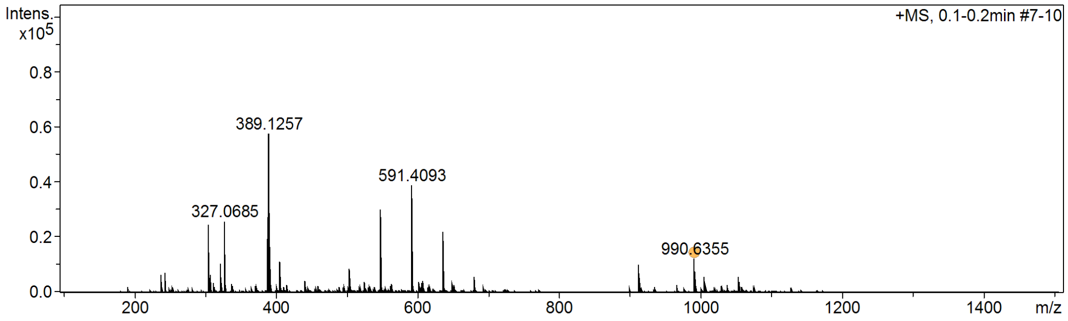


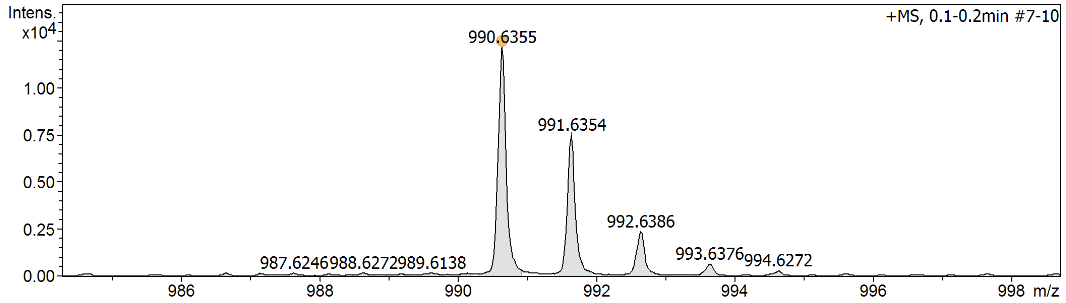


| Meas. m/z | # | Ion Formula | m/z | err [ppm] | mSigma | # Sigma | Score | rdb | e⁻ Conf | N-Rule |
|---|---|---|---|---|---|---|---|---|---|---|
| 990.6355 | 1 | C51H85N9NaO9 | 990.6362 | -0.8 | 11.2 | 1 | 100.00 | 13.5 | even | ok |

Bruker Compass DataAnalysis 4.1 printed: 3/21/2023 10:37:54 AM Page 1 of 1

**Figure S39**. HRMS measurement for acyl-surugamide A3 (**7**)

**Table S9**. 1D and 2D NMR (600 MHz, DMSO-$d_6$) data for acyl-surugamide A4 (**8**)

| | $\delta_C$ | $\delta_H$, mult ($J$ in Hz) | COSY | HMBC |
|---|---|---|---|---|
| **L-Ile[1]** | | | | |
| 1 | n.d. | - | | |
| 2 | 57.4 | 4.21, dd (8.3, 8.3) | 3, NH | |
| 3 | 35.6 | 1.75, m | 2, 4b, 6 | |
| 4 | 24.4 | a. 1.45, m | 4b, 5 | |
| | | b. 1.10, m | 3, 4a | |
| 5 | 10.6 | 0.81[a] | 4a | |
| 6 | 15.1[a] | 0.79[b] | 3 | |
| NH | | 8.24, d (8.3) | 2 | |
| **D-allo-Ile[2]** | | | | |
| 1 | n.d. | - | | |
| 2 | 56.4 | 4.21, dd (7.6, 4.5) | 3, NH | |
| 3 | 36.5 | 1.80, m | 2, 4a, 6 | |
| 4 | 25.7 | a. 1.33, m | 3, 4b, 5 | |
| | | b. 1.19, m | 4a | |
| 5 | 11.4 | 0.82[a] | 4a | |
| 6 | 14.4 | 0.80[b] | 3 | |
| NH | | 7.95, d (7.6) | 2 | |
| **N-pyruvoyl-L-Lys[3]** | | | | |
| 1 | n.d. | - | | |
| 2 | 52.0 | 4.29, m | 3b, NH | |
| 3 | 31.5 | a. 1.55, m | 3b | |
| | | b. 1.42, m | 2, 3a | |
| 4 | 22.5 | a. 1.17, m | 5 | |
| | | b. 1.12, m | | |
| 5 | 28.3 | a. 1.37, m | 6a, 6b | |
| | | b. 1.30, m | | |
| 6 | 38.6 | a. 3.06, m | 5a, 6b, 6-NH | |
| | | b. 3.00, m | 5a, 6a, 6-NH | |
| 6-NH | | 8.51, dd (6.0, 6.0) | 6a, 6b | |
| NH | | 7.69, br s | 2 | |
| 1’ | n.d. | - | | |
| 2’ | 197.3 | - | | |
| 3’ | 24.9 | 2.32, s | | 2’ |
| **L-Ile[4]** | | | | |
| 1 | n.d. | - | | |
| 2 | 57.7 | 3.88, dd (6.7, 6.7) | 3, NH | |
| 3 | 35.8 | 1.44, m | 2, 4b, 6 | |
| 4 | 24.6 | a. 1.10, m | 5 | |
| | | b. 0.80[b] | 3, 5 | |
| 5 | 11.0 | 0.79, dd (7.4, 7.4) | 4a, 4b | |
| 6 | 14.8 | 0.44, d (6.8) | 3 | |
| NH | | 7.71[c] | 2 | |
| **D-Phe[5]** | | | | |
| 1 | n.d. | | | |
| 2 | 54.5 | 4.39, ddd (11.6, 8.3, 3.3) | 3a, 3b, NH | |
| 3 | 36.5 | a. 3.24, m | 2 | |
| | | b. 2.68, dd (13.6, 11.6) | 2 | |
| 4 | 138.0 | - | | |
| 5/9 | 129.0 | 7.22, m | | 3, 7 |
| 6/8 | 128.1 | 7.24, m | 7 | 4 |
| 7 | 126.2 | 7.17, t (7.2) | 6/8 | 5/9 |

| | | | | |
|---|---|---|---|---|
| NH | | 8.41, d (8.4) | 2 | |
| **D-Leu[6]** | | | | |
| 1 | n.d. | - | | |
| 2 | 52.2 | 4.28, m | 3a, 3b, NH | |
| 3 | 40.3 | a. 1.81, m | 2, 3b | 5, 6 |
| | | b. 1.47, m | 2, 3a, 4 | |
| 4 | 24.3 | 1.66, m | 3b, 5, 6 | 3, 5, 6 |
| 5 | 21.5 | 0.86, d (6.5) | 4 | 3, 4, 6 |
| 6 | 23.1 | 0.92, d (6.5) | 4 | 3, 4, 5 |
| NH | | 7.72[c] | 2 | |
| **L-Ile[7]** | | | | |
| 1 | 169.9 | - | | |
| 2 | 57.2 | 4.09, dd (7.6, 7.6) | 3, NH | 1 |
| 3 | 36.0 | 1.76, m | 2, 4b, 6 | |
| 4 | 24.1 | a. 1.28, m | 5 | |
| | | b. 1.11, m | 3, 5 | |
| 5 | 11.2 | 0.79[b] | 4a, 4b | |
| 6 | 15.1[a] | 0.80[b] | 3 | |
| NH | | 7.26 | 2 | |
| **D-Ala[8]** | | | | |
| 1 | 172.4 | - | | |
| 2 | 48.0 | 4.26, dd (6.7, 6.7) | 3, NH | |
| 3 | 18.8 | 1.20, d (6.7) | 2 | 1 |
| NH | | 7.86, d (6.7) | 2 | |

[a-c] Resonances with the same superscripts within a column are overlapped.

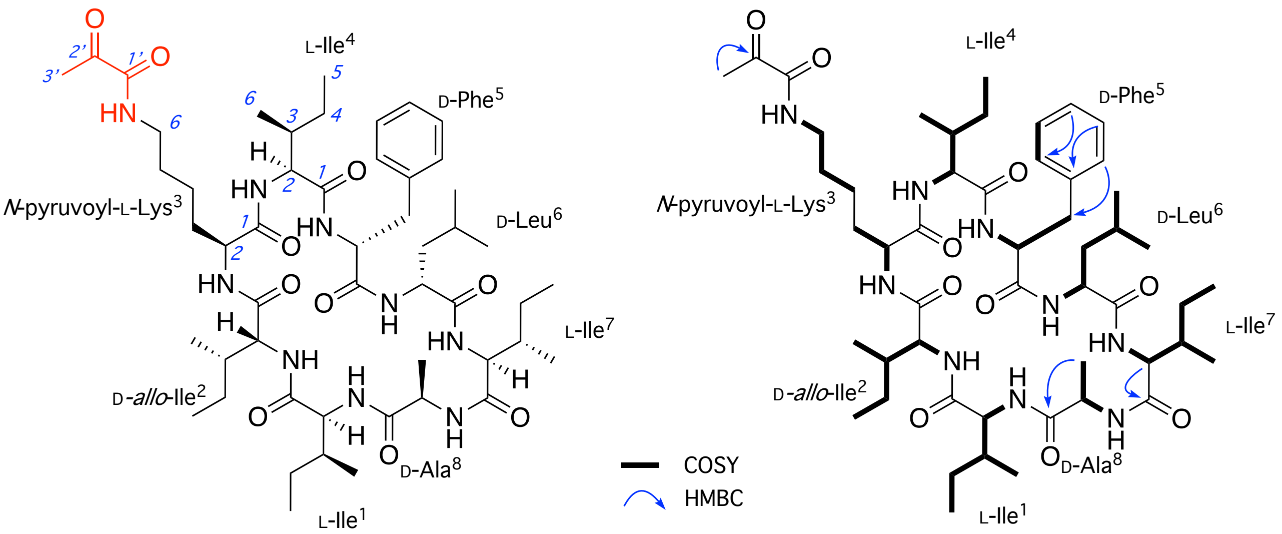


acyl-surugamide A4 (**8**)

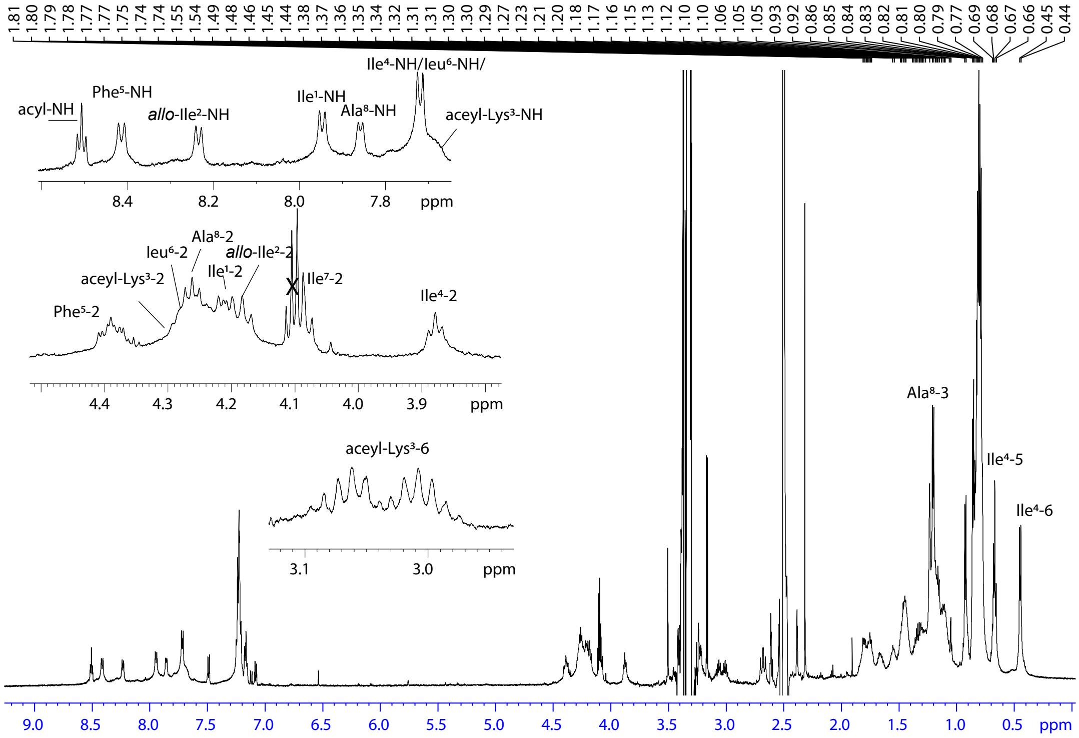


**Figure S40**. $^{1}$H NMR (600 MHz, DMSO-$d_6$) spectrum of acyl-surugamide A4 (**8**)

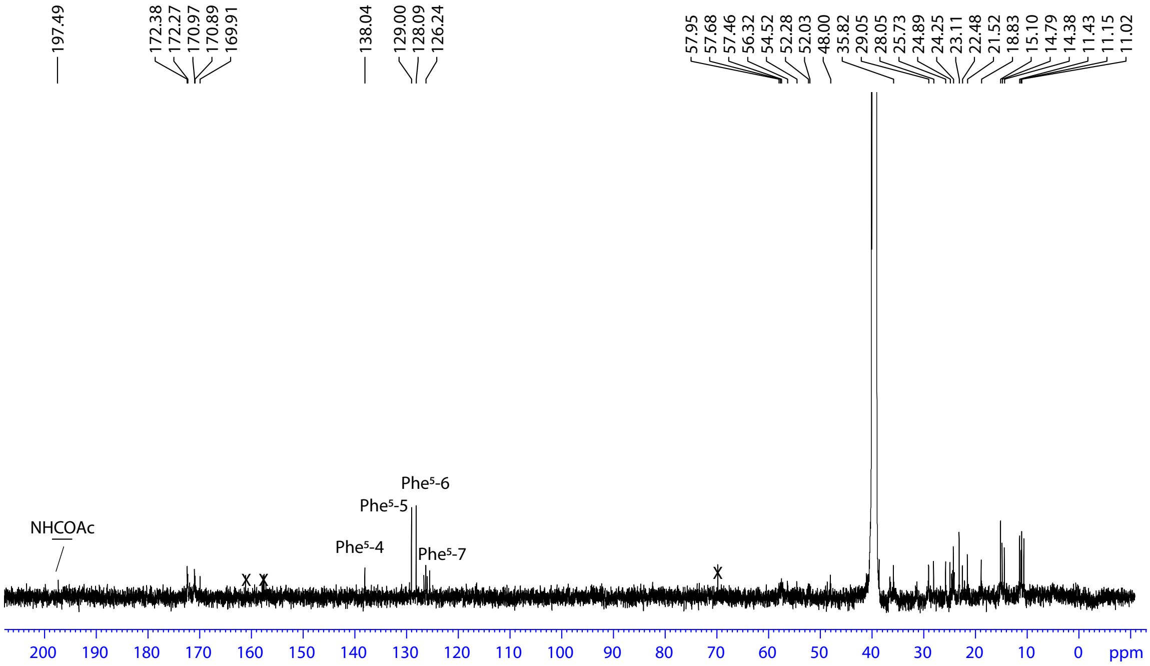


**Figure S41**. $^{13}$C NMR (150 MHz, DMSO-$d_6$) spectrum of acyl-surugamide A4 (**8**)

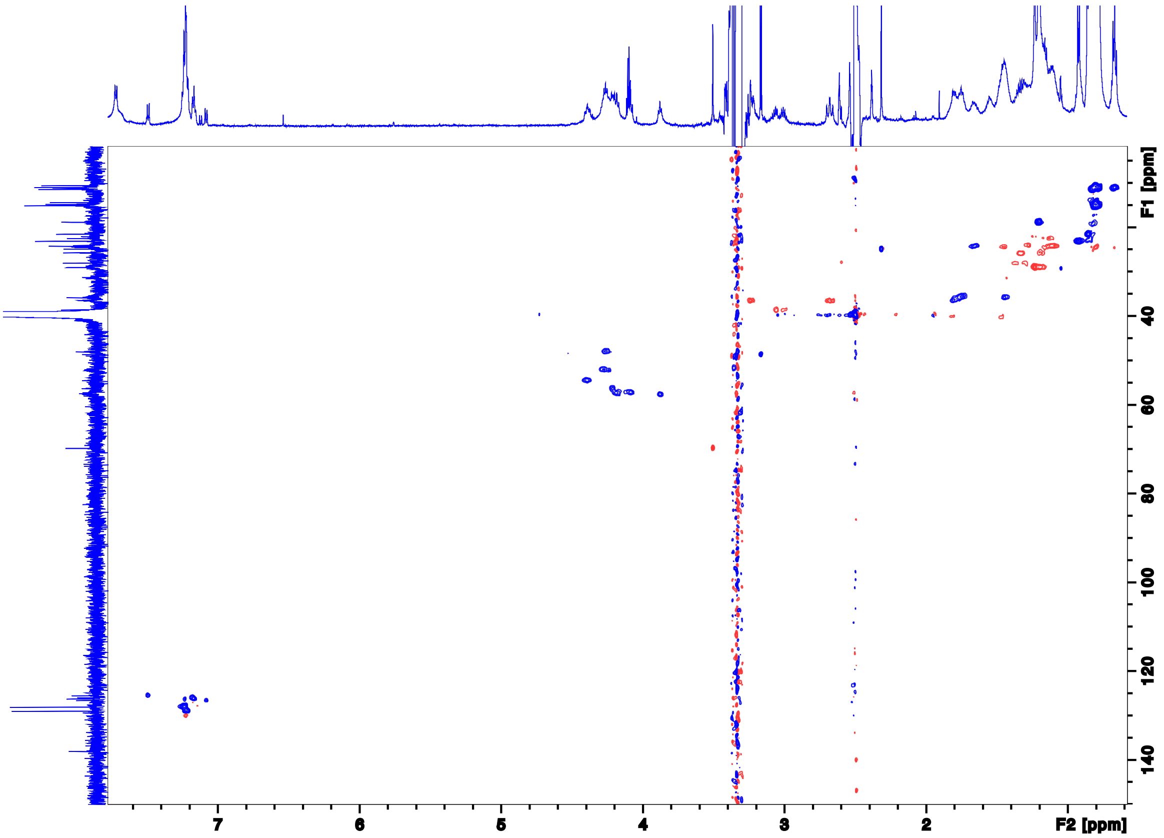


**Figure S42**. HSQC NMR (600 MHz, DMSO-$d_6$) spectrum of acyl-surugamide A4 (**8**)

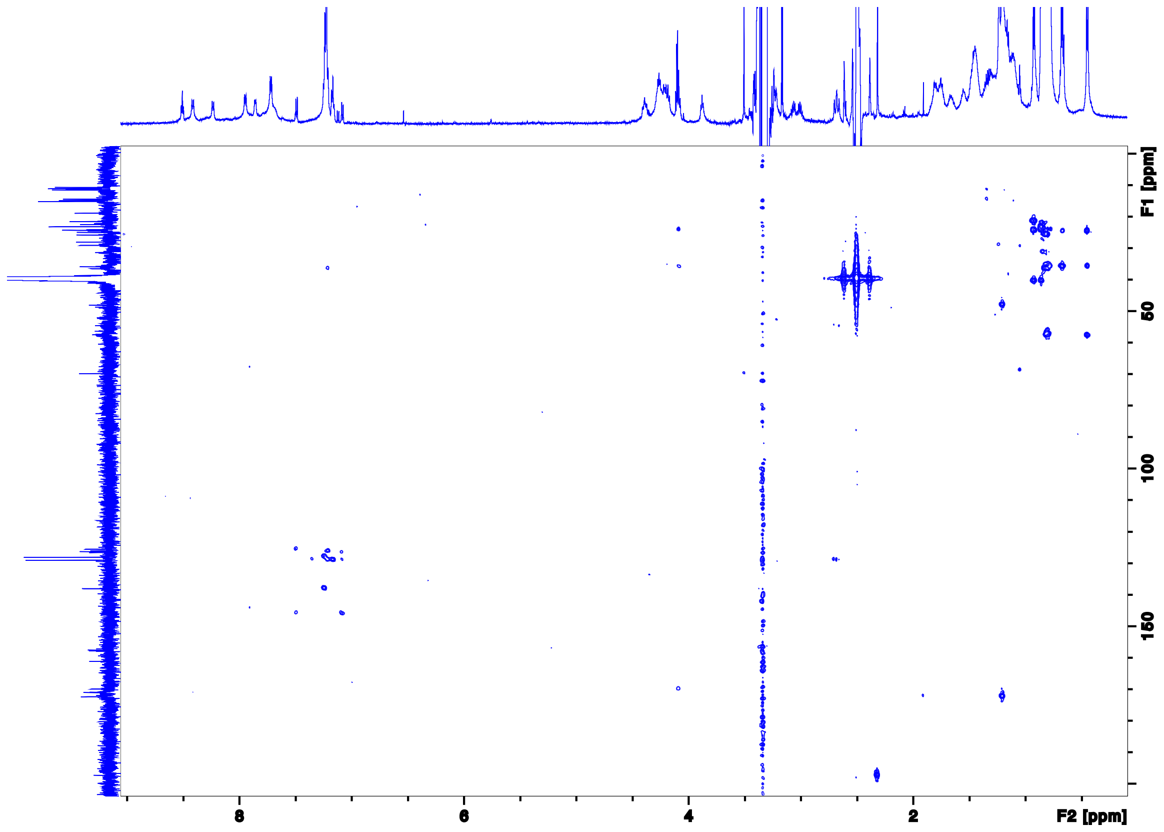


**Figure S43**. HMBC NMR (600 MHz, DMSO-$d_6$) spectrum of acyl-surugamide A4 (**8**)

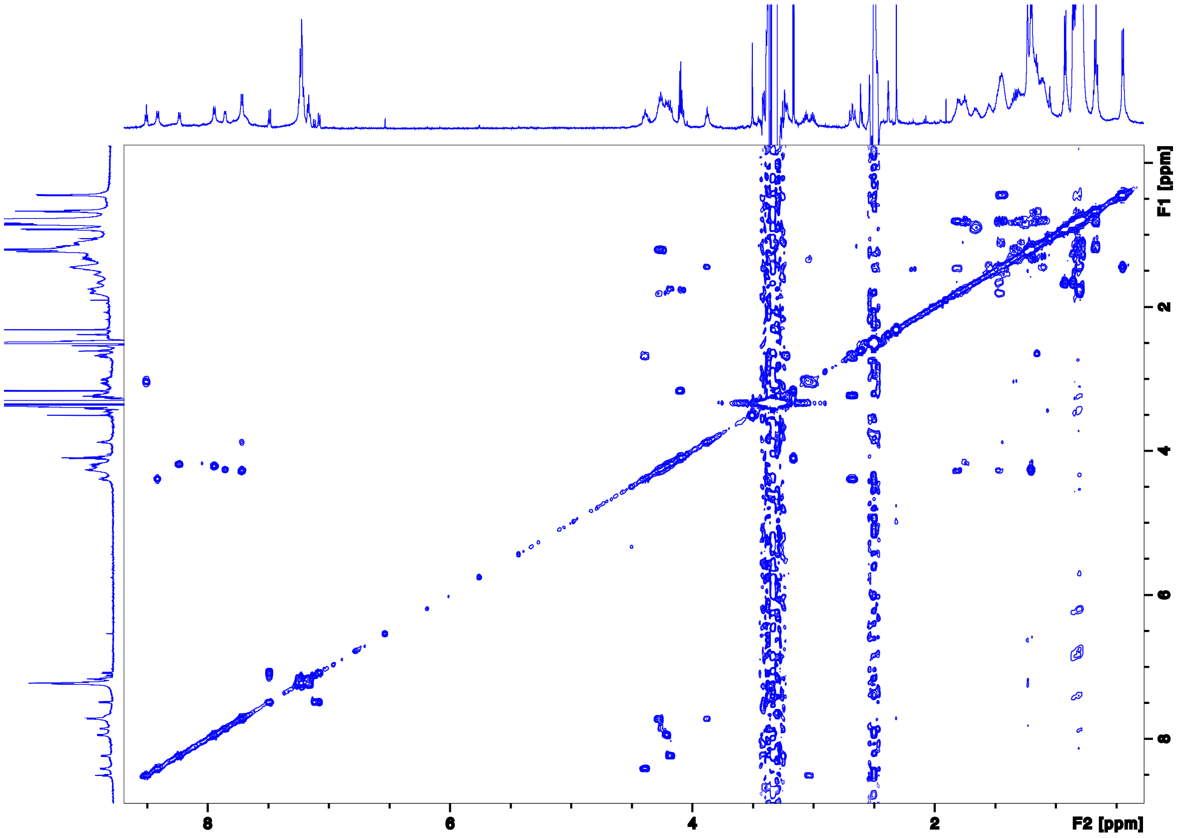


**Figure S44**. COSY NMR (600 MHz, DMSO-$d_6$) spectrum of acyl-surugamide A4 (**8**)

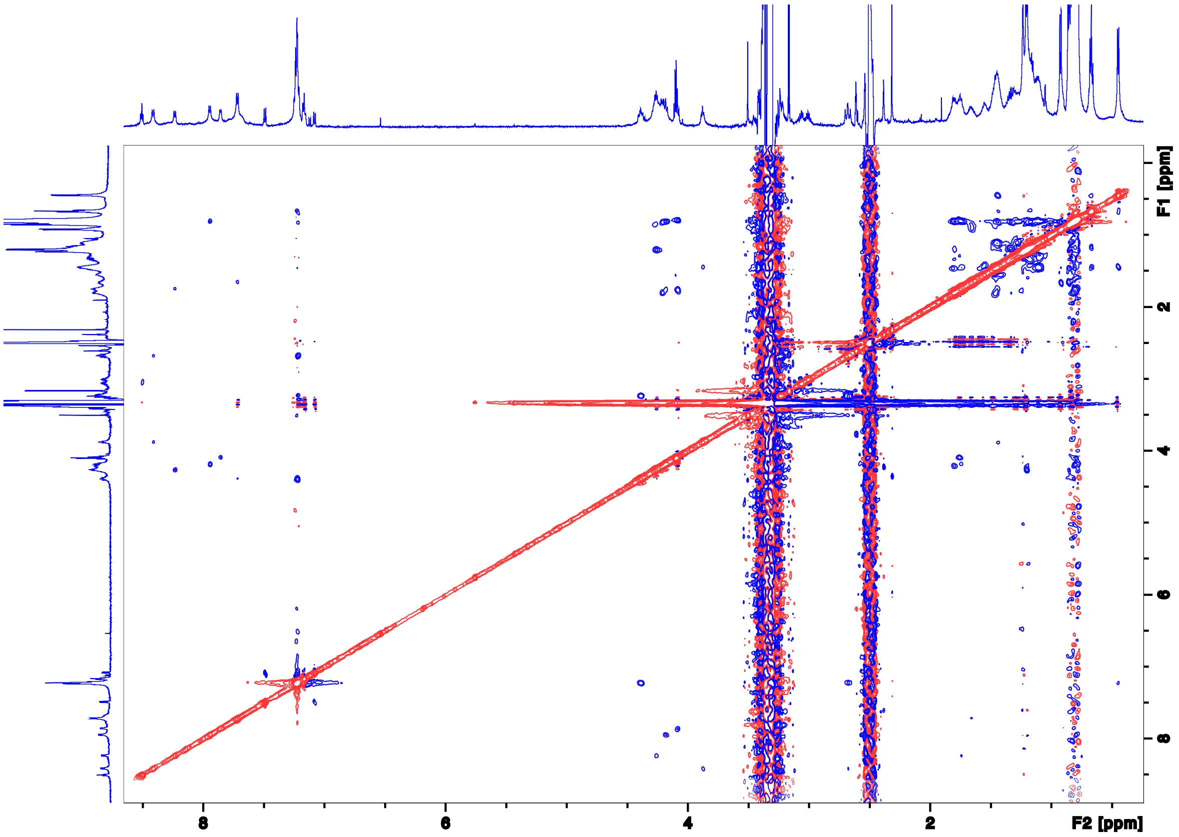


**Figure S45**. ROESY NMR (600 MHz, DMSO-$d_6$) spectrum of acyl-surugamide A4 (**8**)

# Mass Spectrum Molecular Formula Report

**Analysis Info**

| | | | |
|---|---|---|---|
| Analysis Name | D:\Data\Taizong\M0112_F17_19_V29_30_Re_v2.d | Acquisition Date | 3/17/2023 4:21:56 PM |
| Method | tune-medhigh_AP.m | Operator | a.salim |
| Sample Name | M0112_F17_19_V29_30_Re_v2 | Instrument / Ser# | micrOTOF 213750.00232 |
| Comment | | | |

**Acquisition Parameter**

| | | | | | |
|---|---|---|---|---|---|
| Source Type | ESI | Ion Polarity | Positive | Set Nebulizer | 0.5 Bar |
| Focus | Not active | | | Set Dry Heater | 180 °C |
| Scan Begin | 100 m/z | Set Capillary | 4500 V | Set Dry Gas | 5.0 l/min |
| Scan End | 1500 m/z | Set End Plate Offset | -500 V | Set Divert Valve | Source |

**Generate Molecular Formula Parameter**

| | | |
|---|---|---|
| Formula, min. | | |
| Formula, max. | | |
| Measured m/z | Tolerance | Charge |
| Check Valence | Minimum | Maximum |
| Nirogen Rule | Electron Configuration | |
| Filter H/C Ratio | Minimum | Maximum |
| Estimate Carbon | | |

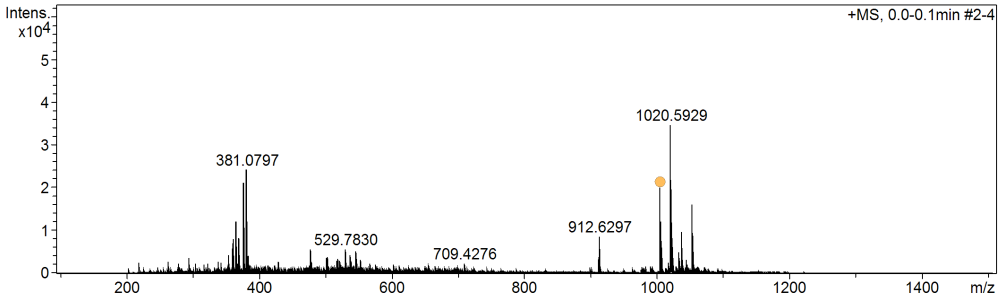


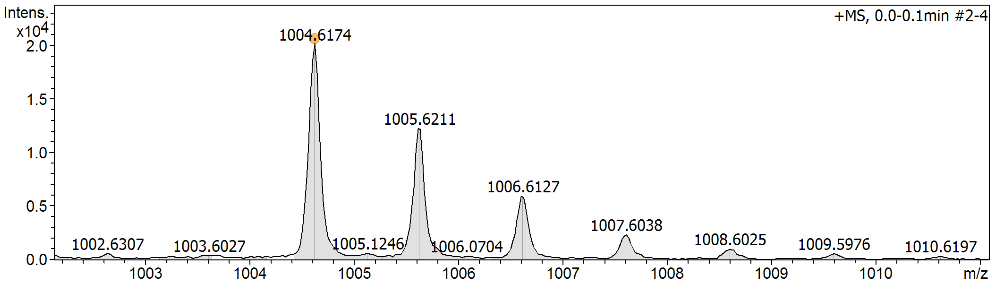


| Meas. m/z | # | Ion Formula | m/z | err [ppm] | mSigma | # Sigma | Score | rdb | e⁻ Conf | N-Rule |
|---|---|---|---|---|---|---|---|---|---|---|
| 1004.6174 | 1 | C51H83N9NaO10 | 1004.6155 | 1.9 | 57.0 | 1 | 100.00 | 14.5 | even | ok |

Bruker Compass DataAnalysis 4.1 printed: 3/20/2023 12:12:46 PM Page 1 of 1

**Figure S46**. HRMS measurement for acyl-surugamide A4 (**8**)

(a) MSMS chromatogram of surugamide A (**3**)

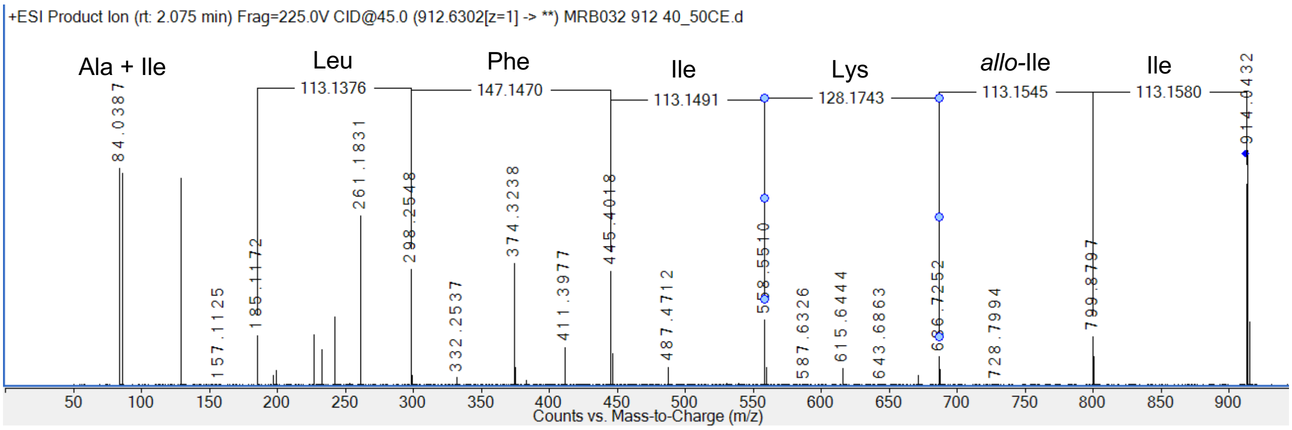


(b) MSMS chromatogram of surugamide K (**4**)

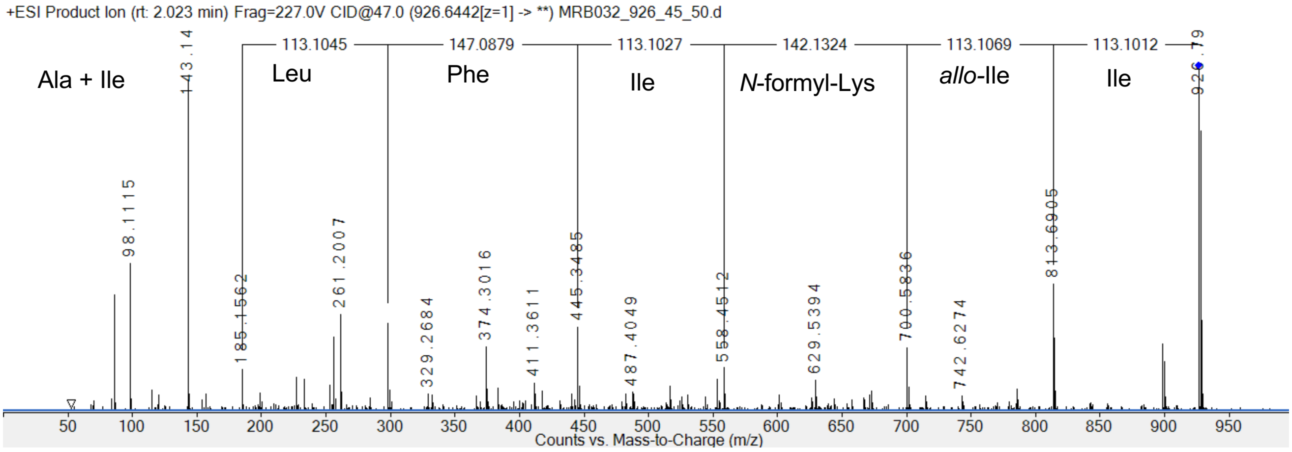


(c) MSMS chromatogram of acyl-surugamide A1 (**5**)

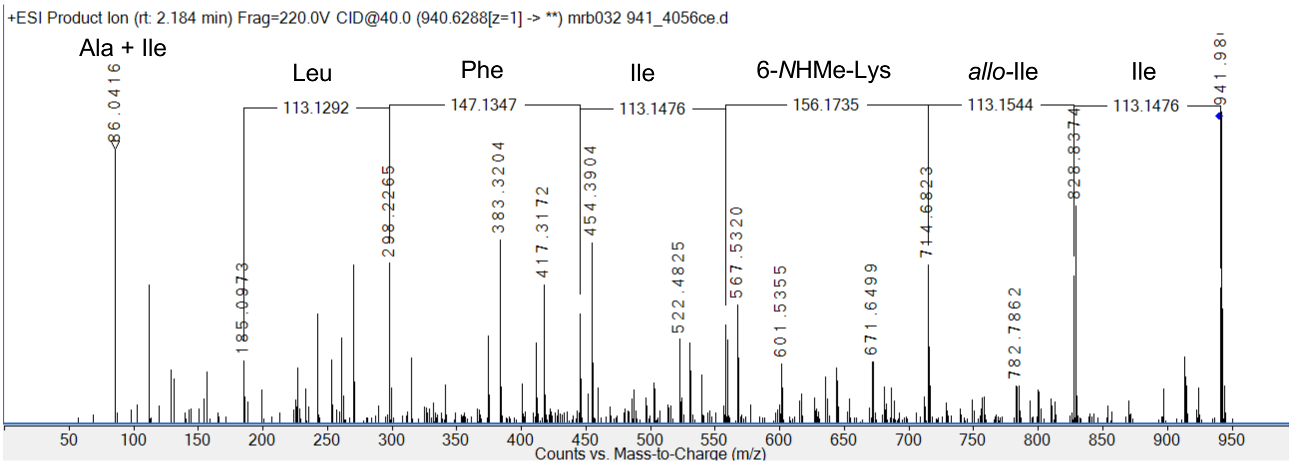

(d) MSMS chromatogram of acyl-surugamide A2 (**6**)

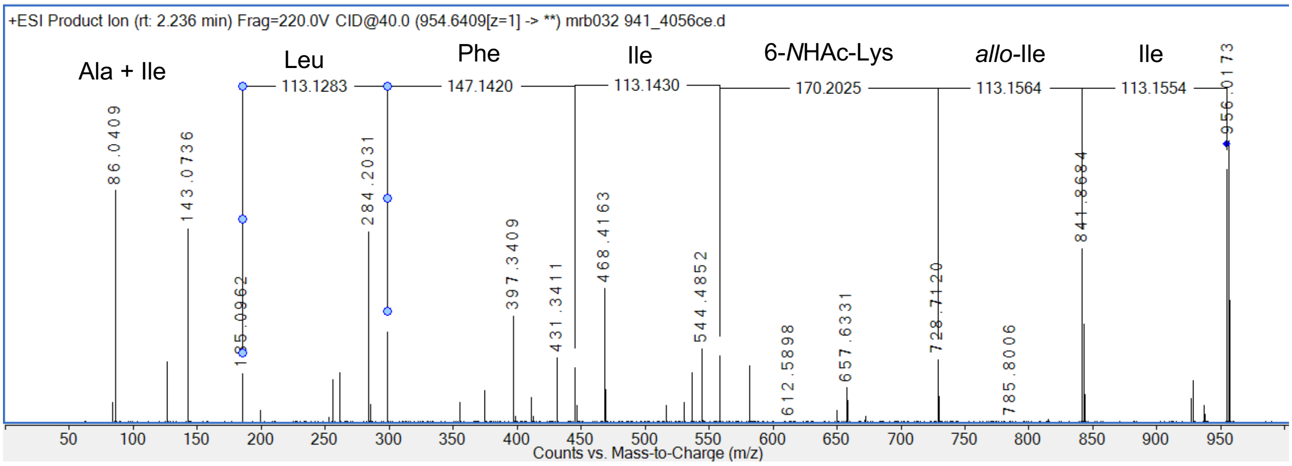


(e) MSMS chromatogram of acyl-surugamide A3 (**7**)

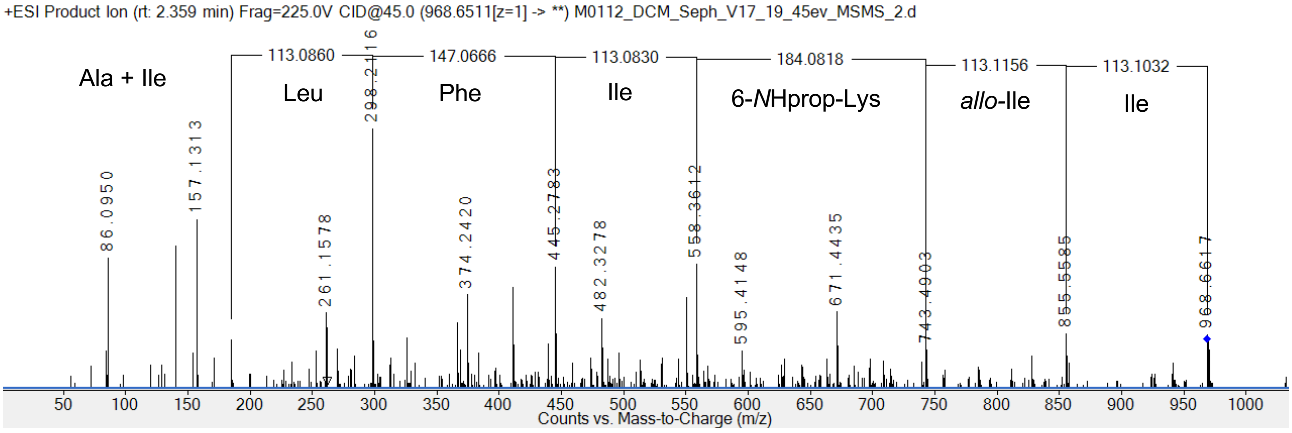


(f) MSMS chromatogram of acyl-surugamide A4 (**8**)

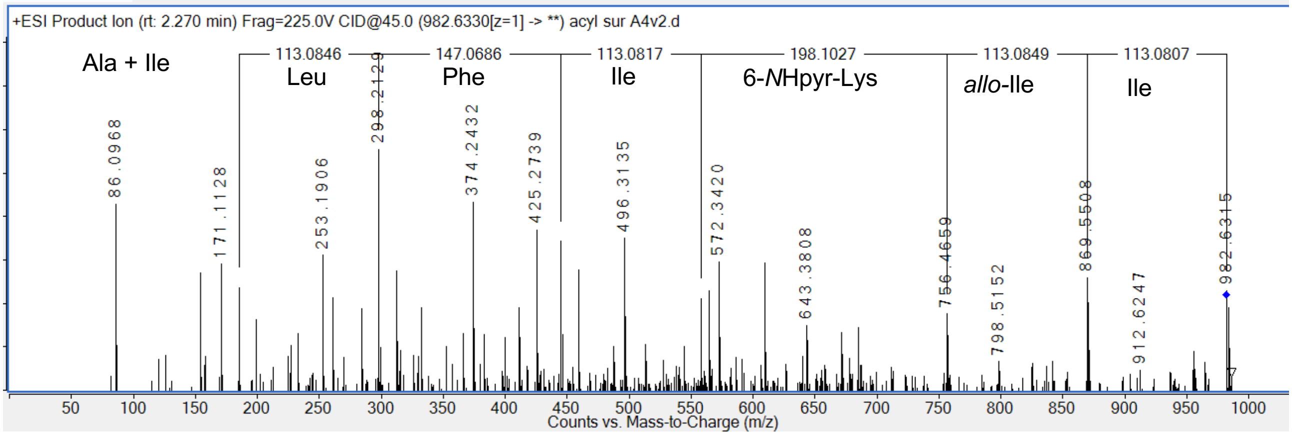

(g) MSMS chromatogram of acyl-surugamide A detected in CMB-MRB032 (not isolated)

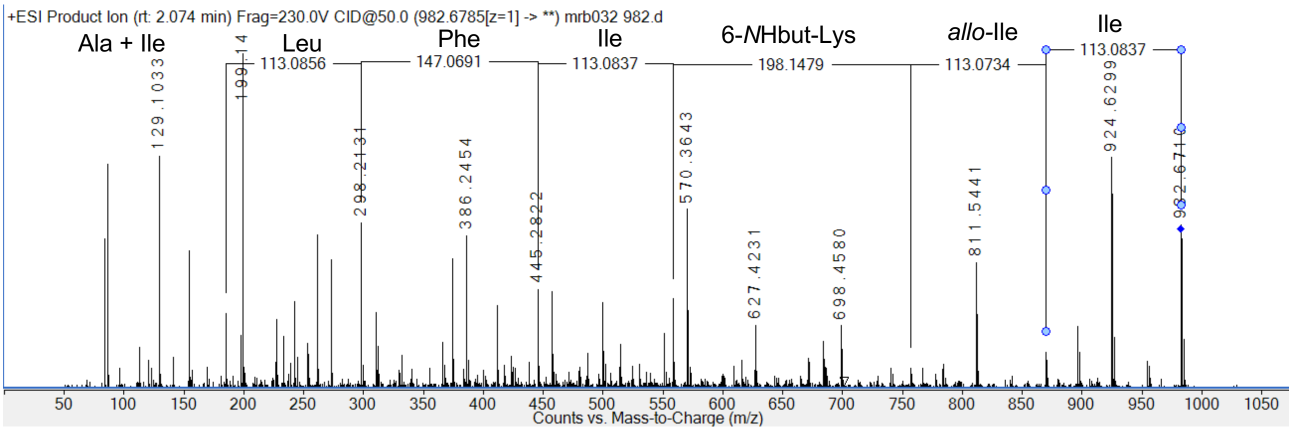


**Figure S47**. MSMS spectra for surugamides **3-8** and acyl-surugamide A

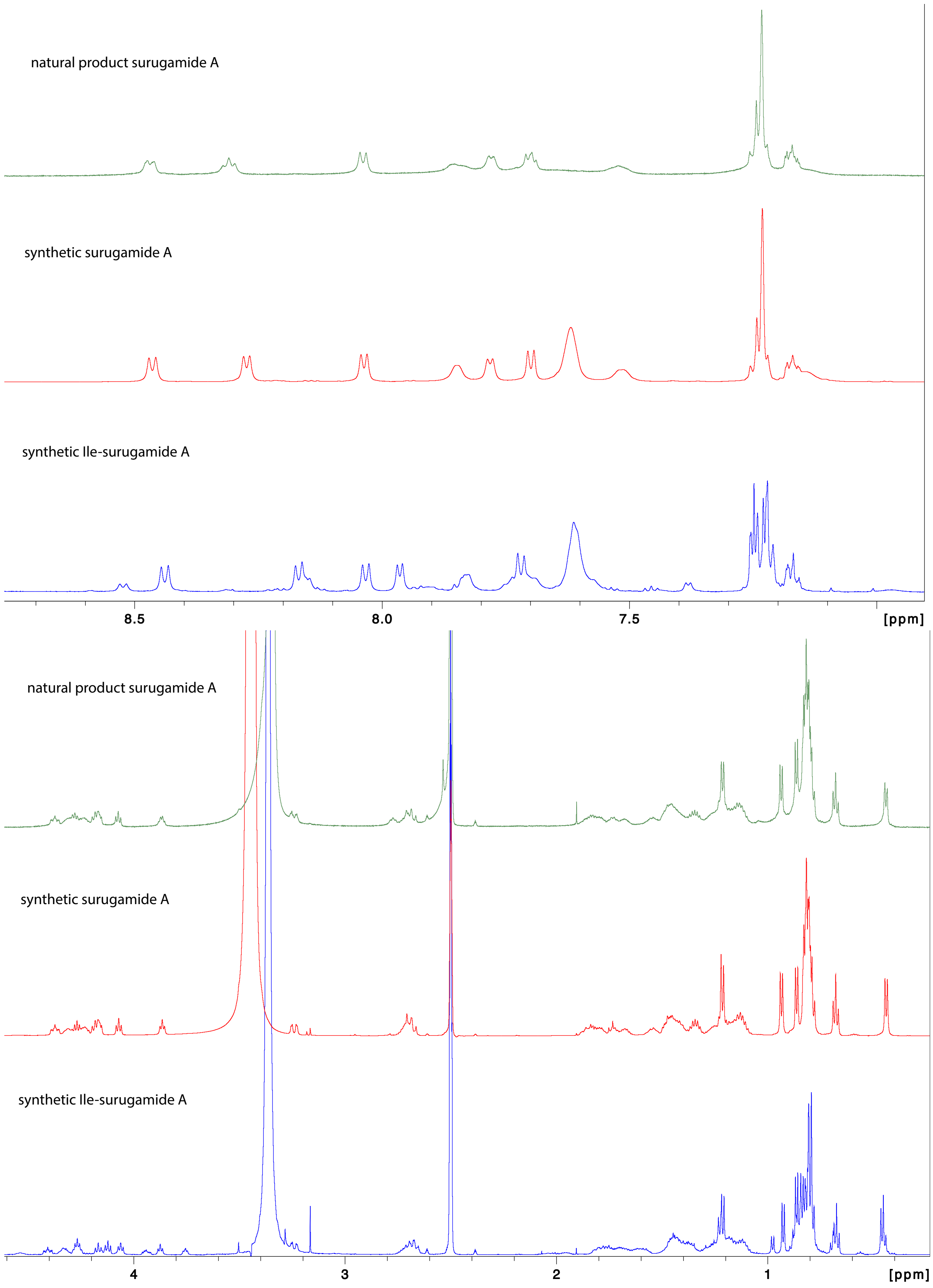


**Figure S48**. $^{1}$H NMR (600 MHz, DMSO-$d_6$) spectra of natural surugamide A (**3**) (green), synthetic surugamide A (**3'**) (red) and surugamide S1 [D-Ile$^{2}$-surugamide A] (**9**) (blue)

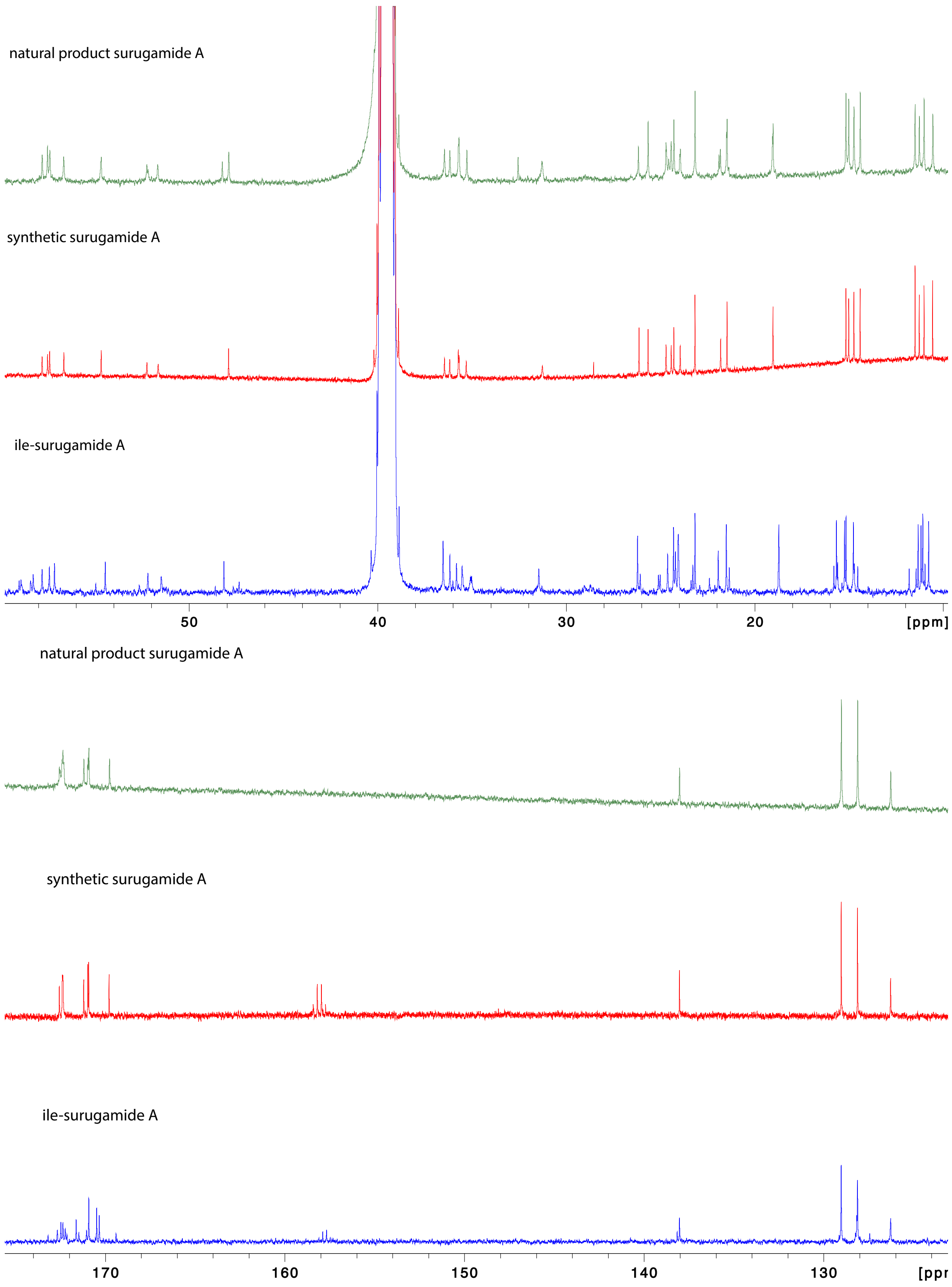


**Figure S49**. $^{13}$C NMR (150 MHz, DMSO-$d_6$) spectra of natural surugamide A (**3**) (green), synthetic surugamide A (**3'**) (red) and surugamide S1 [D-Ile$^2$-surugamide A] (**9**) (blue)

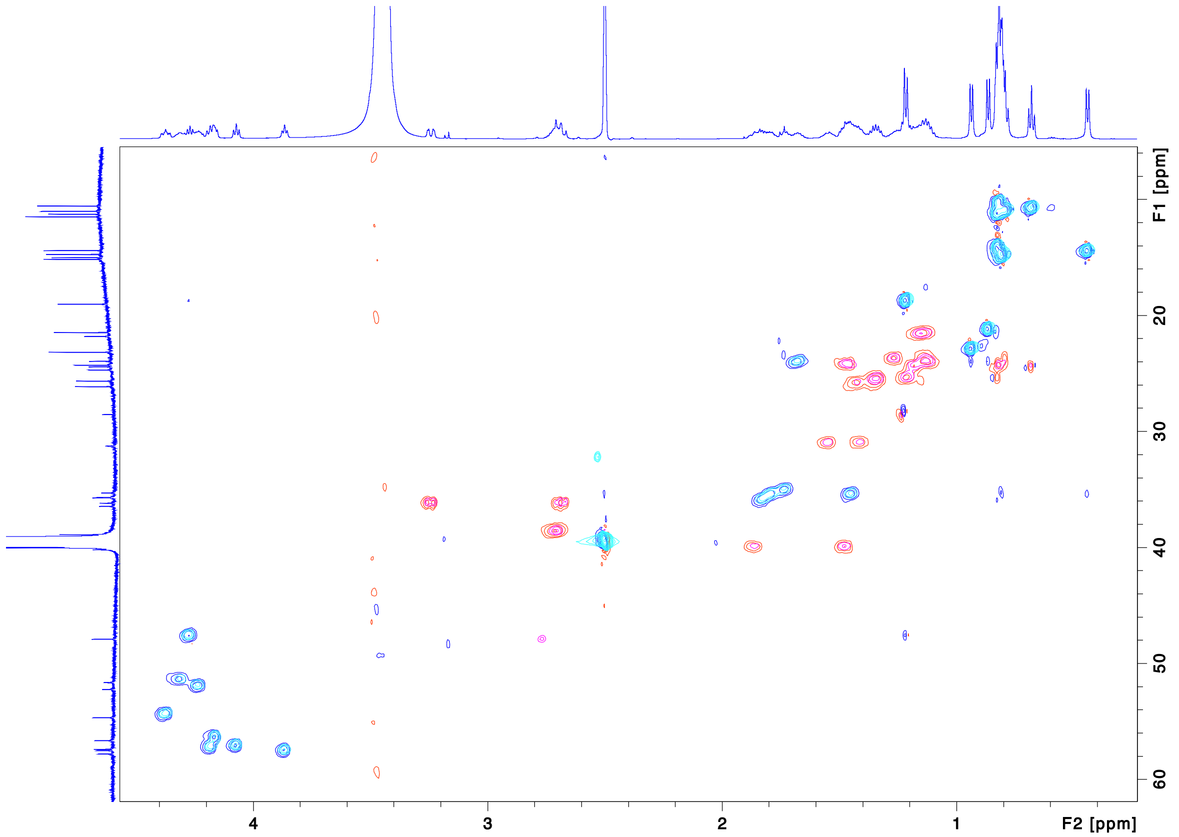


**Figure S50.** Overlay of HSQC (600 MHz, DMSO-d6) spectra of natural surugamide A (**3**) (cyan and magenta peaks) and synthetic surugamide A (**3'**) (blue and red peaks)

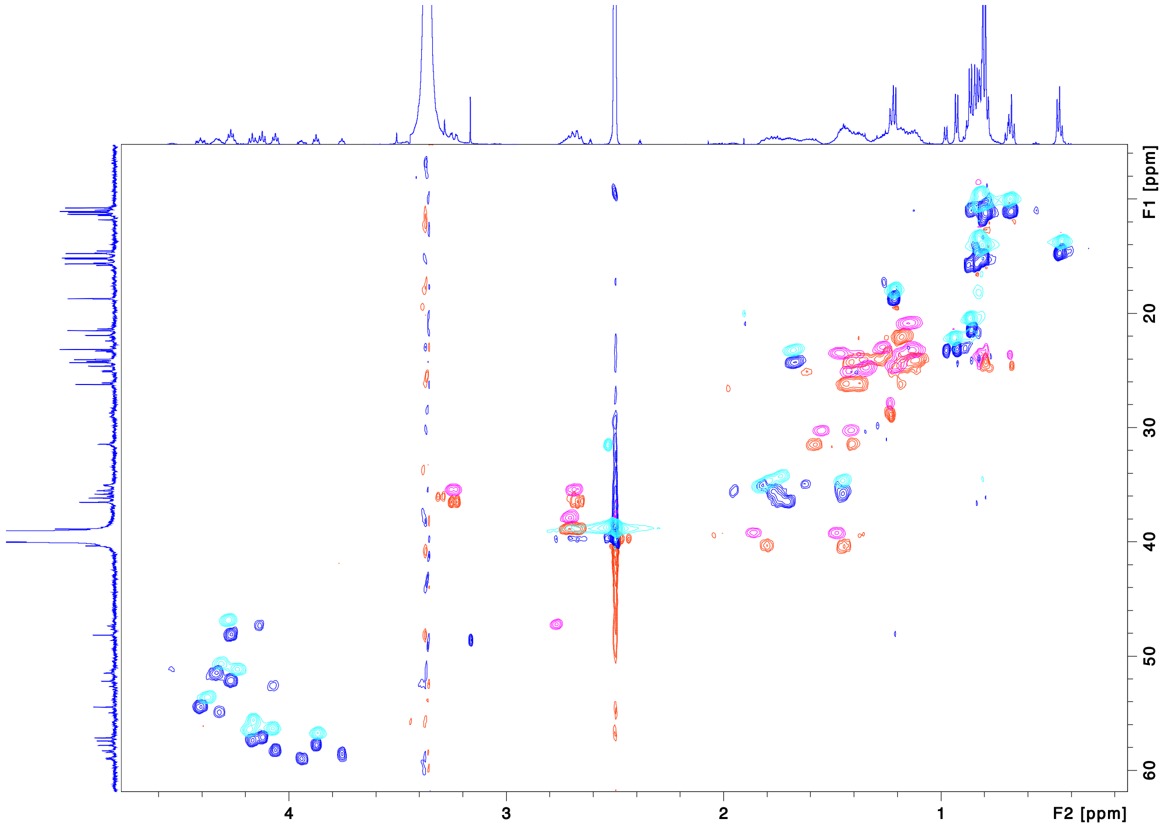


**Figure S51**. Overlay of HSQC (600 MHz, DMSO-$d_6$) spectra of natural surugamide A (**3**) (cyan and magenta peaks) and surugamide S1 [D-Ile$^2$-surugamide A] (**9**) (blue and red peaks)

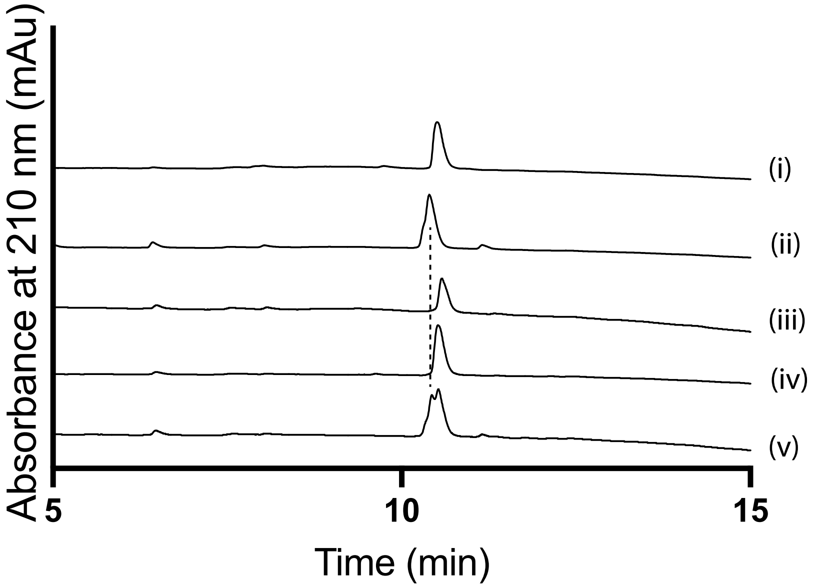


**Figure S52**. HPLC profile of (i): synthetic surugamide A (**3'**); (ii) surugamide S1 [synthetic D-Ile$^2$ surugamide A] (**9**); (iii) natural surugamide A (**1**); (iv) co-injection of synthetic surugamide A (**3'**) and natural surugamide A (**3**); (v) co-injection of surugamide S1 [synthetic D-Ile$^2$ surugamide A] (**9**) and natural surugamide A (**3**).

## 7. Spectroscopic data of synthetic products

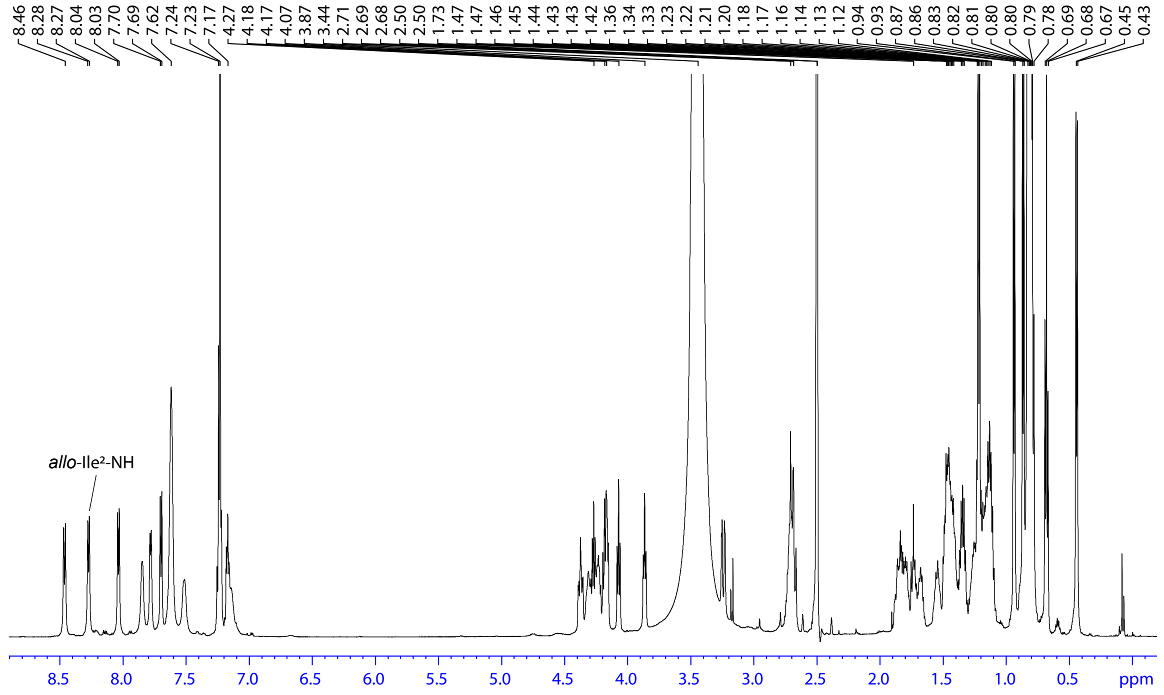


**Figure S53**. $^{1}$H NMR (600 MHz, DMSO-$d_6$) spectrum of synthetic surugamide A (**3'**).

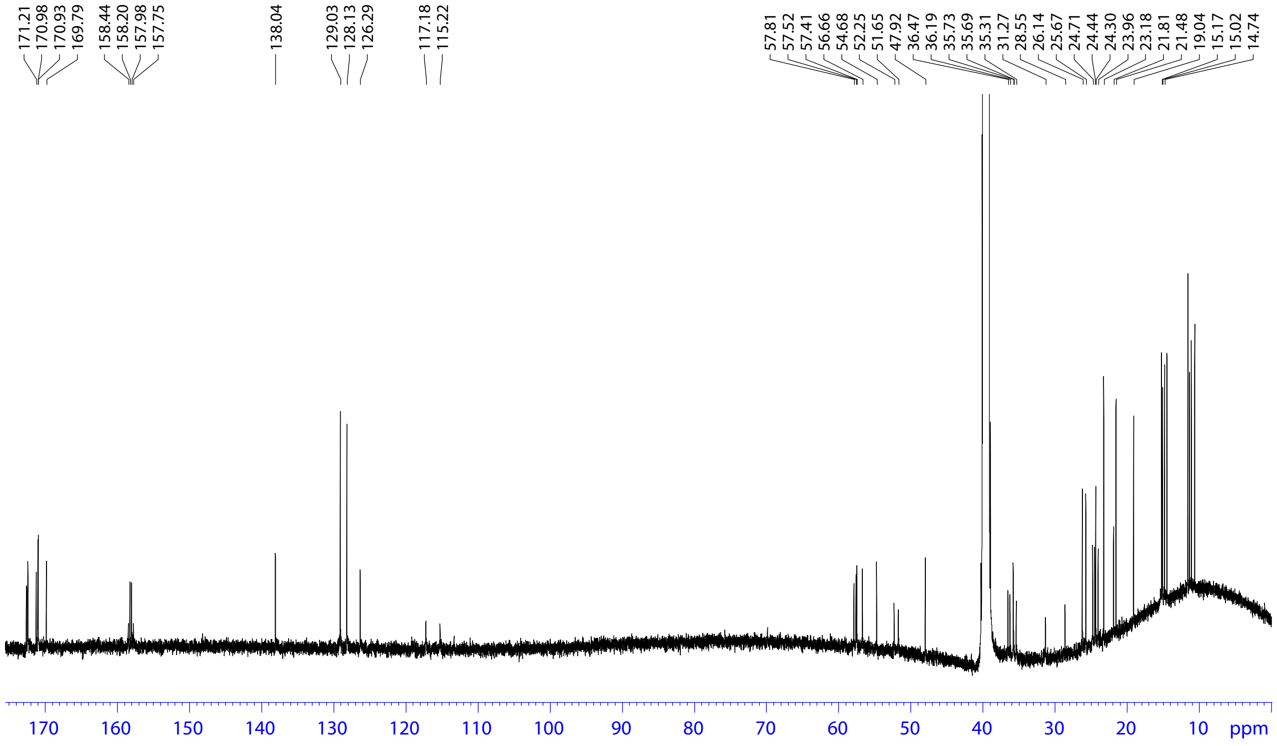


**Figure S54**. $^{13}$C NMR (150 MHz, DMSO-$d_6$) spectrum of synthetic surugamide A (**3'**).

# Mass Spectrum Molecular Formula Report

**Analysis Info**

| | | | | |
|---|---|---|---|---|
| Analysis Name | D:\Data\Taizong\Synthetic Surugamide A alloIle.d | Acquisition Date | 6/1/2023 11:09:47 AM | |
| Method | tune-medhigh_AP.m | Operator | a.salim | |
| Sample Name | Synthetic Surugamide A alloIle | Instrument / Ser# | micrOTOF | 213750.00 232 |
| Comment | | | | |

**Acquisition Parameter**

| | | | | | |
|---|---|---|---|---|---|
| Source Type | ESI | Ion Polarity | Positive | Set Nebulizer | 0.5 Bar |
| Focus | Not active | | | Set Dry Heater | 180 °C |
| Scan Begin | 100 m/z | Set Capillary | 4500 V | Set Dry Gas | 5.0 l/min |
| Scan End | 1500 m/z | Set End Plate Offset | -500 V | Set Divert Valve | Source |

**Generate Molecular Formula Parameter**

| | | |
|---|---|---|
| Formula, min. | | |
| Formula, max. | | |
| Measured m/z | Tolerance | Charge |
| Check Valence | Minimum | Maximum |
| Nirogen Rule | Electron Configuration | |
| Filter H/C Ratio | Minimum | Maximum |
| Estimate Carbon | | |

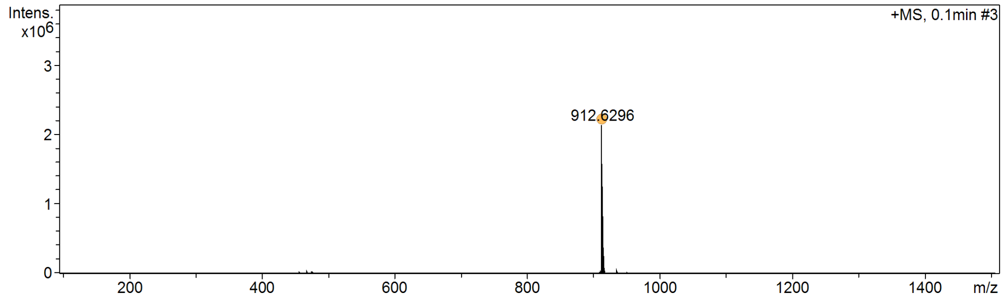


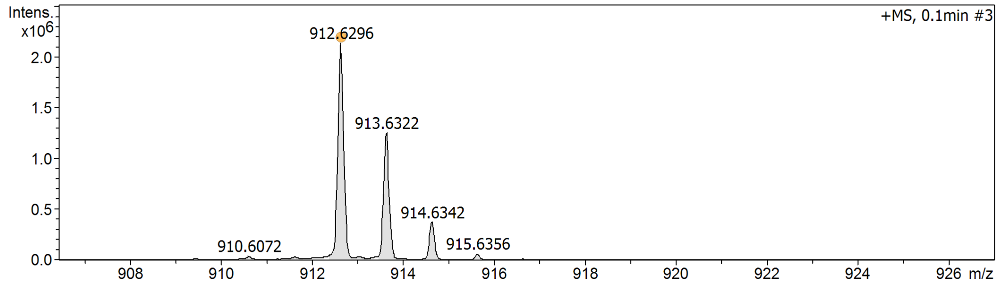


| Meas. m/z | # | Ion Formula | m/z | err [ppm] | mSigma | # Sigma | Score | rdb | e⁻ Conf | N-Rule |
|---|---|---|---|---|---|---|---|---|---|---|
| 912.6296 | 1 | C48H82N9O8 | 912.6281 | 1.7 | 9.5 | 1 | 100.00 | 12.5 | even | ok |

Bruker Compass DataAnalysis 4.1 printed: 6/1/2023 11:15:53 AM Page 1 of 1

**Figure S55**. HRMS measurement for synthetic surugamide A (**3'**).

# Mass Spectrum Molecular Formula Report

**Analysis Info**

| | | | |
|---|---|---|---|
| Analysis Name | D:\Data\Taizong\M0112_V21_24_P CHLORIDE_V6-8.d | Acquisition Date | 5/25/2023 10:09:49 AM |
| Method | tune-medhigh_AP.m | Operator | a.salim |
| Sample Name | M0112_V21_24_P CHLORIDE_V6-8 | Instrument / Ser# | micrOTOF 213750.00232 |
| Comment | | | |

**Acquisition Parameter**

| | | | | | |
|---|---|---|---|---|---|
| Source Type | ESI | Ion Polarity | Positive | Set Nebulizer | 0.5 Bar |
| Focus | Not active | | | Set Dry Heater | 180 °C |
| Scan Begin | 100 m/z | Set Capillary | 4500 V | Set Dry Gas | 5.0 l/min |
| Scan End | 1500 m/z | Set End Plate Offset | -500 V | Set Divert Valve | Source |

**Generate Molecular Formula Parameter**

| | | |
|---|---|---|
| Formula, min. | | |
| Formula, max. | | |
| Measured m/z | Tolerance | Charge |
| Check Valence | Minimum | Maximum |
| Nirogen Rule | Electron Configuration | |
| Filter H/C Ratio | Minimum | Maximum |
| Estimate Carbon | | |

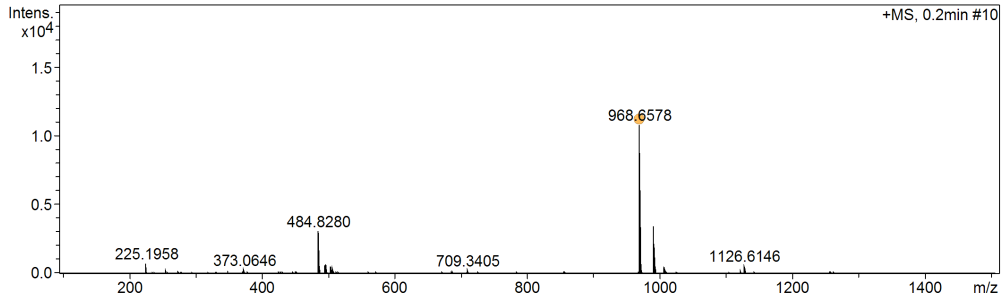


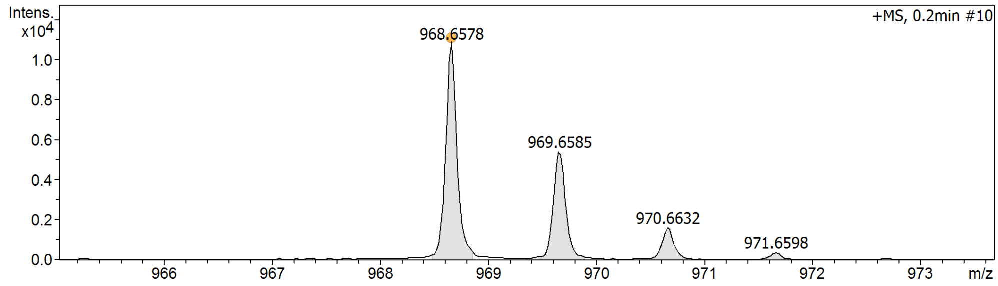


| Meas. m/z | # | Ion Formula | m/z | err [ppm] | mSigma | # Sigma | Score | rdb | e⁻ Conf | N-Rule |
|---|---|---|---|---|---|---|---|---|---|---|
| 968.6578 | 1 | C51H86N9O9 | 968.6543 | 3.6 | 49.2 | 1 | 100.00 | 13.5 | even | ok |



**Figure S56**. HRMS measurement for semi-synthetic acyl-surugamide A3 (**7'**)

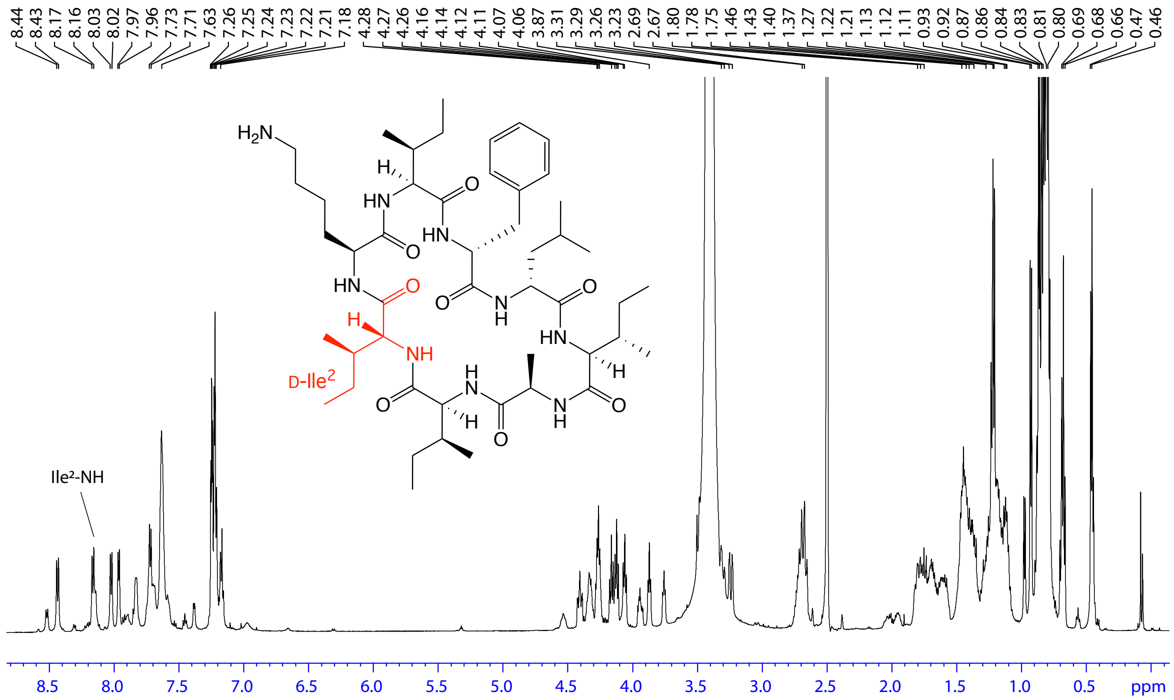


**Figure S57**. $^{1}$H NMR (600 MHz, DMSO-$d_6$) spectrum of surugamide S1 [D-Ile[2] surugamide A] (**9**).

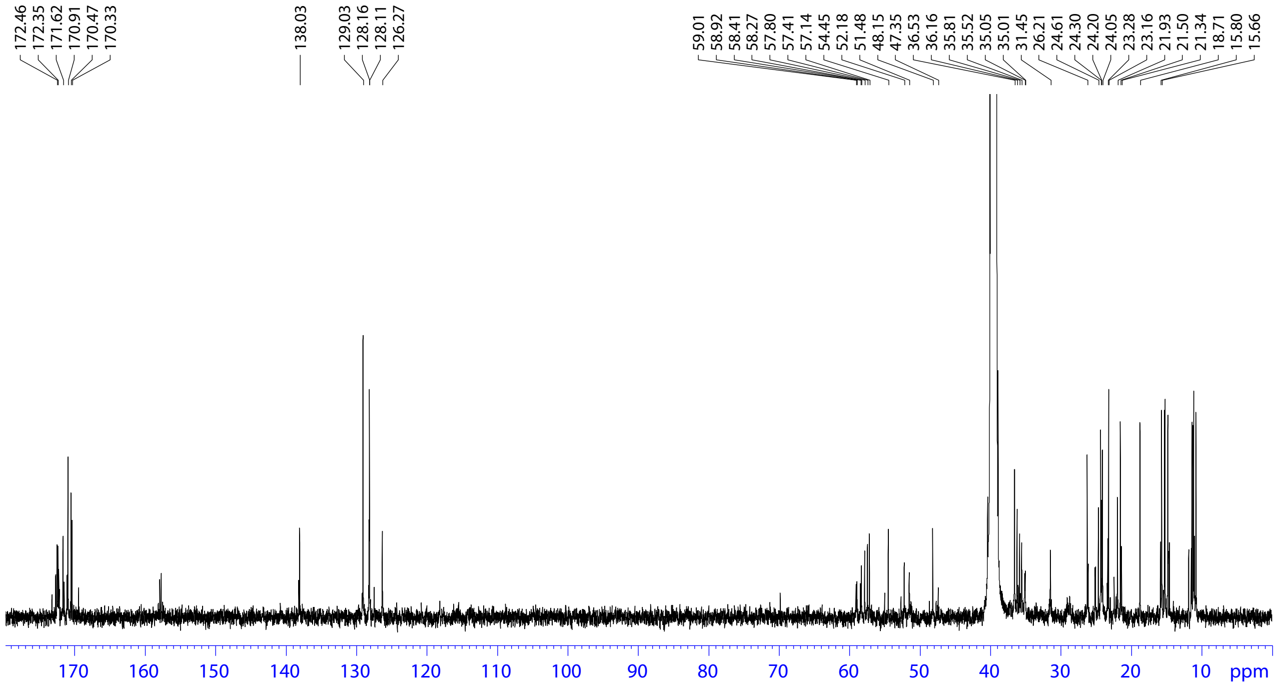


**Figure S58**. $^{13}$C NMR (150 MHz, DMSO-$d_6$) spectrum of synthetic surugamide S1 (**9**)

# Mass Spectrum Molecular Formula Report

**Analysis Info**

| | | | |
|---|---|---|---|
| Analysis Name | D:\Data\Taizong\Synthetic Surugamide A Ile.d | Acquisition Date | 6/1/2023 11:06:56 AM |
| Method | tune-medhigh_AP.m | Operator | a.salim |
| Sample Name | Synthetic Surugamide A Ile | Instrument / Ser# | micrOTOF 213750.00232 |
| Comment | | | |

**Acquisition Parameter**

| | | | | | |
|---|---|---|---|---|---|
| Source Type | ESI | Ion Polarity | Positive | Set Nebulizer | 0.5 Bar |
| Focus | Not active | | | Set Dry Heater | 180 °C |
| Scan Begin | 100 m/z | Set Capillary | 4500 V | Set Dry Gas | 5.0 l/min |
| Scan End | 1500 m/z | Set End Plate Offset | -500 V | Set Divert Valve | Source |

**Generate Molecular Formula Parameter**

| | | | |
|---|---|---|---|
| Formula, min. | | | |
| Formula, max. | | | |
| Measured m/z | Tolerance | Charge | |
| Check Valence | Minimum | Maximum | |
| Nirogen Rule | Electron Configuration | | |
| Filter H/C Ratio | Minimum | Maximum | |
| Estimate Carbon | | | |

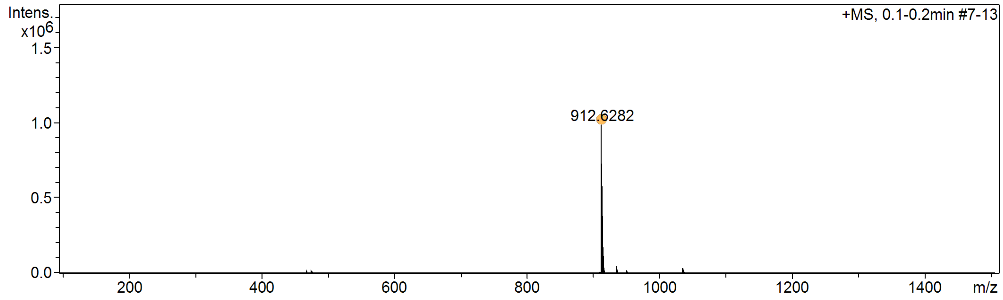


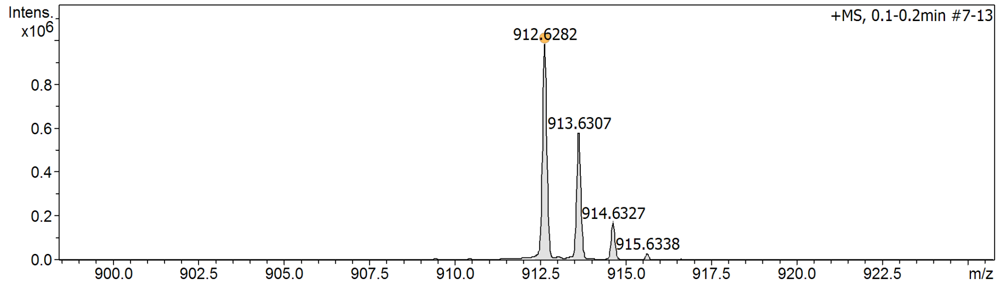


| Meas. m/z | # | Ion Formula | m/z | err [ppm] | mSigma | # Sigma | Score | rdb | e⁻ Conf | N-Rule |
|---|---|---|---|---|---|---|---|---|---|---|
| 912.6282 | 1 | C48H82N9O8 | 912.6281 | -0.2 | 10.7 | 1 | 100.00 | 12.5 | even | ok |

**Figure S59**. HRMS measurement for surugamide S1 [D-Ile$^2$ surugamide A] (**9**).

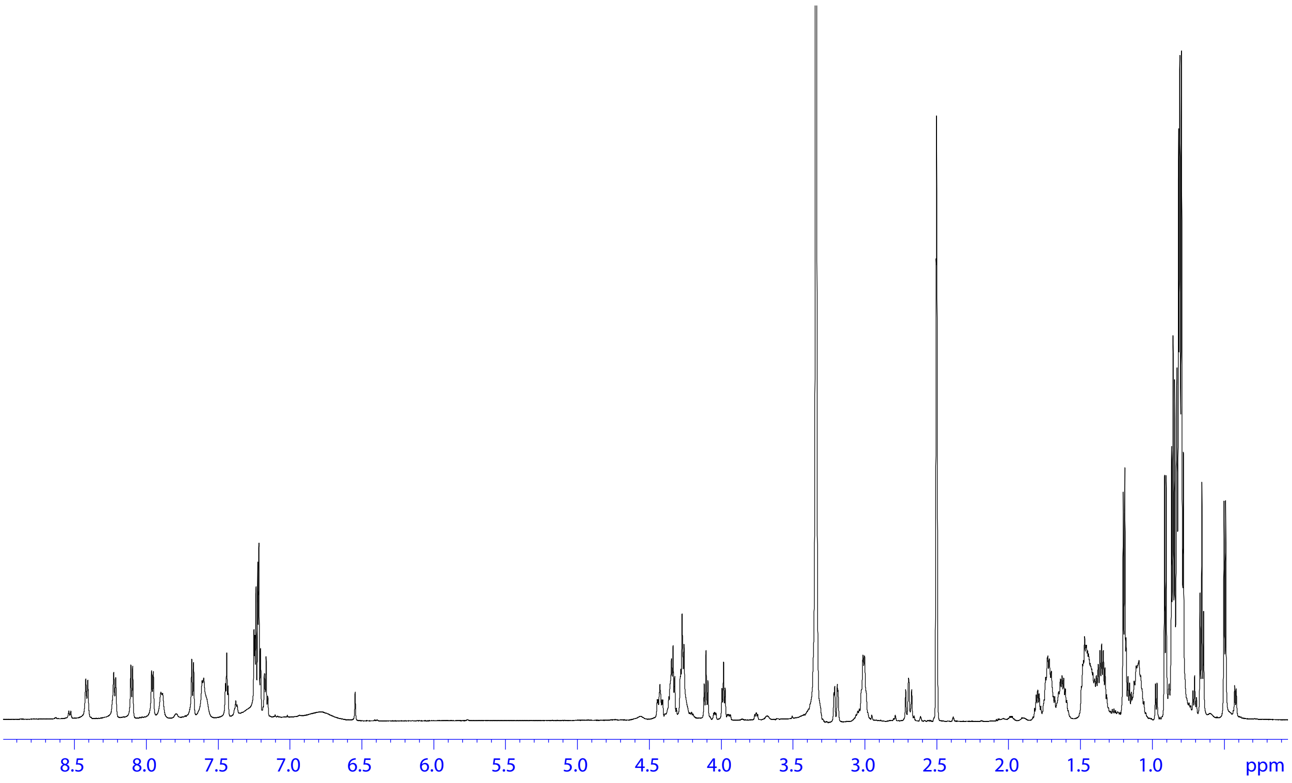


**Figure S60**. $^{1}$H NMR (600 MHz, DMSO-$d_6$) spectrum of synthetic surugamide S2 (**10**)

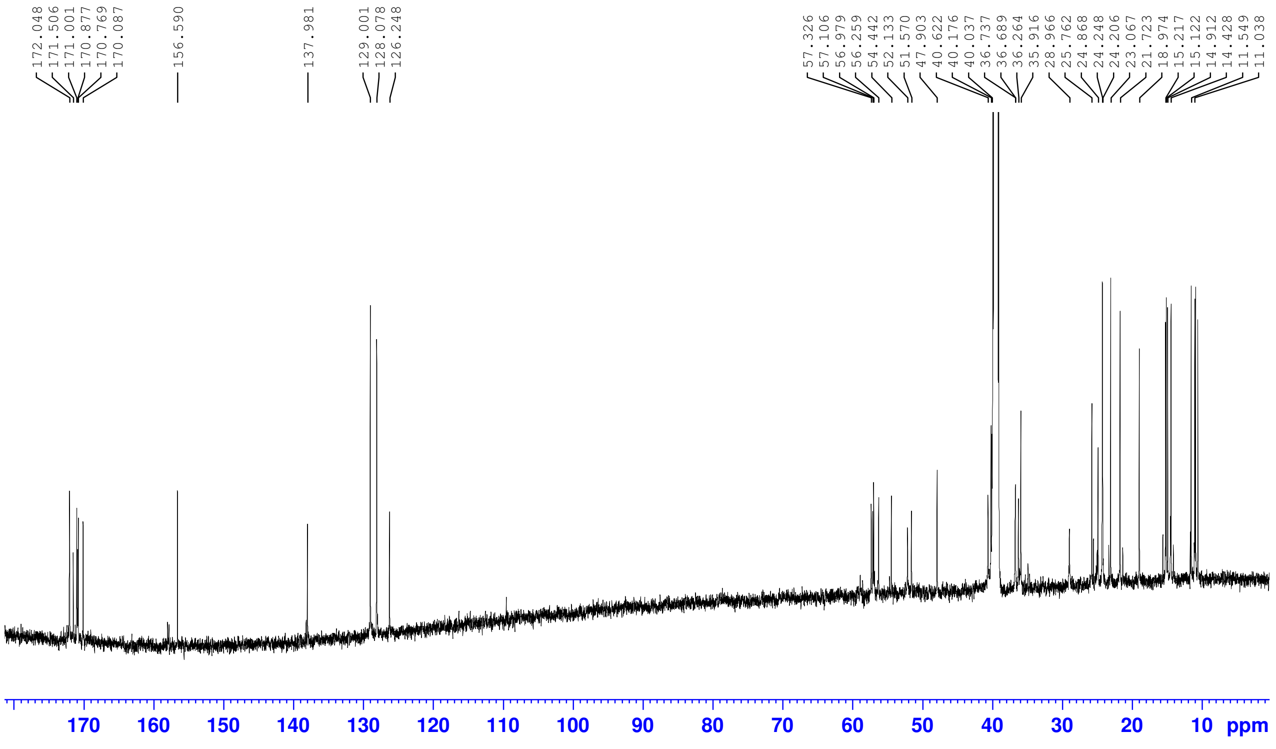


**Figure S61**. $^{13}$C NMR (150 MHz, DMSO-$d_6$) spectrum of synthetic surugamide S2 (**10**)

# Mass Spectrum Molecular Formula Report

**Analysis Info**

| | | | | |
|---|---|---|---|---|
| Analysis Name | D:\Data\Taizong\Arg Surugamide A allo.d | Acquisition Date | 6/1/2023 10:20:57 AM | |
| Method | tune-medhigh_AP.m | Operator | a.salim | |
| Sample Name | Arg Surugamide A allo | Instrument / Ser# | micrOTOF | 213750.00232 |
| Comment | | | | |

**Acquisition Parameter**

| | | | | | |
|---|---|---|---|---|---|
| Source Type | ESI | Ion Polarity | Positive | Set Nebulizer | 0.5 Bar |
| Focus | Not active | | | Set Dry Heater | 180 °C |
| Scan Begin | 100 m/z | Set Capillary | 4500 V | Set Dry Gas | 5.0 l/min |
| Scan End | 1500 m/z | Set End Plate Offset | -500 V | Set Divert Valve | Source |

**Generate Molecular Formula Parameter**

| | | | | | |
|---|---|---|---|---|---|
| Formula, min. | | | | | |
| Formula, max. | | | | | |
| Measured m/z | | Tolerance | | Charge | |
| Check Valence | | Minimum | | Maximum | |
| Nirogen Rule | | Electron Configuration | | | |
| Filter H/C Ratio | | Minimum | | Maximum | |
| Estimate Carbon | | | | | |

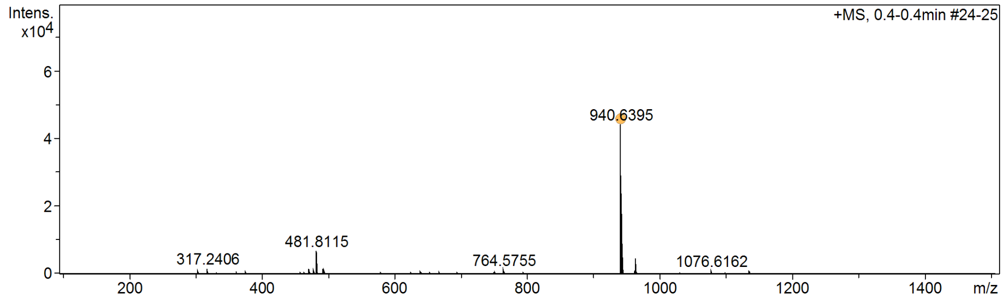


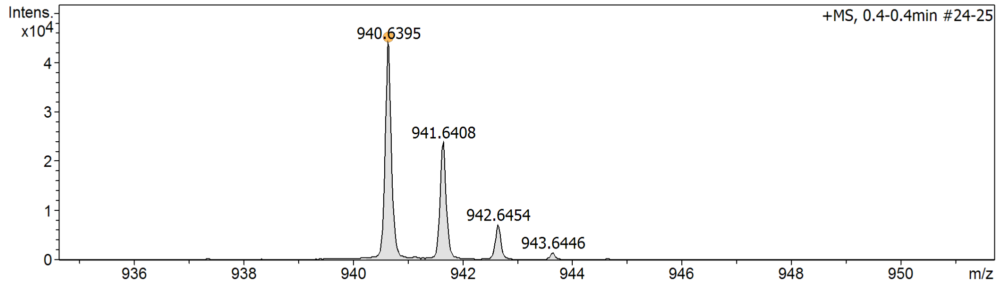


| Meas. m/z | # | Ion Formula | m/z | err [ppm] | mSigma | # Sigma | Score | rdb | e⁻ Conf | N-Rule |
|---|---|---|---|---|---|---|---|---|---|---|
| 940.6395 | 1 | C48H82N11O8 | 940.6342 | -5.6 | 15.4 | 3 | 1.24 | 13.5 | even | ok |

**Figure S62**. HRMS measurement for synthetic surugamide S2 (**10**).

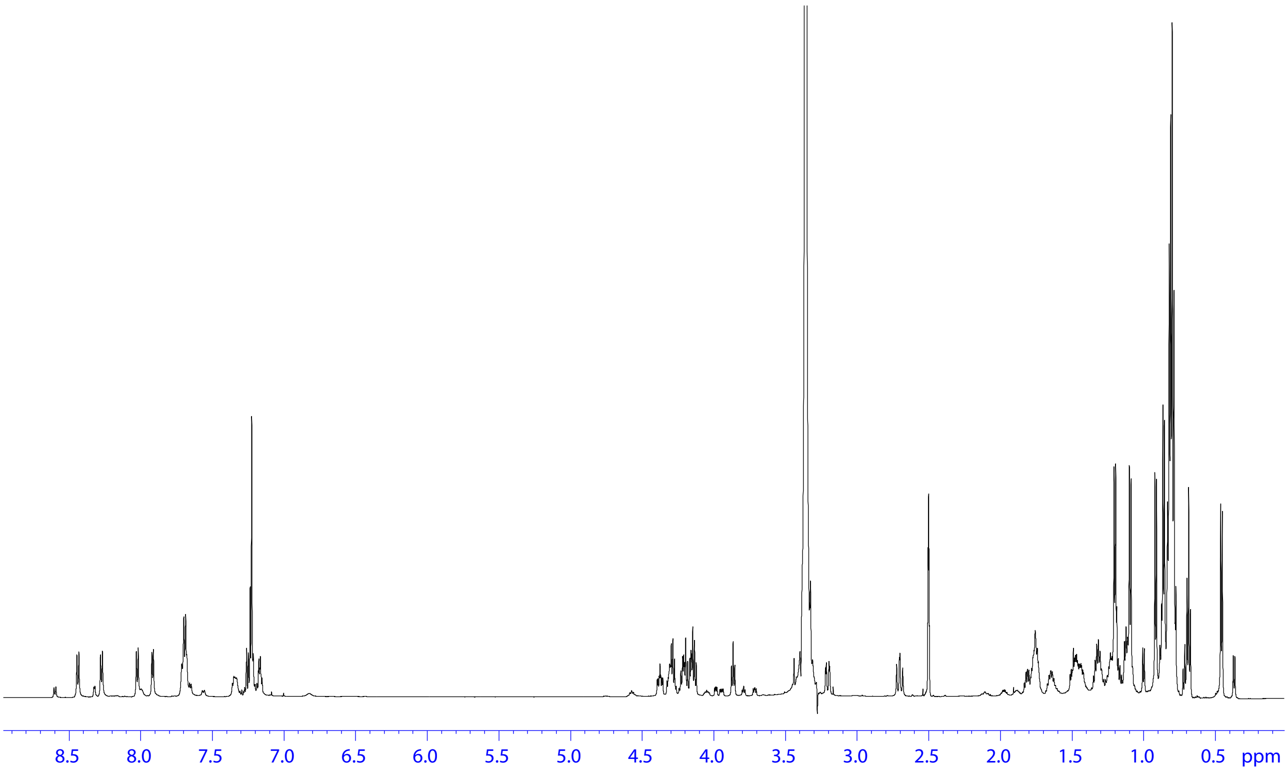


**Figure S63**. $^{1}$H NMR (600 MHz, DMSO-$d_6$) spectrum of synthetic surugamide S3 (**11**)

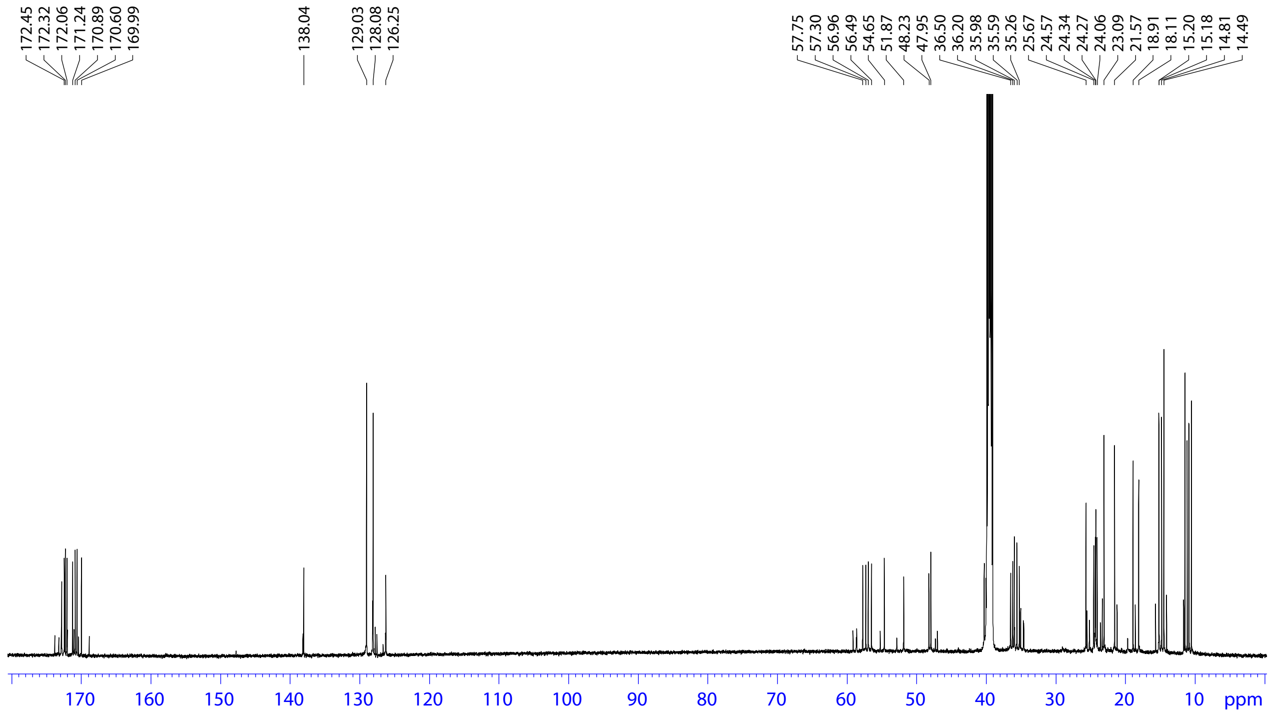


**Figure S64**. $^{13}$C NMR (150 MHz, DMSO-$d_6$) spectrum of synthetic surugamide S3 (**11**)

# Mass Spectrum Molecular Formula Report

**Analysis Info**

| | | | |
|---|---|---|---|
| Analysis Name | D:\Data\Taizong\Ala Surugamide A allo.d | Acquisition Date | 6/1/2023 10:18:24 AM |
| Method | tune-medhigh_AP.m | Operator | a.salim |
| Sample Name | Ala Surugamide A allo | Instrument / Ser# | micrOTOF 213750.00232 |
| Comment | | | |

**Acquisition Parameter**

| | | | | | |
|---|---|---|---|---|---|
| Source Type | ESI | Ion Polarity | Positive | Set Nebulizer | 0.5 Bar |
| Focus | Not active | | | Set Dry Heater | 180 °C |
| Scan Begin | 100 m/z | Set Capillary | 4500 V | Set Dry Gas | 5.0 l/min |
| Scan End | 1500 m/z | Set End Plate Offset | -500 V | Set Divert Valve | Source |

**Generate Molecular Formula Parameter**

| | | | | | |
|---|---|---|---|---|---|
| Formula, min. | | | | | |
| Formula, max. | | | | | |
| Measured m/z | | Tolerance | | Charge | |
| Check Valence | | Minimum | | Maximum | |
| Nirogen Rule | | Electron Configuration | | | |
| Filter H/C Ratio | | Minimum | | Maximum | |
| Estimate Carbon | | | | | |

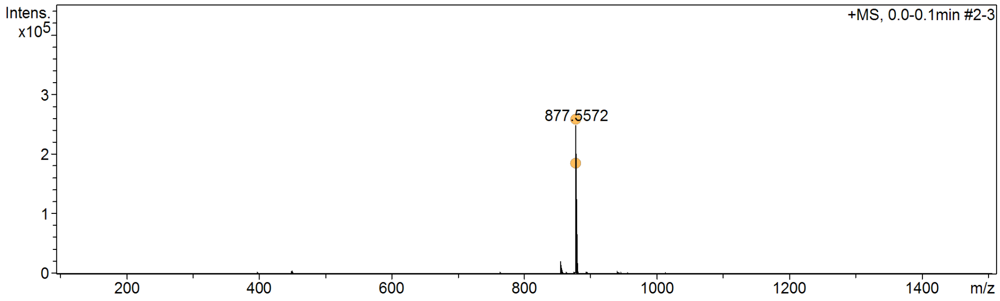


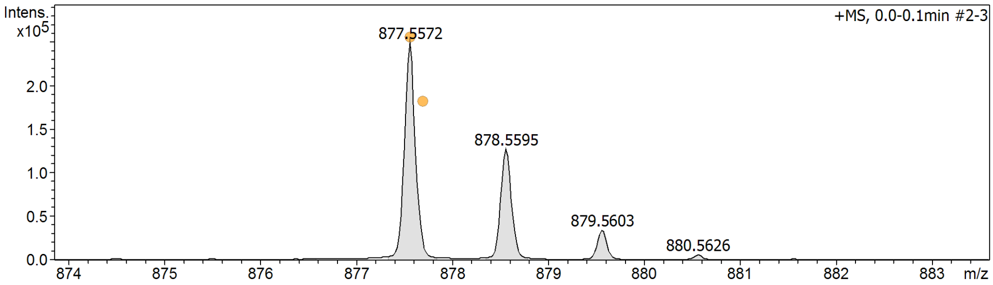


| Meas. m/z | # | Ion Formula | m/z | err [ppm] | mSigma | # Sigma | Score | rdb | e⁻ Conf | N-Rule |
|---|---|---|---|---|---|---|---|---|---|---|
| 877.5572 | 1 | C45H74N8NaO8 | 877.5522 | -5.8 | 12.0 | 1 | 2.21 | 12.5 | even | ok |
| 877.6891 | 1 | C41H89N12O8 | 877.6921 | 3.4 | n.a. | n.a. | n.a. | 3.5 | even | ok |

**Figure S65**. HRMS measurement for synthetic surugamide S3 (**11**).

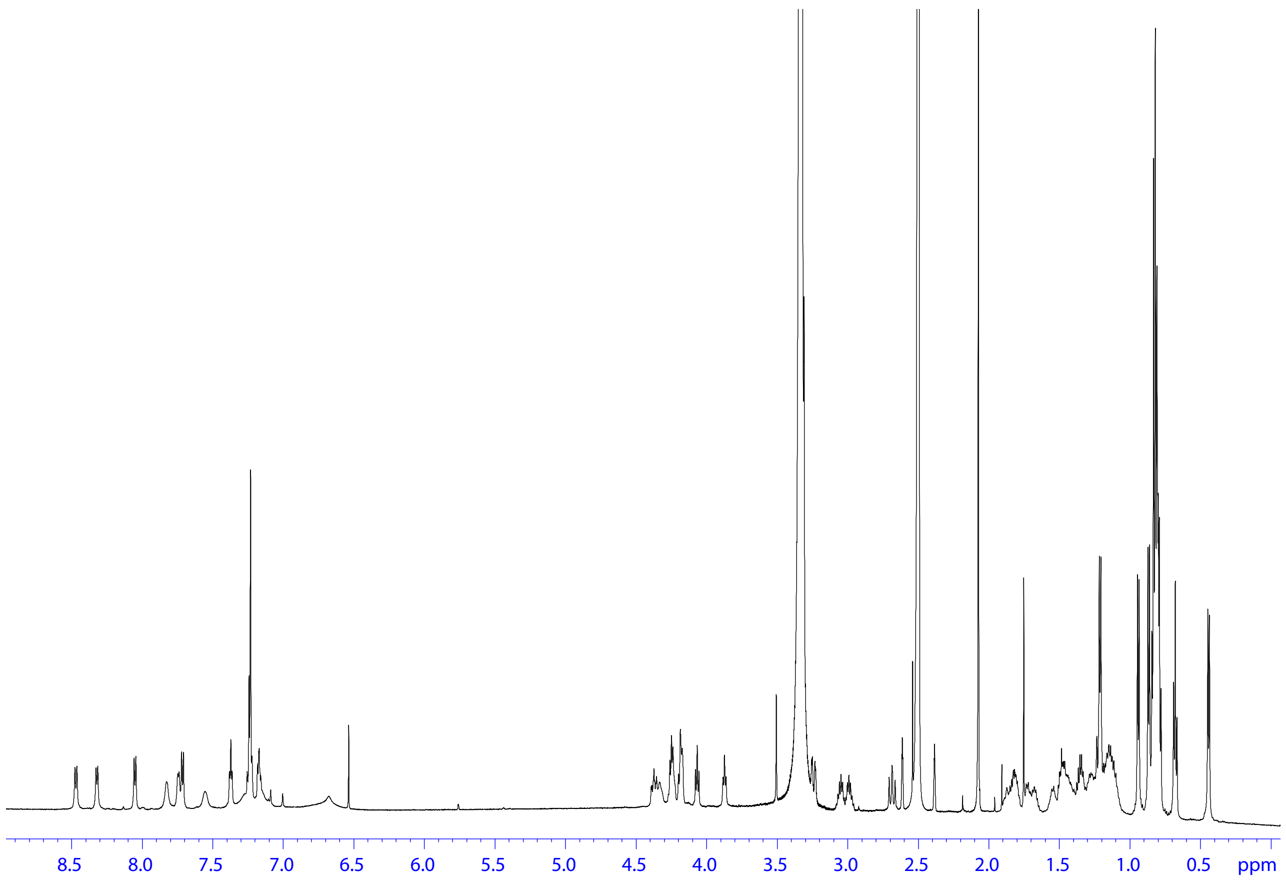


**Figure S66**. $^{1}$H NMR (600 MHz, DMSO-$d_6$) spectrum of semi-synthetic surugamide S4 (**12**)

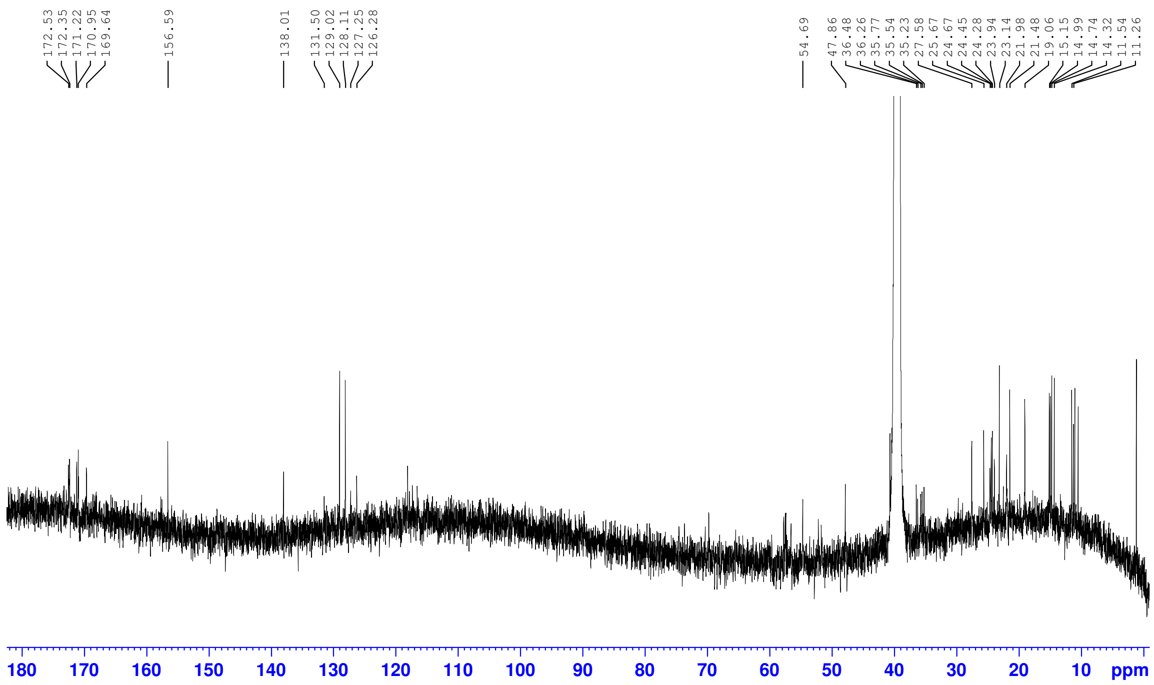


**Figure S67**. $^{13}$C NMR (150 MHz, DMSO-$d_6$) spectrum of semi-synthetic surugamide S4 (**12**)

# Mass Spectrum Molecular Formula Report

**Analysis Info**

| | | | |
|---|---|---|---|
| Analysis Name | D:\Data\Taizong\surugamide A_guanidine.d | Acquisition Date | 9/14/2022 3:23:06 PM |
| Method | tune-medhigh_AP.m | Operator | a.salim |
| Sample Name | surugamide A_guanidine | Instrument / Ser# | micrOTOF 213750.00232 |
| Comment | | | |

**Acquisition Parameter**

| | | | | | |
|---|---|---|---|---|---|
| Source Type | ESI | Ion Polarity | Positive | Set Nebulizer | 0.5 Bar |
| Focus | Not active | | | Set Dry Heater | 180 °C |
| Scan Begin | 100 m/z | Set Capillary | 4500 V | Set Dry Gas | 5.0 l/min |
| Scan End | 1500 m/z | Set End Plate Offset | -500 V | Set Divert Valve | Source |

**Generate Molecular Formula Parameter**

| | | |
|---|---|---|
| Formula, min. | | |
| Formula, max. | | |
| Measured m/z | Tolerance | Charge |
| Check Valence | Minimum | Maximum |
| Nirogen Rule | Electron Configuration | |
| Filter H/C Ratio | Minimum | Maximum |
| Estimate Carbon | | |

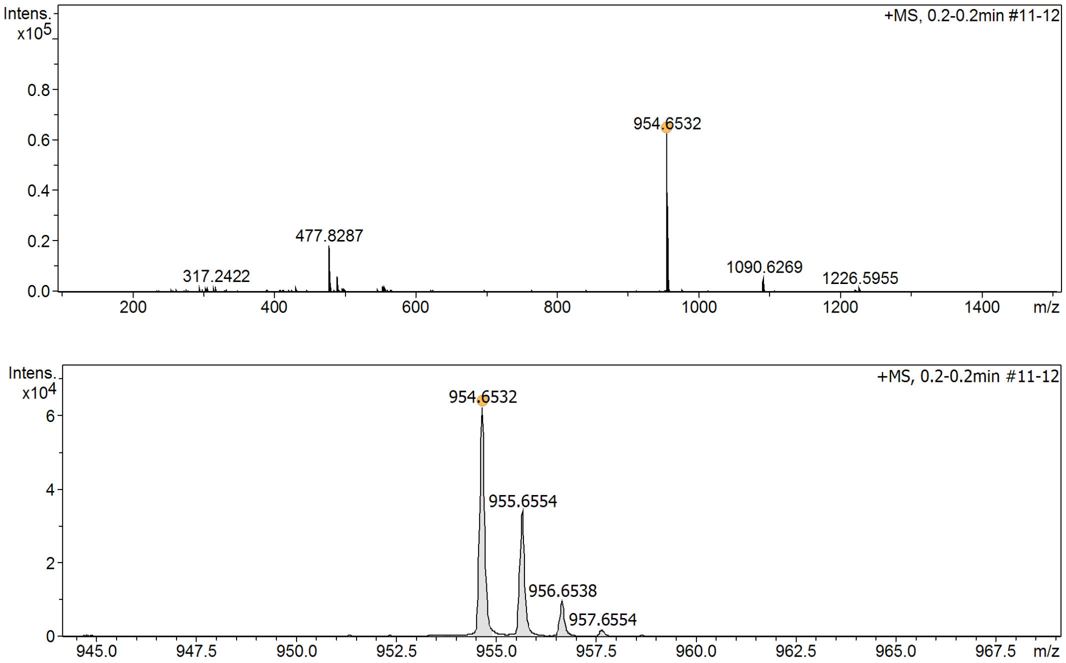


| Meas. m/z | # | Ion Formula | m/z | err [ppm] | mSigma | # Sigma | Score | rdb | e⁻ Conf | N-Rule |
|---|---|---|---|---|---|---|---|---|---|---|
| 954.6532 | 1 | C49H84N11O8 | 954.6499 | 3.5 | 22.2 | 6 | 40.52 | 13.5 | even | ok |

Bruker Compass DataAnalysis 4.1 printed: 9/14/2022 3:37:32 PM Page 1 of 1

**Figure S68**. HRMS measurement for semi-synthetic surugamide S4 (**12**).

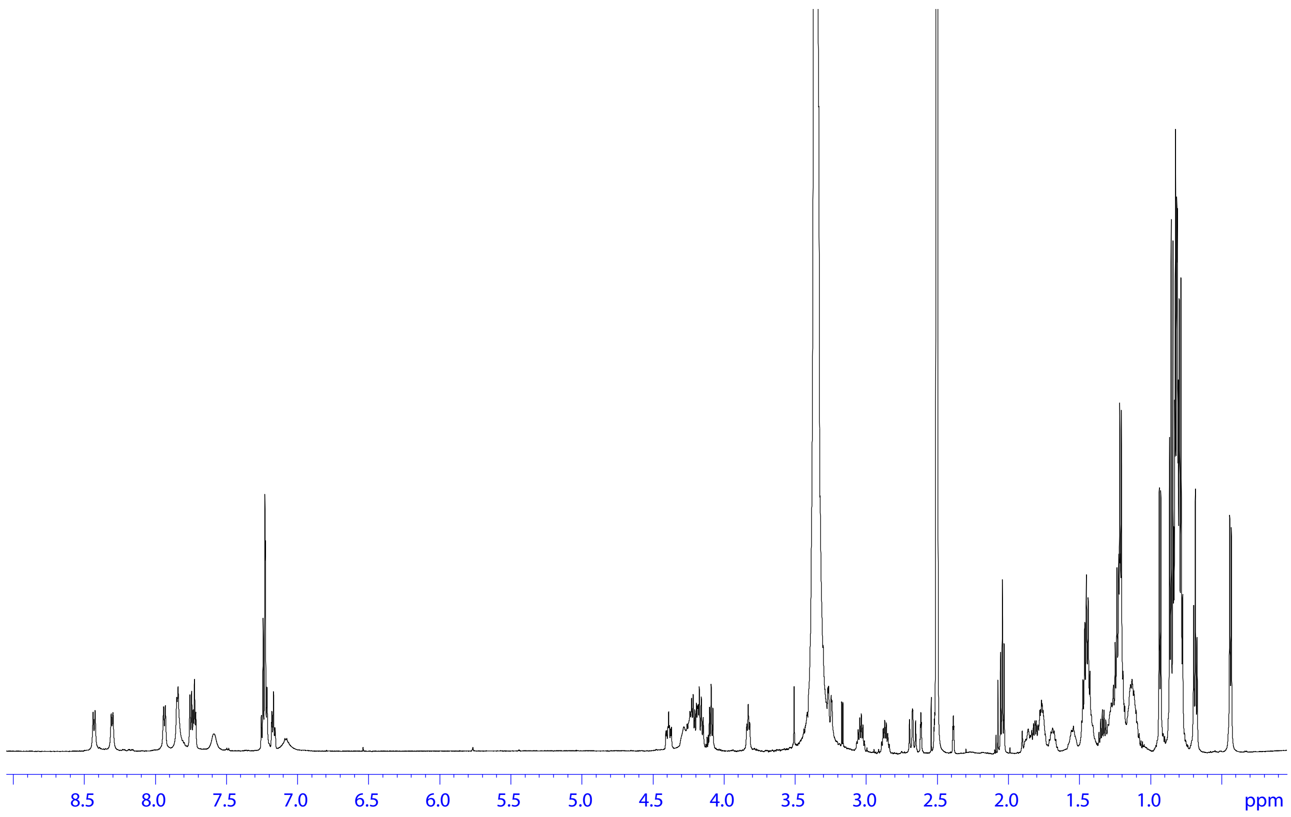


**Figure S69**. $^{1}$H NMR (600 MHz, DMSO-$d_6$) spectrum of semi-synthetic acyl-surugamide AS1 (**13**)

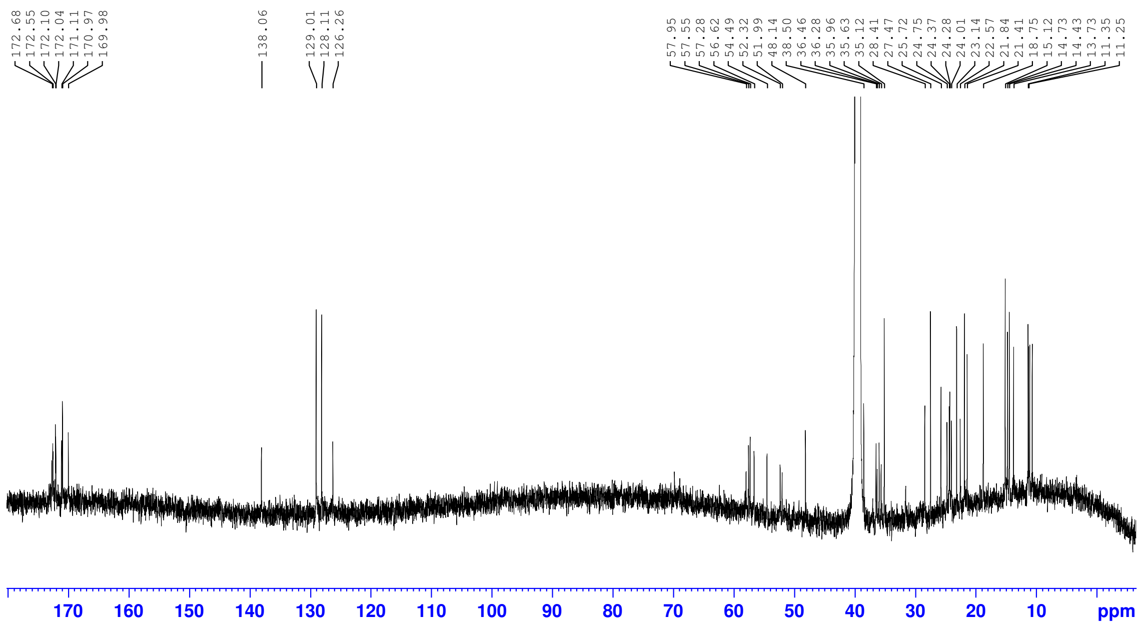


**Figure S70**. $^{13}$C NMR (150 MHz, DMSO-$d_6$) spectrum of semi-synthetic acyl-surugamide AS1 (**13**)

# Mass Spectrum Molecular Formula Report

**Analysis Info**

| | | | | |
|---|---|---|---|---|
| Analysis Name | D:\Data\Taizong\M0112_F20_24_valeric acid_V17_19.d | Acquisition Date | 11/23/2022 12:46:00 PM | |
| Method | tune-medhigh_AP.m | Operator | a.salim | |
| Sample Name | M0112_F20_24_valeric acid_V17_19 | Instrument / Ser# | micrOTOF | 213750.00232 |
| Comment | | | | |

**Acquisition Parameter**

| | | | | | |
|---|---|---|---|---|---|
| Source Type | ESI | Ion Polarity | Positive | Set Nebulizer | 0.5 Bar |
| Focus | Not active | | | Set Dry Heater | 180 °C |
| Scan Begin | 100 m/z | Set Capillary | 4500 V | Set Dry Gas | 5.0 l/min |
| Scan End | 1500 m/z | Set End Plate Offset | -500 V | Set Divert Valve | Source |

**Generate Molecular Formula Parameter**

| | | |
|---|---|---|
| Formula, min. | | |
| Formula, max. | | |
| Measured m/z | Tolerance | Charge |
| Check Valence | Minimum | Maximum |
| Nirogen Rule | Electron Configuration | |
| Filter H/C Ratio | Minimum | Maximum |
| Estimate Carbon | | |

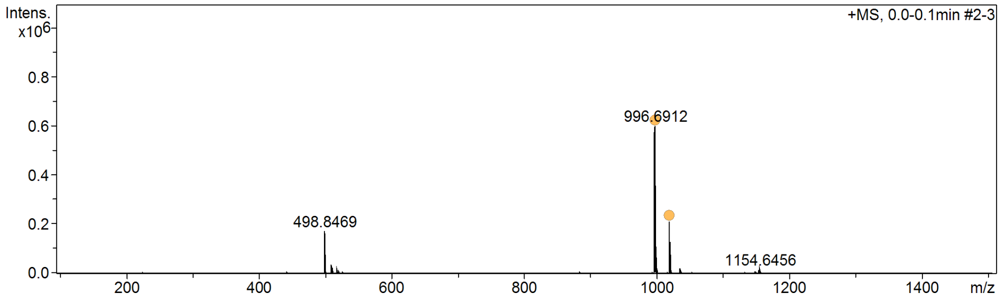


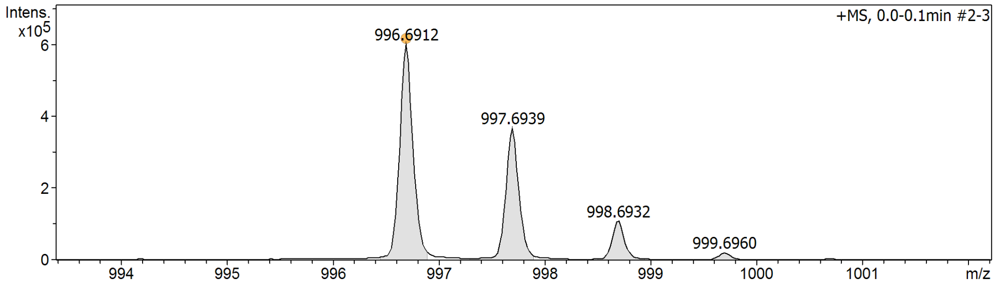


| Meas. m/z | # | Ion Formula | m/z | err [ppm] | mSigma | # Sigma | Score | rdb | e⁻ Conf | N-Rule |
|---|---|---|---|---|---|---|---|---|---|---|
| 996.6912 | 1 | C53H90N9O9 | 996.6856 | -5.6 | 15.1 | 1 | 1.42 | 13.5 | even | ok |
| | 2 | C48H90N11O11 | 996.6816 | -9.6 | 16.9 | 2 | 0.00 | 9.5 | even | ok |
| | 3 | C47H90N13O10 | 996.6928 | 1.7 | 18.3 | 3 | 100.00 | 9.5 | even | ok |

**Figure S71**. HRMS measurement for semi-synthetic acyl-surugamide AS1 (**13**)

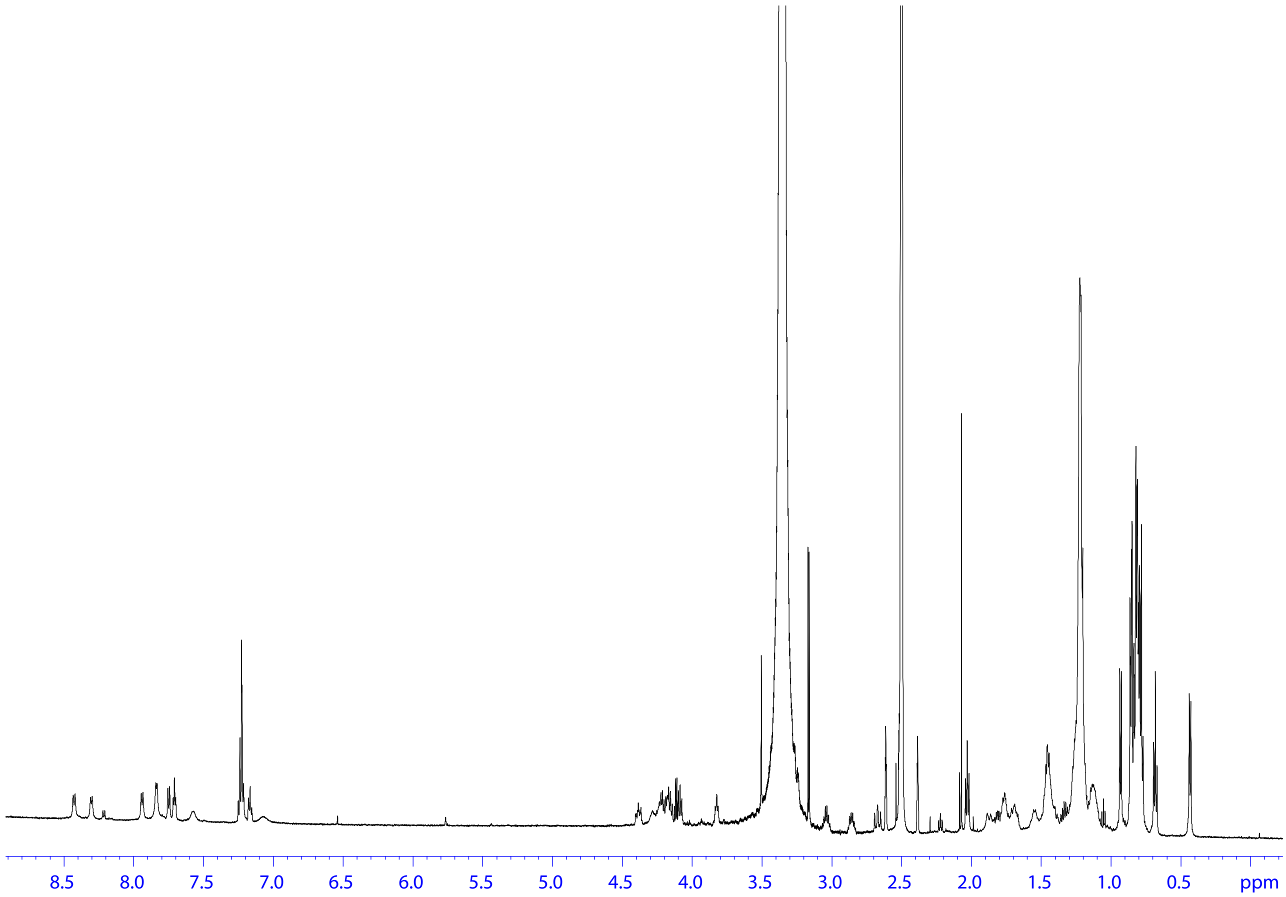


**Figure S72**. $^{1}$H NMR (600 MHz, DMSO-$d_6$) spectrum of semi-synthetic acyl-surugamide AS2 (**14**)

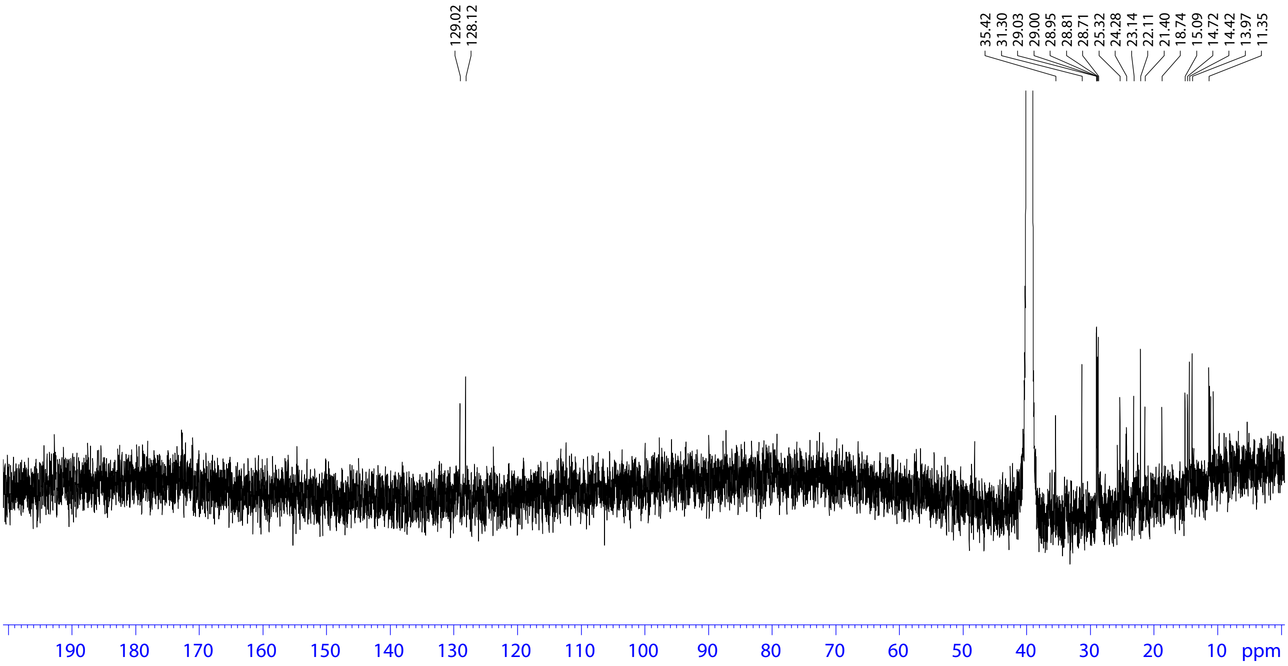


**Figure S73**. $^{13}$C NMR (150 MHz, DMSO-$d_6$) spectrum of semi-synthetic acyl-surugamide AS2 (**14**)

# Mass Spectrum Molecular Formula Report

**Analysis Info**

| | | | |
|---|---|---|---|
| Analysis Name | D:\Data\Taizong\M0112_F20_24_lauric acid_V8_10.d | Acquisition Date | 11/23/2022 8:46:52 PM |
| Method | tune-medhigh_AP.m | Operator | a.salim |
| Sample Name | M0112_F20_24_lauric acid_V8_10 | Instrument / Ser# | micrOTOF 213750.00232 |
| Comment | | | |

**Acquisition Parameter**

| | | | | | |
|---|---|---|---|---|---|
| Source Type | ESI | Ion Polarity | Positive | Set Nebulizer | 0.5 Bar |
| Focus | Not active | | | Set Dry Heater | 180 °C |
| Scan Begin | 100 m/z | Set Capillary | 4500 V | Set Dry Gas | 5.0 l/min |
| Scan End | 1500 m/z | Set End Plate Offset | -500 V | Set Divert Valve | Source |

**Generate Molecular Formula Parameter**

| | | |
|---|---|---|
| Formula, min. | | |
| Formula, max. | | |
| Measured m/z | Tolerance | Charge |
| Check Valence | Minimum | Maximum |
| Nirogen Rule | Electron Configuration | |
| Filter H/C Ratio | Minimum | Maximum |
| Estimate Carbon | | |

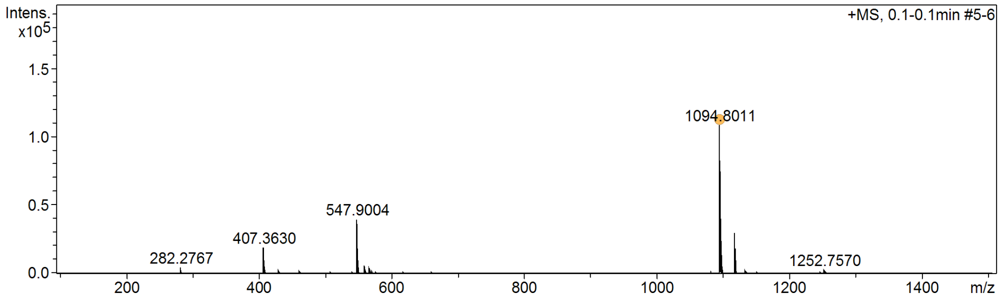


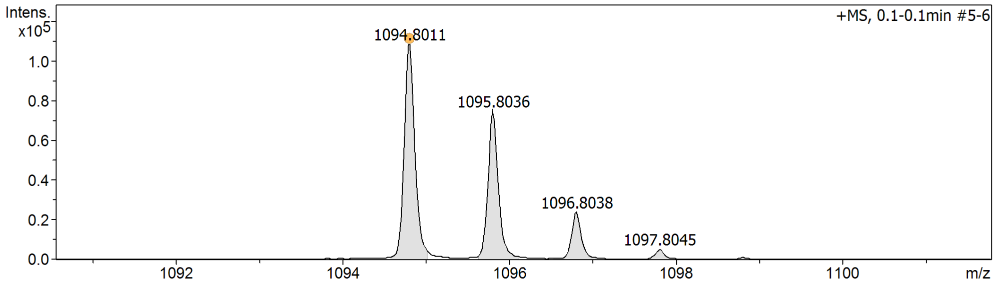


| Meas. m/z | # | Ion Formula | m/z | err [ppm] | mSigma | # Sigma | Score | rdb | e¯ Conf | N-Rule |
|---|---|---|---|---|---|---|---|---|---|---|
| 1094.8011 | 1 | C60H104N9O9 | 1094.7952 | -5.5 | 20.5 | 2 | 0.34 | 13.5 | even | ok |

**Figure S74**. HRMS measurement for semi-synthetic acyl-surugamide AS2 (**14**)

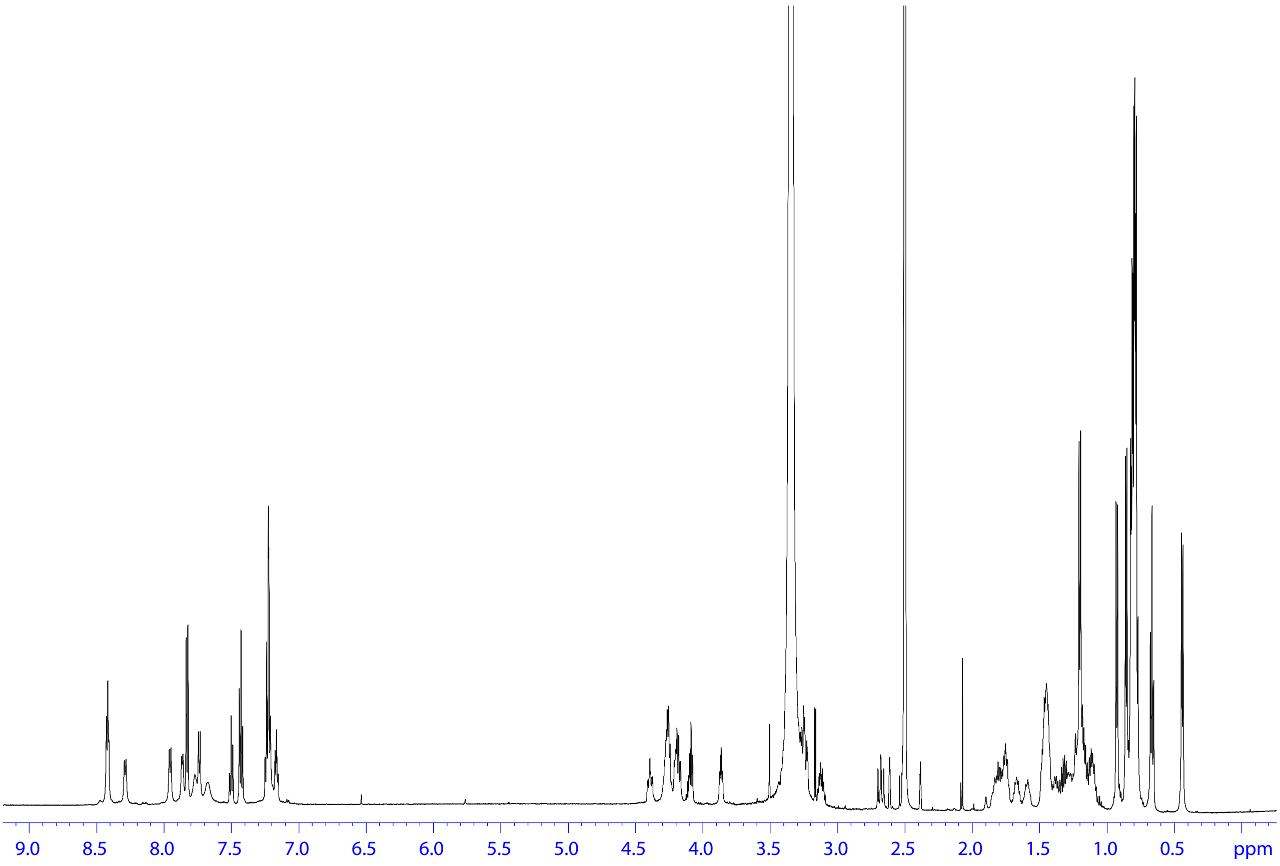


**Figure S75**. $^{1}$H NMR (600 MHz, DMSO-$d_6$) spectrum of semi-synthetic acyl-surugamide AS3 (**15**)

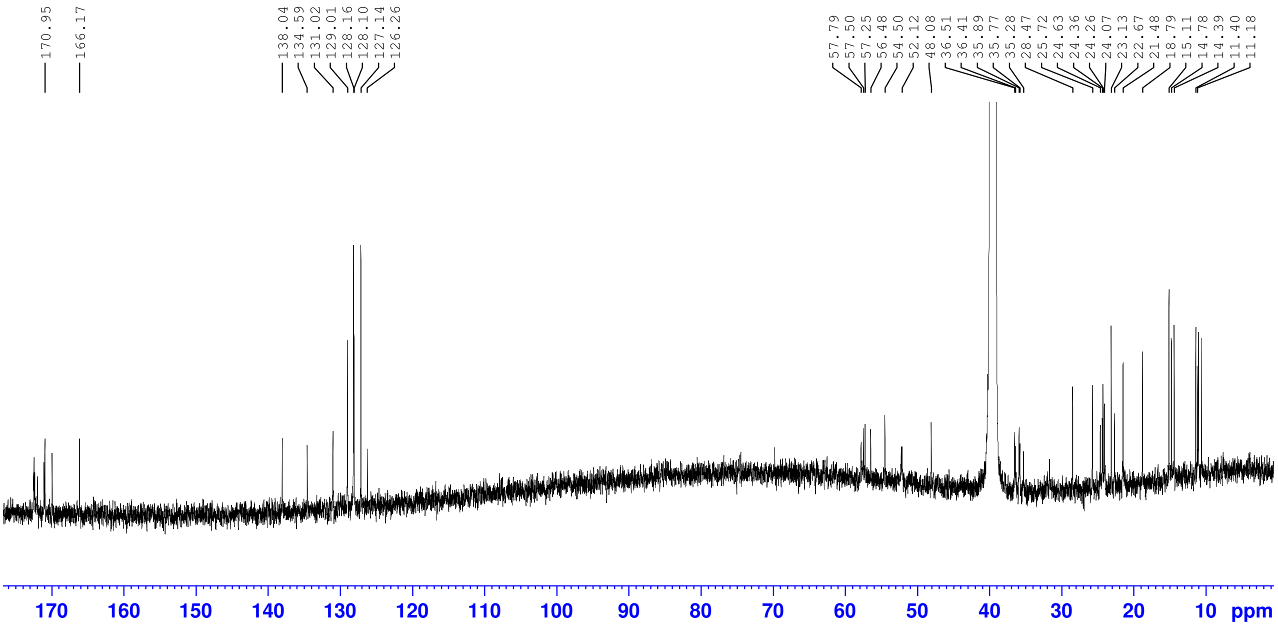


**Figure S76**. $^{13}$C NMR (150 MHz, DMSO-$d_6$) spectrum of semi-synthetic acyl-surugamide AS3 (**15**)

# Mass Spectrum Molecular Formula Report

**Analysis Info**

| | | | |
|---|---|---|---|
| Analysis Name | D:\Data\Taizong\M0112_F20_24_benzoic acid_V14_17.d | Acquisition Date | 11/23/2022 4:27:01 PM |
| Method | tune-medhigh_AP.m | Operator | a.salim |
| Sample Name | M0112_F20_24_benzoic acid_V14_17 | Instrument / Ser# | micrOTOF 213750.00232 |
| Comment | | | |

**Acquisition Parameter**

| | | | | | |
|---|---|---|---|---|---|
| Source Type | ESI | Ion Polarity | Positive | Set Nebulizer | 0.5 Bar |
| Focus | Not active | | | Set Dry Heater | 180 °C |
| Scan Begin | 100 m/z | Set Capillary | 4500 V | Set Dry Gas | 5.0 l/min |
| Scan End | 1500 m/z | Set End Plate Offset | -500 V | Set Divert Valve | Source |

**Generate Molecular Formula Parameter**

| | | | | | |
|---|---|---|---|---|---|
| Formula, min. | | | | | |
| Formula, max. | | | | | |
| Measured m/z | | Tolerance | | Charge | |
| Check Valence | | Minimum | | Maximum | |
| Nirogen Rule | | Electron Configuration | | | |
| Filter H/C Ratio | | Minimum | | Maximum | |
| Estimate Carbon | | | | | |

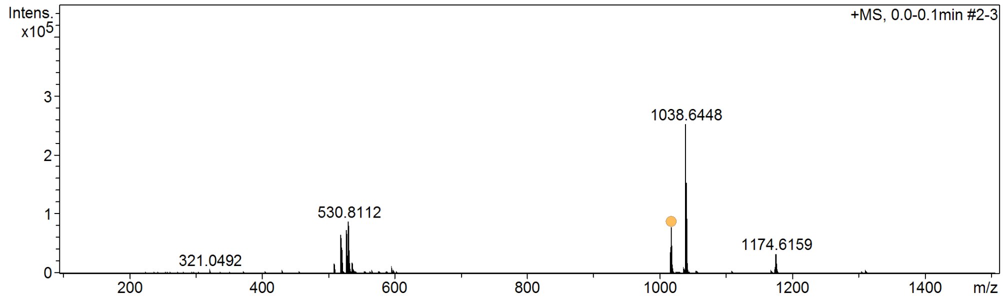


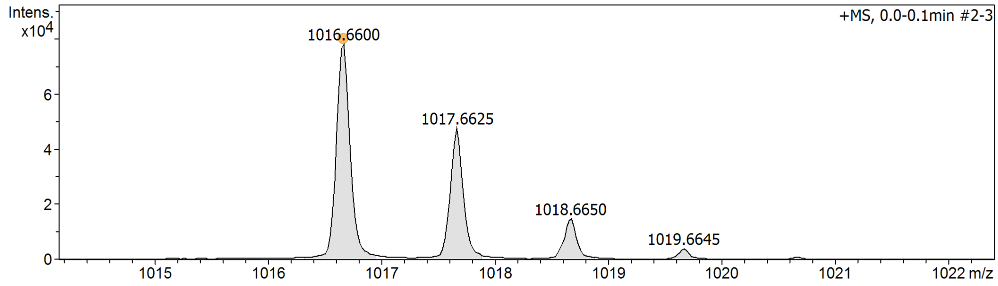


| Meas. m/z | # | Ion Formula | m/z | err [ppm] | mSigma | # Sigma | Score | rdb | e⁻ Conf | N-Rule |
|---|---|---|---|---|---|---|---|---|---|---|
| 1016.6600 | 1 | C55H86N9O9 | 1016.6543 | -5.6 | 21.1 | 4 | 0.48 | 17.5 | even | ok |

**Figure S77**. HRMS measurement for semi-synthetic acyl-surugamide AS3 (**15**).

## 8. Detection of antimycins in crude extracts

(A) QTOF chromatogram of CMB-MRB032 with single ion extractions (SIE) for surugamide A and antimycins

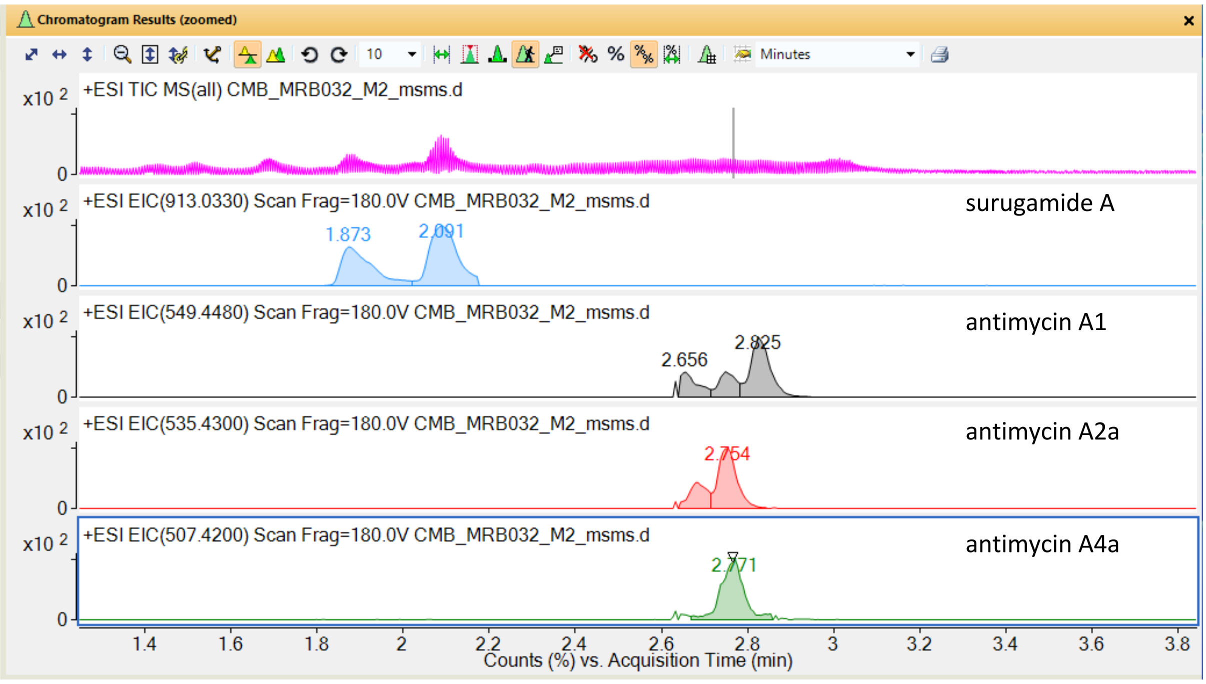


(B) QTOF chromatogram of CMB-M0112 with SIE for surugamide A and antimycins

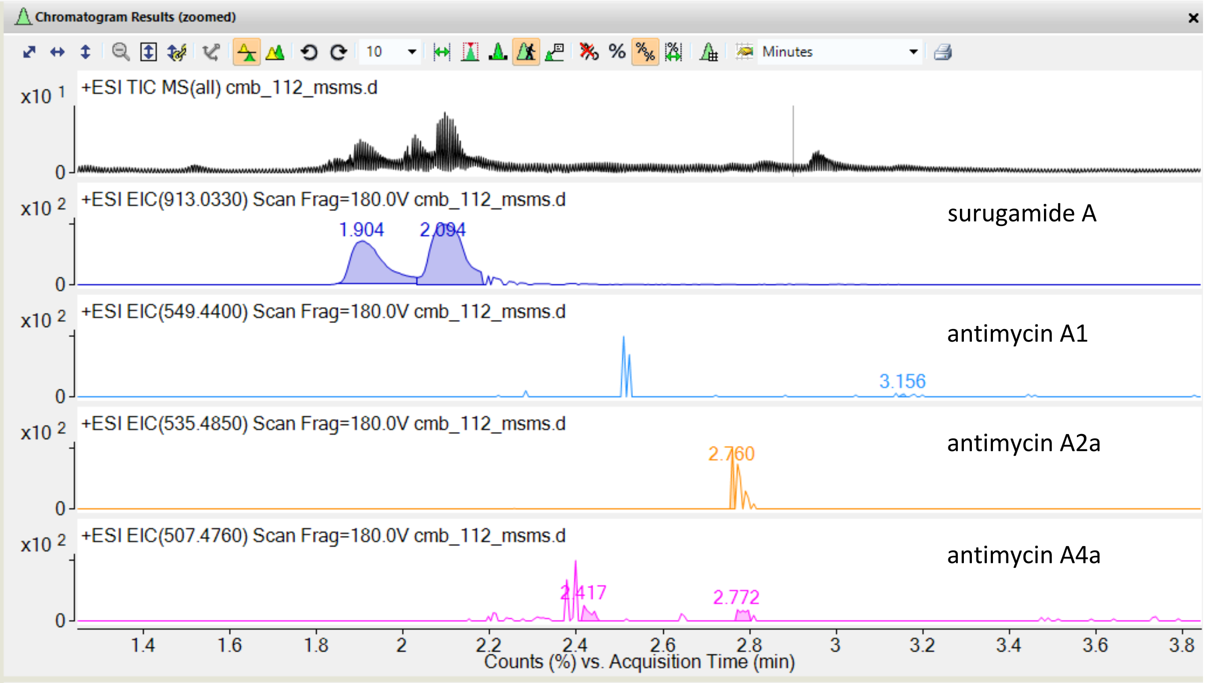

(C) QTOF chromatogram of CMB-CS051 with SIE for surugamide A and antimycins

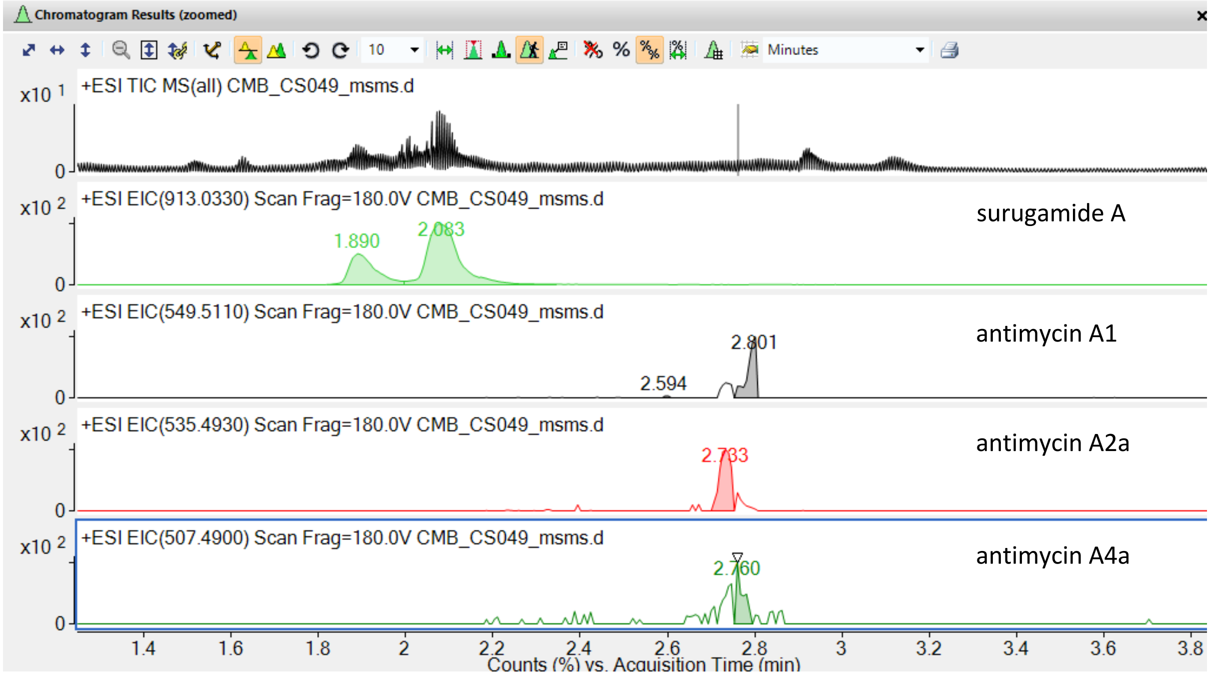


(D) QTOF chromatogram of S4S-00191A07 with SIE for surugamide A and antimycins

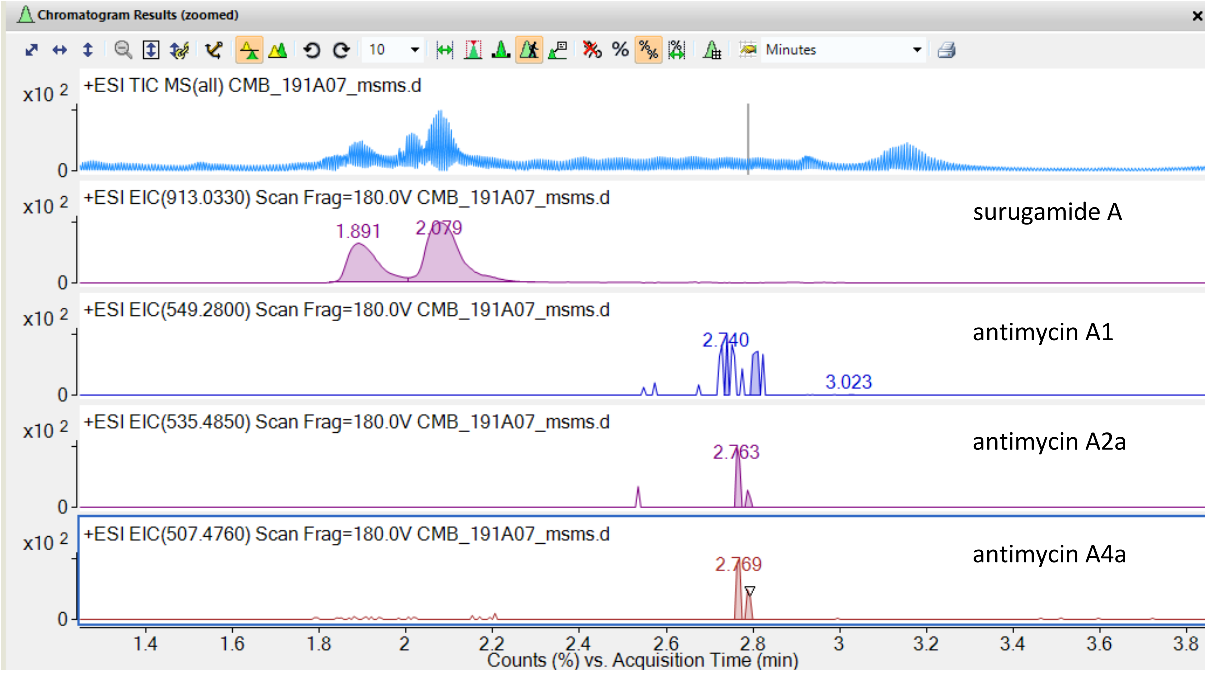


**Figure S78.** Single ion extractions (SIE) for antimycins in CMB-MRB032, CMB-M0112, CMB-CS051, S4S-00191A07 crude extracts

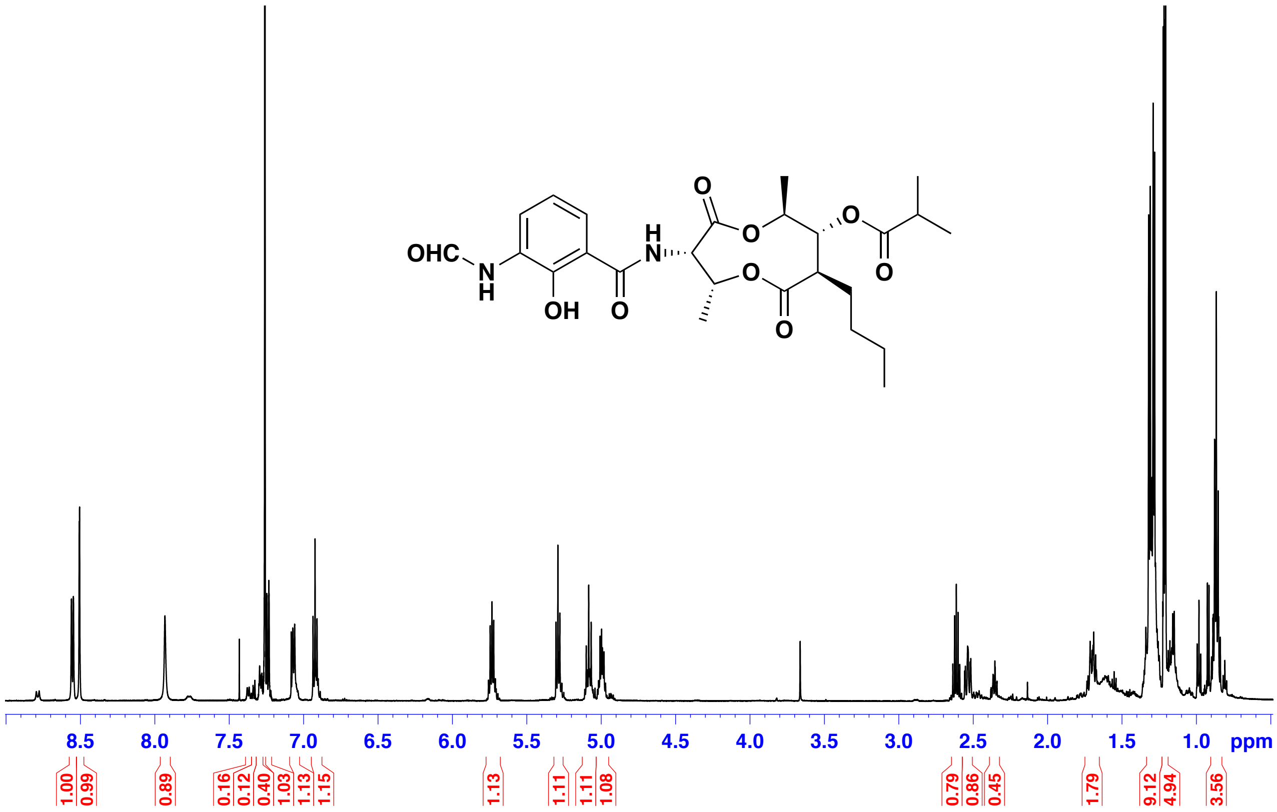

**Figure S79.** $^{1}$H NMR (600 MHz, $CDCl_3$) spectrum of antimycin A4a (**1**)[a]

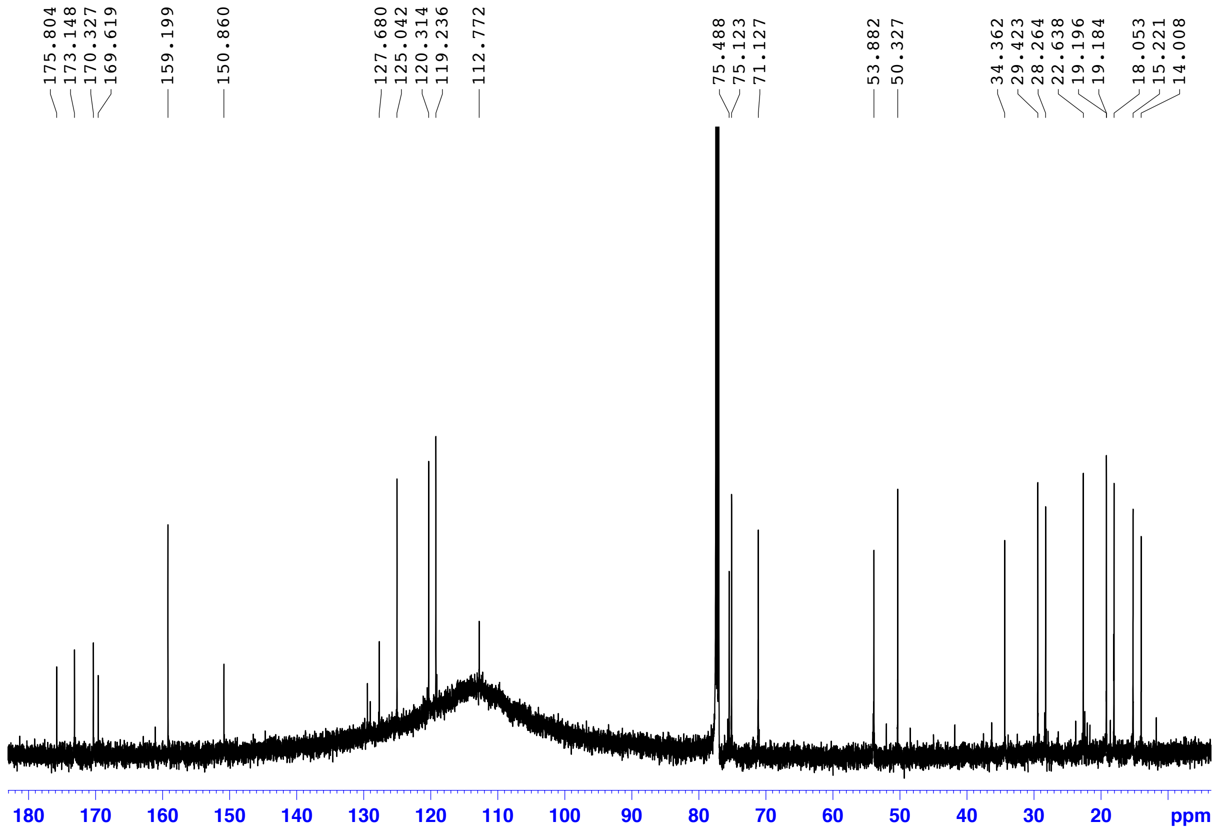

**Figure S80.** $^{13}$C NMR (600 MHz, $CDCl_3$) spectrum of antimycin A4a (**1**)[a]

[a] $^{1}$H and $^{13}$C NMR data match those of literature values (Barrow, C.J., et al. *J. Antibiotics* **1997**, *50*, 729-733; Inai, M., et al. *Eur. Org. Chem.* **2011**, 2719-2729)

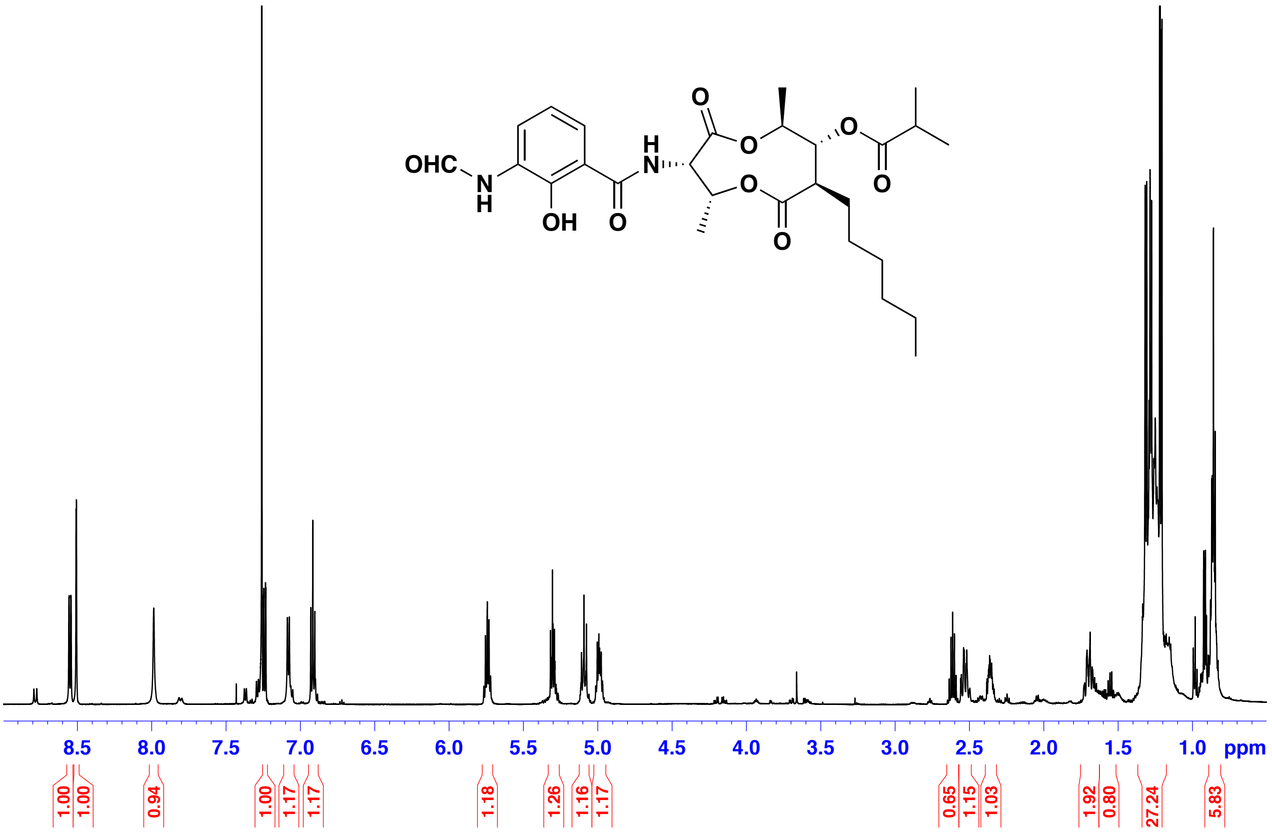


**Figure S81.** $^{1}H$ NMR (600 MHz, $CDCl_3$) spectrum of antimycin A2a (**2**)[a]

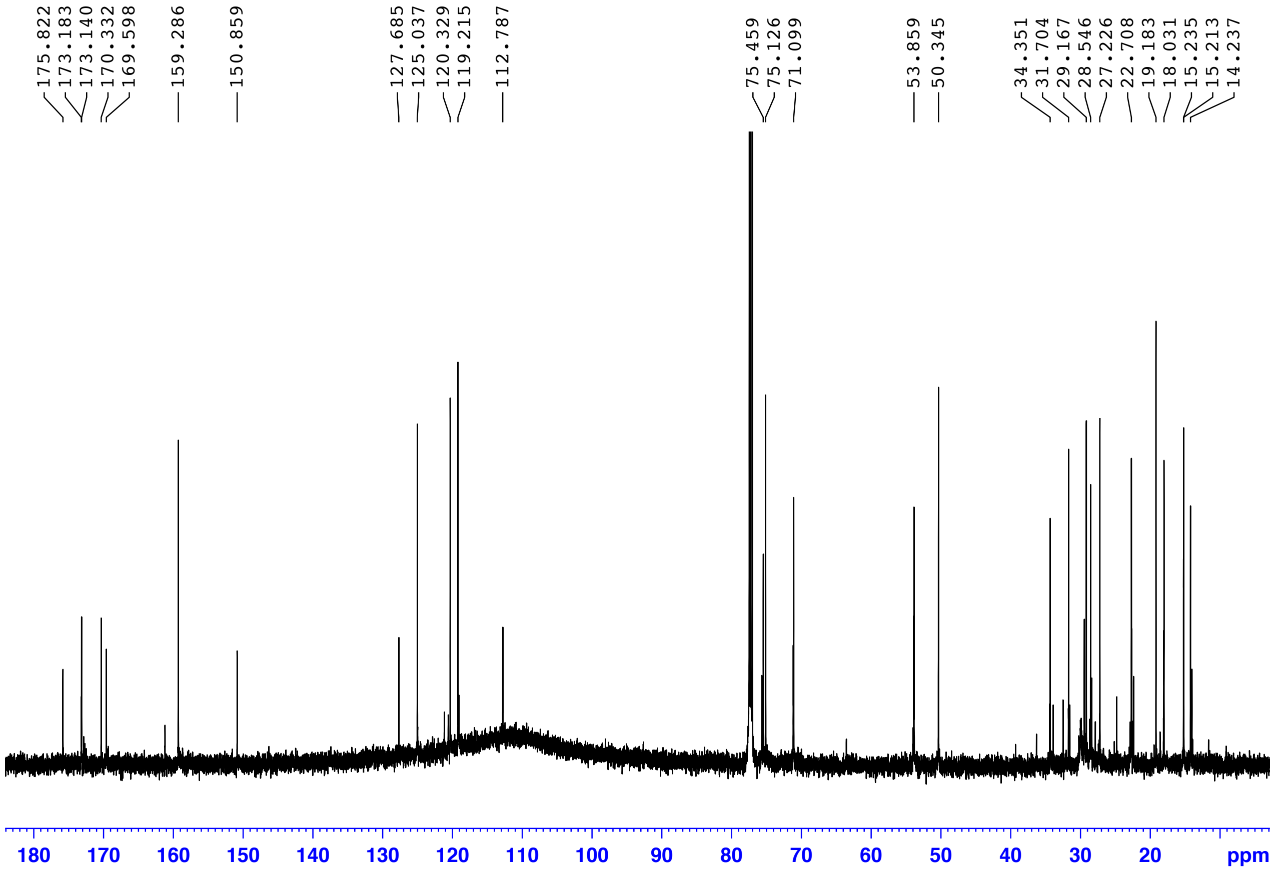


**Figure S82.** $^{13}C$ NMR (600 MHz, $CDCl_3$) spectrum of antimycin A2a (**2**)[a]

[a] $^{1}H$ and $^{13}C$ NMR data match those of literature values (Barrow, C.J., et al. *J. Antibiotics* **1997**, *50*, 729-733; Inai, M., et al. *Eur. Org. Chem.* **2011**, 2719-2729)

## 9. Bioassay results

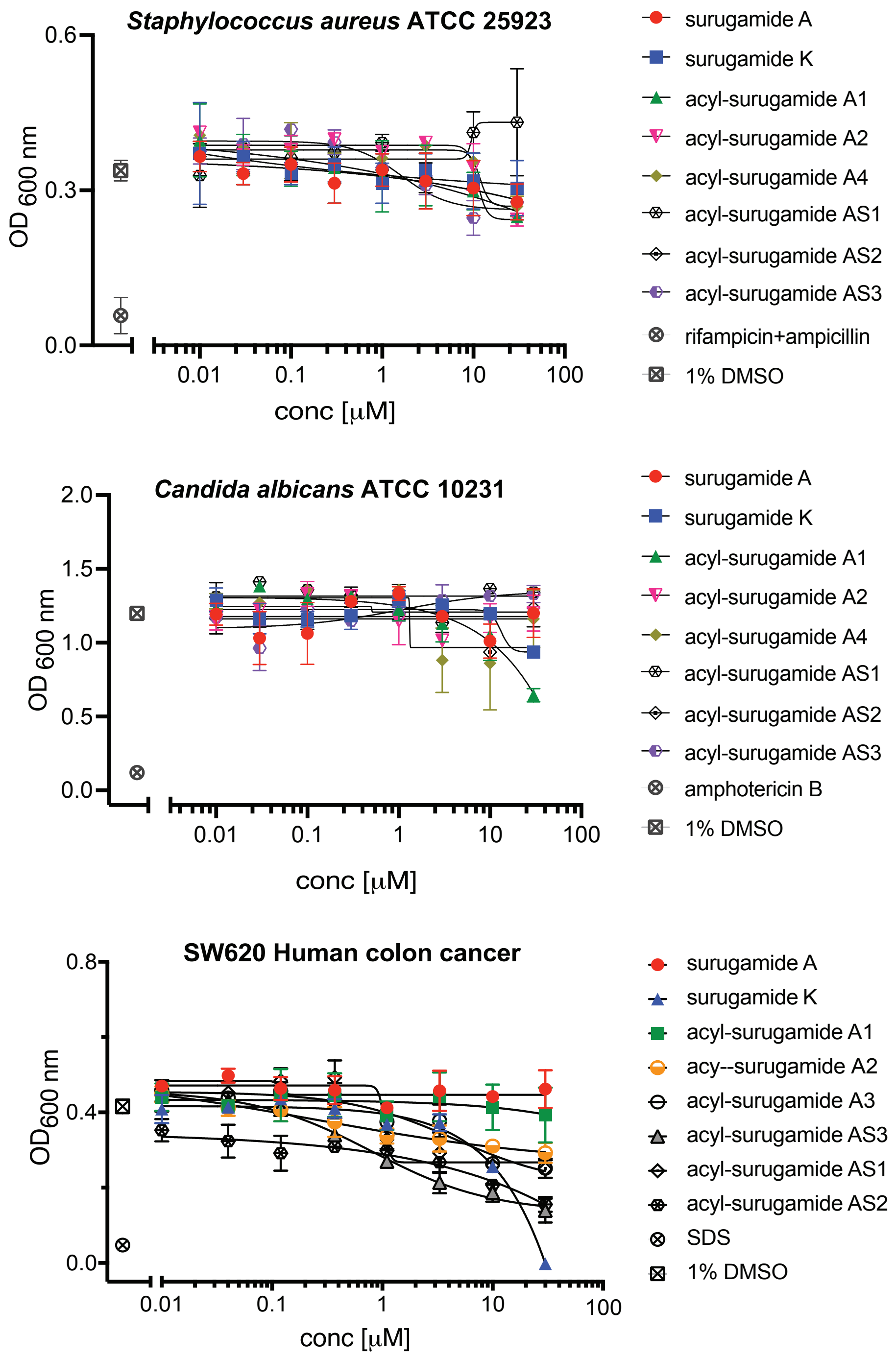


**Figure S83**. Antibacterial and cytotoxicity data for surugamides

## 10. Comparison of champacyclin and surugamide A

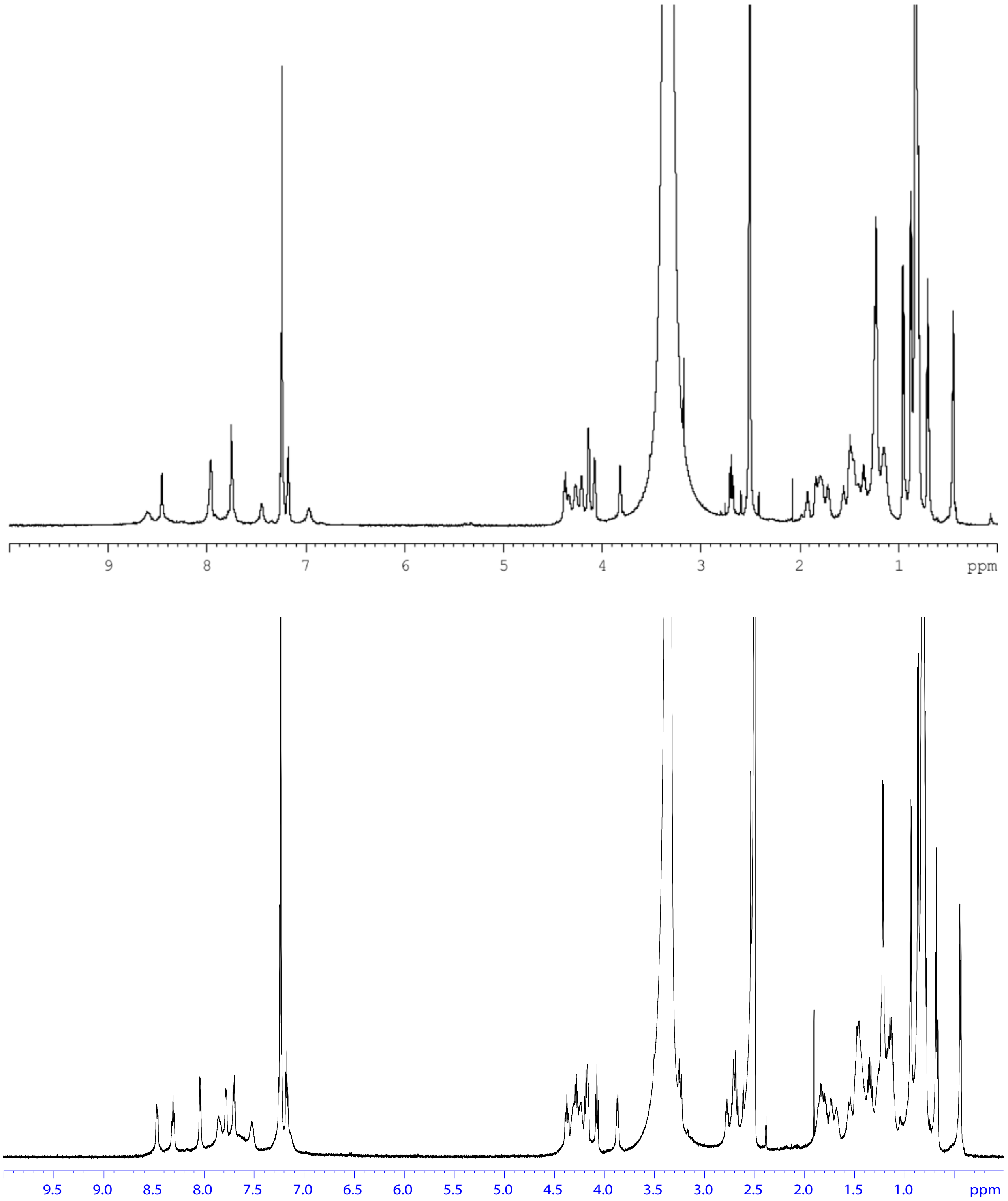


**Figure S84**. $^{1}H$ NMR (600 MHz, DMSO-$d_6$) of champacyclin (top spectrum, adapted from Pesic et al, *Mar Drugs* **2013**, 11, 4834, supplementary materials) and surugamide A from CMB-MRB032 (bottom spectrum).

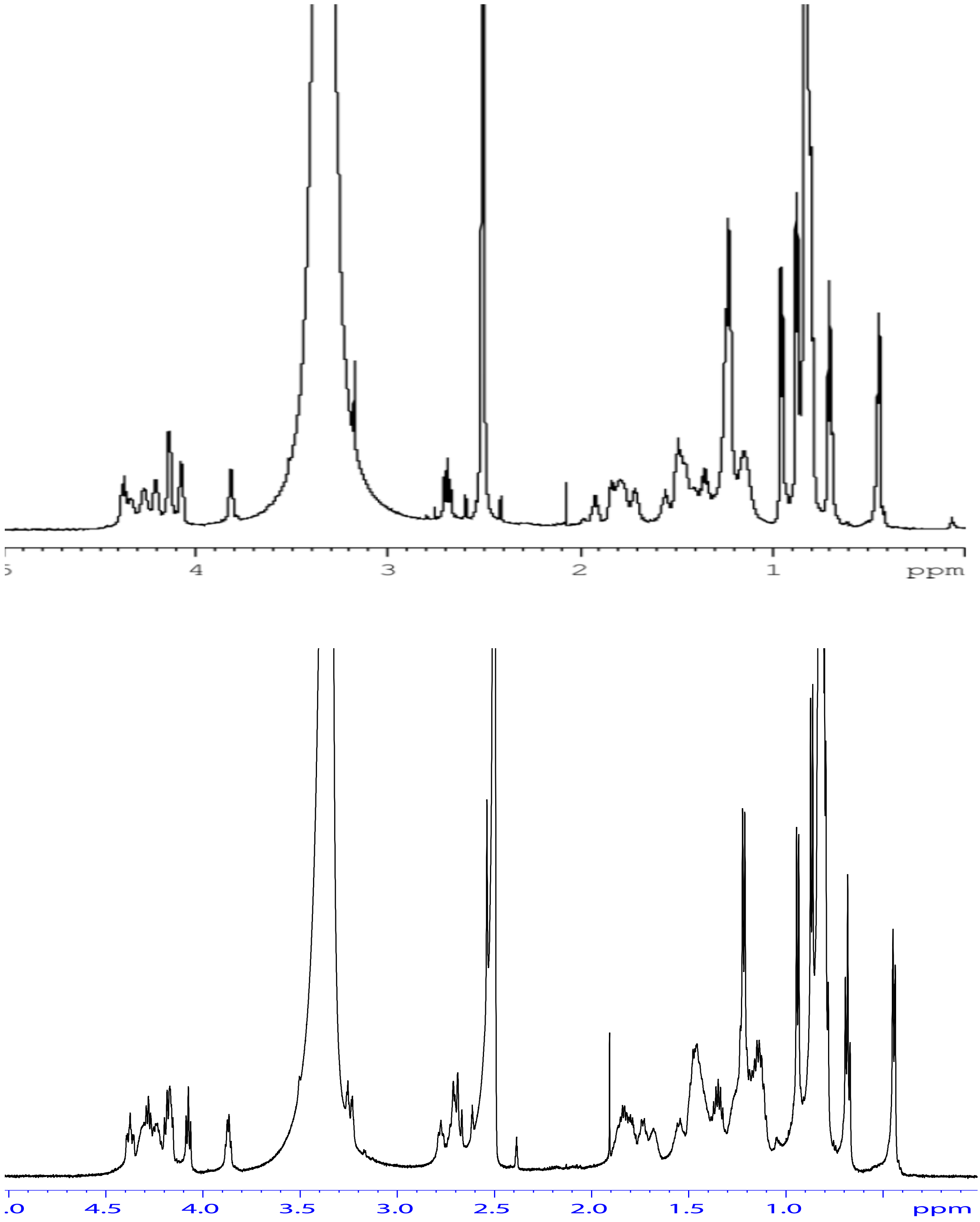


**Figure S85**. Expanded region of $^{1}H$ NMR (600 MHz, DMSO-$d_6$) of champacyclin (top spectrum, adapted from Pesic et al, *Mar Drugs* **2013**, 11, 4834, supplementary materials) and surugamide A from CMB-MRB032 (bottom spectrum).

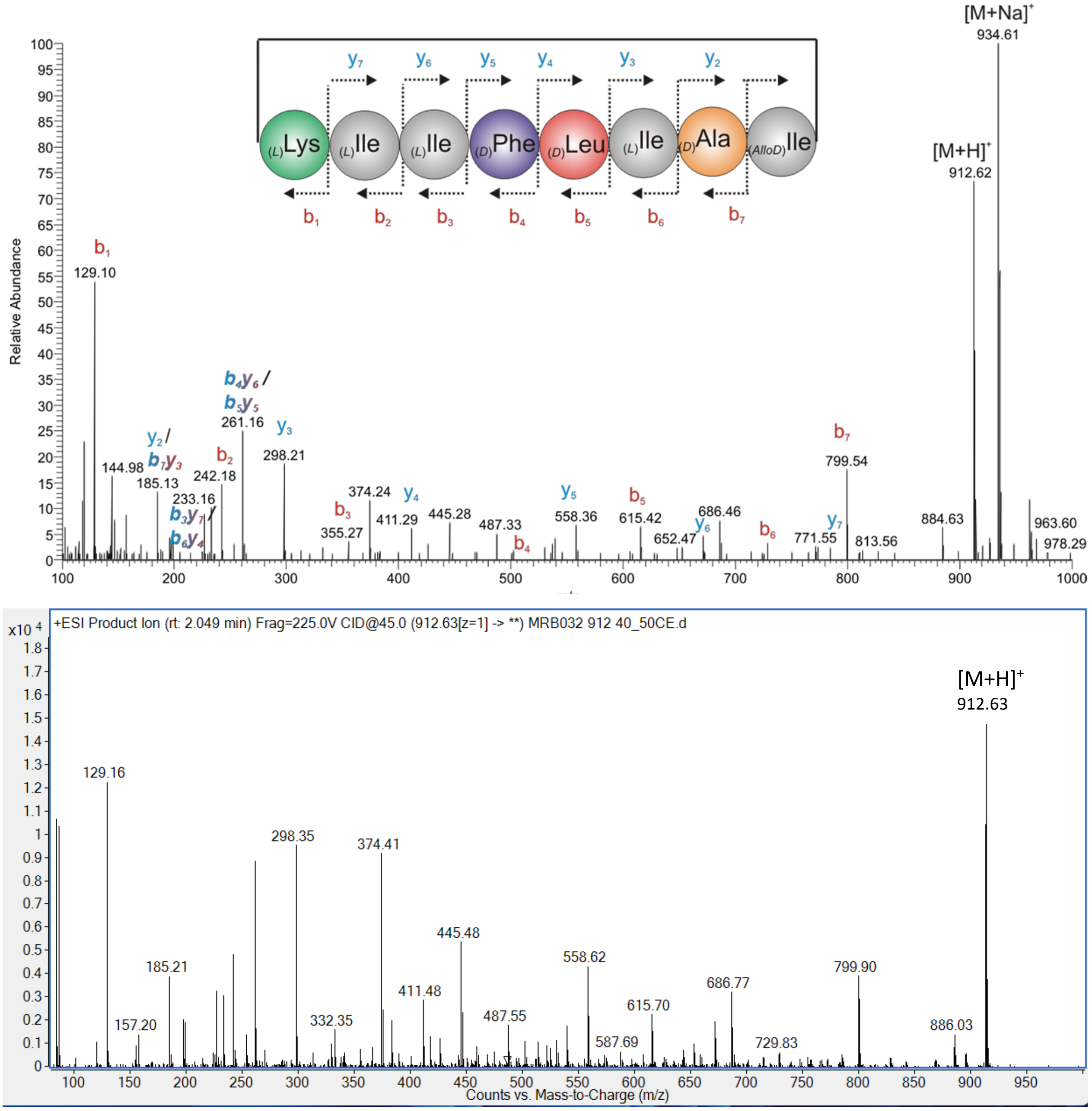


**Figure S86**. MSMS chromatograms of champacyclin (top spectrum, adapted from Pesic et al, *Mar Drugs* **2013**, 11, 4834) and surugamide A from CMB-MRB032 (bottom spectrum). See Figure S47a for the MSMS annotated spectrum of surugamide A.
